# Glioblastoma: Current Status, Emerging Targets, and Recent Advances

**DOI:** 10.1021/acs.jmedchem.1c01946

**Published:** 2022-07-05

**Authors:** Amandeep Thakur, Chetna Faujdar, Ram Sharma, Sachin Sharma, Basant Malik, Kunal Nepali, Jing Ping Liou

**Affiliations:** † School of Pharmacy, College of Pharmacy, Taipei Medical University, 250 Wuxing Street, Taipei 11031, Taiwan; ‡ Department of Biotechnology, Jaypee Institute of Information Technology, Noida 201307, India; § Department of Sterile Product Development, Research and Development-Unit 2, Jubiliant Generics Ltd., Noida 201301, India

## Abstract

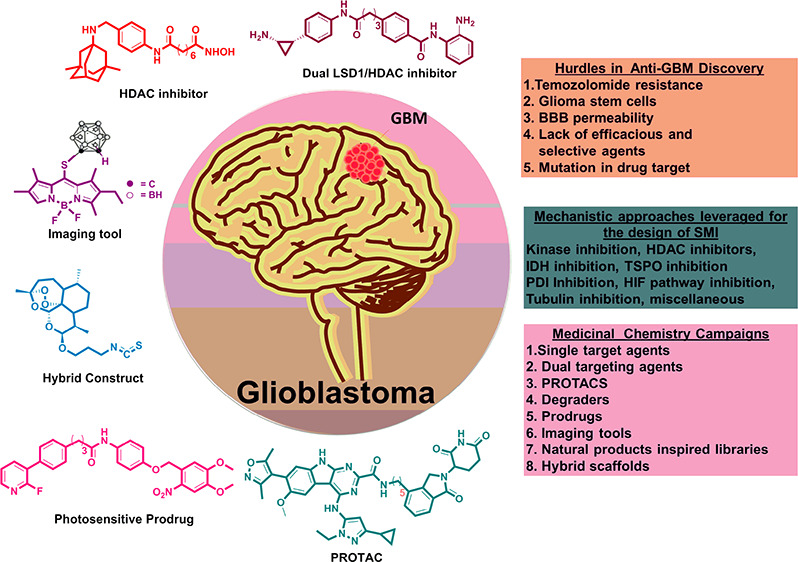

Glioblastoma (GBM) is a highly malignant brain tumor characterized by a heterogeneous population of genetically unstable and highly infiltrative cells that are resistant to chemotherapy. Although substantial efforts have been invested in the field of anti-GBM drug discovery in the past decade, success has primarily been confined to the preclinical level, and clinical studies have often been hampered due to efficacy-, selectivity-, or physicochemical property-related issues. Thus, expansion of the list of molecular targets coupled with a pragmatic design of new small-molecule inhibitors with central nervous system (CNS)-penetrating ability is required to steer the wheels of anti-GBM drug discovery endeavors. This Perspective presents various aspects of drug discovery (challenges in GBM drug discovery and delivery, therapeutic targets, and agents under clinical investigation). The comprehensively covered sections include the recent medicinal chemistry campaigns embarked upon to validate the potential of numerous enzymes/proteins/receptors as therapeutic targets in GBM.

## 1 Background

Glioblastoma (GBM), defined as a grade IV astrocytoma, is a highly malignant brain tumor^1^ characterized by a heterogeneous population of genetically unstable and highly infiltrative cells that are resistant to chemotherapy. Surgery alone is usually insufficient to treat GBM, and complete surgical resection is not possible because the whole tumor is challenging to remove without damaging normal brain tissue. Considering the cytological heterogeneity of GBM, a commonly employed methodology known as optimal multi-modality treatment involves surgery flanked by chemotherapy and radiotherapy. Despite numerous efforts directed toward establishing optimum treatment programs, GBM patients generally show a poor prognosis and experience tumor progression with high mortality and a median survival of only 12–15 months.^2−10^Table 1 presents the approaches currently used in the clinic to treat GBM.

**Table 1:** Approaches Currently Used in Clinic for the Treatment of GBM

treatment	details
**Surgery**	• Surgical resection is considered to be the backbone of therapy for the management of GBM.^151−154^
	• Significant advancements have been made to safely maximize the extent of resection and the technological tools used by surgeons, including the following:
	(a) intra-operative navigation technology that involves the use of volumetric imaging to locate a lesion/anatomical structure within the surgical field^155^
	(b) electrophysiological monitoring and functional brain mapping based on the use of electrodes to functionally map sensory and motor primary cortical regions and related sub-cortical circuits^155^
	(c) fluorescent markers for maximizing the tumor visualization
	• Current standard therapy is based on maximum surgical removal of the tumor followed by radiotherapy and chemotherapy.^156^

**Chemotherapy**	
temozolomide (TMZ)	• Orally active alkylating agent
	• Approved by the U.S. FDA March 2005
	• Exerts its action via cytosolic conversion of TMZ into 3-methyl(triazen-1-yl)imidazole-4-carboxamide (MTIC) and subsequently methylates DNA guanine bases (N-7 or O-6 position) (Figure 1A)^157^
	• The standard of care therapy for patients with GBM is concomitant adjuvant TMZ chemotherapy and radiotherapy.
	• O-6 methylguanine-DNA methyl transferase (MGMT)-mediated innate resistance to TMZ hinders its therapeutic utility (Figure 1B).^157^
	• Bone marrow suppression, nausea, and emesis are the complications reported.^154,158^

1,3-bis(2-chloroethyl)-1-nitrosourea (BCNU)	• BCNU (also called carmustine, Gliadel wafer) is an alkylating agent approved for the treatment of brain tumors.^159,160^
	• It prevents DNA replication and transcription via formation of interstrand cross-links in DNA.^159^
	• Bone marrow suppression, nausea, and emesis are the complications reported.^154,158,160^

lomustine (CCNU)	• CCNU is another nitrosourea alkylating compound approved for the treatment of recurrent GBM.^158,160^

**Anti-angiogenic Therapy**	
bevacizumab	• Monoclonal antibody directed to the VEGF-A, resulting in downregulation of angiogenesis^161^
	• Approved for the treatment of recurrent GBM by the U.S. FDA in 2009^161^
	• Hypertension is the complication reported.^154,158,161^

**Radiotherapy**	• Therapy using radiation is usually done following surgery. Comparative studies have demonstrated that a combination of surgery and radiation therapy is more effective than surgery alone. If the location of the GBM is not appropriate for surgery, radiotherapy can be considered as the sole treatment approach.^162^
	• TMZ is given along with radiotherapy to increase the sensitivity of the tumor to the radiation.^157^
	• The current standard of care involves fractionated delivery of external beam radiation (60 Gy in 2-Gy fractions over 6 weeks, initially 46 Gy in 2 Gy/fraction followed by a boost plan of 14 Gy in 2 Gy/fraction).^162^
	• For glioma that are located deep in the brain, proton therapy that uses charged particles (protons) instead of the X-rays is employed.^154,158,163^

**Alternating Electric Field Therapy**	
	• Tumor-treating fields represent a new and non-invasive technique based on electrostimulation for GBM, utilizing alternating electrical fields to disrupt tumor growth.^164^
	• The first-generation tumor-treating field device was approved by the U.S. FDA in 2011 for treatment of recurrent GBM.
	• Approved in 2015 as an adjuvant therapy for newly diagnosed GBM^164^

**Figure 1:**
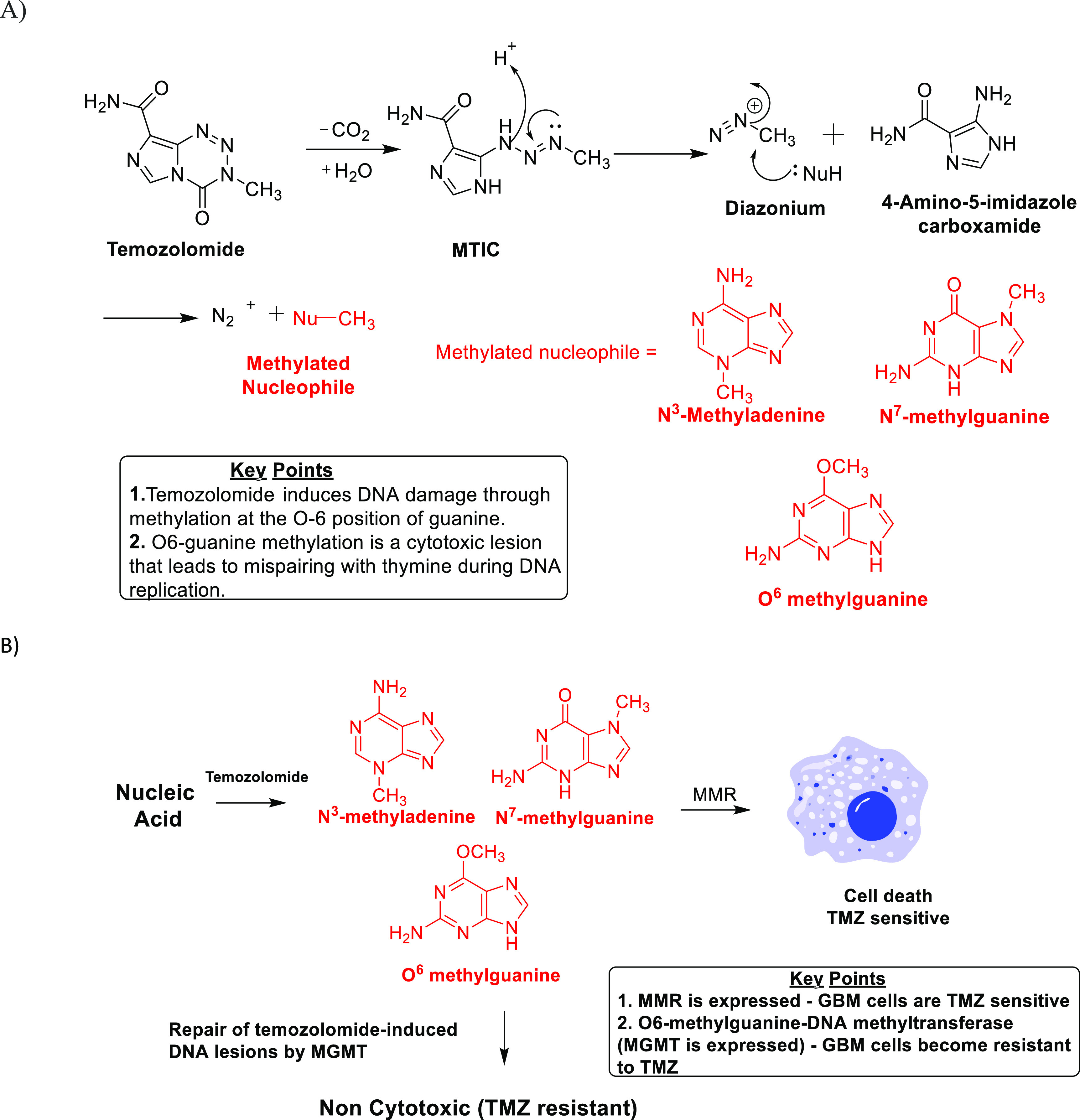
(A) Mechanism of action of TMZ. (B) MGMT-mediated innate resistance to TMZ.

The notorious nature of GBM in the context of resistance to chemotherapy has been a major obstacle during the development stages of efficacious therapy for its treatment. Presently, the anti-GBM drug armory mainly relies on temozolomide (TMZ), an oral alkylating agent, as the first-line chemotherapeutic drug in GBM treatment. TMZ kills cancer cells via guanine/adenine methylation-mediated DNA base pair mismatches and subsequent DNA damage-induced reactive oxygen species (ROS) accumulation (Figure 1A).^11,12^ The literature indicates that methylguanine-DNA methyl transferase (MGMT)-mediated innate resistance to TMZ (a first-line chemotherapeutic GBM drug) is the primary reason for the failure of this GBM treatment (Figure 1B).^13^ However, some studies ascertaining that MGMT expression is silenced in approximately half of GBM patients have revealed that the development of therapeutic resistance is complex in GBM and additional factors are responsible for the development of resistance to TMZ, such as GBM stem cells (GSCs).^13−20^ GSCs represent a small subset of cells within a malignant tumor, known as cancer stem-like cells (CSCs), that demonstrate ability similar to that of normal stem cells and are more resistant to anti-cancer therapeutics than bulk tumor cells.^21,22^ These revelations indicate that CSCs can survive after therapy and become an underlying cause of tumor recurrence.^13−20^ In this context, a search for potential anti-cancer interventions that exert simultaneous disruption of GBM and brain tumor stem cell homeostasis is needed. In addition to TMZ, bevacizumab is approved by the U.S. FDA for the treatment of primary and recurrent GBM; however, the outcome of some studies demonstrates the failure of bevacizumab to prolong overall survival.^23^ Along with overall survival failure, it was found that the administration of bevacizumab led to the overexpression of the receptor tyrosine kinase (RTK) c-Met, thereby causing tumor relapse.^24,25^

In addition to the resistance issue, the obstacles that must be approached involve issues related to delivery to the brain because the existence of the blood–brain barrier (BBB) lowers the efficiency of systemic drug delivery to the target tumor in the brain. Attempts at using dose escalation of drugs to enhance their therapeutic efficiency have often culminated in increased toxicity to normal cells and have elevated the risk of adverse effects. Many efforts toward optimizing drug cocktails (combination therapy) to counter the high genetic heterogeneity of GBM in patients have also proven fruitless, and the limitations were again attributed to the enhanced risk of adverse effects.^26,27^ Additionally, because the central nervous system (CNS) is considered a region for active immunosurveillance, immunotherapy is also being exhaustively explored as a potential strategy for GBM. However, the complex state of a patient’s immune dysfunction in GBM also poses several challenges for immunotherapy.^28^

Previous literature has indicated that, despite demonstrating striking efficacy and selectivity, the progress of small-molecule inhibitors as anti-GBM agents has often been halted by their poor BBB permeability as well as drug resistance issues. To overcome the above obstacles, the prudent design of libraries of mechanistically diverse small-molecule inhibitors comprising lipophilic structural components appears to be a practical step forward. Accordingly, an increasing number of studies have investigated this direction for construction of new assemblages as therapeutic options in GBM. Notably, the experience and intuition of the medicinal chemist play key roles in the design of chemical tools with ideal physicochemical properties to emerge as CNS drugs. Recently, some reviews have been published that presented therapeutic strategies and recent advances in GBM therapy.^29−32^ However, there is an opportunity to assemble a compilation that mainly focuses on robust drug design strategies employed by the medicinal chemist to furnish anti-GBM adducts. Thus, we embarked on the task of compiling a comprehensive review of GBM that primarily focuses on recently conducted medicinal chemistry campaigns and briefly presents various aspects of drug discovery related to GBM (challenges, therapeutic targets, and small-molecule inhibitors undergoing clinical trials). The scientific literature covered in this Perspective indicates that kinases (phosphoinositide 3-kinases (PI3K),^33−38^ focal adhesion kinase (FAK),^39−41^ DYRK,^42^ and 3-phosphoinositide-dependent kinase 1 (PDK1)^43,44^) have been extensively targeted through the pragmatic design of heterocyclic compounds (triazines, pyrimidines, indoles, oxoindoles, 6:5 fused heterocycles, and others). Notably, these research groups have conducted a series of studies on kinase inhibitors as anti-GBM agents, and their efforts have led to the identification of potent adducts worthy of detailed investigation.^45−53^ Another target that has been reasonably utilized for the construction of anti-GBM agents in the recent past is histone deacetylase (HDAC).^54−61^ The drug design strategies for HDAC inhibitors discussed in this Perspective clearly depict the flexibility of the three-component HDAC inhibitory model. Specifically, the structural alteration of the surface recognition part of the HDAC inhibitory pharmacophore has been the main focus of the medicinal chemist to extract anti-GBM effects through the inhibition of several HDAC isoforms. Several structure–activity relationship (SAR) studies were performed to design inhibitors of isocitrate dehydrogenase (IDH)^62−65^ as well as translocator protein (TSPO).^66−70^ Notably, optimized IDH and TSPO inhibitory scaffolds were exhaustively examined for modification at various sites to attain a clear-cut understanding of the impact of such alterations on the activity. In light of the promising outcomes, it is anticipated that these aforementioned endeavors might emerge as model studies to further numerous future pursuits on IDH and TSPO inhibitors as anti-GBM agents. Protein disulfide isomerase (PDI),^71,72^ tubulin,^73−83^ and hypoxia-inducible factor (HIF)^84,85^ have also garnered significant attention as potential targets and spurred researchers to furnish inhibitors in the pursuit of anti-GBM efficacy. Additionally, the researchers have capitalized on the concept of balanced modulation of two targets as well as the degradation of the targets to outwit the notoriety of GBM cells. Studies discussed in this Perspective on dual MDM-2–TSPO inhibitors,^86^ dual HDAC1–LSD inhibitors,^87^ dual PDK1–aurora kinase inhibitors,^88^ dual RGD integrin–MDM protein inhibitors,^89^ and others^90−92^ are expected to pave the way for the initiation of similar programs to expand the size of the anti-GBM pipeline. Notably, at the preliminary and preclinical levels, medicinal chemists have explored synthetic adducts as well as natural product libraries to furnish new chemical architectures for the treatment of GBM.^93−100^ Imaging tools and chemical probes for GBM (radio-iodinated tracers with specificity to PARP-1,^101^ microtubules,^102^^18^F-labeled radiotracers,^103^ carborane-containing boron dipyrromethenes,^104,105^ and cyanine–gemcitabine^106^) have also been generated. Moreover, many preliminary studies merely focusing on the cellular effects of the new scaffolds have also been included in this work.^107−150^ Although mechanistic studies were not performed, the study results appear to be promising, and the pinpointed potent scaffolds can be subjects of future investigation. Importantly, the literature covered in this Perspective validates the potential of numerous enzymes/proteins/receptors as therapeutic targets in GBM. Several interesting scaffold construction approaches, such as fragment stitching, scaffold installation, regiovariation, bioisosteric replacement, structure simplification, structure rigidification, and molecular hybridization leveraged by the medicinal chemist to design new small-molecule inhibitors with anti-GBM potential, along with SAR, bioactivity, molecular modeling, and other studies conducted to elucidate the mechanisms, are comprehensively discussed in this compilation. These approaches have culminated in generating a voluminous library of CNS-penetrating scaffolds capable of tackling the shield (BBB), and we are quite hopeful that some of the candidates might emerge as potential anti-GBM agents for the clinic.

## 2 Barriers in Anti-glioblastoma Drug Discovery and Delivery

The presence of several barriers, including the BBB, blood–brain–tumor barrier (BBTB), intra-brain tissue diffusion, and drug resistance, has often hindered the drug discovery and delivery process for GBM. Prior to commencement of the task of designing new anti-GBM scaffolds, a thorough understanding of these factors is imperative to amplify the translational rate of preclinical studies to clinical explorations. These physiological barriers restrict the entry of drugs into the brain and make GBM treatment more challenging.

### 2.1 Blood–Brain Barrier (BBB)

The CNS is vascularized with uniquely architectured blood vessels known as the BBB. These blood vessels strictly regulate the movement of ions, molecules, and cells between the blood and the brain. The BBB is designed for proper neuronal function and to protect neural tissue from toxins and pathogens. The BBB is a major obstacle for efficient chemotherapy because it reduces the effective penetration of drugs into the brain and spinal cord due to its highly selective permeability for oxygen and nutrients.^165,166^ Additionally, anatomical features such as the presence of multi-drug-resistant proteins further restrict the entry of drugs into the brain. These anatomical features prevent the accumulation of administered drug molecules inside the brain, resulting in the failure of the administered drugs to achieve the desired pharmacological impact.^167,168^ Additionally, when drugs are transferred through transcellular diffusion, they are metabolized by several metabolic enzymes. For example, decarboxylation of 3-(3,4-dihydroxyphenyl)alanine to dopamine occurs during transit.^169,170^

More than 98% of small drug molecules cannot cross the BBB. The BBB halts more than 95% of drug molecules at the drug development stage. Thus, targeted drug delivery to the brain is not a prime focus area for most pharmaceutical giants.

Previous studies have also revealed that the BBB is a dynamic interface that keeps changing its morphology and physiology under certain pathological conditions. In the presence of such stringent barriers, GBM cells can aggressively infiltrate the surrounding tissues and progress exponentially. Single GBM cells can aggressively develop tumors by infiltration into surrounding tissues and eventually can breach the tight BBB following a multi-step process. GBM cells migrate and accumulate around the existing blood vessels. This causes displacement of the astrocytic end feet processes from vessels. The involvement of TGF-β2, caveolin-1, ROS, and pro-inflammatory peptides in the induction of matrix metalloproteinase (MMP) degradation of tight junctions contributes significantly to the breach of GBM cells through the BBB.^171−173^

Unfortunately, even a disrupted BBB does not allow the permeation of drug molecules to tumor cells because different inhibitory mechanisms, such as drug resistance, poor blood perfusion, and high intra-tumoral interstitial pressure, are still active.^174−176^ Additionally, a disrupted BBB leads to major clinical complications such as vasogenic brain edema and a significant increase in intra-cranial pressure (leaky BBB).^171−173^

### 2.2 Blood–Brain–Tumor Barrier (BBTB)

The progression of GBM from low-grade tumors to high-grade tumors alters the structure, function, and organization of the BBB. This transformation of tumors leads to the invasion of surrounding healthy brain tissue, including BBB disruption, resulting in the formation of neoplastic lesions. These neoplastic lesions have a network of newly built blood vessels that is often referred to as the BBTB. Compared with the BBB, the BBTB is considered more permeable. However, the BBTB is still significantly less permeable than any other tumor neovasculature developed in any other organ of the body. Therefore, the BBTB is also a major challenge for brain drug delivery.^177^

Collectively, GBM is associated with the formation of a highly abnormal lymphatic vasculature and is the most vascularized among human tumors.^178^ Notably, the GBM neovasculature and its heterogeneity determine the permeability of the drug. The GBM neovasculature demonstrates variable vessel diameter and density and can be classified into three different types: (i) continuous, non-fenestrated endothelial vasculature; (ii) continuous, fenestrated endothelial vasculature; and (iii) discontinuous endothelial vasculature.^179^ The neovessels commonly show abnormal endothelial hyperplasia, pinocytic vesicles, fenestration, and opening or loss of tight junctions between endothelial cells. Although the permeability of the BBTB is enhanced by these abnormalities, the cranial microenvironment and specificity of glioma reduce the permeability, thereby hindering the delivery of most anti-tumor agents.^180−182^

### 2.3 Intra-brain Tissue Diffusion of Drugs

Once they pass through the BBB, the drugs reach the cerebrospinal fluid (CSF) and brain extracellular space (ECS). From there, they eventually reach the targeted lesion. The diffusion efficiency of drugs in the ECS is limited by several factors, including the structural and physicochemical properties of the drugs and the physiological properties of the ECS.^180−182^ High infiltration of GBM cells into the brain parenchyma or neighboring brain tissues is another challenge. Because most drugs cover only a few millimeters around the delivery site, a larger area must be targeted to counter the problem of infiltration.^183^ Under such conditions, targeting signaling events and regulatory pathways involved in the migration and invasion of GBM cells appears to be an effective approach.^184^

### 2.4 Chemoresistance and Radiation Resistance of GBM Cancer Stem-like Cells (CSCs)

CSCs are a sub-population of cells within a tumor mass that reproduce tumors and drive malignant progression after treatment. Strong experimental and clinical evidence suggests that CSCs can resist ionizing radiation and chemotherapy.^185−187^ Several cellular factors enable CSCs to possess chemotherapy and radiation resistance, such as an increased DNA damage repair capacity, increased survival signaling, and upregulated ROS scavengers.^188−191^ Notably, TMZ resistance, a troubling issue, is primarily driven by GSCs. Revelations in this context indicate that enriched populations of stem-like CD133^+^ cells mediated via upregulation in DNA repair mechanisms are produced by radiation and chemotherapy regimens. Recently, studies have identified reliable GSC markers, including CD133, CD44, CD15, CD70, S100A4, ALDH1A3, Nanog, SOX-2, and Nestin. Outcomes of fate mapping studies using genetic barcoding have indicated that chemotherapy leads to evolutionary selective pressure that causes the expansion of drug-resistant GSCs. Although GSCs comprise a very low percentage of cells in GBM tumors, their ability to regenerate tumor heterogeneity makes them a potential target for emerging anti-neoplastic therapeutic approaches.^192−195^

### 2.5 Factors Affecting Brain Drug Delivery

The potential of drug molecules to cross the BBB and treat GBM is affected by several factors, including the physicochemical properties of the drug molecule, its pharmacokinetic (PK) profile, characteristics of the drug delivery system (DDS), and the pathophysiological condition of the patient. Most of the drug molecules used to treat GBM are non-specific agents that target actively dividing cells. Thus, these therapeutic agents not only kill cancerous cells but also destroy actively dividing healthy cells, and physiological aberrations, including immunological suppression, mental depression, and neurological degeneration, have been reported.^196,197^ Treatment strategies should be designed to overcome the cell cycle dependence and lack of specificity of chemotherapeutic agents.^178^

Notably, the physiochemical properties of the pharmaceutical agent, such as the size, flexibility, chemical conformation, ionization, and lipophilicity of the drug molecule, play critical roles in determining the ability of the drugs to reach the targeted site in the brain. Generally, drugs that are moderately lipophilic tend to cross the BBB through passive diffusion, while polar molecules act as better drug molecules if taken through active transport across the CNS. Key disclosures indicate that CNS drugs (basic) exist in an equilibrium between their charged and neutral states under physiological conditions or are amphiphilic if they also possess an acidic group. Additionally, brain permeation is favored by possessing a positive charge at pH 7–8.^198,199^ It has been reported that tertiary nitrogen-bearing compounds (structural attributes of numerous CNS drugs) exhibit a higher degree of brain permeation.^200^ As such, the partitioning of the drugs into membrane lipids occurs as neutral species and depends on the concentration of the neutral species and its lipophilic properties. Acids and bases that are too strong are usually precluded from BBB penetration, such as carboxylic acids, which demonstrate difficulty in penetrating the CNS.^201^ Thus, the p*K*_a_ limits for BBB penetration defined by Fischer et al.^202^ are between 4 and 10.

A relative comparison of CNS with non-CNS drugs indicates that drugs belonging to the former category are smaller and more lipophilic and have fewer hydrogen-bond donors and lower polar surface area (PSA). The profile of a desirable CNS candidate depicts the following values: cLogP = 2.8, cLogD = 1.7, HBD = 1, TPSA = 44.8 Å^2^, p*K*_a_ = 8.4, RB = 4.5, and MW = 305.3 Da (the median values are derived from an analysis of marketed CNS drugs).^203,204^

In addition to the above-mentioned, an appropriate PK profile (absorption, distribution, metabolism, and excretion), which plays a key role in defining the disposition of a drug candidate and ultimately its development as a suitable marketable drug candidate, is equally important.^153^ Notably, the lack of an appropriate PK profile of both developmental and marketed drugs leads to failure in advanced development stages and market withdrawal.^205^ Additionally, substantial potency plus selectivity combined with the ability to achieve target tissue concentrations above a certain threshold value is desired to achieve the optimum therapeutic efficacy of a drug candidate. To attain the above-mentioned features, structural optimization of the chemical architectures has become an imperative task of drug discovery campaigns, and the implementation of logical strategies by the medicinal chemist can favorably modulate the PK properties of an agent.

Similarly, the selection of a suitable dosage form is also equally critical. As mentioned previously, the physicochemical properties, such as the particle size, zeta potential, lipophilicity, permeability, and dissolution rate, of the drug delivery tool directly influence the potential of drug molecules to cross the BBB. Interestingly, these characteristics can be customized by selecting a suitable carrier system and a suitable composition using suitable formulation methodology, modification of the surface chemistry, and grafting of the surface with specific ligands. In the past few years, extensive work has been conducted in this area, and various approaches have been explored to improve the specific biodistribution, surface characteristics, and targeting of anti-cancer drugs.^179^ The potential of other therapeutic approaches, such as gene targeting^184^ and the use of aptamers as delivery agents,^182^ has also been explored. Despite the significant progress in this field, the present scenario necessitates the introduction of potentially effective DDSs that specifically target GBM cells without affecting healthy cells.

Additionally, existing pathological conditions and drug affinities for efflux mechanisms influence the pharmacological outcome of the drug.^206,207^ Factors such as systemic enzymatic stability, mode of absorption, clearance rate, and site of administration are also of considerable importance.

## 3 Therapeutic Targets and Small-Molecule Inhibitors Undergoing Clinical Investigations

Significant explorations have been conducted to identify immunotherapeutic and chemotherapeutic targets to treat GBM. This section presents a brief overview of potential targets for GBM (Table 2) along with an update on small-molecule inhibitors undergoing explorations in various phases of clinical trials (Table 3).

**Table 2:** Therapeutic Targets for GBM

**Cytokines and Cytokine Receptors**	
cytokines	• Immunotherapy augments the immune response to get rid of neoplastic cells. This includes various categories, such as adoptive cell therapy, monoclonal antibodies, checkpoint molecules, and vaccination.^208−212^
	• The immune system has key signaling molecules, i.e., cytokines, which at both signaling and receptor levels have proved to be potential biomarkers in GBM. They are observed to be overexpressed in GBM cells as compared to normal brain tissue and are being considered as potential therapeutic targets for GBM.
	• Tumor growth in patients with GBM is attributed to differential regulation of pro-inflammatory and anti-inflammatory cytokines causing a shift in immune landscape.
	• Cytokines are delivered locally, which makes it difficult to determine, and this is considered a shortcoming for cytokines to be used as a GBM biomarker. The other limitation of using cytokines as a biomarker for GBM is decreased sensitivity owing to the difficult identification of the window when there is a change in cytokine release.^213,214^

interleukin-4 (IL-4) receptors	• Other cytokines involved in several immunologic processes are anti-inflammatory cytokines, IL-4 receptors. IL-4 is an admissible biomarker and therapeutic target, as it is observed to be overexpressed in GBM.
	• IL-4 and pseudomonas endotoxin are used to create IL-4 toxin that is cytotoxic to GBM cells. To add on, IL-4R is considered to be a potential biomarker for GBM cells, which can be used as a base to develop targeted therapies.^213,215^

interleukin-13 (IL-13) receptor	• Structurally similar to IL-4 receptors, there is another anti-inflammatory cytokine, IL-13 receptor, which is manifested in higher levels in human glioma cells as compared to healthy cells, rendering it a potential biomarker and tumor-specific antigen.^213,216^

**Immune Checkpoints**	
PD-I	• Immune checkpoints keep balance of the immune system by participating in prevention or promotion of the development of many autoimmune diseases. Immune checkpoint molecules such as co-inhibitory and co-stimulatory molecules are recruited to modulate T cell responses.^213,217−219^
	• PD-I, also known as CD279, is a co-inhibitory checkpoint molecule which binds to its ligands (PD-L1 and PD-L2, respectively) to suppress the immune response. PD-I signaling helps in generating an anti-inflammatory response by decreasing the production of cytokines eventually to prevent autoimmune attacks.
	• PD-L1 has been observed to be an overexpressed biomarker in GBM tissue as compared to normal brain tissue. Impeding the T cell activation of CD-4 and CD-8 and enabling gliomas to escape immune-mediated attacks, PD-I expression plays an important role in diagnosis and clinical response in patients; however, it cannot be completely categorized as positive or negative signaling, which makes it incompetent to be used alone as a helpful biomarker for GBM.^213,220^

CTLA-4	• CTLA-4 (CD152) is a negative checkpoint regulator which has been largely investigated in cancer immunotherapy. Recent findings indicate that CTLA-4 correlates with immune and clinical characteristics of glioma.^213,221^

**Immune Modulators and Regulators**	
TIM-3	• TIM-3 is a surface protein which modulates immune suppression and induces T cell apoptosis. TIM-3 is also an important target, as it is overexpressed in various cancers, showing events of T cell exhaustion, allowing cancer to escape immune-mediated cell death.
	• The Karnofsky Performance Status score indicates that elevated TIM-3 expression is related with higher grades of glioma and poor functions, clinically. Therefore, future clinical trials are required to verify its role.^213,222−225^

immune regulators	• Several positive immune regulators have been proved to improve survival in animal models of GBM. CD137 (4-1BB), when used along with anti-CTLA-4 antibody and radiation therapy, assists T cell proliferation and escalates survival in GBM murine models, likely by increasing immune activity against tumors.
	• Another stimulatory checkpoint molecule is the glucocorticoid-induced TNFR-related gene, which acts by increasing Treg cell proliferation. OX4OL is another stimulatory checkpoint molecule which is associated with prolonged survival in murine models for GBM. Hence, exploring more about stimulatory checkpoint molecules as potential biomarkers can be another helpful strategy for GBM treatment.^213,226^

**Receptor Tyrosine Kinase (RTK)**	
epidermal growth factor receptor (EGFR)	• A transmembrane glycoprotein, EGFR is a member of the tyrosine kinase superfamily of receptors. Literature precedents indicate that many EGFR gene alterations were found to be involved in GBM, including amplifications, deletions, and single nucleotide polymorphisms (SNPs). Detected in 40–60% of GBM cases, EGFR amplifications are generally indicative of poor prognosis. In light of the aforementioned, monoclonal antibodies directed against wild-type EGFR and EGFR along with numerous small-molecule tyrosine kinase inhibitors have been extensively studied in GBM.^227^

met proto-oncogene (MET)	• MET, a RTK required for embryonic development and tissue repair, is found to be dysregulated in GBM. The mechanisms involved in this dysregulation includes somatic mutations, rearrangement, amplification, and overexpression of MET and hepatocyte growth factor (HGF, ligand for MET) that leads to autocrine loop formation.
	• In addition, an inverse correlation has also been evidenced between MET expression and patient survival, suggesting that MET is upregulated in GBM.^228^

PI3K/Akt/mTOR (PAM) pathway	• The PAM pathway has been shown to be activated in 90% of all GBM.^229,230^ The outcomes of some studies revealed that the PI3K signaling cascade regulates the motility of differentiated GBM cells and has only a marginal effect on their survival when subjected to combination treatment with a chemotherapeutic agent.^231^ In light of these findings, pan-PI3K inhibitors, isoform-selective and dual PI3K/mammalian target of rapamycin (mTOR) inhibitors, were exhaustively explored in the recent past in GBM, and optimistic results were attained.

vascular endothelial growth factor receptor (VEGFR)	• VEGFR is considered to be the most abundant and important mediator of angiogenesis in GBM, and its upregulated expression is directly associated with the poor prognosis and malignancy of gliomas.
	• Multiple strategies have been established to address VEGF/VEGFR-mediated angiogenesis, such as VEGFR signaling suppression, VEGF blockade, and VEGF trap. The optimistic results evidenced in the majority of the cases exercising the aforementioned strategies support VEGFR inhibition as a candidate for a specific and less toxic therapeutic strategy than cytotoxic therapy.^227,232^

**Serine/Threonine-Specific Protein Kinase (STK)**	
protein kinase C (PKC)	• PKC is a serine/threonine kinase that is highly expressed in GBM, resulting in the proliferation, survival, invasion, and migration of GBM cells.^233^ The isoforms of PKC are involved in the chemoresistance through various pathways, the contributions of which depend on phosphorylation of tyrosine residues.^234^ This understanding of PKC makes it an promising target against GBM.^235^

transforming growth factor beta (TGF-β)	• Literature precedents reveal that TGF-β is solely present in GBM tissues and is seen in higher levels in tumor-bearing animals, causing immune suppression, which promotes cancer growth. Therefore, poor prognosis in patients with GBM are associated with enhanced levels of TGF-β. Although more studies are needed to prove its sensitivity and specificity, still TGF-β can be considered a useful biomarker for GBM. TGF-β has three groups: TGF-β mRNA translational inhibitors, TGF-β neutralizing antibodies, and TGF-β receptor modulators. Overall, TGF-β is considered to be a potential immunotherapeutic target.^213,236−240^

endoglin	• Endoglin (CD105) is a structural part of the TGF-β receptor that causes the new vessels to form and endothelial cells to proliferate. Several studies have revealed that CD105 can emerge as a potent prognostic indicator and biomarker for monoclonal antibody treatment in GBM patients.^213,241^

other STKs	• Raf proto-oncogene (RAF),^242^ mitogen-activated protein kinase (MAPK),^243^ p38 MAP kinase/mitogen-activated protein kinase 14 (p38MAPK),^244^ mechanistic target for rapamycin kinase 1 (mTORC),^245^ cyclin-dependent kinase 4/6 (CDK 4/6),^246^ Wee1 G2 checkpoint kinase (Wee1),^247^ protein kinase C beta (PRKCB),^235^ and DNA-dependent protein kinase (DNA-PK)^248^ represent the prominent targets for GBM belonging to this category.

**Focal Adhesion Kinase (FAK)**	• Several studies have established the relationships between FAK and proliferation, survival, and migration, as well as angiogenesis and glioma malignancy grade. Moreover, revelations in the context of stimulation of CSC renewal by FAK make it a prudent therapeutic target for GBM.^249−251^

**Other Kinases**	• Platelet-derived growth factor receptor-alpha (PDGFRA),^252^ human epithelial growth factor receptor 2 (HER/ERBB2),^253^ human epithelial growth factor receptor 3 (HER/ERBB3),^254^ Met proto-oncogene/hepatocyte growth factor receptor (MET/HGFR),^255^ fibroblast growth factor receptor (FGFR),^256^ Kit proto-oncogene (KIT),^256^ insulin-like growth factor 1 receptor (IGF1R),^257^ colony-stimulating factor 1 receptor (CSF1R),^258^ anaplastic lymphoma kinase (ALK),^259^ Ros proto-oncogene 1 (ROS1),^260^ Ret proto-oncogene (RET),^261^ Bruton tyrosine kinase (BTK),^262^ Eph receptor A3 (EPHA3),^263^ neurotropic tyrosine receptor kinase 1 (NTRK1),^264^ Axl receptor kinase (AXL),^265^ Mer proto-oncogene tyrosine kinase (MER),^266^ Abelson murine leukemia viral oncogene homolog 1 (ABL),^267^ Src proto-oncogene (SRC1),^268^ Janus kinase 1 (JAK1),^269^ mitogen-activated and stress-activated protein kinase 1,^270^ dual specificity kinase DYRK3,^271^ CDC-like kinases (CLK),^272^ and SRC kinase^273^ represent the prominent targets for GBM belonging to this category.

**Epigenetic Targets**	
histone deacetylase (HDAC)	• Alterations in sequence and/or expression of gene coding for HDACs have been reported to be implicated in GBM pathogenesis and progression.^2,274^ In attempts to capitalize on these revelations, explorations were conducted to evaluate the efficacy of FDA-approved HDAC inhibitors against GBM.^275^
	• To add on, stemness properties in GSCs were diminished on treatment with SAHA, indicating that HDACs plays a role in preserving stemness characteristics in GBM.^2^ In particular, the strategy of selectively inhibiting the HDAC6 isoform appears to be quite promising owing to the elevated levels of HDAC6 in GBM and GSCs.^61,276,277^

poly(ADP-ribose) polymerase (PARP)	• Studies indicate that PARP targeting can sensitize GBM cells to ionizing radiation and chemotherapy.
	• Olaparib (PARP inhibitor) demonstrated an ability to potentiate radiation and TMZ chemotherapy in preclinical studies and is currently undergoing clinical stage investigation.^278^

topoisomerase	• Topoisomerase as a therapeutic target has been leveraged for the treatment of high-grade gliomas, such as GBM. Several clinical trials are ongoing in pursuit of evaluating the cocktail of topoisomerase inhibitors with other chemotherapeutic drugs in GBM.
	• A recent investigation revealed the mediation of GSCs to replication stress-inducing drugs, indicating that Top2β might emerge as a new target for gene therapy in GBM.^279^

enhancer of zeste homolog 2 (EZH2)	• EZH2, a crux subunit of the PRC2, is a HMT enzyme responsible for methylating lysine 27 (mono-, di-, and trimethylation) in histone H3 (H3K27) and is involved in regulation of cell stemness and epithelial-to-mesenchymal transition (EMT) in gliomas.
	• It has been found to be responsible for multi-drug resistance development, and there is evidence that EZH2 inhibition restores normal drug sensitivity in GBM.^280^
	• EZH2 has also been identified as a promising target for H3K27M mutant pediatric gliomas.^281^

EphA receptors	• EphA2 is involved in the proliferation of GBM, and EphA2 agonists showed potential growth inhibition of GBM cells.^282^
	• Overexpression of EphA3 is reported on the tumor-initiating cell population in glioma.
	• EPhA3 is involved in the maintenance of tumor cells in a less differentiated and stem-cell-like state in glioma.^283,284^

bromodomains	• It is well known that BET bromodomain proteins recognize lysine-acetylated histones and regulate gene expression. Some studies have reported elevated levels of bromodomain proteins BRD2 and BRD4 in GBM. In light of the aforementioned, BET protein inhibition is being considered as a prudent strategy to emerge as a potential therapeutic approach for GBM patients that experience TMZ-resistant tumors.^285^ Both small-molecule inhibitors and degraders of the BET proteins have garnered the attention of researchers in the recent past.

lysine-specific demethylase 1 (LSD1)	• LSD1 represents another epigenetic target that has been found to exert favorable trends via a chemical strategy affording its inhibition. As such, LSD1 is a histone modifier that actively participates in the process of gene transcription along with the regulation of methylation dynamics of non-histone proteins. A recent study reported induction of senescence in GBM via LSD1 inhibition through a HIF-1α-dependent pathway.^286^
	• It has also been reported that sensitization of GBM cells to HDAC inhibitors can be attained through LSD1 inhibition, and this disclosure further presents the cooperation between LSD1 and HDACs for the regulation of cell death pathways in GBM cell lines.^287^
	• In a nutshell, LSD1 inhibition along with simultaneous dual inhibition of LSD1 and HDAC is presently being conceived as a potential strategy for the treatment of GBM.

isocitrate dehydrogenase	• Mutations in IDH1 and IDH2 have been evidenced in over 80% of low-grade gliomas (LGGs) and secondary GBM.^288^
	• Moreover, it is also assumed that IDH1/2 mutations lead to the initiation of oncogenic events that cause epigenetic remodeling in neural progenitor cells. This exerts inhibition of normal cellular differentiation processes that ultimately promotes gliomagenesis.^289^
	• In this context, the inhibition of IDH is being evaluated as an effective approach for the development of therapeutics for GBM.

**Pathways**	
JAK/STAT	• JAK/STAT signaling has been identified as an important driver of gliomagenesis and treatment resistance. In this context, the combination of JAK and STAT inhibitors needs to be evaluated to ascertain conclusive benefits.^290^

nuclear factor kappa B (NF-κB) signaling pathway	• Reports regarding the participation of NF-κB in apoptosis, cellular proliferation, angiogenesis, metastasis, invasion, and many other processes implicated in GBM pathobiology ascertain the candidature of NF-κB regulation as an imperative pharmacological target for the treatment of GBM therapy.
	• Owing to the aforementioned, several phytoconstituents were evaluated and were found to have NF-κB modulatory effects against GBM along with cancer cell selectivity.^291^

**Other Targets**	
G protein-coupled receptors (GPCRs)	• Studies centered at the investigation of GPCR expression in GSCs revealed the exclusive expression of several GPCRs, such as LPHN2, GPR37, CALCRL, HRH2, GPR73, S1PR_5_, GPR128, and GPR103, thereby presenting the candidature of GPCRs as molecular modulators to control the stem cell phenotype.^292^
	• Smoothened frizzled class receptor (SMO),^293^ C-X-C motif chemokine receptor 4 (CXCR4),^294^ dopamine receptor D2 (DRD2),^295^ and dopamine receptor D3 (DRD3)^296^ represents the prominent targets for GBM belonging to this category.

cell surface receptor	• Integrin-mediated signaling pathways cause modification of the brain microenvironment and support tumoral niche formation that promotes the invasiveness and survival of glioma cells. In particular, RGD-binding integrins play an important role in the epithelial–mesenchymal transition process.^297^
	• In view of this, design, synthesis, and evaluation of antagonists of integrin are presently being attempted as a part of some structural engineering programs.
	• Lymphocyte activating 3 (LAG3),^298^ Fas cell surface death receptor (CD95),^299^ and Adam metallopeptidase domain 10/17 (ADAM 10/17)^300^ represents the prominent targets for GBM belonging to this category.

signal transducer and activator of transcription 3 (STAT-3)	• The association of STAT3 has been identified as a critical initiator and regulator of tumorigenic transformation in GBM. Moreover, it is also involved in GSC maintenance.^301^

translocator protein (TSPO)	• TSPO, at present, is being explored as a marker in positron emission tomography (PET) for the visualization of brain lesions. To add on, the results of some studies reveal the elevated levels of TSPO expression and indicate the involvement of TSPO in tumorigenesis and glioma progression.^302^
	• Overall, TSPO targeting is presently being conceived as a mechanism to negate the apoptotic-resistant, invasive, and aggressive nature of GBM.^303^

murine double minute-2 (MDM2)	• Impaired functioning of p53 tumor suppressor through either genetic mutation or sequestration by other protein leads to development of cancer and chemoresistance. p53 availability is generally reduced in GBM due to binding to MDM2 oncoprotein that gets accumulated in the tumor cells at high concentrations. These revelations certainly present the inhibition of MDM2 as a logical strategy to design therapeutics for GBM.^297,304^

Rap1a GTPase	• Rap1 belongs to the Ras family of small GTPases and is involved in the regulation of migration of both normal cells and cancer cells.
	• A recent study demonstrated an increase in U-87MG glioma spheroid invasion on collagen in response to PDGF stimulation. Furthermore, it was also found that the chronic elevation of Rap1a expression in GBM tumors leads to disease progression.
	• Collectively, Rap1a is presently given due consideration for exhaustive exploration to confirm its role in cellular proliferation (GBM tumor growth).^305^

microtubules	• Alteration of microtubules dynamics evidenced in cancer cells is linked to chromosomal instability, aneuploidy, and development of drug resistance.
	• Numerous studies have ascertained the sensitivity of glioma to microtubule-targeting agents, and microtubules represent a validated target for the design of tubulin inhibitors at the preclinical level.
	• Future attempts need to be directed toward the development of CNS-penetrating microtubule-targeting agents that can enhance the therapeutic value of such agents in neuro-oncology.^306^

others	• Heparanase type 4,^307^ aldehyde dehydrogenase,^308^ adenosine A3 receptor,^309^ pyruvate kinase,^310^ human thymidine phosphorylase,^311^ glucose transporter type 4,^312^ nicotinic acetylcholine receptors,^313^ heat shock protein (HSP) 27,^314^ AMPA receptor,^315^ angiopoietin 1/2,^316^ placental growth factor,^317^ Ras proto-oncogene,^318^ GTPase,^319^ indoleamine 2,3-dioxygenase (IDO),^320^ farnesyltransferase,^321^ exportin 1,^322^ Wilms tumor 1,^323^ proteasome,^324^ and Wnt^325^ are other targets that expand the list for the medicinal chemist to develop new anti-GBM agents.

**Table 3:**
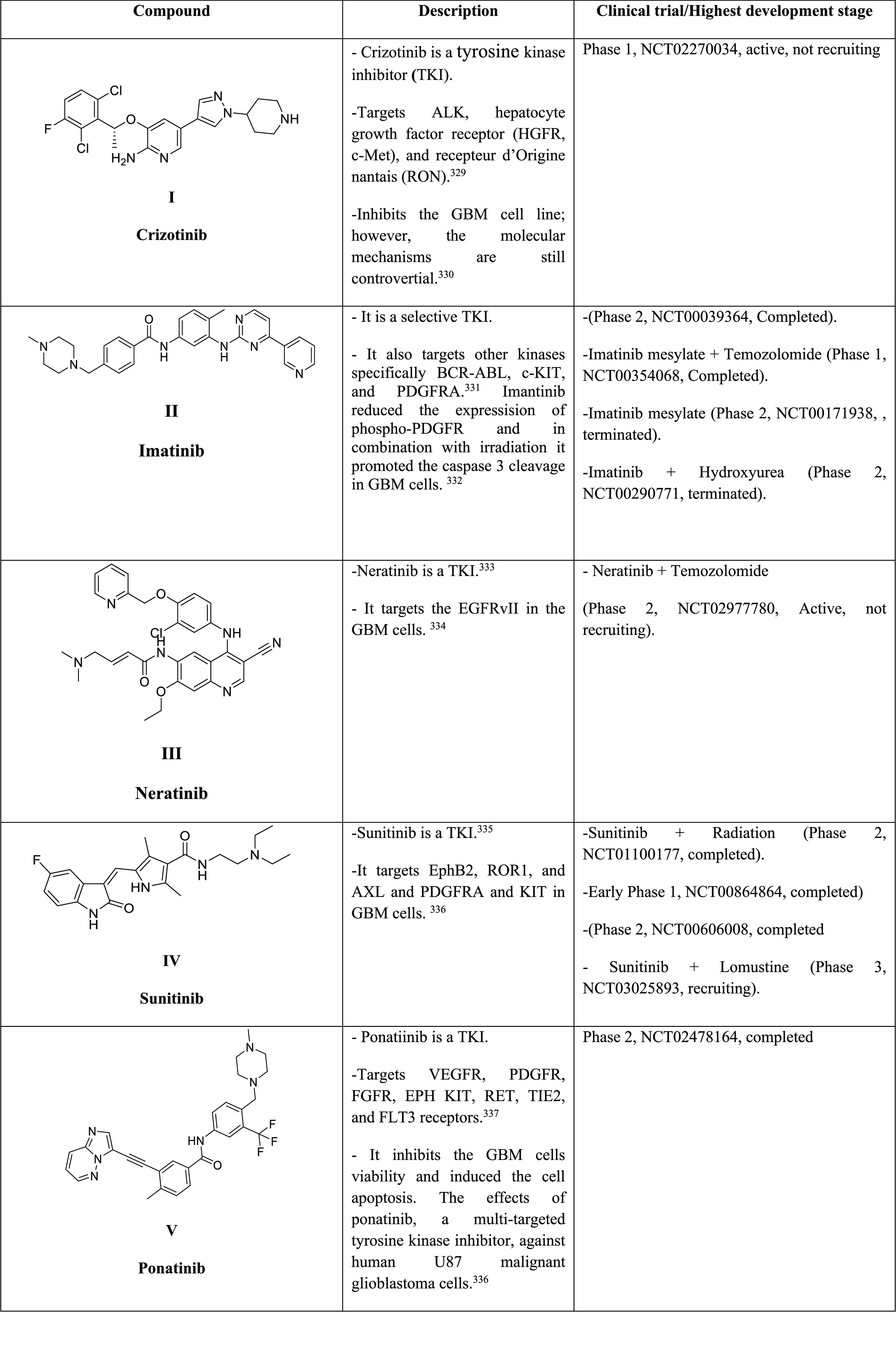

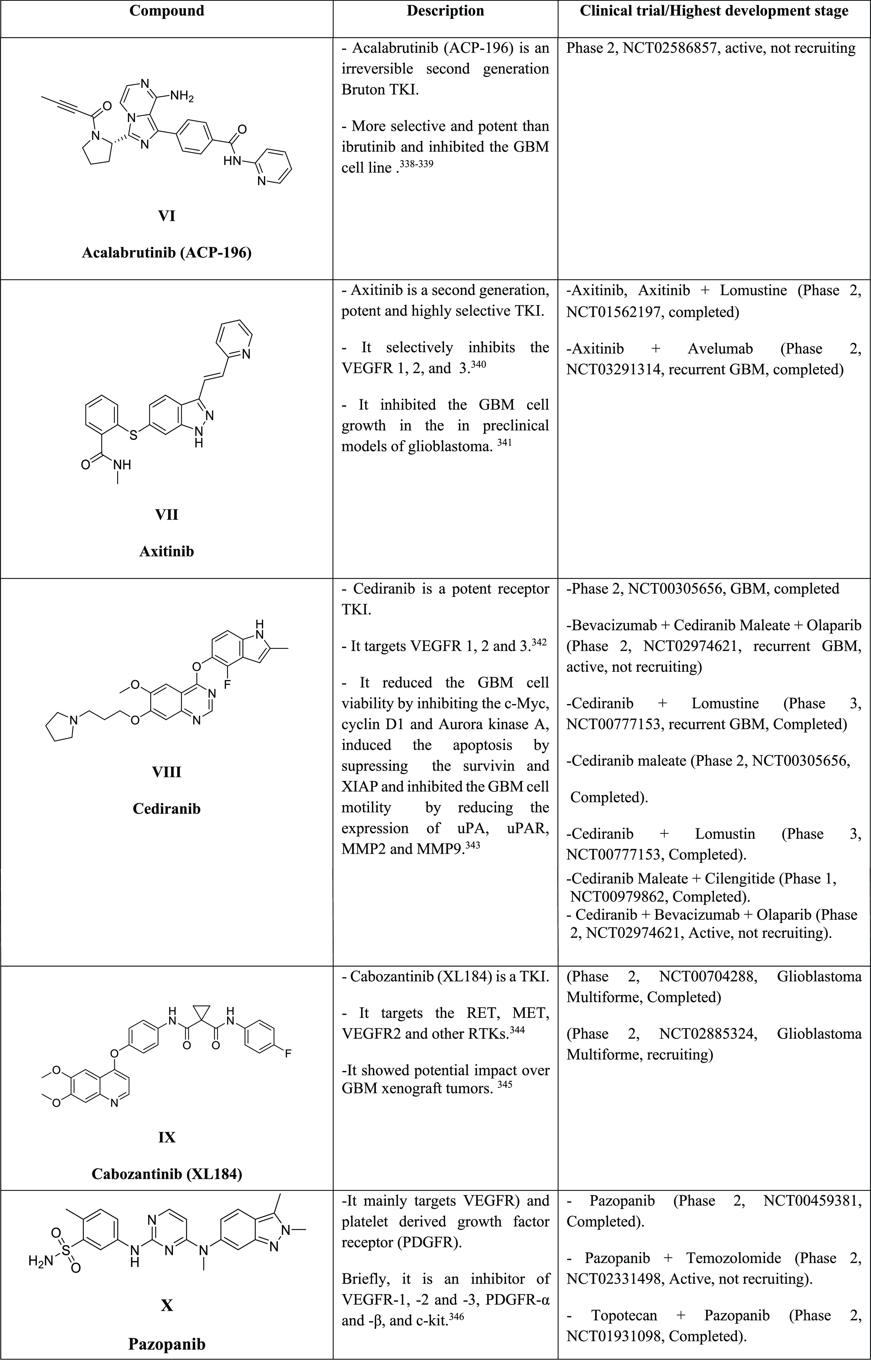

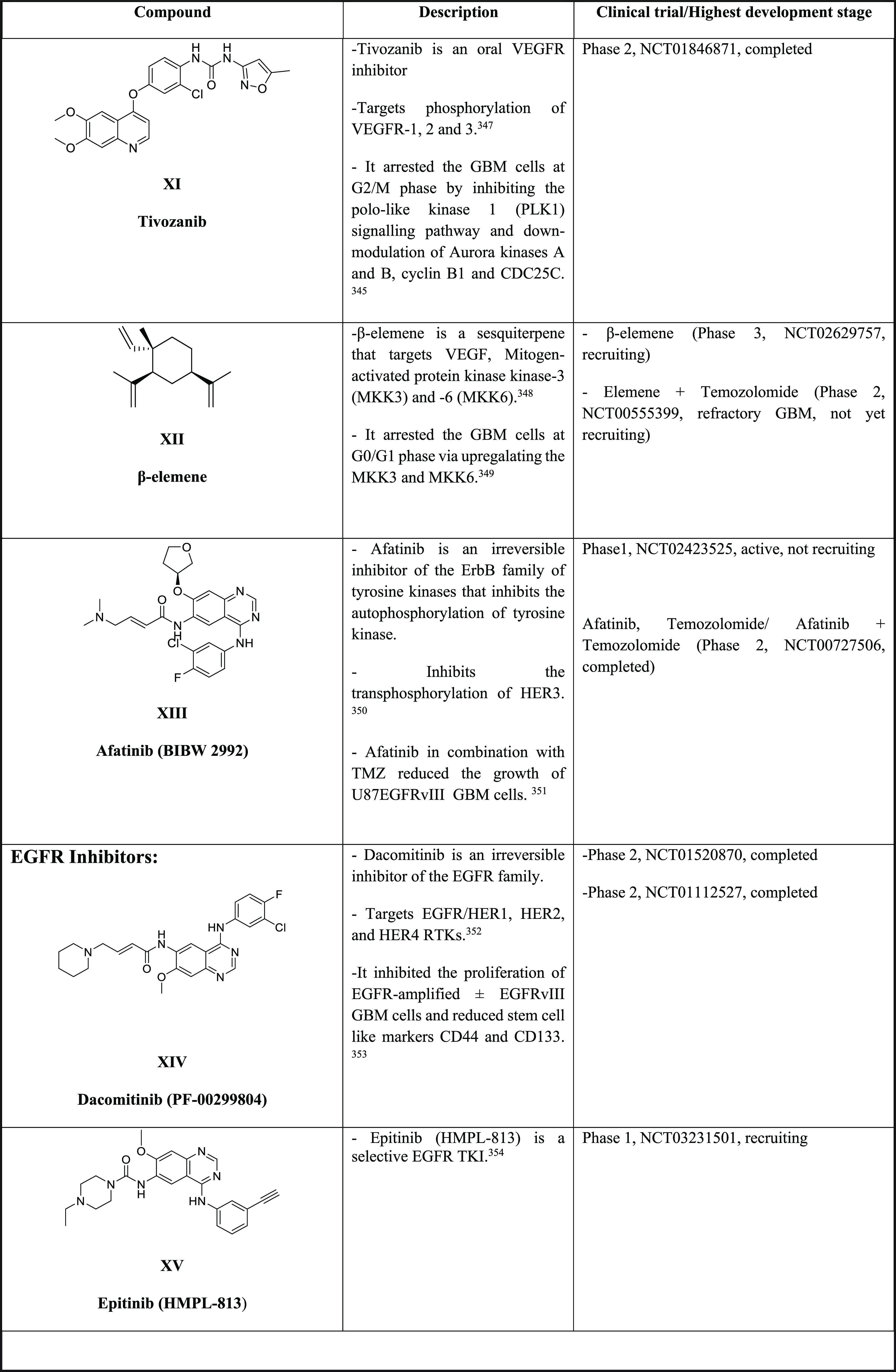

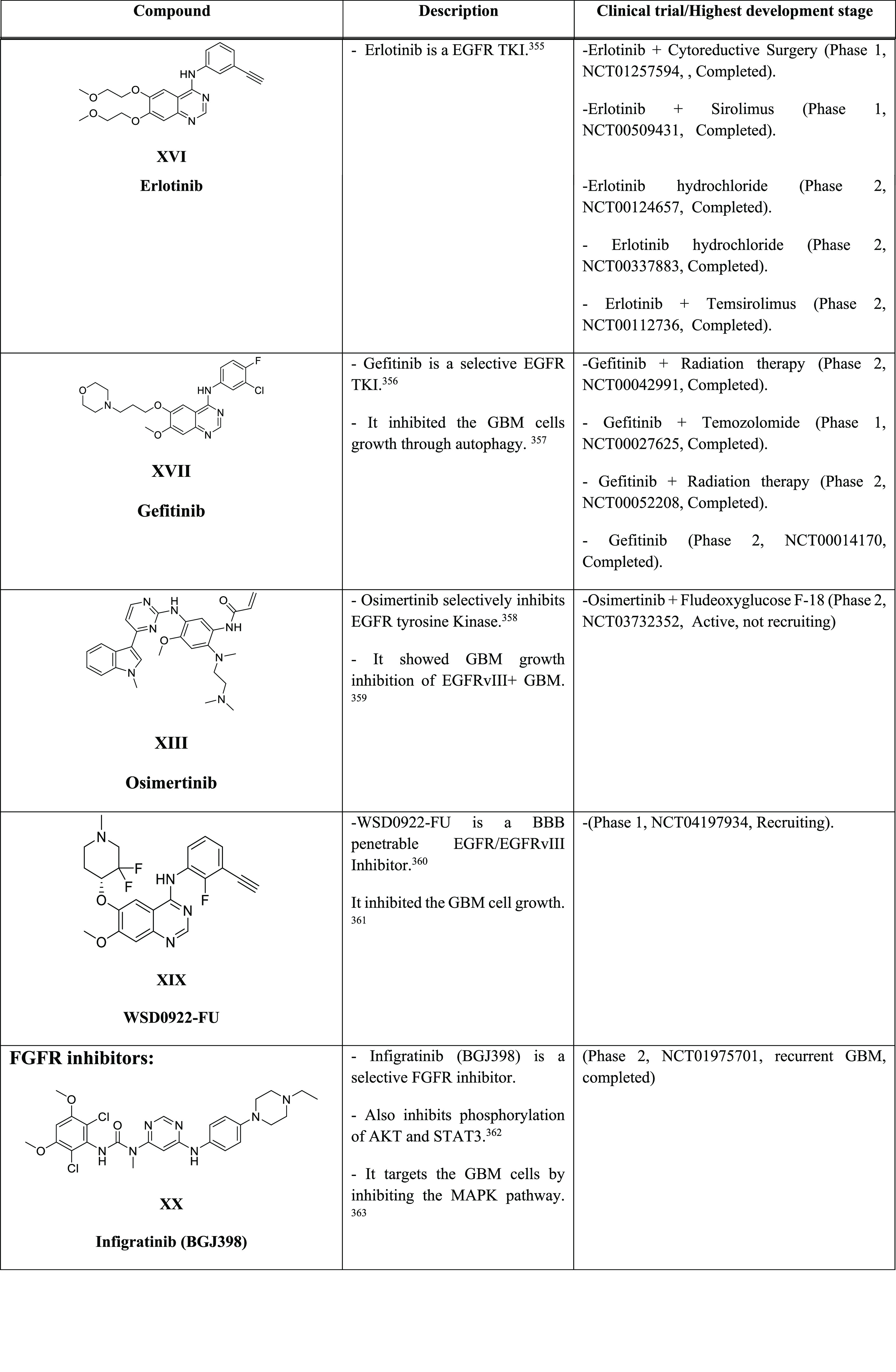

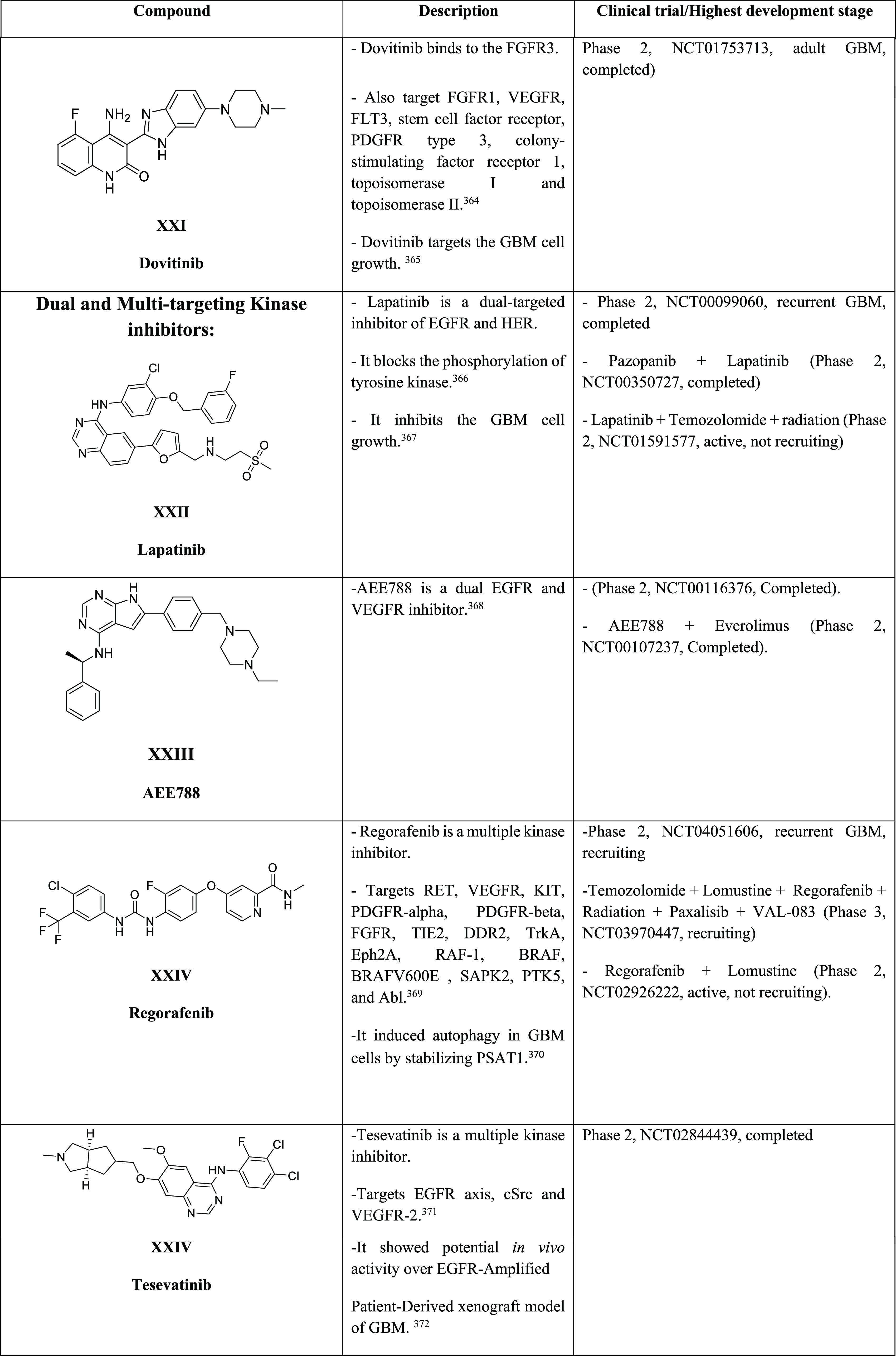

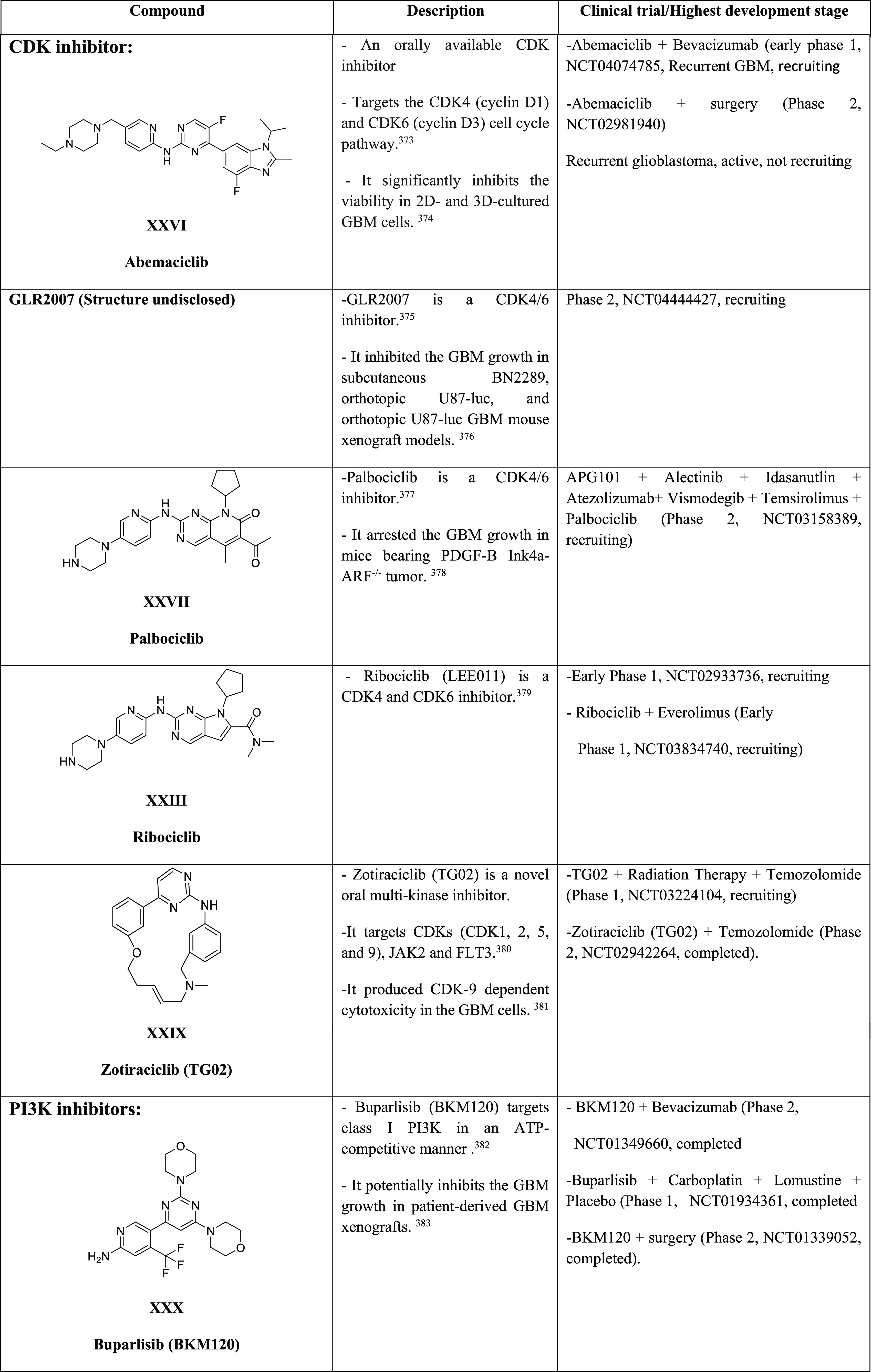

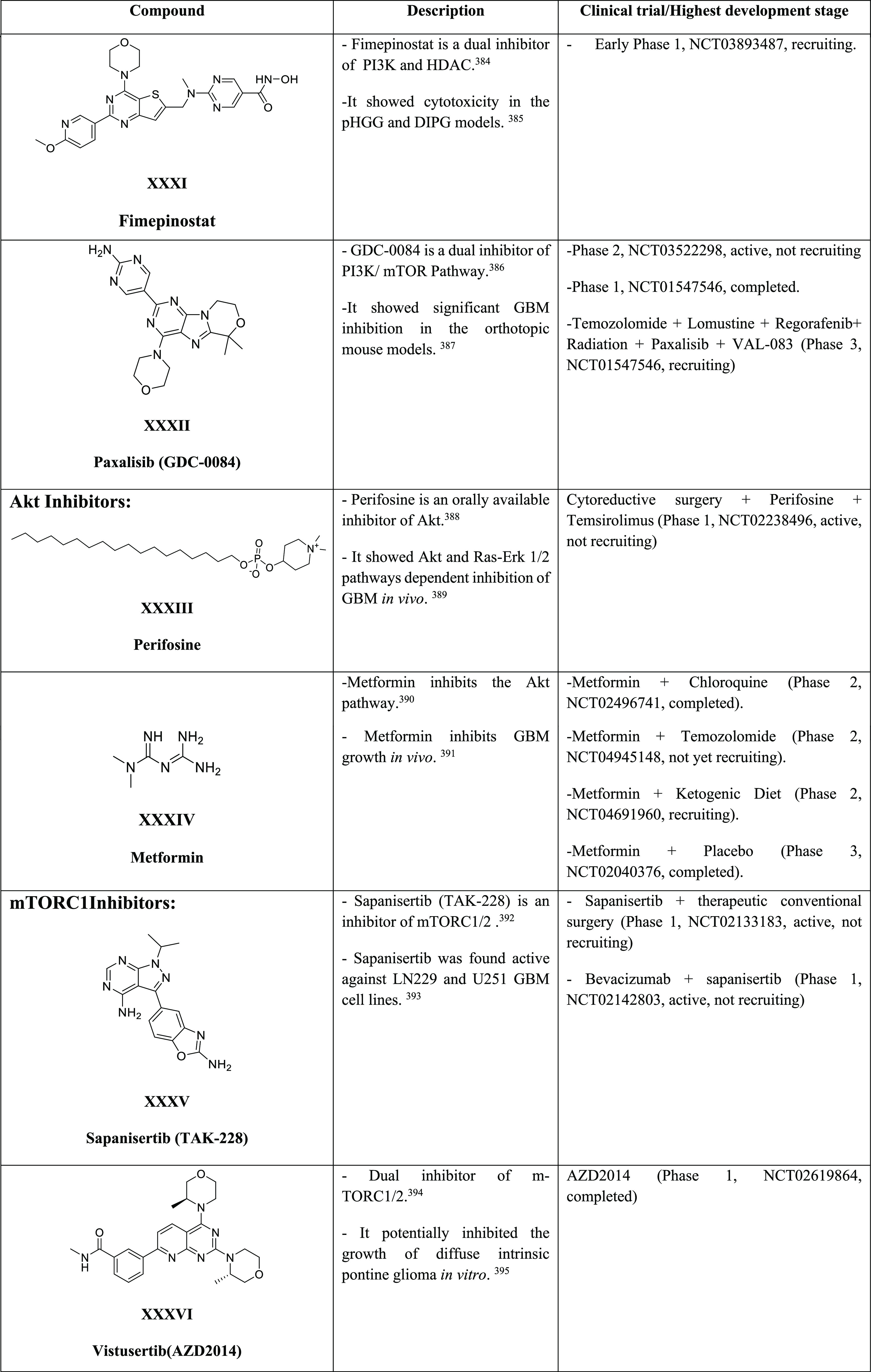

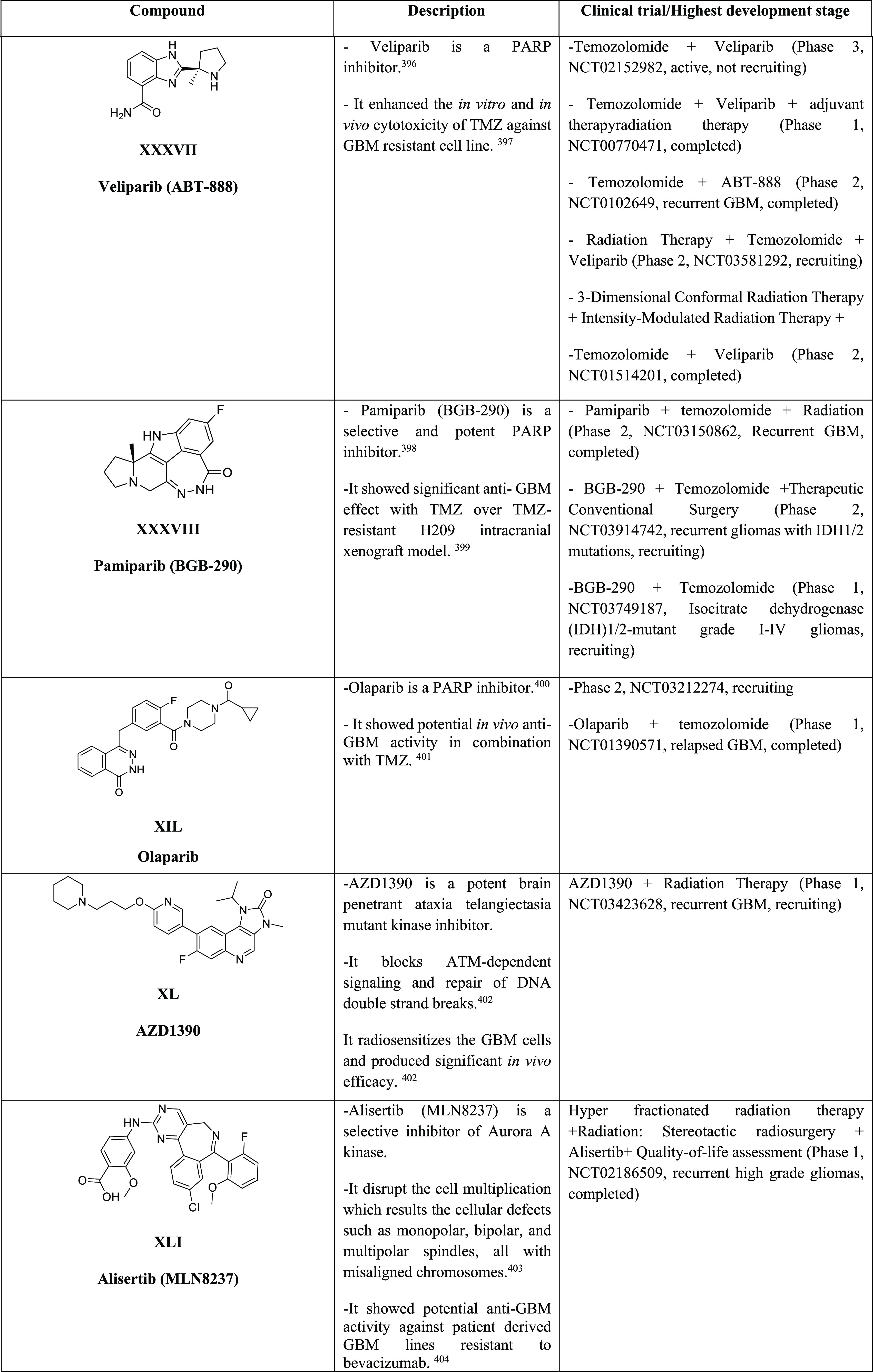

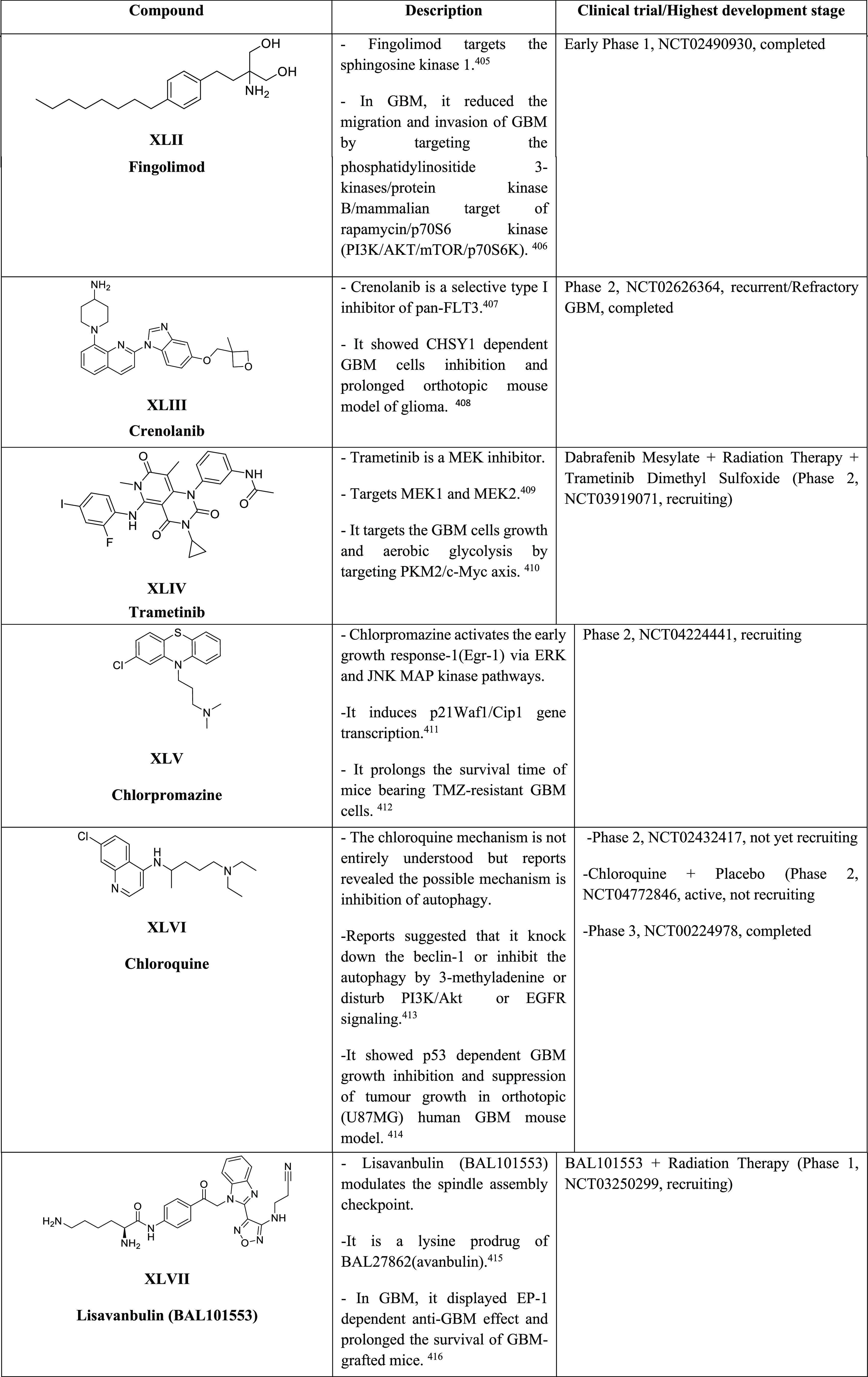

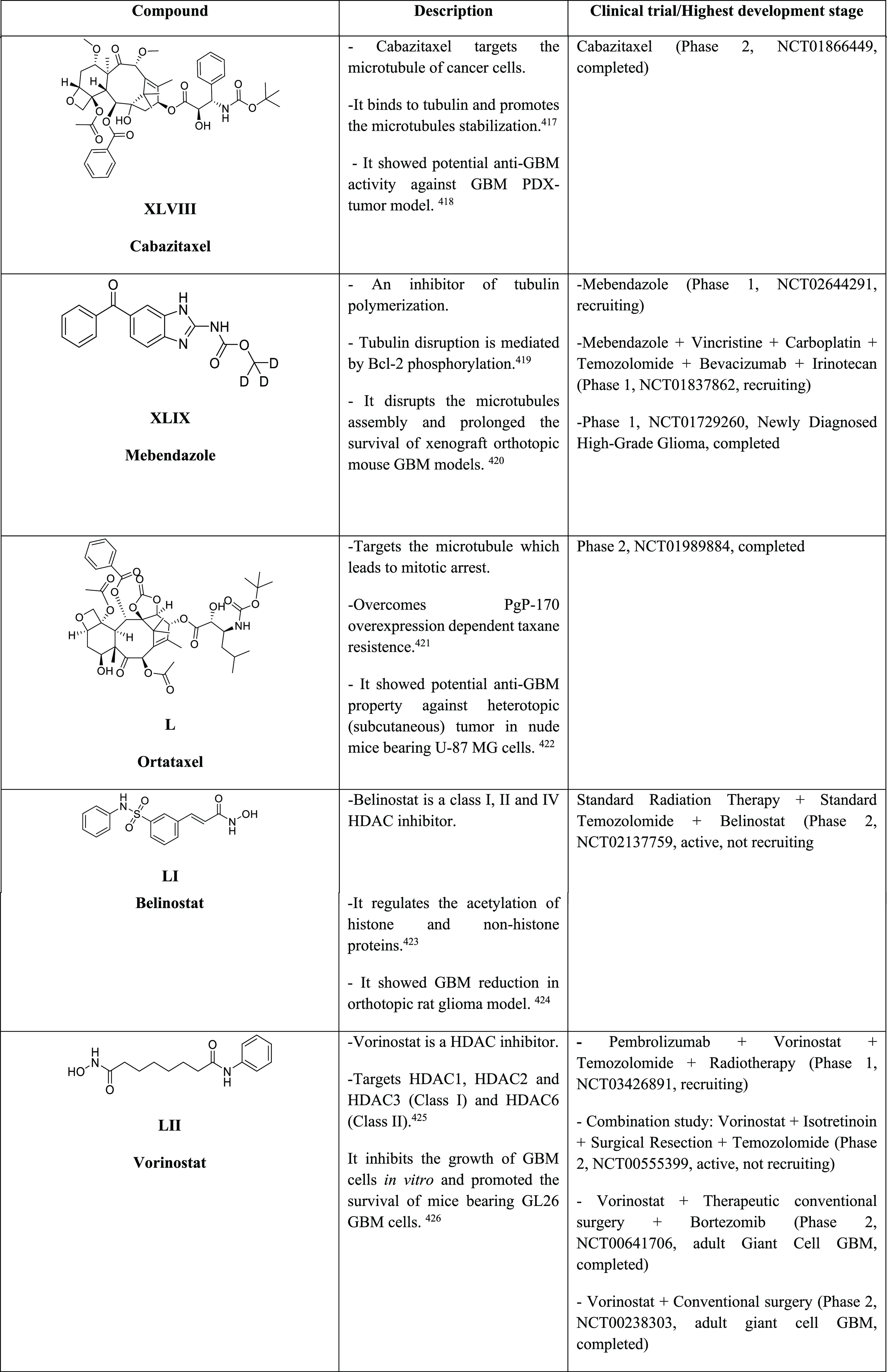

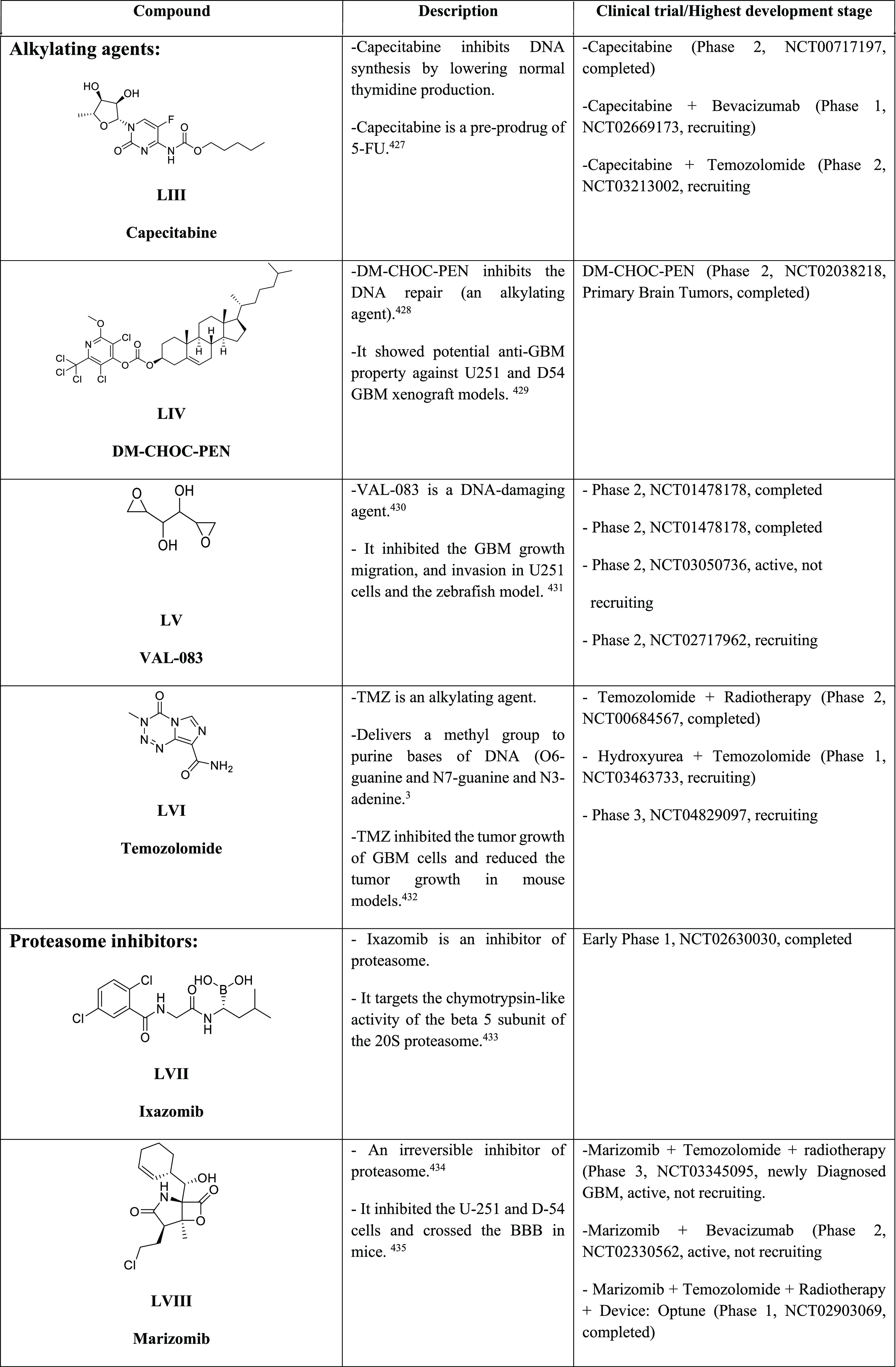

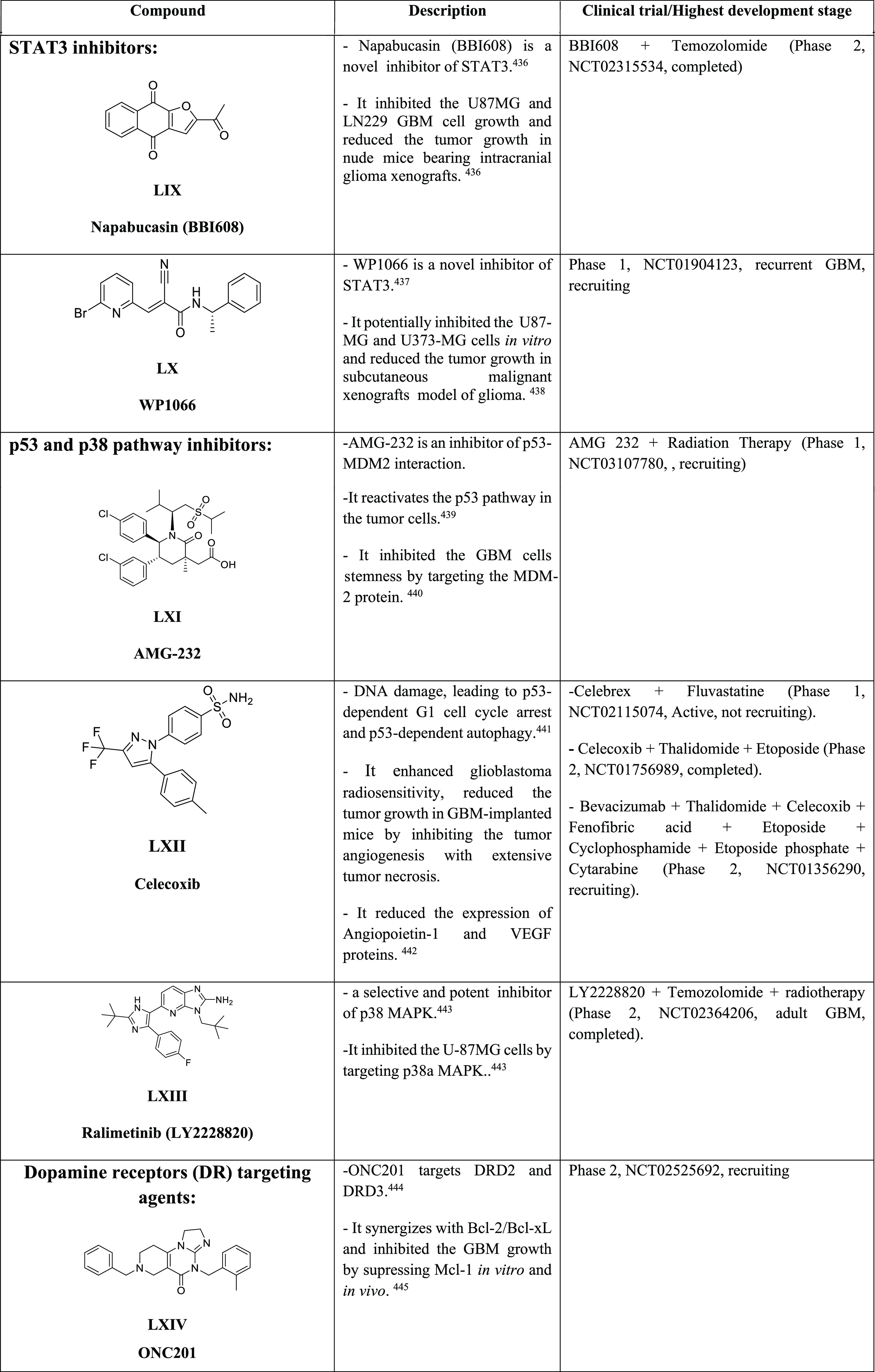

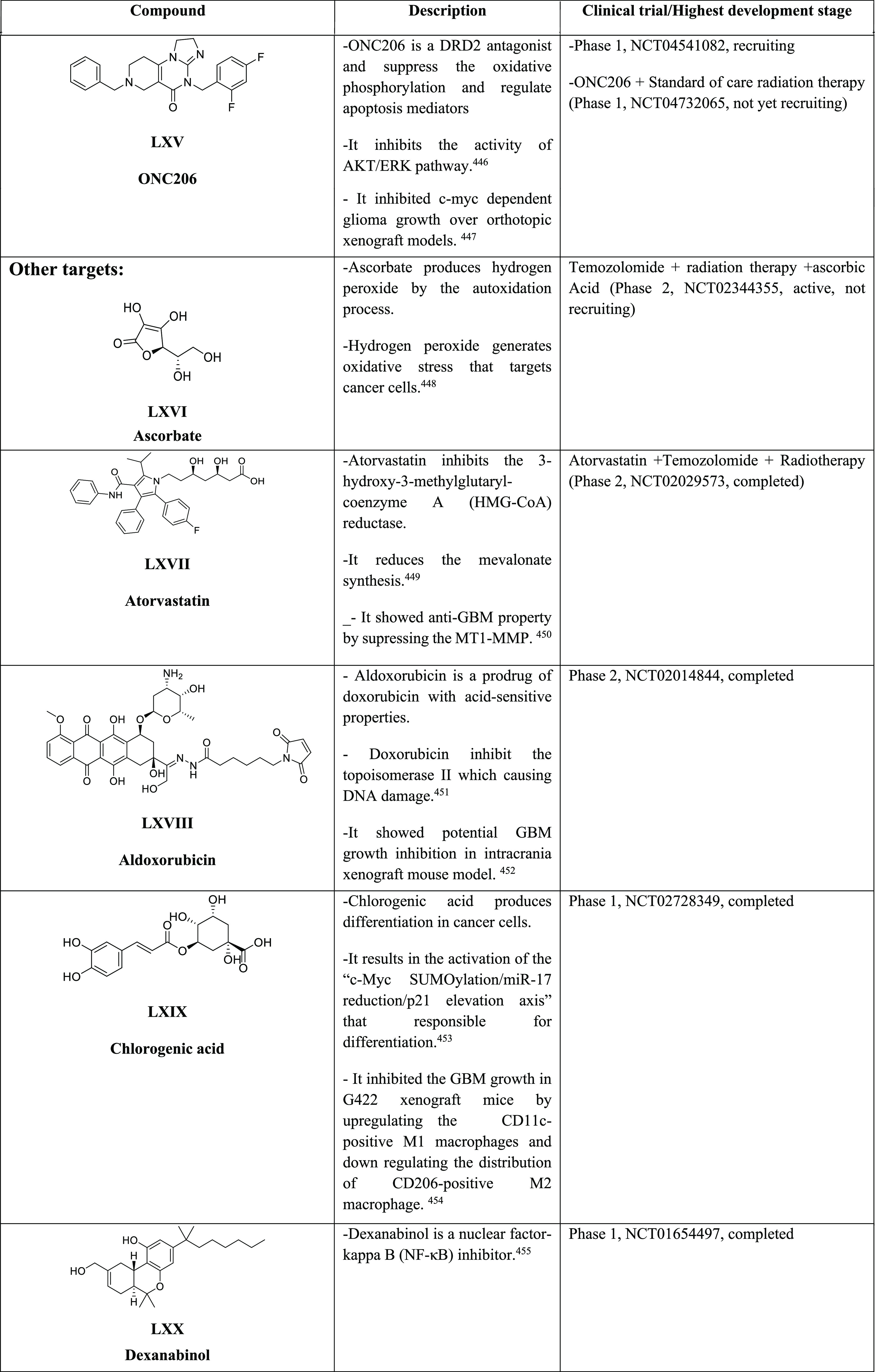

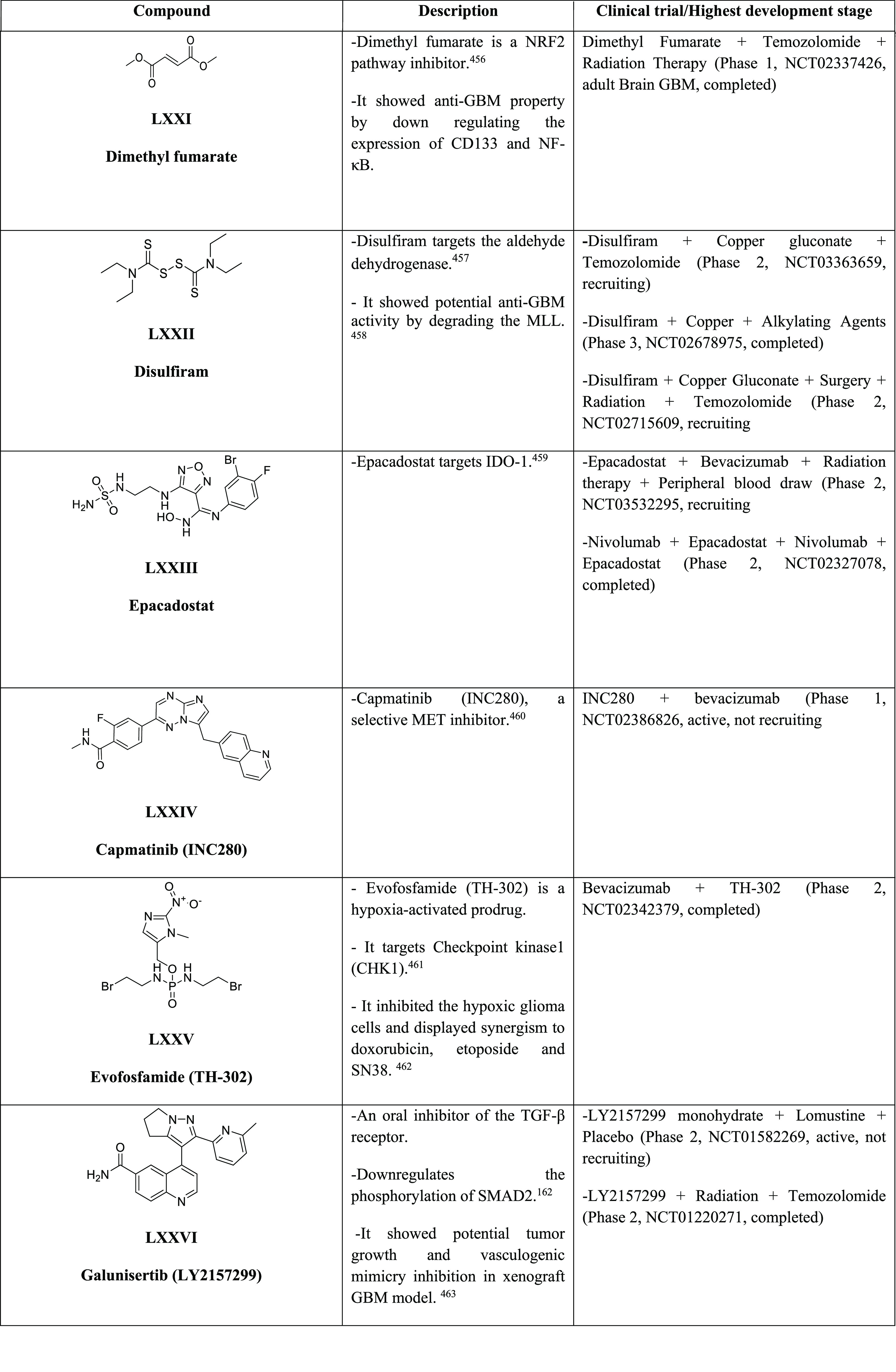

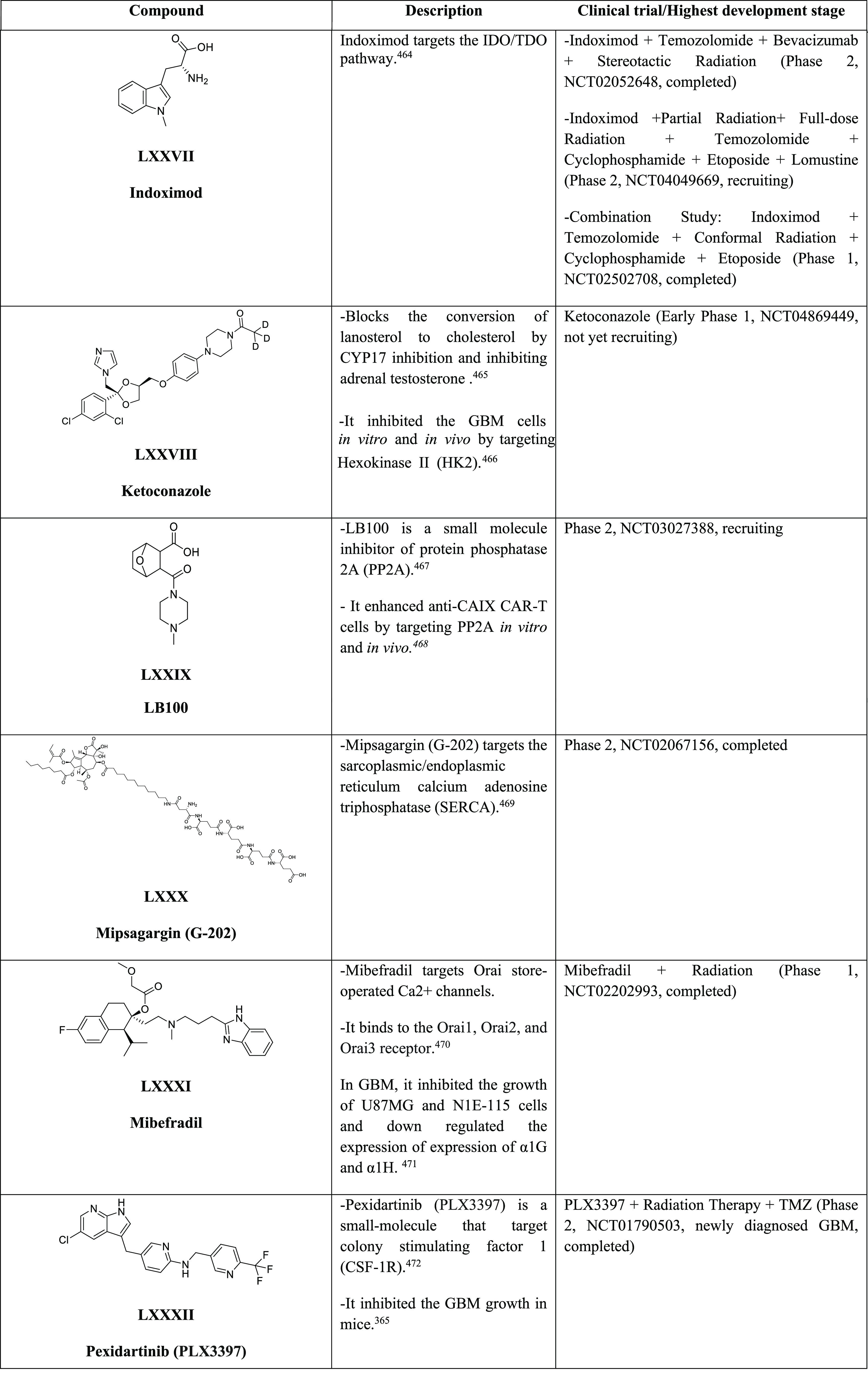

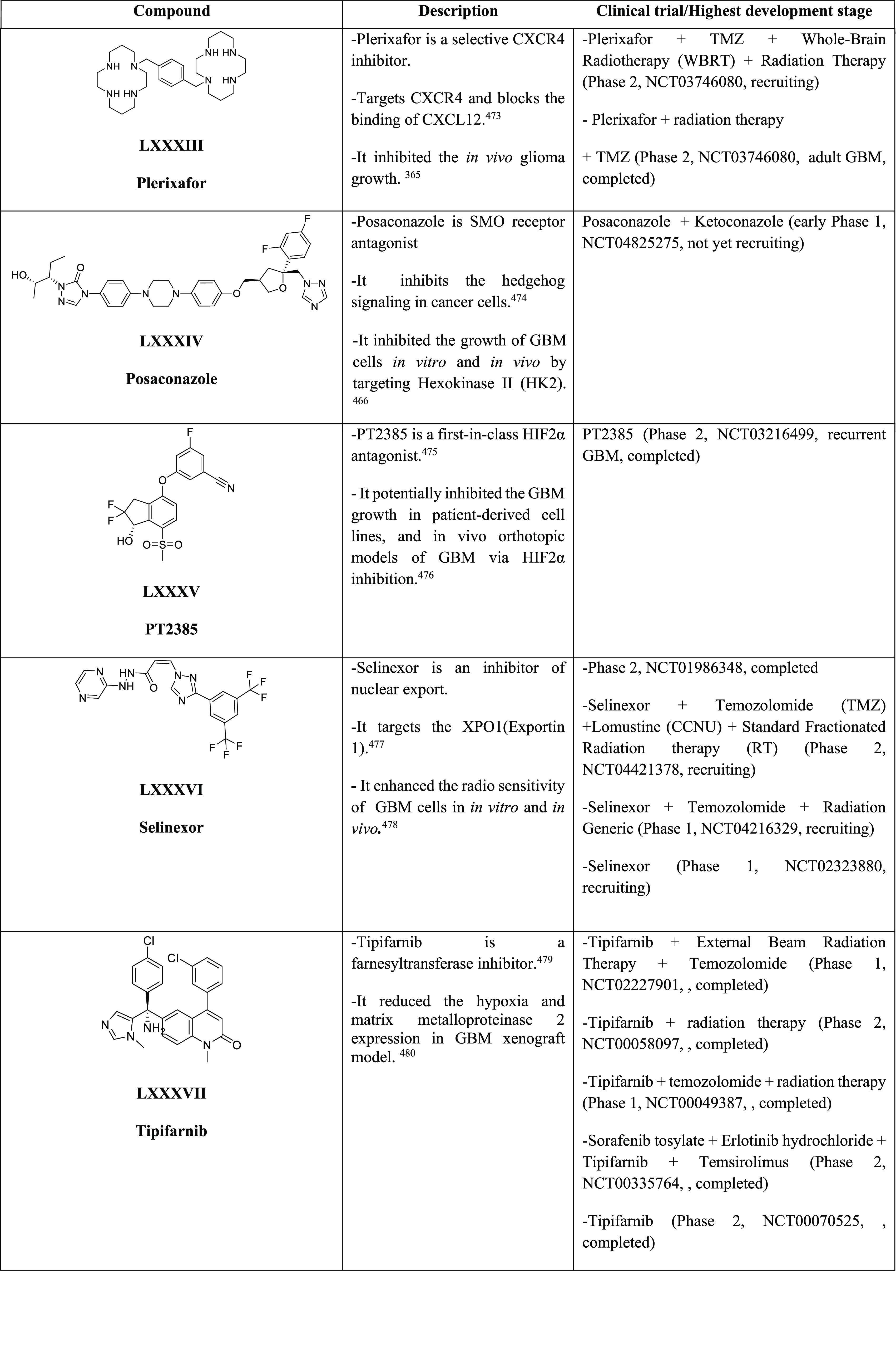
Small-Molecule Inhibitors under Clinical Trials for Glioblastoma (a)

a Data collected from https://www.clinicaltrials.gov.

Apart from diverse chemotherapeutic targets, reprogramming of GBM cells has also emerged as a potential approach that promotes the differentiation of GBM cells to neuron-like cells through transcription factor-mediated reprogramming.^326^ Notably, Asc1, Brn2, and Ngn2 (ABN) were found to be predominant transcription factors that abruptly reduced the growth of GBM cells *in vitro* and *in vivo* and promoted the conversion of GBM cells to non-divisible neurons.^327^ Recently, a study revealed the potential of small molecules to reprogram GBM cells. Lee et al. identified a cocktail of forskolin, ISX9, CHIR99021 I-BET 151, and DAPT that successfully reprogrammed malignant cells into neurons.^328^ The involvement of small molecules in GBM cell reprogramming promotes the applicability of small molecules and opens the door for medicinal chemists to design synthetically relevant reprogrammable scaffolds for GBM.

## 4 Recent Medicinal Chemistry Campaigns

Medicinal chemists have exerted numerous efforts to capitalize on the imperative revelations made by biologists regarding the involvement of factors/targets in the initiation and progression of glioma. Many of the logically constructed assemblages are currently being investigated in preliminary/preclinical explorations. This section covers the drug design strategies employed to furnish rationally assembled scaffolds, along with a discussion of the results of the cellular and enzymatic assays coupled with SAR studies, molecular modeling studies, and mechanistic insights (*in vitro* and *in vivo*) revealed during the biological evaluation of the new anti-GBM constructs.

### 4.1 Kinase Inhibitors

The PI3K/mTOR signaling pathway is important for the survival, growth, motility, and metabolism of cells.^484,485^ In the PI3K pathway, PI3K (lipid kinases) or mTOR (mammalian target of rapamycin/PI3K-related protein kinases) are activated by RTKs to generate phosphatidylinositol-3,4,5-trisphosphate (PIP3).^486^ The subsequent activation of PI3K activates the mTOR complex, namely, complex 1 [mTORC1 = mTOR + RAPTOR (regulatory-associated protein of mTOR), directly or indirectly, resulting in the division and growth of cells through the synthesis of protein due to the activation/phosphorylation of p70 ribosomal S6 kinase (S6K) and translation initiation factor 4E-binding protein (4E-BP). In addition, complex 2 [mTORC2 = RICTOR (rapamycin-insensitive companion of mTOR)] is activated by PI3K signaling along with growth factors through unknown processes, causing organization of the cytoskeleton, lipid metabolism, cell survival, and Akt kinase phosphorylation.^487−490^ The involvement of the PI3K/Akt/mTOR (PAM) pathway has been reported in GBM patients, where various signaling proteins, such as the loss of function of tensin homolog (PTEN), affect the pathogenesis of GBM along with PI3K.^485,491^ PTEN, a tumor suppressor gene, negatively regulates PIP3 levels and the PI3K/Akt pathway through a protein phosphatase that triggers mTOR activity, resulting in the proliferation and survival of the cells. Additionally, RTK/PI3K/Akt signaling pathway activation results in the stabilization of HIF1α, which leads to the development of cancer.^492^

**Figure 2:**
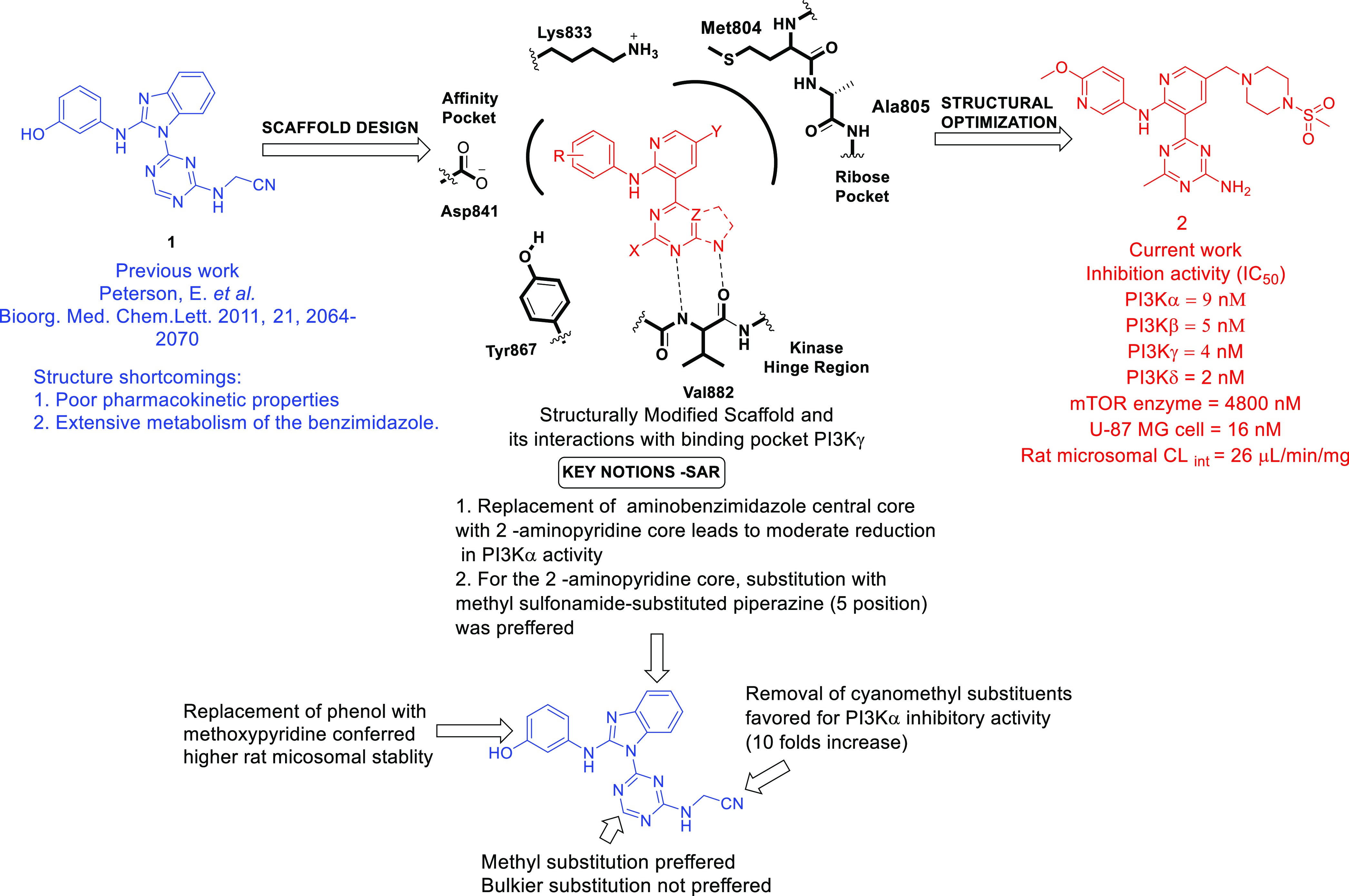
Selective class I phosphatidylinositol 3-kinases inhibitors.

Considering the activation of the PAM signaling network in GBM, Smith et al. designed a novel series of potent and selective class-I PI3K inhibitors that demonstrated striking tumor growth inhibitory potential against the U-87MG human GBM cell line (Figure 2).^33^ The group utilized a previously reported dual PI3K/mTOR inhibitor (**1**) as a chemical probe to understand the binding mode using different isoforms of PI3K. The bidentate hydrogen-bonding interaction of the triazine ring of **1** with Val882 and the hydrogen-bonding interaction of the phenolic −OH with Asp841 and Tyr867 are necessary for binding to the PI3Kγ pocket. Despite demonstrating substantial efficacy, poor PK properties and extensive metabolism of benzimidazole **1** were some of the shortcomings associated with its use, and this disclosure rendered the scope of structurally refining its chemical architecture to the authors. Given this clear understanding, a novel structure comprising a monocyclic or bicyclic hinge binder linked to a central 2-aminopyridine core was designed (Figure 2).^33^ The structure binding to the receptor showed that the monocyclic or bicyclic heterocycle at the 3 position of pyridine interacted with the hinge region amino acid Val882, and a small lipophilic substitution at position X was required to fill the hydrophobic pocket near Tyr867. According to the information available for the reported compound **1**, the amino phenol moiety was responsible for glucuronidation *in vivo*, which made it pharmacokinetically inferior. To overcome this issue, the amino phenol moiety was replaced with methoxypyridine and indazole. Additionally, alkoxycyclohexane and piperazine sulfonamide substitutions were planned to explore the ribose pocket for additional binding with Met804 and Ala805 of PI3Kγ. Subsequently, a series of designed compounds was synthesized by a multi-step synthetic route using Suzuki–Miyaura coupling reactions, S_N_Ar reactions, hydrogenation, and other chemical reactions. All the synthesized compounds were profiled for inhibitory potential toward PI3K isoforms, mTOR kinase and U-87MG (human GBM cell line). The SAR study was focused on establishing well-defined properties required to inhibit PI3Kα because of its involvement in GBM. Overall, the structural optimization as depicted in Figure 2 culminated in identifying a substantially active PI3K inhibitor (**2**). The crystal structure of **2** bound to PI3Kα also suggested that the compound displayed affinity toward the binding pocket and interacted with the major amino acids Tyr867, Asp841, Ala805, and Lys802. Furthermore, an *in vitro* PK study of **2** was performed, and the results were intriguing because **2** demonstrated a mean residual time of 1.6 h, a clearance (CL) of 1.7 L/(h·kg), and a *V*_SS_ value of 2.6 L/kg. Additionally, the hepatocyte growth factor (HGF)-stimulated PI3K signaling inhibition ability of **2** was assessed in a mouse liver pharmacodynamic (PD) assay where a lower dose of 25 mg/kg exhibited near-complete target coverage for 8 h, while a higher dose of 75 mg/kg maintained sufficient plasma concentrations for 24 h. The tumor growth inhibition potential of **2** was evaluated in a U-87MG xenograft model in CD1 nude mice at oral doses of 3, 10, 25, and 75 mg/kg q.d. Additionally, **2** exerted a dose-dependent inhibition of tumor growth with ED_50_ = 6.0 mg/kg. Furthermore, tumor stasis was achieved at 25 mg/kg q.d. At the lower dose, no tumor reduction was observed, while a higher dose reduced the tumor weight by 15% after dosing for 14 days. Given the above-mentioned findings, a daily dose of **2** for at least 8 h per day over 14 days might attain tumor inhibition >60%. Collectively, the results culminated in identifying **2** as a selective and potent PI3Kα inhibitor requiring further optimization to emerge as a drug candidate.

**Figure 3:**
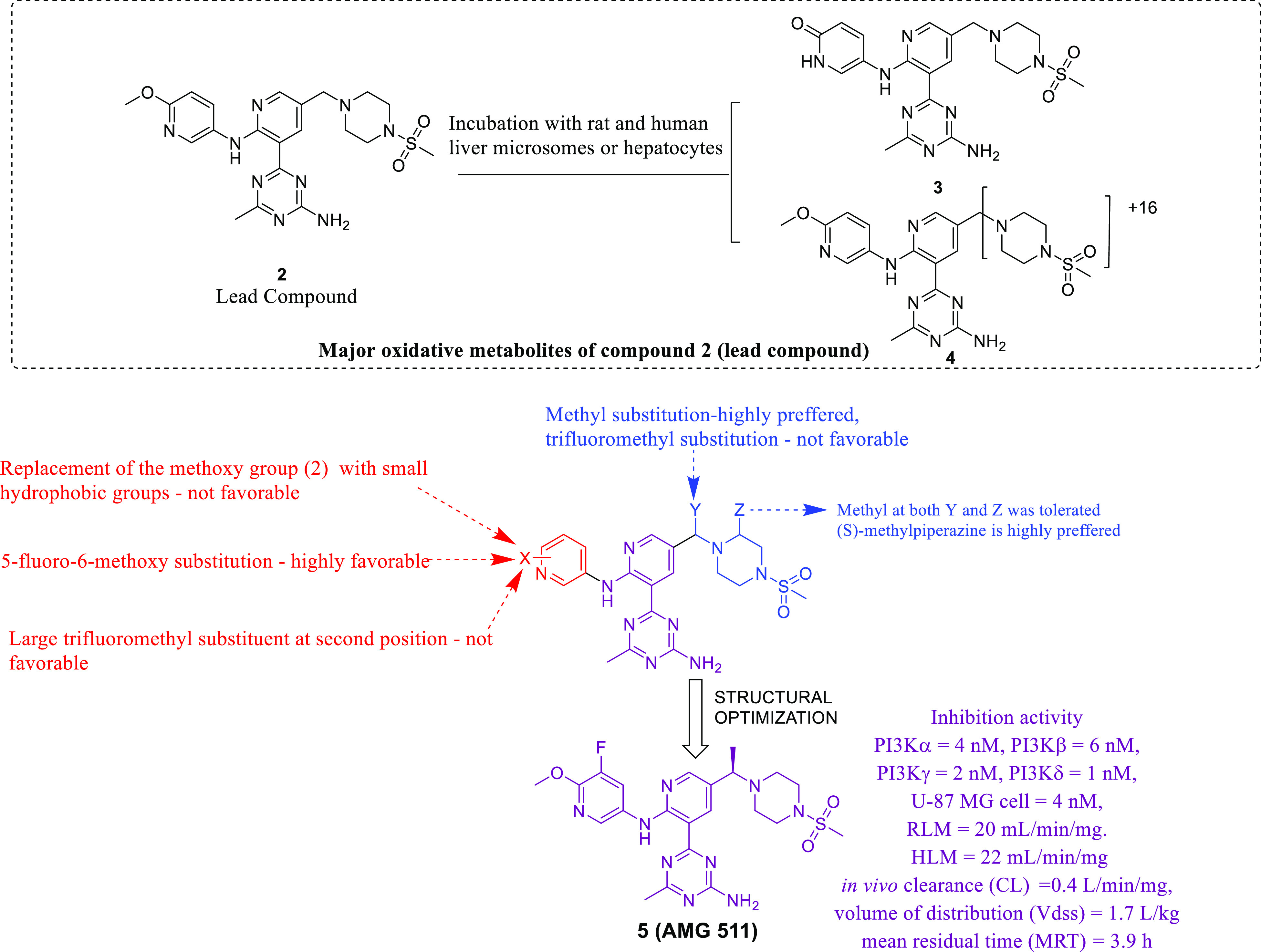
Major metabolites of lead compound and identification of clinical candidate AMG 511.

Later, the group continued this work and published a series of compounds with improved potency and PK properties (Figure 3).^34^ Initially, **2** was investigated comprehensively by administering an intravenous dose to bile-duct-cannulated rats, and the drug was quantified in excreta (urine, bile, and feces) for up to 24 h. Quantification of the drug in urine, bile, and feces showed that only 2.5% of the drug was excreted in its parent form, which suggested that metabolism was the major clearance pathway. Therefore, metabolite studies were performed to identify the metabolites by incubating **2** with rat and human liver microsomes or hepatocytes. LC-MS analysis revealed that most of the metabolites were formed due to oxidative metabolism. Additionally, two metabolic pathways were identified at the methoxypyridine and benzylic piperazine regions that led to metabolites **3** and **4**. Based on these revelations, compounds with improved PK properties needed to be developed. Thus, a series of compounds was synthesized by modifying the metabolic spots (vulnerable sites) of the structure. A total of 21 compounds were synthesized and evaluated against the PI3Kα, PI3Kβ, PI3Kγ, PI3Kδ, and U-87MG human GBM cell lines. Among the synthesized compounds, **5** was the most active, with IC_50_ = 4 nM (PI3Kα), 6 nM (PI3Kβ), 2 nM (PI3Kγ), 1 nM (PI3Kδ), and 4 nM (U-87MG). Additionally, a rat and human liver microsomal (RLM and HLM) study was performed. Compound **5** displayed excellent results with RLM and HLM values of 20 and 22 μL/min/mg, respectively. The SAR was evaluated for two different metabolic spots (oxidation regions) of the compound, as shown in Figure 3. Based on the results of the SAR study, the PK profiles of a set of compounds were evaluated, and **5** showed an attractive PK profile because it displayed the lowest *in vivo* clearance (0.4 L/kg/h), a high volume of distribution (1.7 L/kg), and a moderate mean residual time (3.9 h). Furthermore, a PD study of **5** was performed in a mouse liver PD model. Compound **5** was administered orally at doses of 3, 10, and 30 mg/kg, and HGF was administered after 6 h to activate PI3K-dependent Akt phosphorylation in the liver. The results revealed that **5** suppressed PI3K signaling in a dose-dependent manner, and the plasma EC_50_ was 228 ng/mL. Tumor inhibition activity was evaluated in a mouse U-87MG glioblastoma xenograft model in which **5** was administered at doses of 1, 3, and 10 mg/kg for 12 successive days. After treatment, a significant reduction in tumor growth (approximately 70%) was observed at a dose of 1 mg/kg q.d., and the ED_50_ was deduced to be 0.6 mg/kg. Overall, the study led to the identification of a new PI3Kα inhibitor that was selected for further clinical evaluation in the treatment of cancer and was named AMG 511.

**Figure 4:**
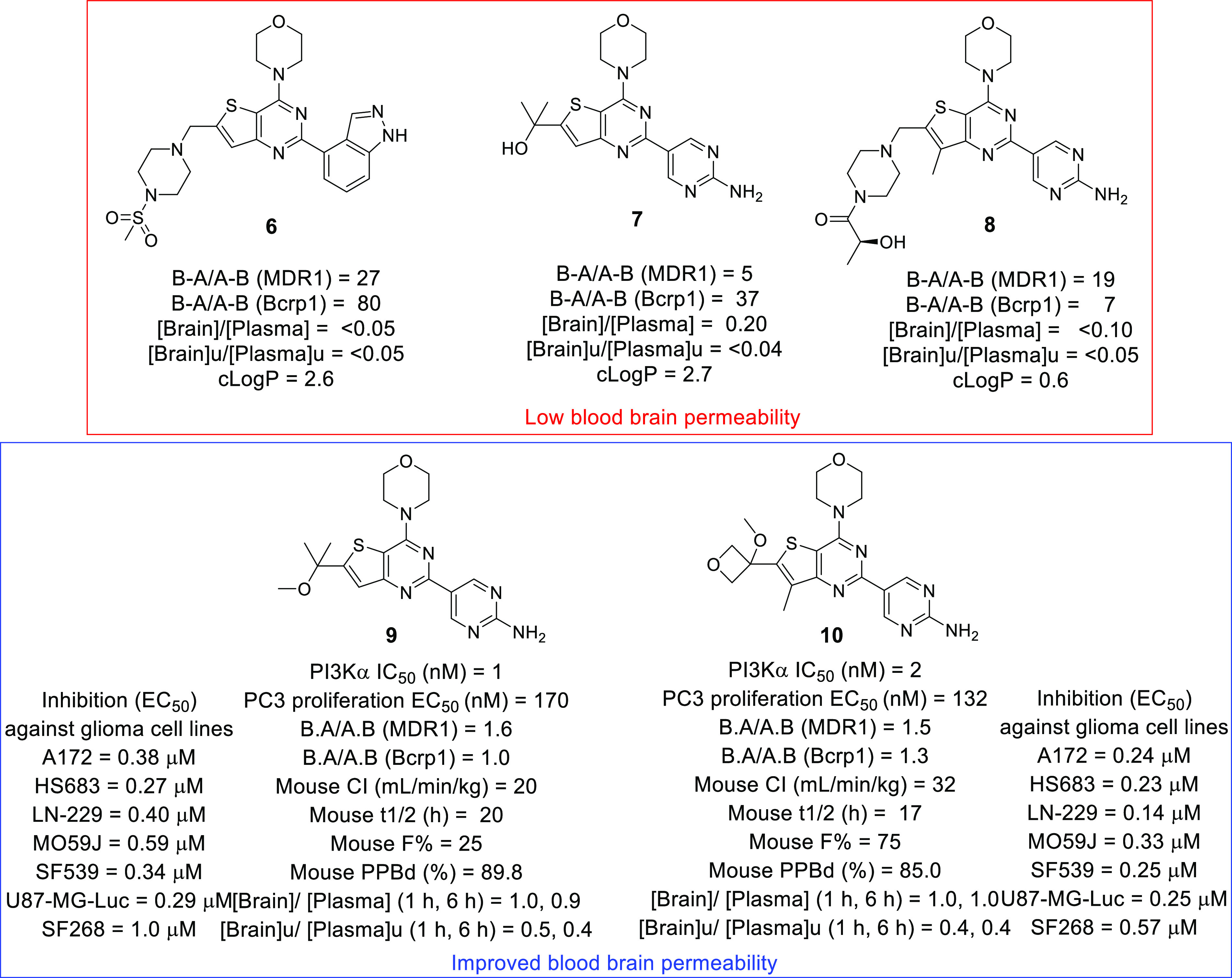
PI3K inhibitors with improved blood–brain penetration for the treatment of GBM.

In 2012, Heffron et al. synthesized a new PI3Kα to overcome the issue of the low BBB permeability encountered with the previously identified compounds, pan-PI3K inhibitor GDC-0941 (**6**) and dual PI3K/mTOR inhibitors GNE-493 (**7**) and GDC-0980 (**8**) (Figure 4).^35^ Among the synthesized compounds, **9** and **10** were the most potent in the series, with *K*_i_ = 1 and 10 nM and 2 and 9 nM against PI3K-α and mTOR, respectively. **9** and **10** showed substantial anti-proliferative activity against PC3 cell lines (EC_50_ = 170 and 132 nM, respectively) along with improved B-A/A-B (MDR1) B-A/A-B (Bcrp1) mouse CI, mouse *t*_1/2_, and mouse *F*% values (Figure 4). Furthermore, the compounds were evaluated in a panel of seven GBM cell lines, A172, HS683, LN-229, MO59J, SF539, U-87-MG-Luc, and SF268. The results were overwhelmingly positive because both **9** and **10** displayed impressive potency, with an EC_50_ range of 0.23–1 μM. Additionally, a U-87 subcutaneous xenograft study of **9** and **10** was performed, revealing the tumor growth inhibitory/tumor weight reduction potential of both compounds. Furthermore, the compounds influenced the expression of the PI3K pathway markers pAKT and pS6RP, indicating the targeted action of the compounds. Altogether, the above-mentioned compounds were efficacious against GBM cell lines with improved BBB permeability.

**Figure 5:**
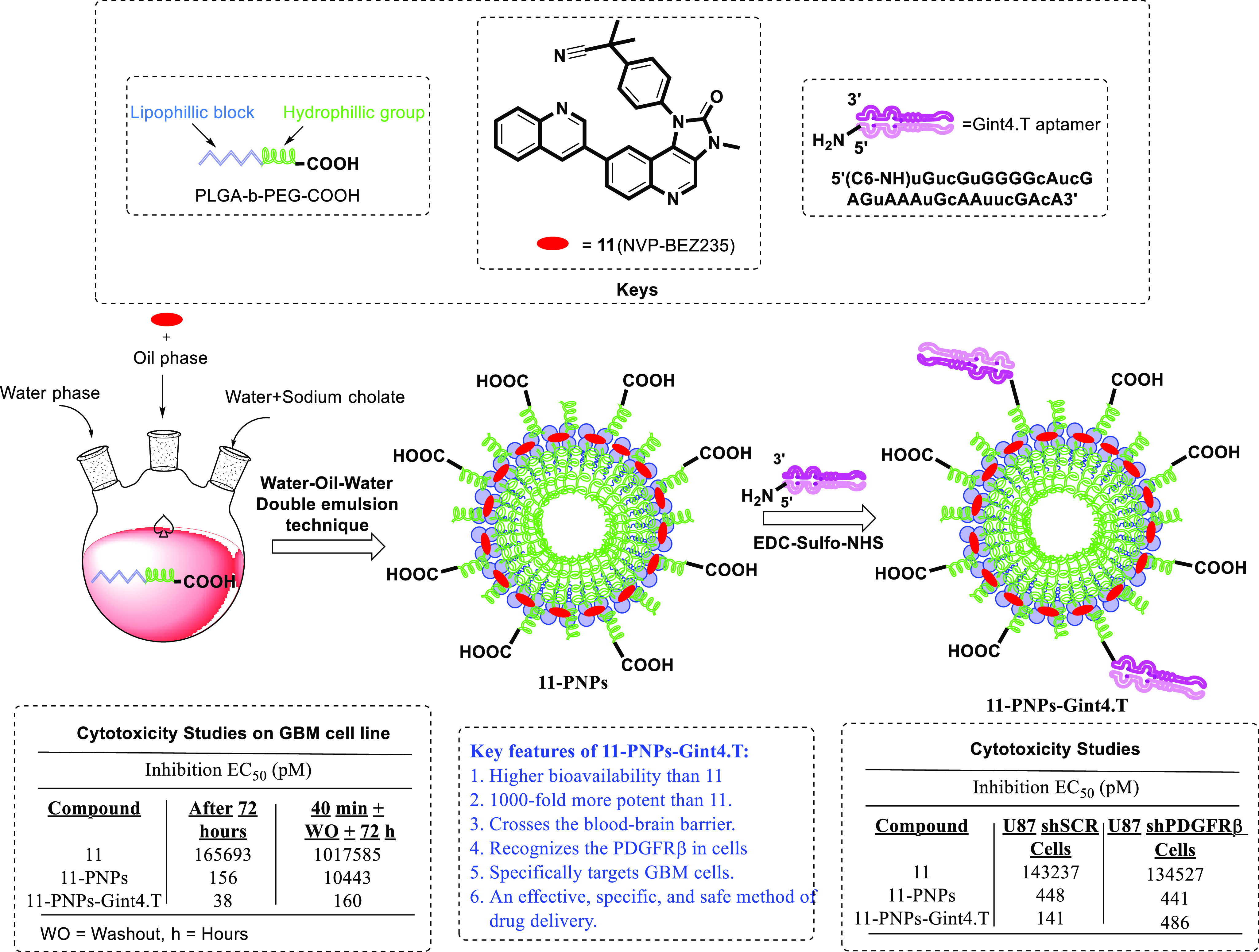
Aptamer-functionalized nanosystems for GBM.

In 2017, Monaco et al. introduced a method for aptamer functionalization of nanosystems that targets GBM through the BBB.^36^ The group fused an anti-PDGFRβ aptamer with biodegradable polymeric nanoparticles (PNPs) to deliver a promising chemotherapeutic agent, dactolisib (**11**, NVPBEZ235) (Figure 5). Dactolisib (**11**) is a potent dual PI3K-mTOR inhibitor under investigation for the treatment of solid tumors and was recently proven to be an efficacious radiosensitizer and chemosensitizer in a preclinical mouse GBM model. Despite its promising activity profile, the poor water solubility of **11** affects its bioavailability, and a high dose is required to achieve a therapeutic effect. Therefore, the water-in-oil-in-water double-emulsion sonication method was used to entrap the drug, followed by amino-terminated conjugation of the anti-PDGFRβ aptamer Gint4.T to a COOH group of the nanosystem. The resultant formulation of **11**-PNPs-Gint4.T was characterized by dynamic light scattering (DLS), where the diameter of the particles was 52 ± 1 nm, with a polydispersity index of 0.169. The amount of aptamer conjugated to the PNPs was evaluated by RT-qPCR analysis in which the concentration of Gint4.T and the conjugation efficiency were 1.4 nM and 5.4%, respectively, with an overall **11**-PNPs-Gint4.T concentration of 18.4 mg/mL. To check the targeting efficiency of the formulation, *in vitro* internalization studies were performed by fusing Gint4.T or scrambled (SCR) aptamers on U-87MG cells. Gint4.T aptamer-loaded formulations with PNPs specifically targeted GBM cells and actively enhanced intracellular uptake. Furthermore, *in vitro* cytotoxicity studies were performed against the GBM cell line, and **11**-PNPs-Gint4.T displayed 1000-fold higher cytotoxicity than free drug **11** (Figure 5). The Gint4.T aptamer specificity was further studied in shSCR and shPDGFRβ U-87MG cells, revealing that **11**-PNPs-Gint4.T was 6500-fold more toxic than **11**, with EC_50_ = 141 and 486 pM, respectively. Additionally, the specific tumor-targeting potential was evaluated by administering Gint4.T PNPs to nude mice bearing intracranial U-87MG tumor xenografts. High-resolution imaging revealed that the anti-PDGFRβ aptamer allowed the nanoparticles to cross the BBB and target glioma cells. Furthermore, the tumor-specific targeting potential was evaluated in mice with brain tumors by delivering **11**-PNPs-Gint4.T for 5 successive days. After treatment with **11**-PNPs-Gint4.T, the mouse brain was again treated with phospho-4EBP1, which lowered the **11**-PNPs-Gint4.T concentration in the brain, indicating the tumor-specific binding of **11**-PNPs-Gint4.T. In conclusion, the aptamer-based nanosystem crossed the BBB and targeted PDGFRβ-expressing glioma cells in the brain, making it an effective delivery system for tumors.

**Figure 6:**
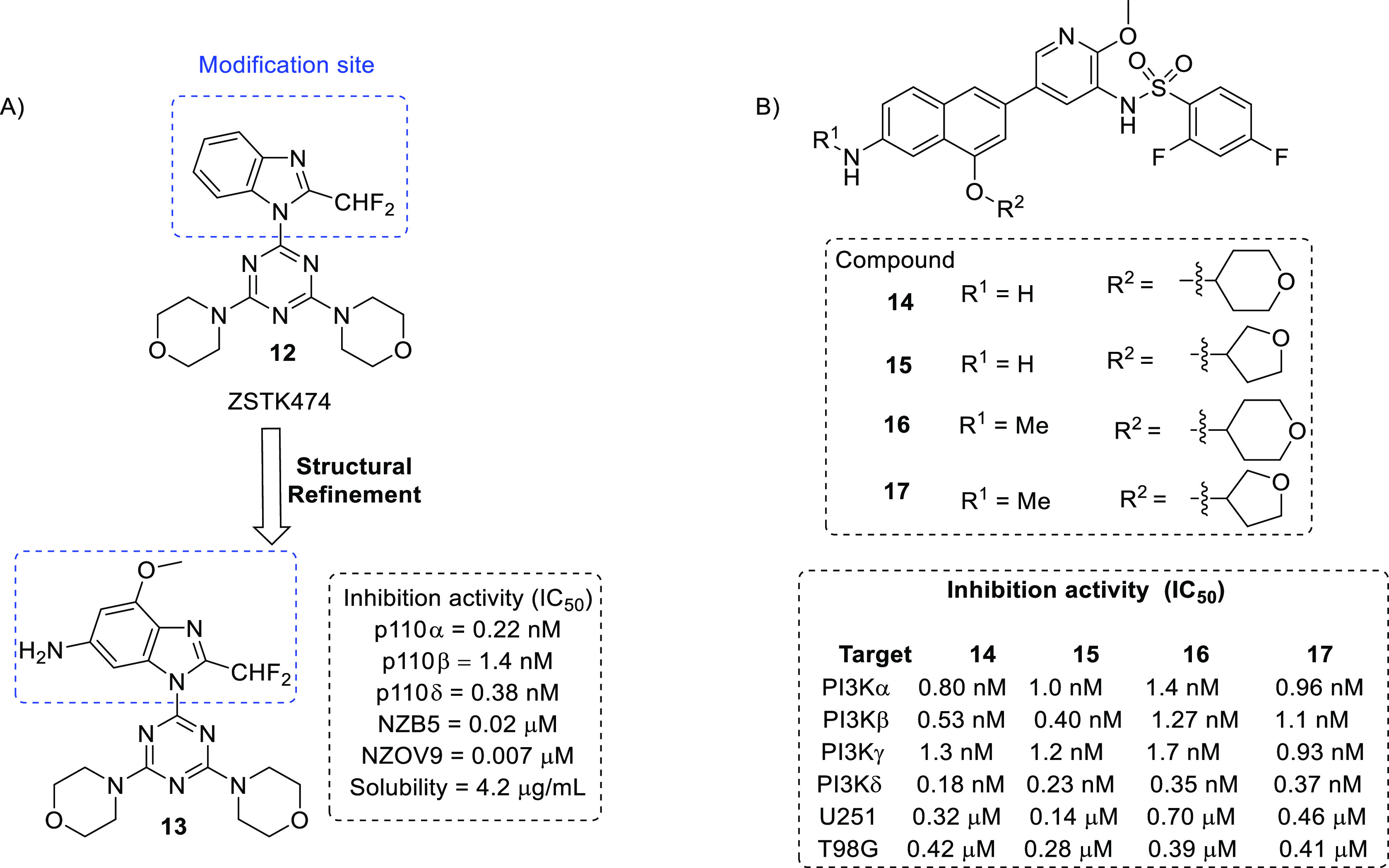
(A) Structural modification of ZSTK474 as PI3K inhibitors. (B) 2-Amino-4-methylquinazoline derivatives as potential PI3K inhibitors.

In 2011, Rewcastle et al. published a SAR study of a class 1 PI3K inhibitor (ZSTK474, **12**) for anti-GBM activity.^37^ The structural alteration program led to the identification of a potent compound bearing a 6-amino-4-methoxy substitution at the benzimidazole ring (Figure 6A). Subsequent evaluation conducted in Rag1–/– mice bearing a U-87MG human GBM tumor xenograft model revealed that **13** significantly inhibited the growth of tumors up to 81% at a dose of 50 mg/kg (i.p. injection) for 10 (q.d.) days; however, the solubility profile of the compound was unfavorable.

In 2018, Lin et al. reported a series of 2-amino-4-methylquinazoline derivatives as potential PI3K inhibitors furnished through scaffold hybridization and hopping strategies.^38^ Among the synthesized compounds, **14**–**17** displayed effective inhibition activity against PI3K isoforms and glioma cell lines (Figure 6B). Furthermore, **14** showed exceptional kinase selectivity against 458 kinases, with an S(1) score of 0.015. Additionally, **16** and **17** displayed significant tumor growth inhibition of >90% in the U-87MG brain xenograft model and showed acceptable safety profiles.

**Figure 7:**
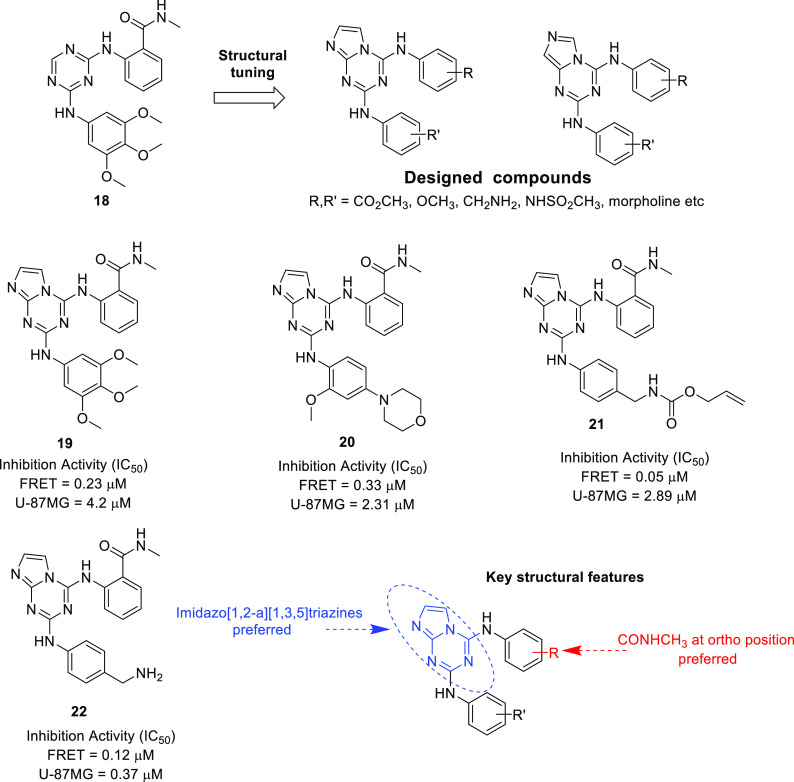
FAK inhibitors as anti-tumor agents.

Focal adhesion kinase (FAK/PTK2) is a tyrosine kinase that is present in the cytoplasm and is responsible for focal adhesions involving the dynamics of cellular migration by linking the actin cytoskeleton with integrin. FAK regulates the survival, proliferation, migration, invasion, and microenvironment of tumor cells like angiogenesis.^493−498^ Tamura et al. revealed the involvement of phosphatase and tensin homolog (PTEN) in the dephosphorylation of active FAK at Y397 in GBM cell lines.^498^ In the active and phosphorylated state, it increases the expression of CCND1/cyclin-D1 and decreases the levels of p21/CDKN1A cyclin-dependent kinase (CDK) inhibitor, causing enhanced proliferation of cells through accelerated transition from the G1-S phase.^499^ Mamillapalli et al. found that PTEN negatively regulated the G1/S phase transition by obstructing S-phase kinase-associated protein-2 expression (SKP2) and ultimately alleviating the levels of p27/CDKN1B.^500^ In GBM, loss of PTEN causes the activation of FAK and apoptotic resistance due to the absence of contact (cell–matrix). According to a study conducted by Alza et al., PF-573228 (an FAK inhibitor) arrests cell proliferation, increases the size of cells, and diminishes neurosphere growth in GBM due to an increase in the levels of β-galactosidase and p27/CDKN1B activity.^501^ The inhibition of FAK also reduces p62/SQSTM-1 expression (autophagy cargo receptor), stimulating p27 transcriptional upregulation (senescent-like phenotype) and leading to proliferation arrest and cell death. Based on the evidence, in 2014, Dao et al. designed a novel series of imidazo[1,2-*a*][1,3,5]triazine derivatives via structural modification of the previously reported potent FAK inhibitory compound **18 (**PHM16), which showed striking anti-tumor activity (Figure 7). In total, 26 regioisomers of imidazo[1,2-*a*][1,3,5]triazines were synthesized and evaluated for FAK activity using a TR-FRET kinase assay. Among the synthesized compounds, **21** was the most potent, with IC_50_ = 50 nM. The SAR study of compounds revealed that the incorporation of imidazo[1,2-*a*][1,3,5]triazine was extremely beneficial for the activity (Figure 7). Furthermore, molecular docking of the most potent compound, **21**, was performed using apo-FAK kinase (PDB ID 4C7T). The binding poses showed that the compound fit well in the binding pocket and interacted with the major amino acids of the binding pocket, Met499, Asp564, Leu567, and Ile428. Furthermore, the selected compounds were evaluated for FAK autophosphorylation ability and growth inhibition potential toward U-87MG cell lines. All the tested compounds showed promising anti-proliferative activity in the mentioned cell lines, with IC_50_ values in the low micromolar range. Additionally, **21** and **22** delayed the progression of the cell cycle and arrested the cell cycle at the G2/M phase in the U-87MG glioma cell line. Furthermore, **19**–**22** inhibited the cell matrix adhesion, migration, and invasion of U-87MG cells. Collectively, these findings underscore the magnificent activity profile of the compounds against human GBM.^39^

**Figure 8:**
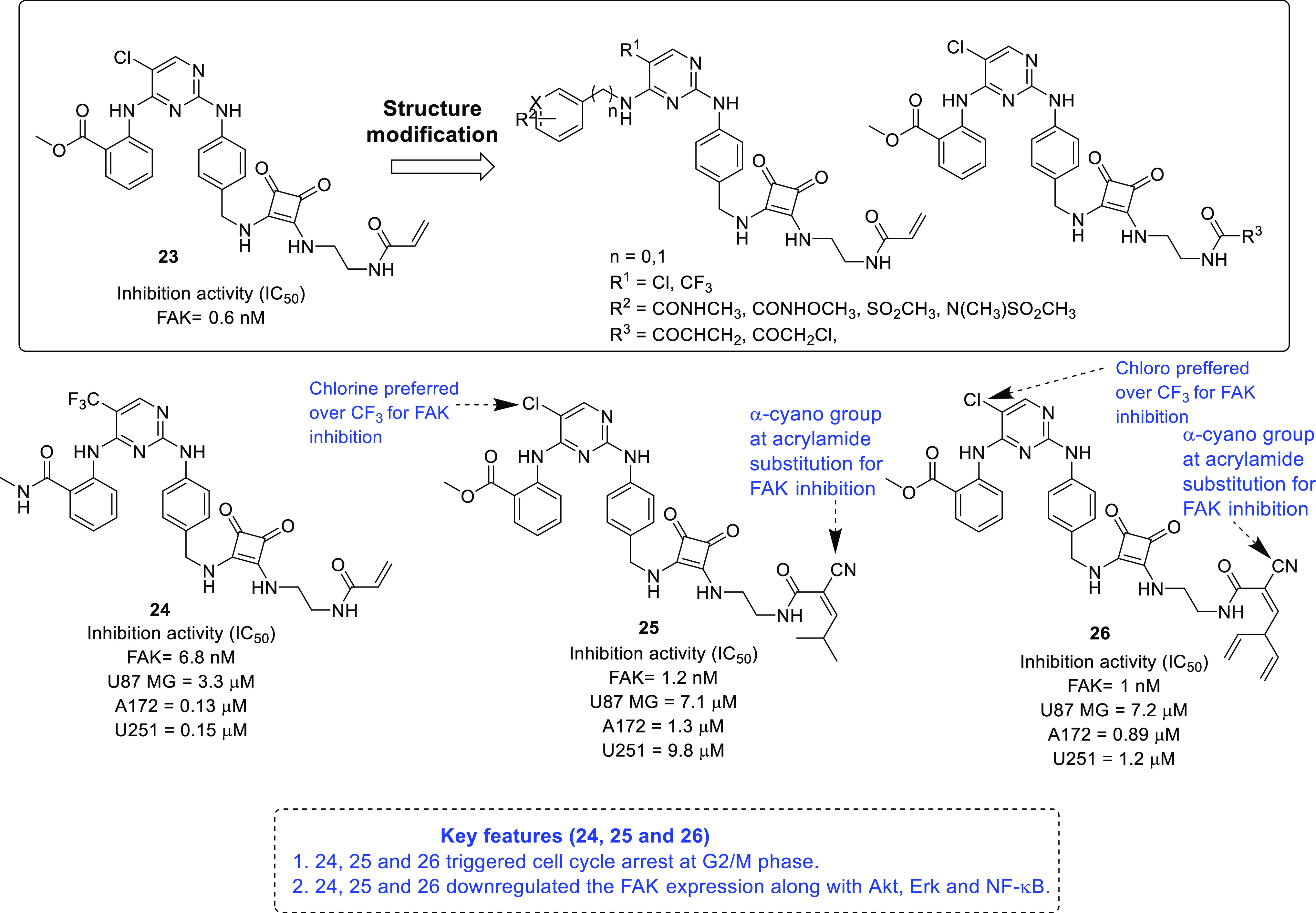
FAK inhibitors as potential anti-GBM agents.

In 2020, Li et al. reported some FAK inhibitors for the treatment of malignant glioma.^40^ In their study, **23**, a previously reported FAK inhibitor, was employed as the lead compound, and two different series of compounds (Figure 8) were furnished by a multi-step synthetic route. All the synthesized compounds were initially evaluated employing a FAK enzymatic assay. Gratifyingly, the compounds demonstrated strikingly promising inhibitory potential with an IC_50_ range of 0.6–16.3 nM. Furthermore, a SAR study was performed, and the results are illustrated in Figure 8. The acrylamide moiety was beneficial for the activity because its replacement with chloromethyl ketone led to reduced activity of the compound. The selected compounds were also evaluated using the kinase selectivity assay of a panel of 10 kinases (Akt, c-Src, PDGFR, c-kit, IGF1R, FGFR1, EGFR, IR, Erk, and Pyk2), and the compounds demonstrated selectivity toward FAK and PyK2 enzymes. The anti-GBM efficacy of the compounds was tested against U-87MG, A172, and U251 cell lines, where **24**–**26** inhibited cell growth at low nanomolar concentrations. Furthermore, the FAK-mediated anti-proliferative activities of the compounds were tested in U-87MG cell lines. The compounds inhibited the growth of U-87MG cells at 3 μM, and the activity was confirmed to be mediated by FAK inhibition, as demonstrated by Western blot analysis. Additionally, the mechanism of the compounds was determined using flow cytometry, which suggested that the compounds triggered cell cycle arrest at G2/M phase. Furthermore, the compounds reduced cell migration and downregulated the expression of FAK along with Akt, Erk, and NF-κB.

**Figure 9:**
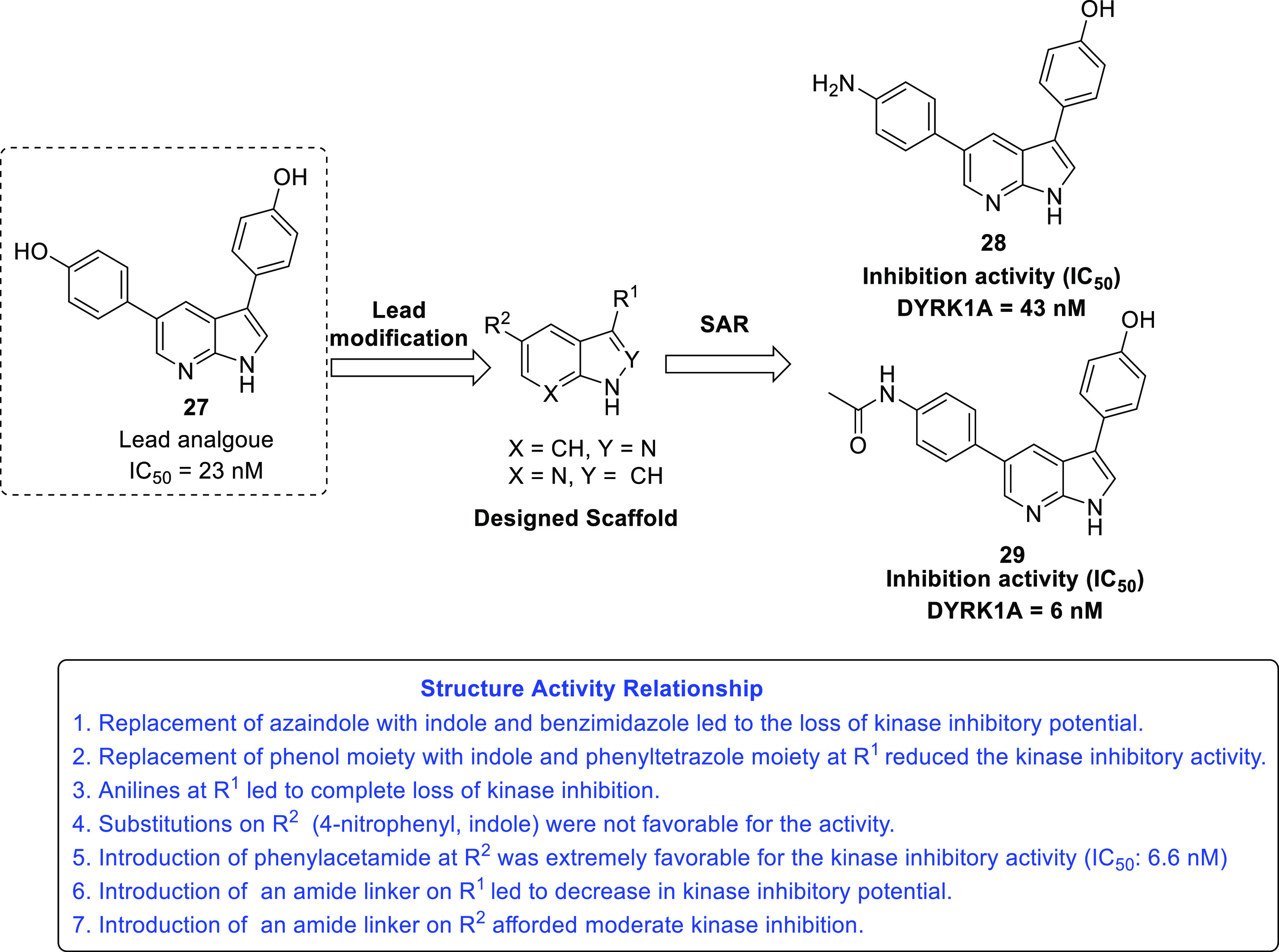
DYRK as a target for the treatment of GBM.

Reports investigating the upregulated expression of the dual specificity tyrosine phosphorylation regulated kinases (DYRK) in some malignancies prompted a research group to employ a lead modification strategy and design a series of novel 7-azaindole derivatives as DYRK inhibitors (Figure 9). All compounds were evaluated against DYRKIA, DYRKIB, DYRK2, and the structurally related CLK1. Structural explorations were conducted on lead structure **27** to improve its activity profile, and the notions found to be critical for the activity are depicted in Figure 9. Specifically, the bioisosteric replacement strategy was utilized for the structural alteration at the C3 and C5 positions of the lead compound. The cell-based assay was performed using the RN1 and WK1 cell lines (GBM cell lines), and it was found that compounds **28** and **29** were strikingly efficacious toward both GBM cell lines. Furthermore, compound **28** was evaluated for EGFR degradation, clonogenic cell survival, migration, and invasion assays, and its activity profile was found to be extremely promising, with the inhibitory potential evidenced at the low micromolar range. In addition, the results of the cellular thermal shift assay (CETSA) demonstrated the ability of compound **28** to penetrate into cells and bind to DYRK1A. As such, compound **28** was endowed with excellent DYRK1A inhibitory activity (IC _50_ = 43 nM) and appears to be a suitable chemical tool for future campaigns.^42^

**Figure 10:**
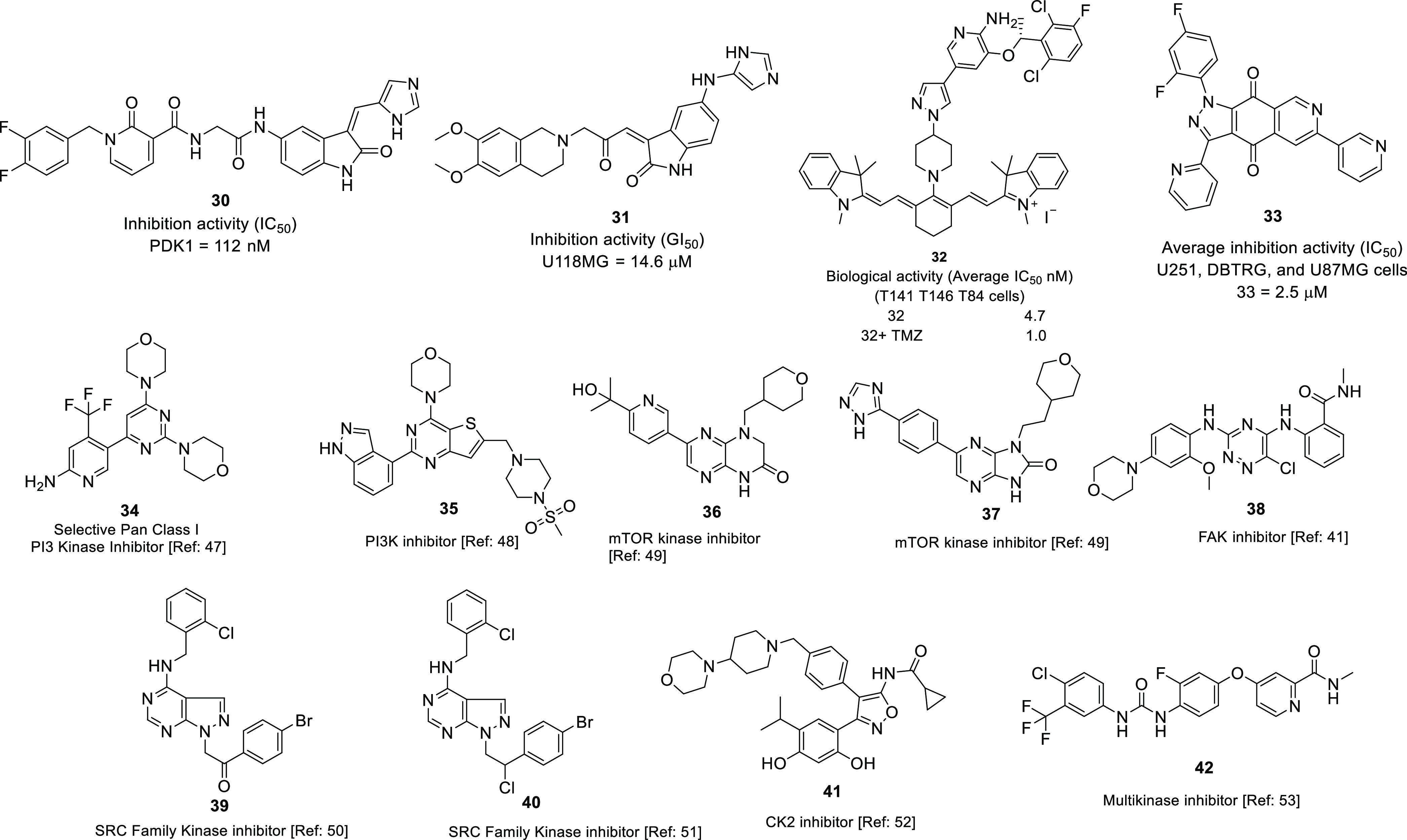
Kinase inhibitors of various classes as potential anti-glioma agents.

PDK1-a Ser/Thr kinase inhibits the formation of acetyl-CoA from pyruvate to stimulate the progression and formation of GBM.^502,503^ PDK is essential to producing lactate from pyruvate and can be used as a therapeutic target along with standard therapy. Various studies have shown the association of PDK1 with the progression of cancer. The overexpression of PDK1 has been observed in gastric cancer due to HIF-1α and the maintenance of melanoma cells.^504,505^ Additionally, the elevated expression of PDK1 has been observed in head and neck carcinoma and non-small-cell lung cancer and in specimens of human GBM, and silencing PDK1 produced significant anti-proliferative and apoptotic effects on 5310 and U251 cells.^503,506,507^ Due to the role of PDK1 in the progression of GBM, Sestito et al. revealed a series of 2-oxindole derivatives as putative PDK1 inhibitors.^43^ A total of 16 compounds were synthesized, where **30** was the most potent in the series, with IC_50_ = 112 nM (Figure 10). Furthermore, **30** inhibited the growth of GSCs isolated from the U-87MG cell line, with IC_50_ = 3.36 ± 0.40 nM and suppressed tumor cell migration. In 2015, Sestito et al. used a series of 2-oxindole-based compounds as PDK1/Akt signaling pathway inhibitors.^44^**31** was identified as the most promising compound against the U118MG cell line, with GI_50_ = 14.6 μM. Additionally, **31** displayed a multi-targeting effect by inhibiting CHEK1, GS3Kα GS3Kβ, and PDK1, with IC_50_ = 274, 884, and 272 nM (each at 10 μM) and 998 nM (at 25 μM), respectively, and induced differentiation among CSCs. The low efficacy of heptamethine cyanine dyes (HMCDs) toward brain cancer cells is an uphill battle. In order to confront the low efficacy, Choi et al. introduced a conjugate of Crizotinib and heptamethine cyanine dye IR-786 which showed potential cytotoxicity against T141, T146, and T84 GBM cell lines.^45^ In an EdU cell proliferation assay, **32** displayed promising anti-proliferative activity, with IC_50_ = 4.7 nM. Interestingly, **32** showed the synergism with TMZ which enhance the potency of compound by 4-fold. Overall, the introduction of **32** intensifies the applicability of heptamethine cyanine dyes (HMCDs) for the GBM. In 2018, Bertuzzi et al. published an efficient synthesis of quinone-fused pyrazoles through 1,3-dipolar cycloadditions as prudent anti-GBM agents.^46^ Various substituted quinone-fused pyrazoles were synthesized with a good yield and evaluated over a panel of U251, DBTRG, and U-87MG GBM cell lines. Among all, **33** displayed potential anti-GBM activity, with IC_50_ = 2.5 μM. Further, the docking studies and biological studies revealed that the **33** possibly inhibits the PI3K/mTOR kinase which is a responsible cofactor of the cancer development. The chemical structures of other potent kinase inhibitors with anti-glioma potential are also presented in Figure 10.^41,47−53^

### 4.2 HDAC Inhibitors

GBM is the most lethal and malignant brain tumor (grade IV) due to the presence of CSCs or tumor-initiating cells, epigenetic mechanisms, and cellular pathways.^508^ The most noticeable epigenetic changes in tumor cells are hyperacetylation/hypomethylation of oncogenes and hypoacetylation/hypermethylation of tumor suppressor genes.^509^ Bezecny et al. reported a mutation in 60% of pediatric glioma cases (Lys 27-to-methionine (K27M)) at one allele of H3F3A and one of the two genes encoding histone H3 variant H3.3, signifying the role of modifications on histone and DNA in GBM through tumor initiation, progression, and resistance to treatment.^510^ Under normal circumstances, histone proteins are responsible for the modulation of chromatin structure/function and the expression of genes. The modifications of histone tails after the translation process include acetylation, ubiquitination, phosphorylation, and methylation and regulate the remodeling of chromatin.^508−512^ Histone acetyl transferases (HATs) transfer acetyl moieties to lysine residues, and HDACs remove them. HATs promote gene transcription and expression, whereas HDACs suppress them and regulate gene expression by directly interacting with transcription factors, such as protein 53, E2f, activator and signal transducer of transcription 3 (Stat3), transcription factor IIE (TFIIE), nuclear factor kappa B (NF-κB), and retinoblastoma protein. In addition, HDACs deacetylate non-histone proteins that are responsible for maintaining homeostasis in cells (apoptosis, progression of the cell cycle, and differentiation) and become abnormal in tumor cells.^513,514^ Lucio-Eterovic et al. revealed that H3 histones are hyperacetylated in GBM and on the progression of astrocytomas to GBM; however, class II and IV HDACs were not found to be expressed, indicating that class II and IV HDACs are amenable to the progression of astrocytoma to GBM.^515^ Moreover, the differential/dysregulated expression of HDAC4, 6, and 8 has been associated with resistance to standard treatment in GBM CSCs due to distorted signaling mechanisms, including the sonic hedgehog (SHH) pathway (crucial for viability, radioresistance, and stemness) and correlates with glioma progression.^515−517^

Furthermore, enhanced levels of class III (NAD-dependent) HDACs, SIRT1/2, have been reported in CSCs of GBM. SIRT1 knockdown enhances the radiosensitivity of GSCs and reduces tumor volume with a positive therapeutic outcome on CD133-positive GBM tumors.^518,519^ Sathornsumetee et al. also reported increased expression of HDACs (1, 3, 6, and 9) in GBM. HDAC inhibitors are used to re-establish the balance of HAT to HDAC activity and sensitize tumor cells to HDAC inhibitors as monotherapeutic agents and in combination with radiation therapy. HDAC inhibitors are reported to be valuable in GBM therapy in preclinical phases, as they enhance tumor cell sensitivity to DNA alkylating chemotherapeutic agents through open chromatin conformation in tumor cells and help reverse abnormal genetic silencing in GBM, leading to the enhanced arrest of the cell cycle and apoptosis.^520^

**Figure 11:**
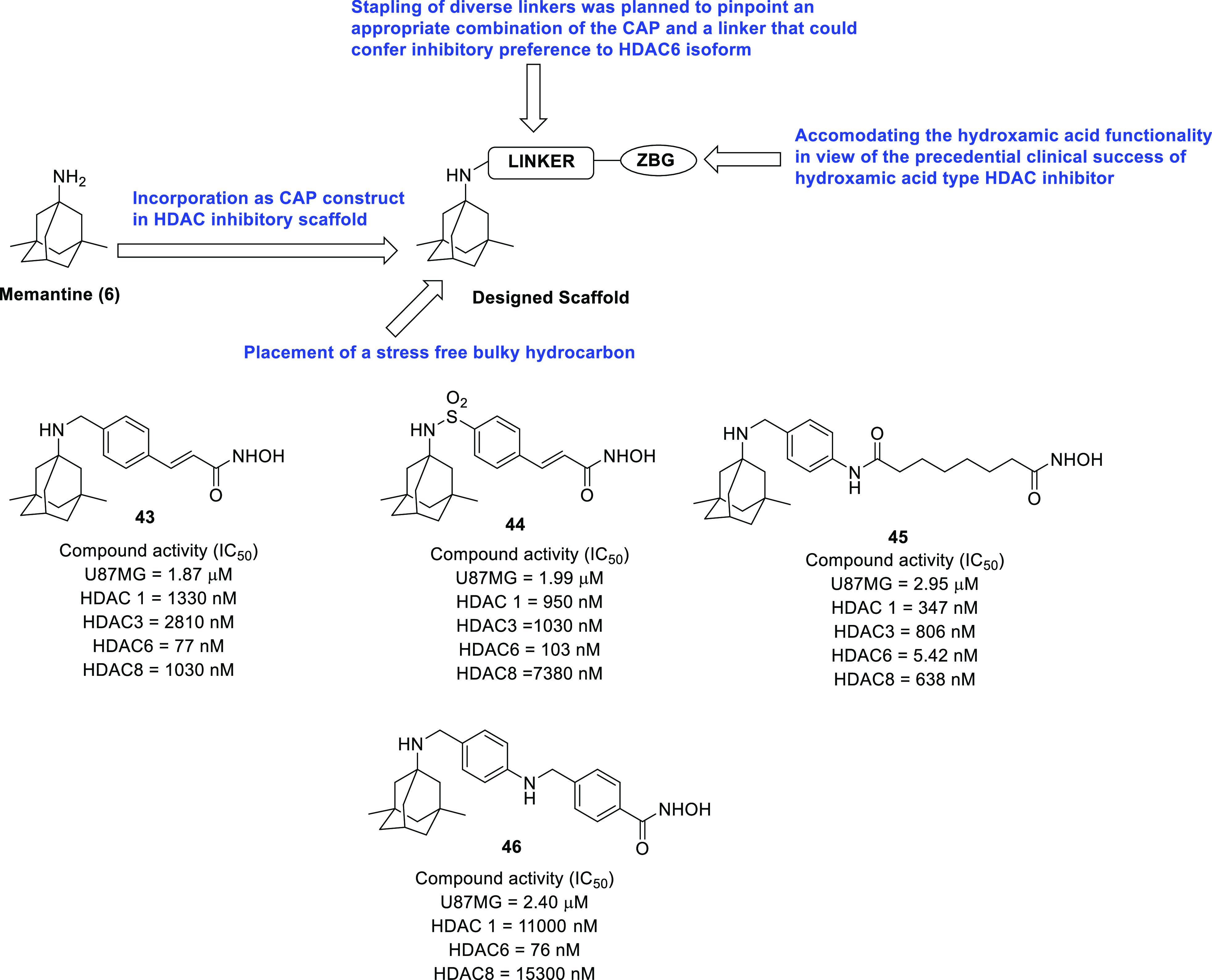
HDAC inhibitors for the treatment of GBM.

Intrigued by the unsuccessful journey of HDAC inhibitors in the context of clinical advancement in GBM, possibly due to a lack of CNS-penetrating ability, Nepali et al. conceived that compensating for the enhanced hydrophilicity conferred by hydroxamic acid functionality via logical installation of CNS drugs (FDA-approved), as the surface recognition part of HDAC inhibitory pharmacophores would be a prudent approach to furnish CNS-penetrating tractable anti-glioma drugs.^54^ The implementation and execution of appropriate actions based on the above-mentioned concept led to the identification of a series of compounds involving the stapling of the memantine core (anti-Alzheimer’s drug, Cap construct) with the zinc binding group via chemically diverse linkers. With this background, Nepali et al. reported some memantine-based HDAC inhibitors as potential anti-GBM agents. All the synthesized compounds were initially evaluated for anti-proliferative activity in the U-87MG glioma cell line. *In vitro* cytotoxicity studies led to the establishment of a structure-cytotoxicity relationship, and several properties were generated that were critical in conferring cell growth inhibitory effects to the designed compounds. The N-benzyl linker used to tether the memantine skeleton with hydroxamic acid functionality was not favorable in terms of inducing anti-glioma effects; however, the incorporation of a vinyl bond and long alkyl chain between the N-benzyl and zinc binding motifs was beneficial, and compounds bearing acrylamide moieties showed promising anti-proliferative effects. Among the synthesized compounds, **43**–**46** exhibited promising anti-proliferative effects (Figure 11). Furthermore, the selected compounds were evaluated for their ability to cause cell cycle arrest using flow cytometry; compounds **43**–**45** caused cycle arrest at G2 phase. Additionally, **45** unregulated the levels of histone H3-K9/K14, histone H3-S10, and α-tubulin caspase-3 and suppressed the (CDK1) cyclin B levels, indicating the apoptosis-promoting ability of the adduct. Compound **45** was also found to be active against TMZ-resistant glioma cells and inhibited the growth of TMZ-resistant U-87MG glioma cells in a dose-dependent manner. To elucidate the mechanism responsible for these striking anti-glioma effects of **45**, all the synthesized compounds were screened against a panel of HDAC isoforms where the compounds displayed moderate inhibitory potential toward HDAC1, HDAC3, and HDAC8 isoforms in the low micromolar range; however, they were substantially selective toward the HDAC6 isoform. Notably, **45** demonstrated a strikingly selective inhibitory potential toward HDAC6, with IC_50_ = 5.42 nM. These results agreed with previous findings of the elevated expression of HDAC6 in GBM. Furthermore, to rationalize the experimental studies using computational studies, docking studies were performed, revealing that **45** displayed good binding affinity with the HDAC6 isoform and interacted with major amino acids W496, H500, H611, F620, and H615 of the enzyme isoforms. The permeability potential of **45** was also evaluated using a parallel artificial membrane permeability assay. This assay rationalized the strategy of incorporating a stress-free bulky hydrocarbon, memantine, as a surface recognition part of the HDAC inhibitory model because **45** demonstrated remarkable CNS-penetrating ability with a permeability value of 33.9. Furthermore, the *in vivo* evaluation results revealed that treatment with hydroxamic acid **45** could prolong the survival of TMZ-resistant U-87MG-inoculated orthotopic mice. In summary, **45** has an impressive anti-GBM profile and warrants further investigation.

**Figure 12:**
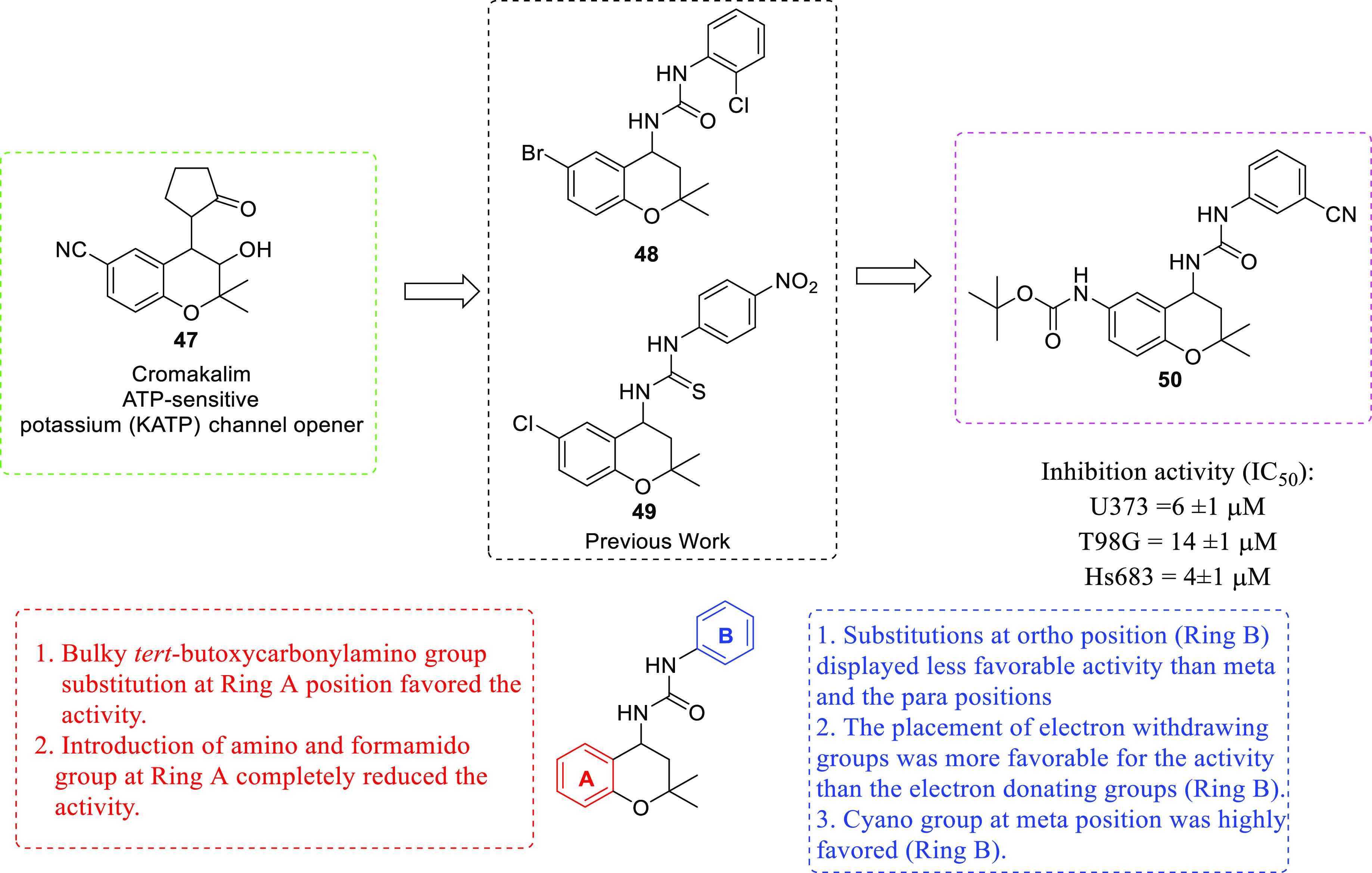
New class III HDAC inhibitors for the treatment of glioma.

In 2017, Schnekenburger et al. identified a new class III HDAC inhibitor, *R*/*S*-*N*-3-cyanophenyl-*N*′-(6-*tert*-butoxycarbonylamino-3,4-dihydro-2,2-dimethyl-2*H*-1-benzopyran-4-yl)urea, as a potent anti-glioma agent.^55^ Previously, the authors disclosed some compounds derived from cromakalim (**47**, an ATP-sensitive potassium (KATP) channel opener) containing an arylurea or arylthiourea moiety at the 4-position (**48**, **49**) that showed anti-glioma activity potential. Given the above, the authors further investigated the amplified benefits in GBM and accordingly synthesized a new series of compounds. The furnished adducts were evaluated against three human high-grade glioma cell lines, U373, T98G, and Hs683. All the synthesized compounds displayed promising activity profiles, and **50** was strikingly potent, with IC_50_ = 6 ± 1, 14 ± 1, and 4 ± 1 μM toward the U373, T98G, and HS683 cell lines, respectively (Figure 12). Furthermore, the therapeutic potential of HDAC SIRT1 and HDAC SIRT-2 in GBM was studied in Hs683 and U373 cells, revealing that SIRT1 was highly expressed in Hs683 cells, whereas SIRT-2 was expressed in U373 cells. Additionally, both SIRTs were knocked down using siRNAs, and cell growth was monitored by video microscopy for 72 h. Considering these findings that siRNA reduction might decrease the cell growth of glioma cell lines, the binding affinities of the synthesized compounds were explored toward the human SIRT1 complex (PDB IDs 4I5I, 4IG9, 4ZZH, 4ZZI, 4ZZJ, and 5BTR) and SIRT2 complex (PDB IDs 4RMG, 4RMH, 1J8F, 3ZGO, 3ZGV, 5DY4, and 5DY5) using Auto Dock Vina followed by an *in vitro* assay. Compound **50** fit well in the binding sites with average dock scores of −9.0 against SIRT-1 and −9.2 against SIRT-2. In the *in vitro* studies, **50** inhibited both SIRT1 and SIRT2, with IC_50_ = 6.2 ± 1.7 and 4.2 ± 1.6 μM, respectively, while no inhibition was observed against HDAC1, 2, 3, 8, 6, 10, and 11 and SIRT-3 activities. Additionally, computer-assisted phase contrast microscopy (quantitative video microscopy) suggested that **50** exerted cytostatic effects rather than cytotoxic effects on both Hs683 and U373 glioma cell lines. Furthermore, **50** induced accumulation in the G1 phase and promoted senescence-associated β-galactosidase (SA-β-gal) activity. The impact of compound **50** on the spheroid-forming capacity of GBM cells was also monitored, which showed that the compound reduced the surface area of tumor spheroids from glioma Hs683 and U373 cell lines. Additionally, **50** abrogated tumor development in the zebrafish xenotransplantation model. Continued evaluation of **50** in the presence of mutated p53 and overexpressed MDR efflux pumps ABCB1 and ABCC1 led the authors to deduce that the aberrant behavior of both did not affect the activity of **50**. Additionally, **50** was tested in the NCI-60 cell line panel, where it displayed a mean GI_50_ value of −5.5 (∼3 μM); however, it did not exhibit effects on peripheral blood mononuclear cells. Overall, compound **50** was found to be a potent inhibitor of class III HDAC that can be used as a lead in the development of potent anti-GBM agents.

**Figure 13:**
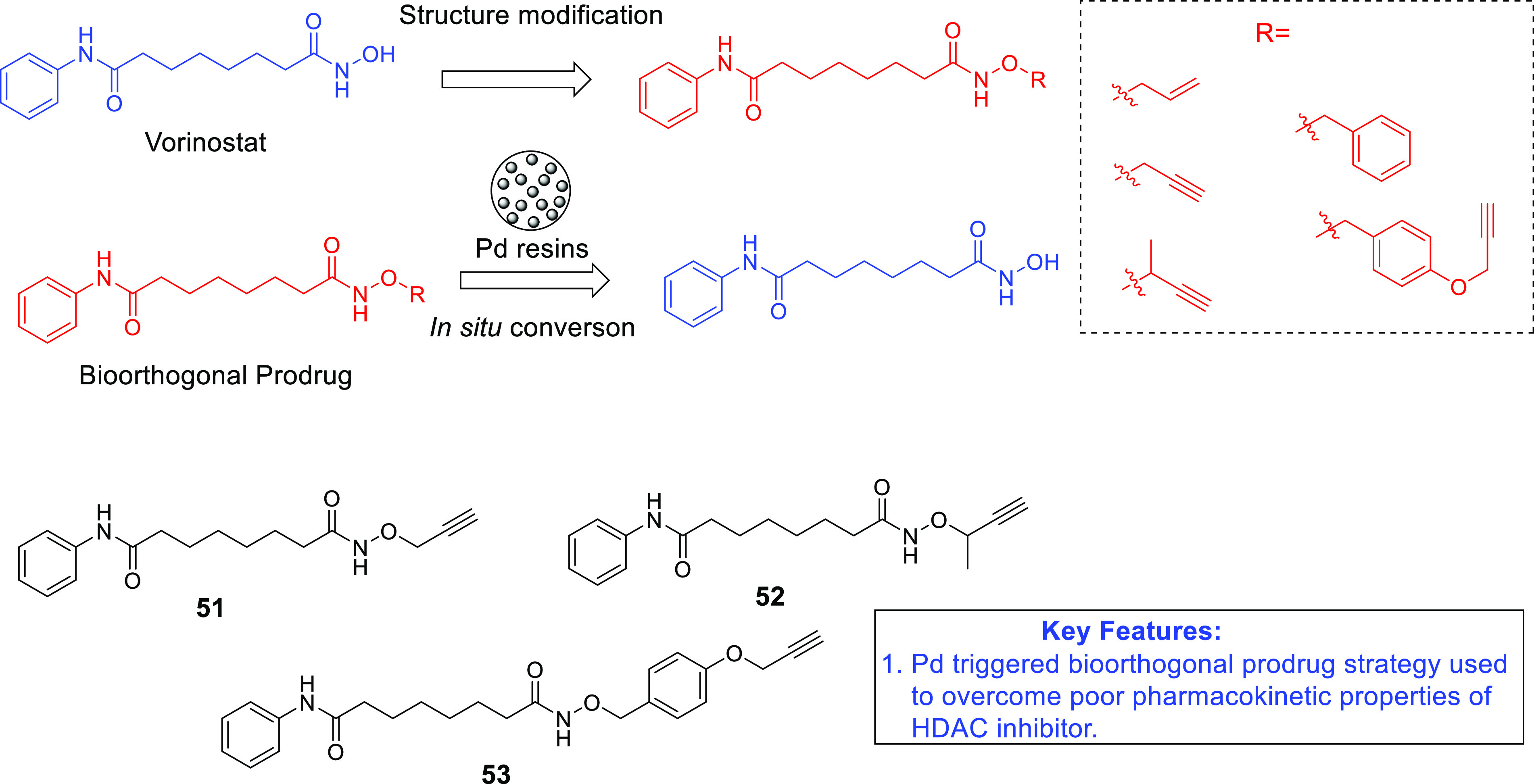
Bioorthogonal uncaging to enhance the pharmacokinetic properties of HDAC inhibitor.

In 2016, Rubio-Ruiz et al. reported an efficient method for the release of vorinostat (SAHA, an FDA-approved hydroxamic acid-type HDAC inhibitor), triggered by palladium-functionalized resins, to enhance its PK properties by modulating the metal chelating effect of hydroxamic groups.^56^ The hydroxamic group of vorinostat binds to the catalytic site of the HDAC enzyme and forms a chelate complex with Zn^2+^ metal. Based on the revealed binding modes, O-alkylated derivatives of vorinostat were synthesized, and their chelating capacity was evaluated (structures shown in Figure 13). The O-alkylated derivatives did not interact with the iron metal, while a color change was observed in the solution containing a mixture of vorinostat and iron. These observations indicated that the compounds completely lost their metal chelating activity following alkylation of the OH group. The same inactivation of compounds was replicated in bioorthogonality studies employing U-87G glioma cells; the O-alkylated derivatives displayed mild cytotoxicity compared with vorinostat. After initial investigations, the Pd-mediated release of synthesized derivatives was observed with FeCl_3_. During analysis, a color change was observed following treatment of the compounds with FeCl_3_, indicating that Pd activated the metal chelating effect. Furthermore, the effect of compounds with Pd was evaluated in U-87G cell lines. Notably, **53** was only activated in the presence of Pd resins, converted to its parent form (vorinostat), and displayed potential anti-proliferative activity. The outcome of the study led to the identification of an effective strategy to overcome the poor pharmacokinetics of HDAC inhibitors using an approach of uncaging an inactive precursor of vorinostat by heterogeneous Pd catalysis in glioma cells.

**Figure 14:**
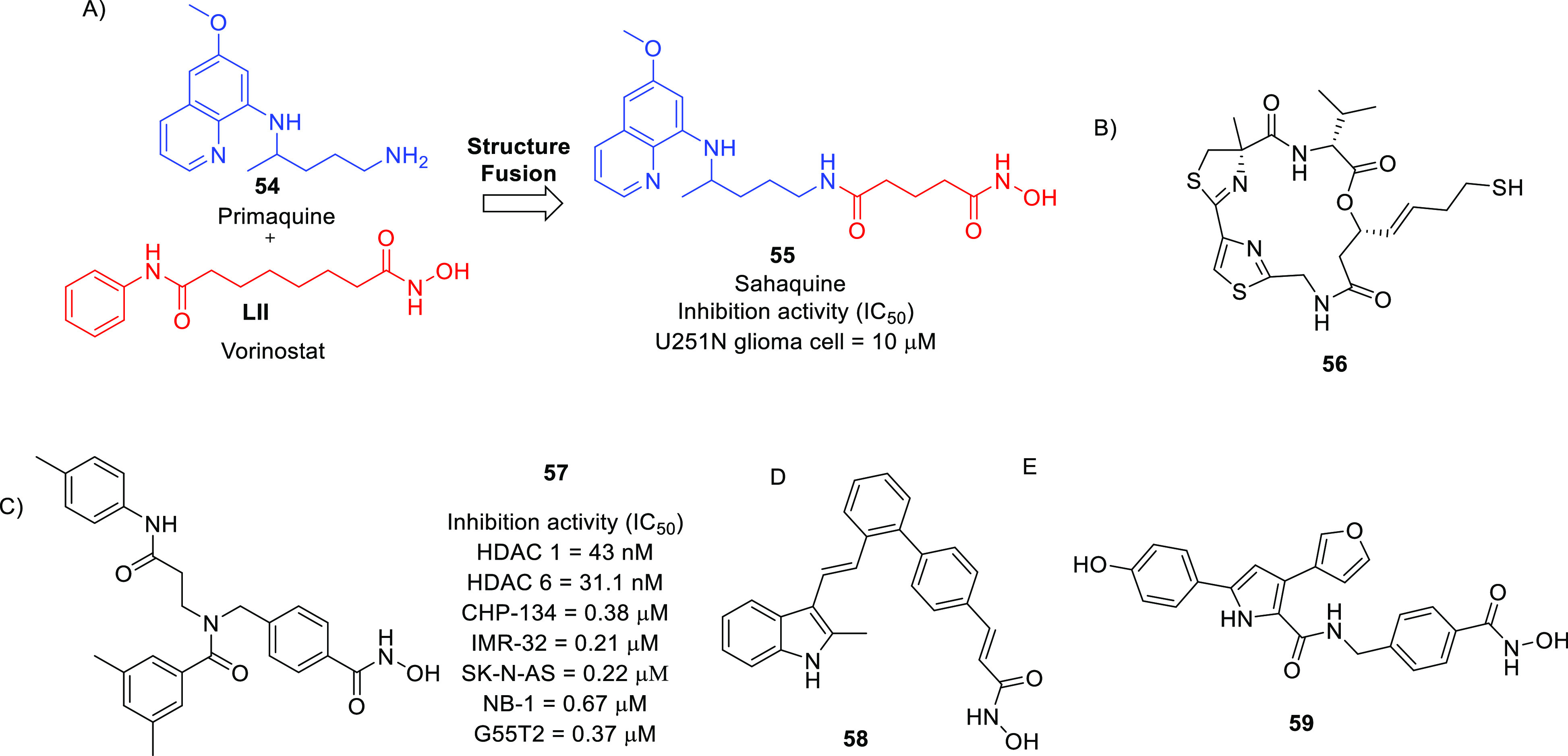
(A) Sahaquine, (B) largazole, (C) peptoid-based histone deacetylase inhibitor, (D) 4-vinylbiphenyl skeleton as histone deacetylase inhibitor, and (E) JOC 1 as potential HDAC inhibitor.

Inspired by the success of hybrid scaffolds as anti-cancer drugs, Zhang et al. designed a novel hybrid of primaquine (anti-malarial drug) (**54**) with vorinostat (LII) as a selective HDAC6 inhibitor.^57^ The design strategy to construct the hybrid was based on the anticipation that the inclusion of a core fragment of primaquine will confer the ability to the resulting adduct to interfere with endosomal trafficking to the plasma membrane and inhibit the multi-drug-resistance transporter P-glycoprotein and autophagy. With the above-mentioned design rationale, the compound was synthesized by fusing the active pharmacophore of varinostat with primaquine and was evaluated in the U251N glioma cell line. The evaluation results revealed that **55** inhibited the U251N cell line with IC_50_ = 10 μM. Enlightened by its promising cell growth inhibitory effects, **55** was evaluated for a cell invasion assay, and **55** did not inhibit cell migration. However, a combination of **55** with quercetin efficiently inhibited cell migration by 42%. Additionally, the hybrid of primaquine (**54**) and sahaquine (**55**) inhibited p-glycoprotein activity at a 10 μM dose. Continued evaluations revealed that **55** selectively inhibited the HDAC-6 isoform and reduced the levels of EGFR, ERK1/2, and Akt alone as well as in combination with quercetin (Figure 14).

Al-Awadhi et al. investigated largazole (**56**) (Figure 14B) to ascertain its potential as a brain-penetrant class I HDAC inhibitor prodrug.^58^ Largazole demonstrated *in vitro* anti-GBM efficacy coupled with BBB-penetrating ability, as evidenced by studies based on measuring the active species (concentration), largazole thiol, in the mouse brain. Additionally, treatment with largazole led to Pax6 upregulation, which suppressed GBM proliferation. Collectively, the results highlight the need for a comprehensive evaluation of largazole in GBM.

Reßing et al. executed a medicinal chemistry campaign to rationally design a novel class of peptoid-based histone deacetylase inhibitors (HDACi).^59^ Eleven peptide-based HDACi were synthesized and screened over CHP-134, IMR-32, SK-N-AS, and NB-1 (neuroblastoma) and G55T2 (glioblastoma) cell lines, where **57** was found to be most potent in the series, with the IC_50_ values shown in Figure 14C. Additionally, the selectivity profile of all compounds was studied for HDAC1 and HDAC6, where **57** was found to be non-selective against HDAC1 and HDAC6. To identify a new scaffold with potential anti-GBM activity against resistant cell lines, Ellert-Miklaszewska et al. screened three diverse groups of scaffolds with 4-vinylbiphenyl skeleton, 3-arylidene-oxindole, and isothiazolonaphthoquinone core substitutions.^60^ The identification of the scaffold was carried out by screening the compounds from the various series over LN18 and T98 GBM cell lines. Among all the compounds, **58**, with a HDAC inhibitor architecture, showed significant cell growth inhibition of more than 70% against the LN18 and T98 cell lines. In addition, it was revealed that **58** potentially inhibited all forms of HDAC with the prudential inhibition of HDAC6 and 8. Overall, these findings suggest that the compounds with HDAC frameworks can serve as potential inhibitors against resistant GBM cell lines (Figure 14D).

In addition to scaffold assembly studies, some efforts have also been directed toward the determination of HDAC6 expression in GBM. Auzmendi-Iriarte et al. conducted HDAC6 expression analysis in GBM using the Rembrandt cohort (28 control and 219 GBM samples), TCGA cohort (4 control and 156 GBM samples), Gravendol cohort (8 control samples and 24 grade II, 85 grade III, and 159 grade IV glioma samples), vital cohort, and Donson cohort.^61^ The results revealed that GBM samples contained high expression of HDAC6, and HDAC6 overexpression correlated with advanced glioma grade and poor patient survival. HDAC6 was also enriched in glioma stem cells, and its expression positively correlated with several GSC markers (SOX2, SOX9, CD133, NESTIN, and OCT4). In addition to the above-mentioned findings, the study also identified JOC 1 (**59**) (Figure 14E) as a small-molecule inhibitor of HDAC6 with GBM cell growth inhibitory potential *in vitro* and *in vivo*. **59** was more effective against the proliferation and self-renewal capacity of a subpopulation of GSCs in single and combined therapy with TMZ. At the molecular level, **59** significantly reduced the expression of the SOX2, SOX9, and BM1 genes (key regulators of a subpopulation of glioma stem cells). In the transcriptomic analysis, **59** decreased the cell cycle pathways and elevated neural differentiation and cell death in glioma stem cells.

### 4.3 Isocitrate Dehydrogenases (IDH) Inhibitors

**Figure 15:**
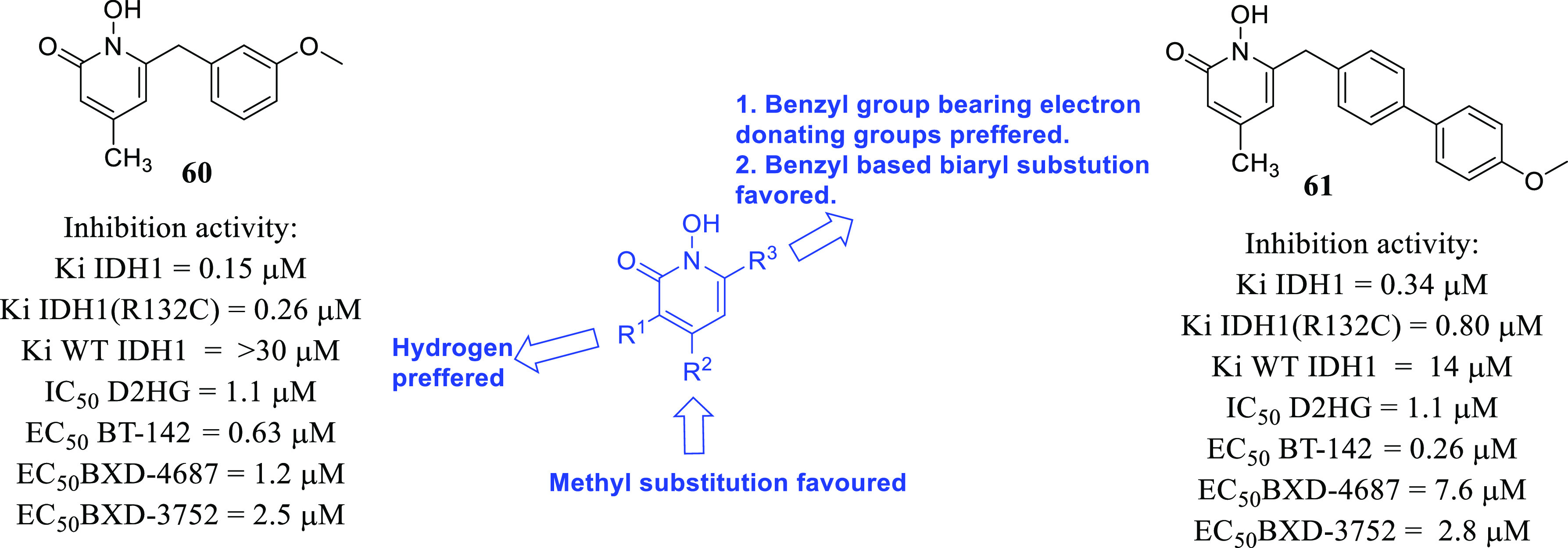
IDH inhibitors.

IDH is an essential enzyme in the tricarboxylic acid cycle that converts isocitric acid (ICT) to α-ketoglutaric acid (α-KG) using Mg^2+^ and NADP^+^ (or NAD^+^) as cofactors. Reports have suggested that because of mutations, IDH converts α-ketoglutaric acid to d-2-hydroxyglutaric acid, which is an unfavorable factor of cancer initiation in glioma. Additionally, it has been found that IDH1 inactivation causes reduced biosynthesis of deoxynucleotide and lipid and enhanced congregation of ROS, which decreases the growth of GBM through RNA obstruction. Moreover, IDH inactivation promotes the sensitivity of tumor cell to both senescence induced by radiation and erlotinib, increasing the survival of mice with xenografts derived from patient.^521,522^ In addition, a decrease in cell growth in orthotopic GBM mouse models was observed after genetic inhibition of IDH3α due to enhanced total NADPH/NADP^+^ ratio, metabolites of pyrimidine pathway, nucleotide biosynthesis, and epigenetic alterations (DNA methylation) of potent growth factors in highly proliferative GBM cells, generating metabolic vulnerability.^523^ To exploit these revelations, Liu et al. designed and synthesized a series of 61 compounds as IDH1 inhibitors.^62^ All the synthesized compounds displayed excellent inhibition within the nanomolar to micromolar range. Among the series, **60** and **61** displayed functional IDH1 inhibition with *K*_i_ = 0.15 and 0.34 μM, respectively. The SAR was investigated, and the properties generated are presented in Figure 15. Based on the SAR results, 10 compounds were evaluated against IDH1 (R132C) and WT IDH1, where all compounds showed inhibition (*K*_i_) in the range of 0.14–9.5 μM. The best compounds, **60** and **61**, inhibited IDH1 (R132C) and WT IDH1 with *K*_i_ = 0.26 and >30 μM and 0.80 and 14 μM, respectively. Furthermore, the impact of the synthesized compounds on D2HG concentrations was evaluated in HT1080 human fibrosarcoma cells, revealing that **60** and **61** inhibited D2HG production, with IC_50_ = 1.1 μM. As part of the continued investigation, a blood–brain permeability study was performed in MDCK-MDR1 cells. **60** penetrated the experimental BBB wall with permeability values of 5.39 (apical to basolateral) and 8.88 (basolateral to apical), and the efflux ratio was 1.7. Additionally, the activity of the selected inhibitor was evaluated against the glioma cell lines BT-142 BXD-4687 and BXD-3752, where **60** and **61** inhibited cell growth with EC_50_ = 0.63, 1.2, and 2.5 μM and 0.26, 7.6, and 2.8 μM, respectively. No growth inhibition was observed in normal fibroblast WI-38 cells, suggesting that the compounds selectively inhibited cell growth in glioma.

**Figure 16:**
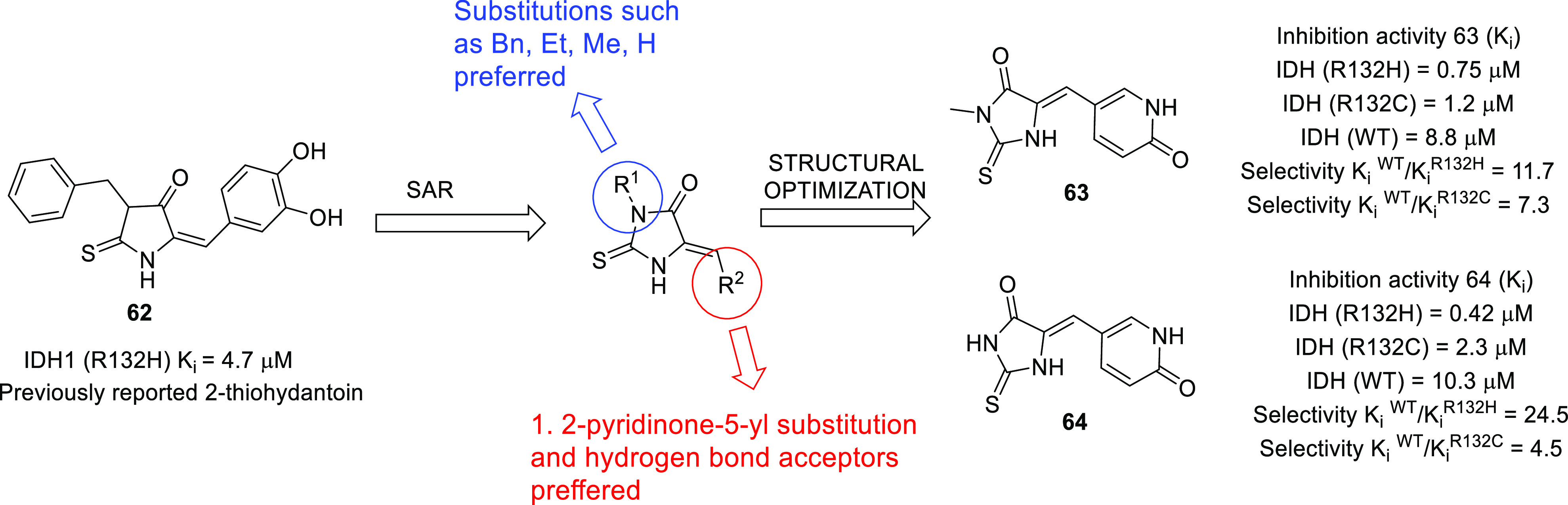
2-Thiohydantoin derivative as IDH inhibitors.

In 2015, Wu et al. published a SAR study of a potent 2-thiohydantoin derivative (**62**) as a cancer-associated mutant IDH-1 inhibitor.^63^ A total of 37 compounds were furnished with various substitutions, and their activity was evaluated against IDH1 (R132H). Among the synthesized series, **63** and **64** were the most potent, with *K*_i_ values shown in Figure 16. The study of the binding pattern of both compounds showed that both compounds exhibited similar binding patterns and were surrounded by pocket residues Thr77, Ser94, Asn96, Gly97, Arg100, Asn101, Arg109, and NADPH via hydrogen bonding. Furthermore, an enzyme kinetic study of the most potent **64** for α-KG and NADPH was performed and expressed as a Lineweaver–Michaelis–Burk or Menten plot, where **64** was found to be competitive with α-KG and non-competitive with NADPH. Moreover, **63** and **64** inhibited BT142 glioma cells bearing IDH1 R132H mutations.

**Figure 17:**
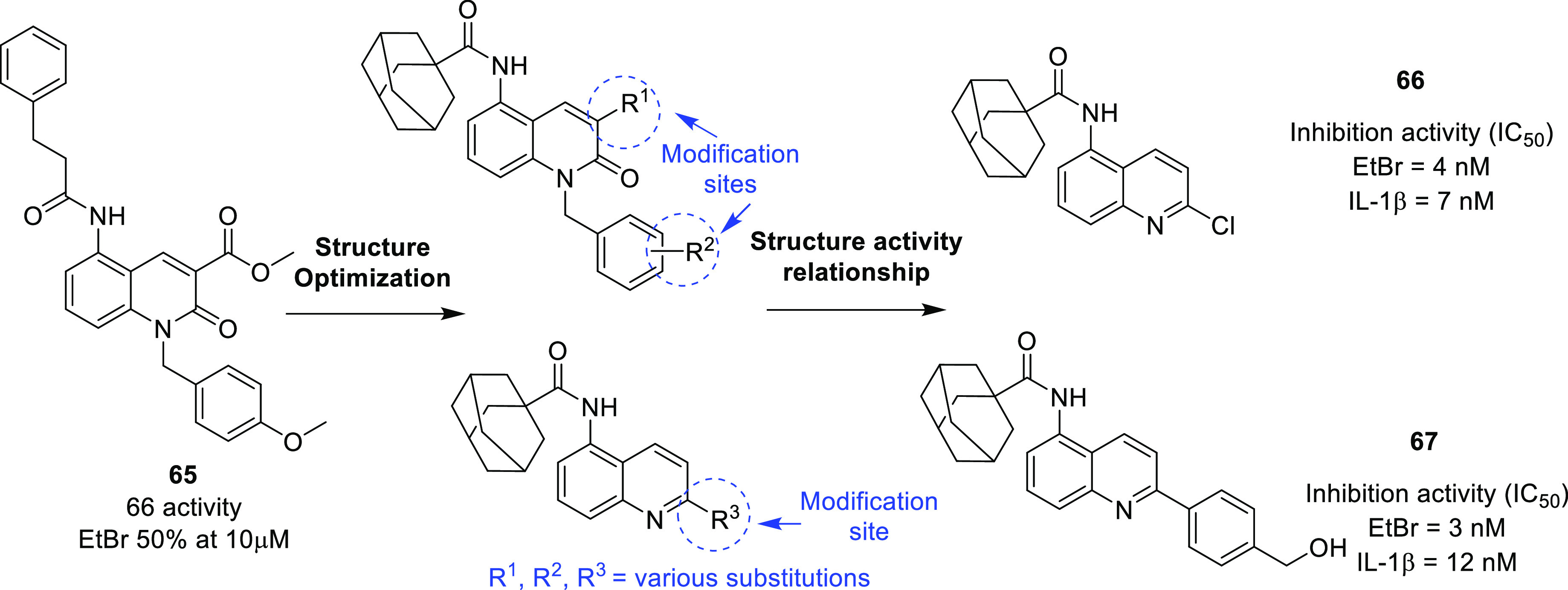
Quinolinone-based P2X7 receptor antagonist as anti-GBM agents.

In 2018, Kwak et al. published the structural modification of the previously reported quinolinone-based compound **65** as a putative P2X7 receptor antagonist.^524^ Structural engineering was executed, which led to a series of quinolinone- and quinolone-based P2X7 receptor antagonists. Among the synthesized compounds, quinolone-based compounds **66** and **67** were found to be most potent in the series, with IC_50_ = 4 and 3 nM against EtBr uptake in hP2X7-expressing HEK293 cells, respectively. Additionally, the functional activity of compounds **66** and **67** based on P2X7R-related signaling in immune cells was studied, and the activity is shown in Figure 17. Furthermore, **66** displayed a potential impact on the growth of TS15-88 GBM cells, where it reduced the sphere size of TS15-88 GBM cells. Overall, quinoline-based derivatives can serve as potential anti-GBM agents.

**Figure 18:**
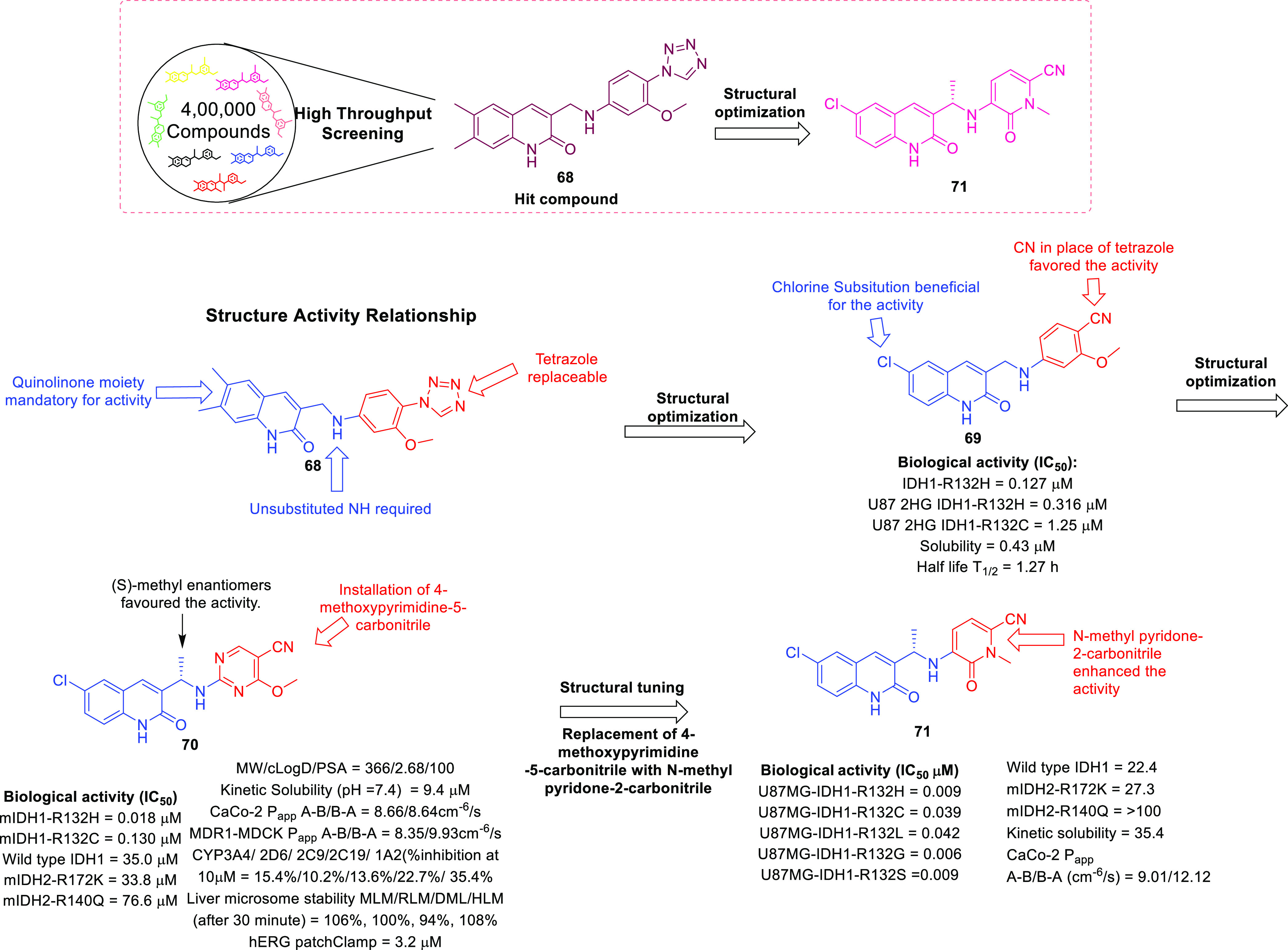
Structure optimization of potent IDH1 inhibitor FT-2102 (Olutasidenib).

In 2019, Lin et al. revealed the discovery of quinolinone derivatives as selective mutant IDH-1 inhibitors endowed with anti-cancer potential.^64^ Initially, 400 000 compounds were screened through high-throughput screening (HTS) using the diaphorase assay. Among the screened compounds, quinolinone was the most suitable scaffold, and **68** was considered a hit compound for mIDH1 activity. Efforts were made to achieve potency, and a SAR was generated for hit **68** (shown in Figure 18). Various substitutions were attempted for the left and right sides of the structure, and compounds were screened against IDH1-R132H. Among the series, **69** was the most promising inhibitor, with IC_50_ = 0.127 μM. Along with the preliminary study, the compound was screened for various IDH1 isoforms using both biochemical and cellular assays, where it showed efficient results with activity in the low micromolar range. In the computational study, **69** bound to the allosteric site of the binding pocket and interacted with the major amino acids Leu120, Ile130, Ile128, Trp267, and Ala258 of the binding pocket. Further explorations (*in vitro* and *in vivo* studies) displayed moderate PK results for **69**, and the results were as follows: MLM left = 42% in 30 min, solubility = 0.43 μM, half-life (*T*_1/2_) = 1.27 h, *C*_max_ = 1.06 μM, and AUC_0–8h_ = 2.45 μM. Thus, the results of the PK studies presented a scope of improvement of the PK properties of **69**. Subsequently, another structure-based guided optimization was performed for **69**, leading to the identification of **70** with striking IDH1 inhibitory activity, as demonstrated by both biochemical and cellular assays. The PK/PD properties of **70** were studied, including the ADME and safety profiles, and the compound showed excellent liver microsomal and plasma stability. **70** displayed low activity for P450 (CYP450) enzymes and exhibited a safety window of 200-fold (shown in Figure 18). Although inhibitor **70** displayed promising results, low solubility and insufficient mouse PK exposure were some of the limitations. Thus, the group continued their structure-based drug design program and reported the discovery of FT-2102 (**71**), also named olutasidenib, as a potent IDH-1 inhibitor. Overall, **71** displayed substantial inhibitory potential against IDH-1 R132H along with good HLM-MLM stability and an improved solubility profile. In biochemical and cellular assays, the compounds showed balanced activity profiles, as shown in Figure 18. Additionally, the BBB penetration ability of **71** was evaluated in male CD-1 mice at oral doses of 5 and 100 mg/kg, where the blood/plasma ratios were 0.24 and 0.38, respectively. In the artificial membrane PAMPA (19.9 × 10^–6^ cm/s mean) and Caco-2 cell system [P_app_ (B/A) (A/B) 9/12), **71** displayed impressive results, with an efflux ratio of 1.35. Owing to an extremely impressive activity profile, **71** (FT-2102) is currently being evaluated in clinical studies for CNS tumors (NCT03684811).^65^

### 4.4 Translocator Protein (TSPO) Inhibitors

The TSPO is a specific marker located in the outer mitochondrial membrane and is used to envisage lesions in brain injury/disease. In GBM, the expression of TSPO has been found to be increased, suggesting its role in the progression and initiation of tumors. Along with other proteins present in mitochondria, such as ATPase, adenine nucleotide transporter (ANT), and voltage-dependent anion channel (VDAC), TSPO regulates the release of Ca^2+^ from mitochondria and the production of ROS and ATP by opening the mitochondrial permeability pore (mPTP), leading to ΔΨm collapse (mitochondrial membrane potential). Subsequent depolarization accelerates the opening of BAK/BAX channels for cytochrome *c* passage into the cytosol and triggers the cascade of apoptosis in mitochondria. In GBM cells, proliferation and invasion are increased with a rise in ATP.^302^ In extensive clinical studies of the heterogeneity of the tumor, diverse morphological adjustments, and interactions with the microenvironment, TSPO can be utilized as a prudent target against GBM.

**Figure 19:**
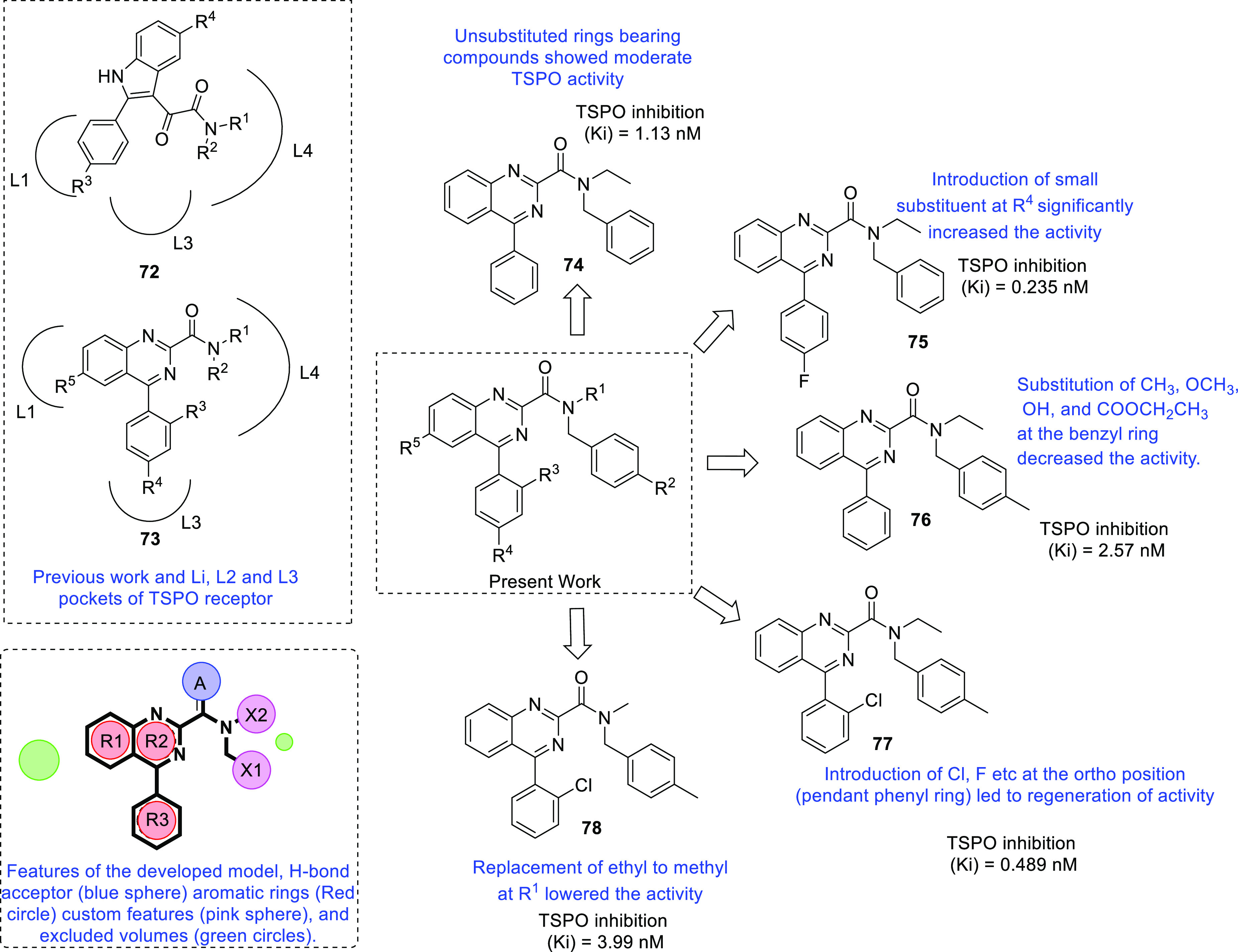
SAR studies of 4-phenylquinazoline-2-carboxamides for TSPO activity.

In 2014, a team led by Martini et al. performed SAR studies on a novel TSPO ligand, 4-phenylquinazoline-2-carboxamide.^66^ Previously, two different sets of compounds (**72**, **73**) were reported by the group to show notable activity against TSPO with *K*_i_ values in the nanomolar/sub-nanomolar range. The pharmacophore/topological model of both series comprised three lipophilic pockets (L1, L3, and L4) and an H-bond donor group (H1). The benzyl moiety served as a common feature in high-affinity TSPO ligands, as shown in Figure 19. Considering these findings, a series of *N*-benzyl-substituted 4-phenylquinazoline-2-carboxamides was designed by varying the number of carbon atoms between 4 and 6 on the carboxamide nitrogen and substituting the C4 position of the phenyl ring with various substituents, such as CH_3_, OCH_3_, OH, COOCH_2_CH_3_, and COOH, to reach the L4 lipophilic pocket of the TSPO binding site. SAR analysis illustrated that unsubstituted **74** demonstrated magnificent TSPO inhibitory activity at low nanomolar concentrations with *K*_i_ = 1.13 nM. Afterward, substitutions at the pendant 4-phenyl ring of lead **74** were made, and the resulting compounds exhibited TSPO inhibitory profiles with *K*_i_ = 0.235–1.68 nM. Substitution of fluorine at the 4′-position of the pendant 4-phenyl ring was the most effective because it produced the best compound of the series, **75** (*K*_i_ = 0.235 nM). Furthermore, the L4 pocket of the receptor was explored by placing diverse substituents at the benzyl moiety (CH_3_, OCH_3_, OH, NO_2_, COOCH_2_CH_3_, and COOH), and no significant enhancement in the activity profile was observed. Additionally, the 3D pharmacophore model of synthesized compounds was constructed using the PHASE suite of the Maestro package of Schroedinger to ascertain the pharmacophoric features, as shown in Figure 19. Furthermore, the anti-proliferative activity of the synthesized compounds was investigated in U343 GBM cells, and compound **77** was the most potent, reducing the cell viability up to 40%. In further studies, **77** dissipated the mitochondrial membrane and inhibited glioma cells through an intracellular pro-apoptotic mechanism induced by TSPO. In conclusion, structural optimization led to potent TSPO inhibitors that may be used as a lead for future investigations.

**Figure 20:**
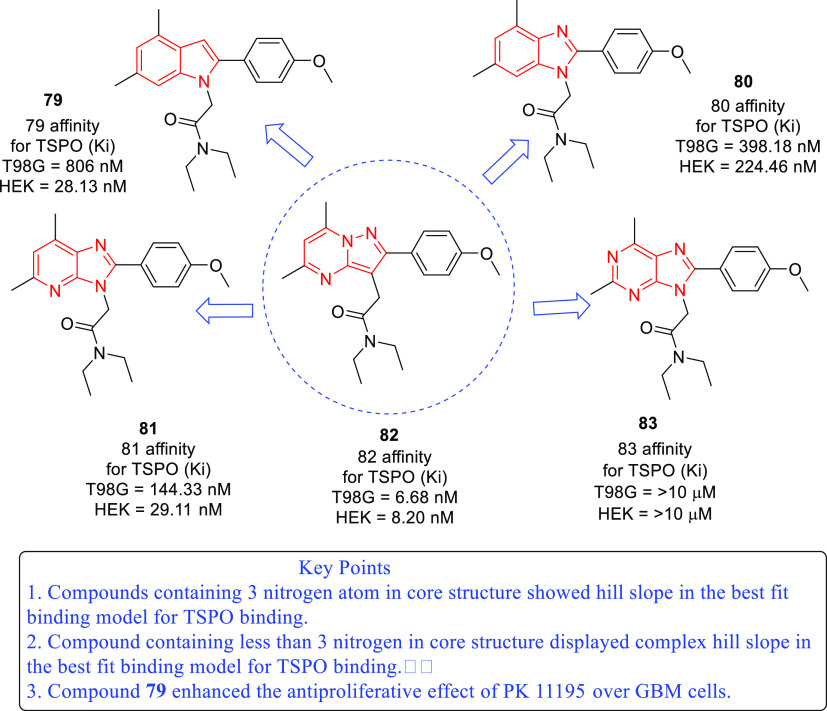
Wild-type TSPO ligand.

Pyrazolo[1,5-*a*]pyrimidine (**79**) is considered a privileged structure endowed with high affinity toward TSPO. To exploit this finding, Narlawar et al. designed pyrazolo[1,5-*a*]pyrimidine analogs and explored scaffolds for allosteric-like modulation of human wild-type TSPO.^67^ A total of five compounds with various cores were synthesized, and their binding interactions were investigated by competing with the radioligand [^3^H]PK 11195 in HEK 293T and T98G cell membranes. The results are presented as Hill slopes. A Hill slope of 1 represents the on-site interactions of the compound, while a Hill slope of −1 and shallower than −1 shows a negative modulation where the compound is predicted to bind at another site. The compounds with nitrogen displayed a Hill slope of 1 that depicted the one-site interaction of compounds at the same site of the radioligand. All the tested compounds (**79–83**) showed activity (*K*_i_) in the low nanomolar range except **83**, which did not demonstrate binding to TSPO (Figure 20). In the T98G GBM cell line, **79** did not demonstrate anti-proliferative activity; however, it enhanced the activity of PK11195 at concentrations of ∼10 and ∼62.5 μM. Overall, the allosteric behavior of TSPO and binding of various heterogeneous compounds may be helpful to design new TSPO ligands.

**Figure 21:**
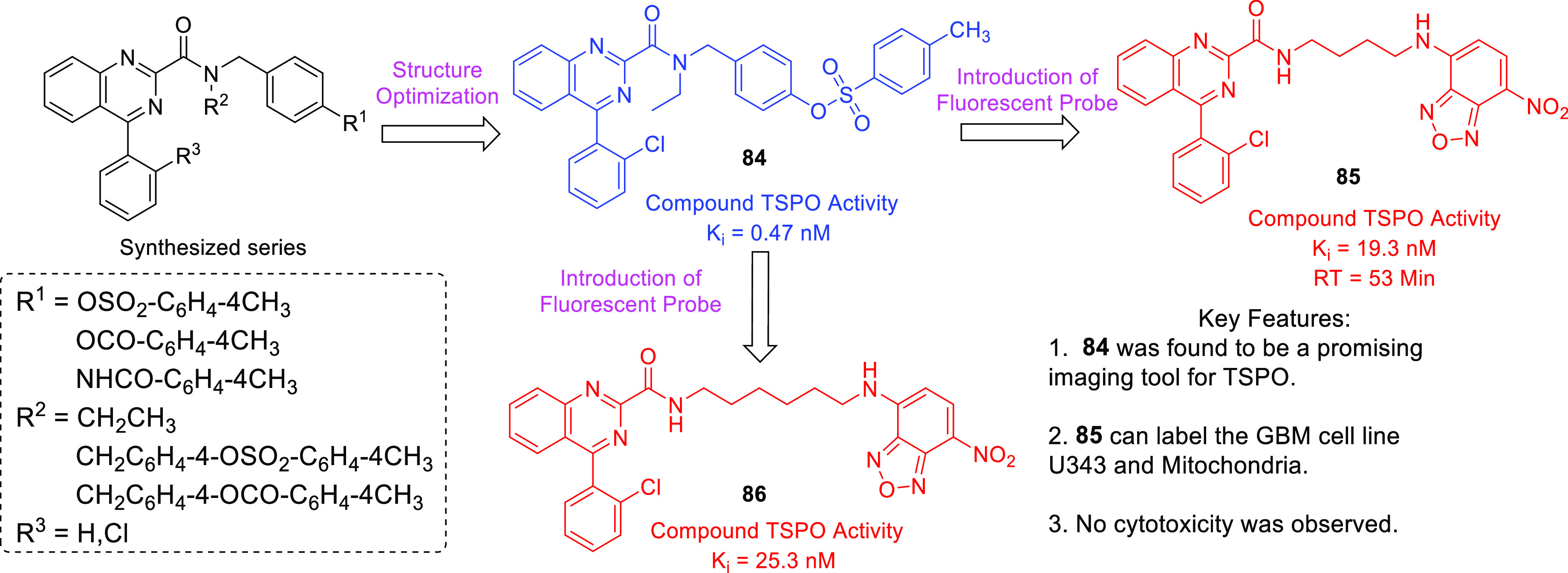
4-Phenylquinazoline-based TSPO ligands.

In 2017, Milite et al. revealed phenylquinazoline-mediated high-affinity fluorescent probes to detect TSPO.^68^ For initial studies, a two-dimensional (2D) pharmacophore/topological model was used to design probes suggesting 4-phenylquinazoline as a promising pharmacophore of TSPO. Additionally, an in-house library of quinazoline was used, and a 3D model of interaction with TSPO was studied. The encouraging results prompted the authors to synthesize the series of compounds that were evaluated for the TSPO binding assay, and the compounds demonstrated activity in the nanomolar range. Among the series, **84** was the most potent, with *K*_i_ = 0.47 nM. **84** was selected for further modifications, and fluorescent probes **85** and **86** were synthesized by inserting 7-nitro-2,1,3-benzoxadiazolyl (NBD) with tetramethylene and hexamethylene as spacers, respectively. The compounds were further evaluated for TSPO binding affinity, where **85** showed effective results with *K*_i_ = 19.2 nM and a retention time (*t*_R_) of 53 min. The fluorescent labeling of **85** and **86** was evaluated in the human GBM cell Line U343, in which both compounds showed uniform cytoplasmic labeling. Additionally, mitochondrial labeling was investigated using MitoTracker Red, and **85** labeled TSPO at the mitochondrial level. Additionally, **85** completely displaced the non-fluorescent ligand PK11195 at a concentration of 50 μM without cytotoxicity (shown in Figure 21). In conclusion, **85** showed promising TSPO and mitochondria labeling and could serve as an effective imaging biomarker for TSPO.

**Figure 22:**
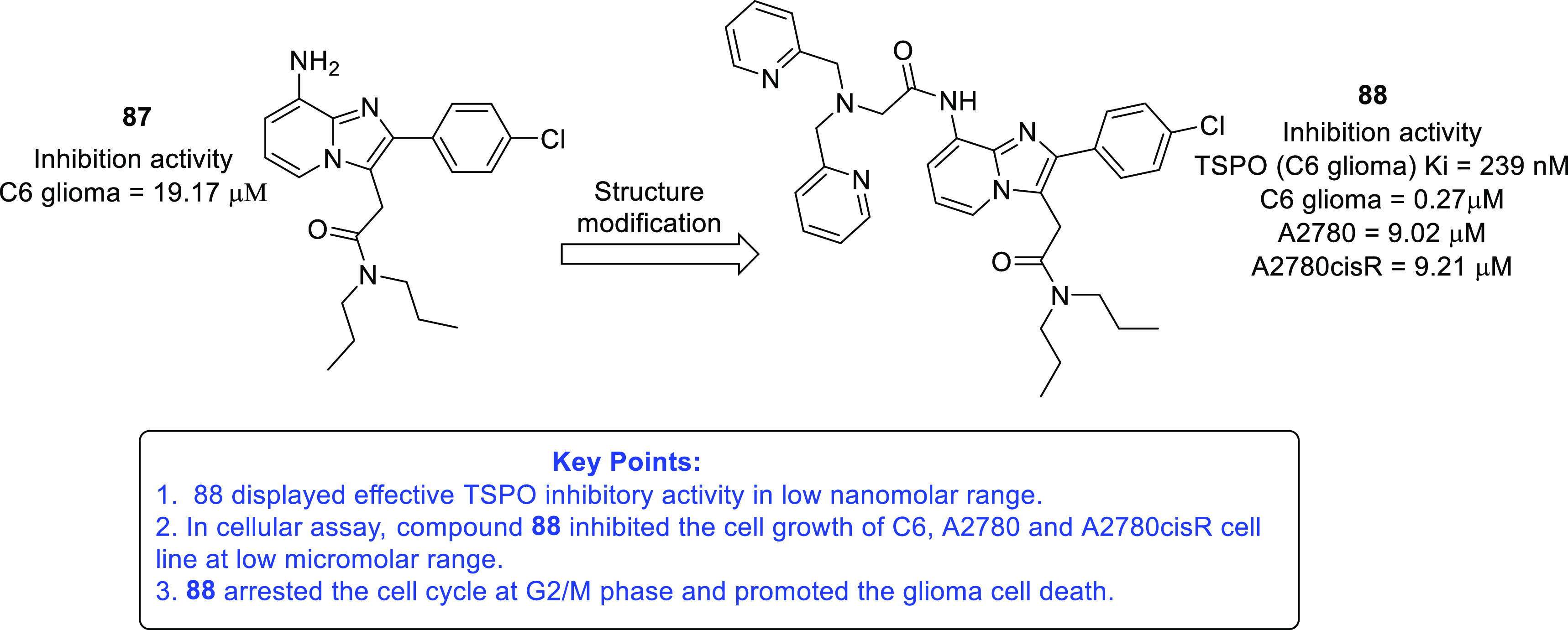
TSPO ligand as potential anti-cancer agent against glioma.

In 2014, Denora et al. published a report on a new selective bifunctional chelating ligand of TSPO with potential activity against glioma cell lines.^69^ The structural template of a previously reported **87** was used to design ligand **88**, and a multi-step synthetic protocol was employed for the synthesis. The results of the biological evaluation revealed that **88** was endowed with an excellent affinity toward TSPO with *K*_i_ = 239 nM. Additionally, **88** was analyzed against C6, A2780, and A2780cisR glioma cell lines, where the compound elicited inhibition at low micromolar ranges, with IC_50_ = 0.27, 9.02, and 9.21 μM, respectively. On further analysis, the loading of **88** with biometals such as Cu induced double-strand DNA cleavage and caused cell death by targeting the mitochondria of the cells. Additionally, **88** enhanced the number of mitochondria-depolarized cells, suggesting the involvement of apoptosis in cell death. Flow cytometry analysis confirmed that **88** arrested the cell cycle at the G2/M phase and promoted cell death (Figure 22).

**Figure 23:**
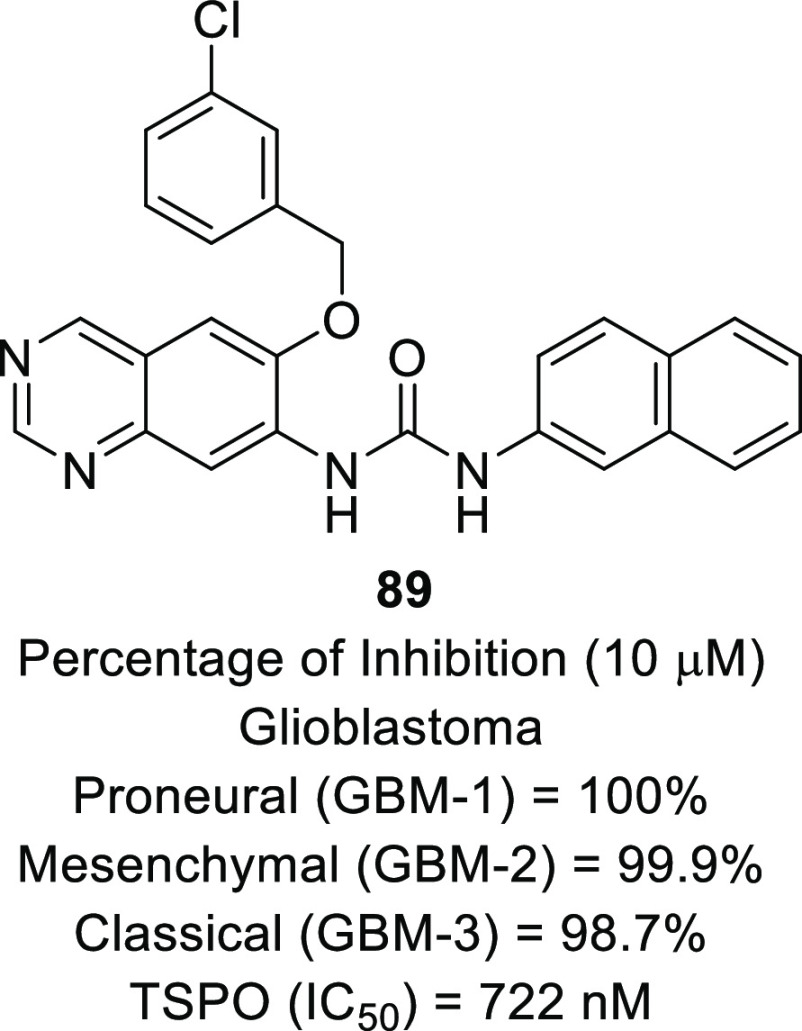
Quinazoline-urea-based TSPO inhibitor against GBM.

In 2015, Elkamhawy et al. reported a series of quinazoline-urea-based compounds that demonstrated significant cell growth inhibitory potential toward proneural (GBM-1), mesenchymal (GBM-2), and classical (GBM-3) GBM.^70^**89** was identified as the most potent TSPO inhibitor that displayed substantial efficacy against the TMZ-resistant glioma cell line and also showed an acceptable toxicity profile (Figure 23).

### 4.5 Protein Disulfide Isomerase (PDI) Inhibitors

The overexpression of PDI6 is involved in the progression, invasion, and migration of GBM cells.^525^ The relationship between clinical–pathological outcomes of GBM and mRNA expression of PDIs studied in the CGGA and TCGA database analysis indicated that the somatic alterations in the GBM (high PDI signature and risk score) are involved in the aberrations of driver oncogenes PIK3CA, MUC16, and TTN and amplification peaks of oncogenes (PDGFRA, PIK3C2B, CDK4, and EGFR). The additional involvement in the deletion peaks of tumor suppressor genes (CDKN2A, TUSC1, PTEN, CDKN2B, BNIP3, and FAS) suggests the importance of PDI in the malignant processes of GBM, including endoplasmic reticulum (ER)-associated degradation, unfolded protein response, cell adhesion, endoplasmic reticulum stress (ERS), WNT signaling pathways, DNA sensing (cytosolic), and apoptosis. In addition, PDIs interact with numerous signaling pathways, such as endoplasmic reticulum-associated protein degradation (ERAD), ERS, and unfolded proteins (UPR).^526^ Moreover, PDIs regulate the activation step of integrins (SH to S-S conversion) and ERS by inhibiting misfolded protein accumulation as well as activate anti-apoptotic caspase-3 and -7, which are responsible for the invasion and migration of glioma cells, their resistance to TMZ, and apoptosis inhibition.^527−530^ In addition to tumor progression and survival, PDIs regulate the immune response in gastric cancer patients by involving PDIA3 complexation with MCH class I through NKG2D ligands.^531,532^ The exhaustive engagement of PDI in tumor development and survival makes PDI a prudent target against GBM.

**Figure 24:**
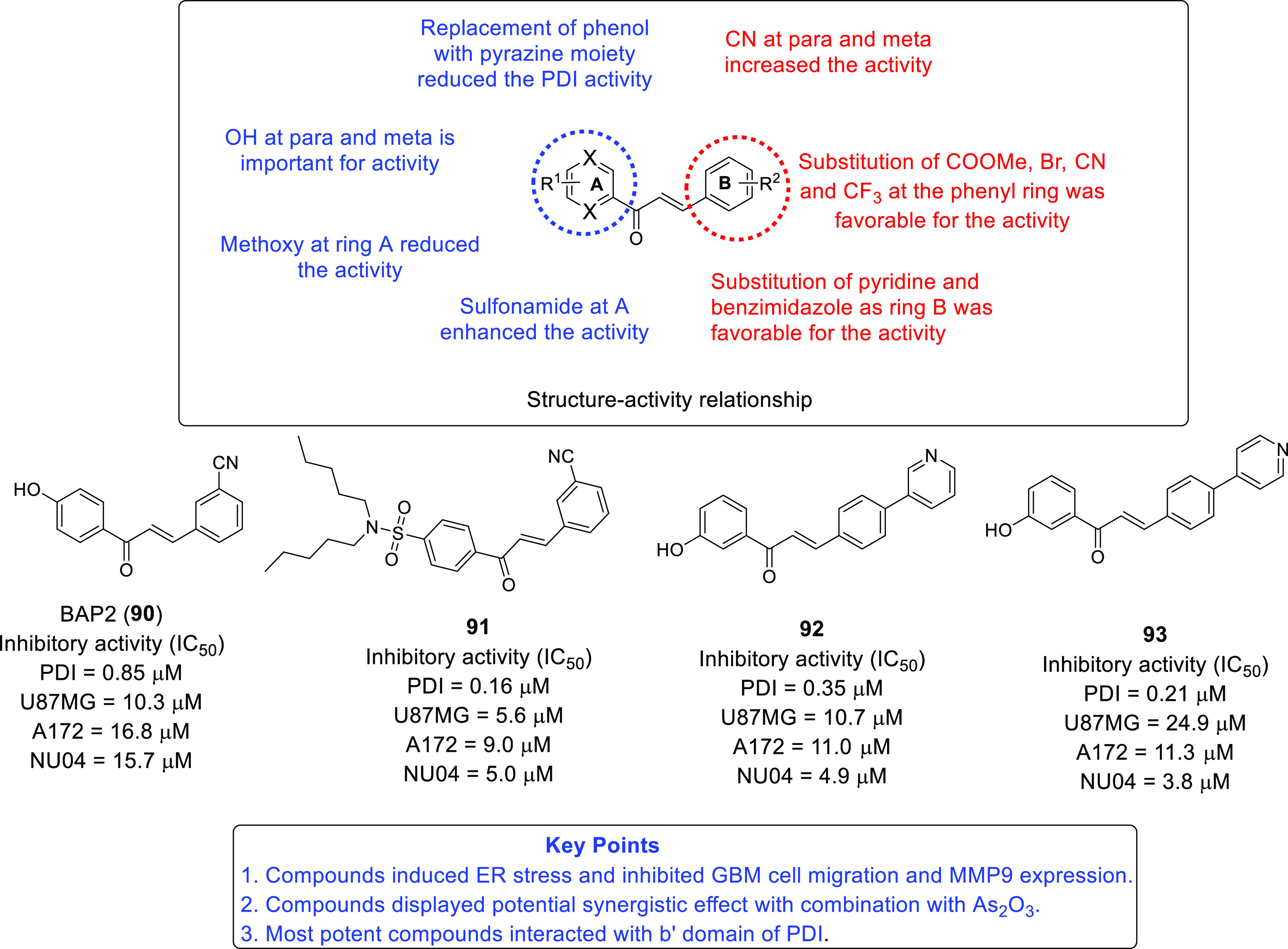
PDI inhibitors for the treatment of GBM.

Encouraged by the involvement of PDI in GBM, Yang et al. unveiled a novel series of allosteric PD1 inhibitors to treat GBM.^71^ The research group conducted a SAR study on the previously identified PDI inhibitor BAP-2 (**90**), obtained from a high-throughput screening (IC_50_ = 930 nM). A total of 68 compounds were synthesized and evaluated against PDI using the PDI reductase assay. For the structural optimization program, the nitrile group was maintained at ring B, and various substitutions were attempted at ring A. A structural scanning program demonstrated that the replacement of hydroxy groups with bromine, methoxy, and amine groups reduced the PDI activity; however, introducing a sulfonamide moiety instead of the hydroxyl group enhanced the PDI activity. Additionally, replacement of the nitrile group with carboxylic acid or carboxymethyl ester at the meta position of ring B led to favorable trends; the resulting compounds displayed better binding toward PDI than chalcones containing electron-withdrawing groups. Furthermore, the replacement of nitrile with a trifluoromethyl group reduced the potency of compounds by 2–4-fold. Overall, the SO_2_NHR substitution at R1 and hydroxy substitution at R2 are the key features for PDI activity. From the preliminary studies, 23 compounds with IC_50_ values lower than 1.5 μM were selected for further evaluations. The selected compounds were tested in a panel of brain cancer cell lines (U-87MG, A172, and NU04), where compounds showed moderate to strong cell growth inhibition in the U-87MG, A172, and NU04 cell lines. Among the evaluated compounds, **91** was the most potent in A172 and NU04 cell lines, with IC_50_ = 5.6 and 9.0 μM, respectively. Additionally, **93** displayed promising results, with IC_50_ = 3.8 μM against NU04 cell lines. In the biochemical thermal shift assay, an elevation of more than 1 °C in melting temperature was observed, strongly indicating that the compounds had good binding interactions with PDI. In contrast, **91** and **93** did not stabilize PDI in the thermal shift assay but still demonstrated good activity, indicating that the compounds might bind to the hydrophobic pocket of the b′ domain. Further analysis revealed that the compounds promoted the ERS response in GBM cells and inhibited cell migration in a dose-dependent manner in the wound-healing assay in A172 cells. Furthermore, the synthesized compounds displayed synergism with arsenic trichloride and DNA damage-inducing radiation therapies, indicating the possible usefulness of these compounds in combination (Figure 24).

**Figure 25:**
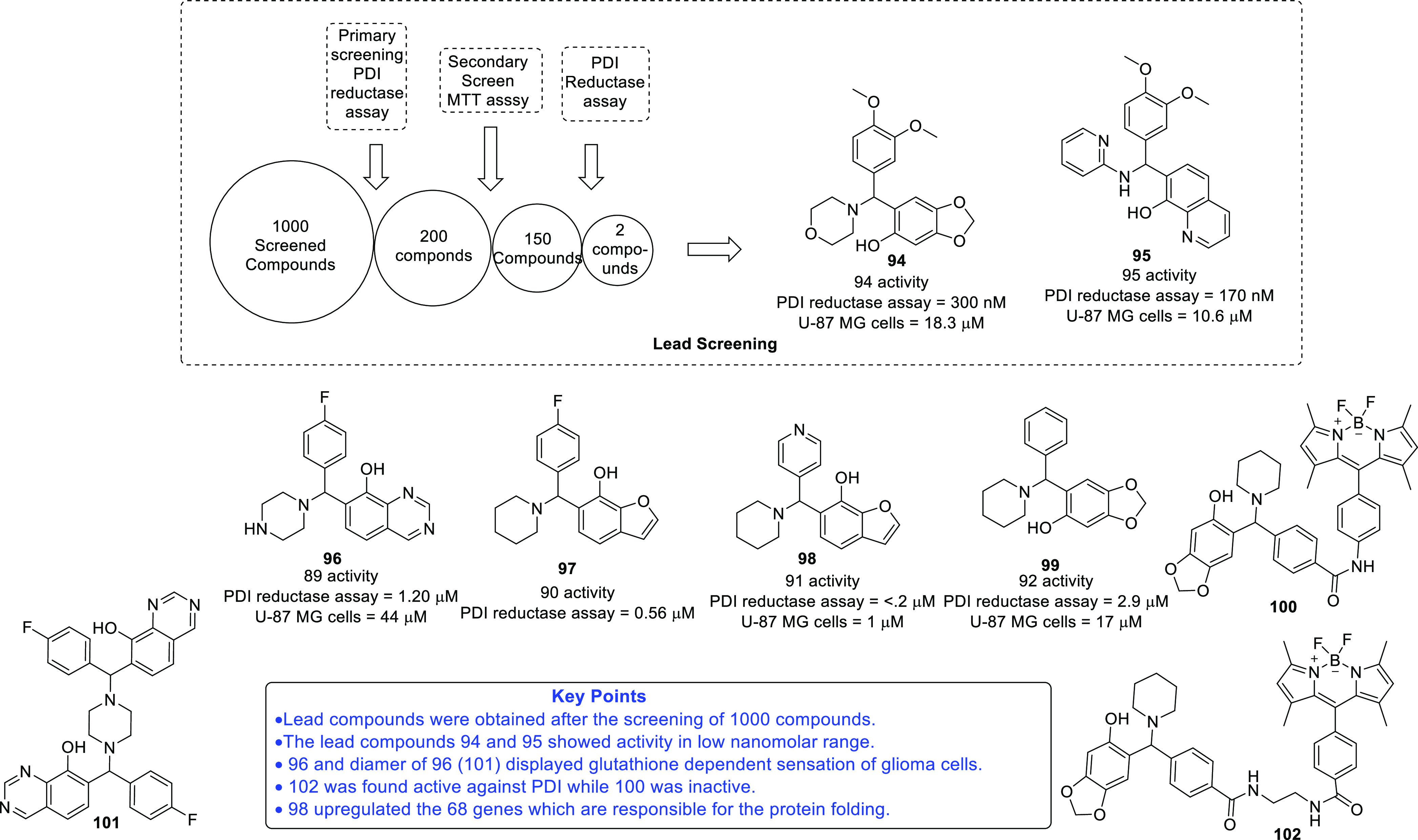
PDI inhibitors lead to optimization for glioma.

In 2020, Shergalis et al. published some aminobenzophenol-based scaffolds as PDI inhibitors to treat GBM.^72^ First, the authors screened approximately 1000 compounds from the National Cancer Institute and found **94** and **95** as potent lead compounds (PDI reductase assay), with IC_50_ = 300 and 90 nM, respectively. The leads were also evaluated against U-87MG GBM cell lines, in which both compounds inhibited cell growth, with IC_50_ = 18.3 μM for **94** and 10.6 μM for **95**. To establish the SAR, a total of 89 compounds from the Chemdiv library and NCI development program were tested using the PDI reductase assay. 5-Hydroxybenzo[*d*][1,3]dioxole substituted with various amines, such as morpholine, piperidine, piperazine, and pyrrolidine, were active and exerted PDI inhibition at an IC_50_ < 1 μM. In contrast, urea substitution was not active, and urea-substituted compounds exhibited a complete loss of activity. For further studies, three series of compounds were synthesized and evaluated PDI activity. Among the synthesized compounds, **96**–**99** showed promising PDI inhibition (Figure 25). Computational analysis suggested that **94** formed a covalent bond to Cys397 or Cys400, and **96** and its diamer, **101**, showed glutathione-dependent sensation of glioma cells. Additionally, the two analogs of **94** with BODIPY fluorescent tags, compounds **100** and **102**, were generated and evaluated for target identification. Subsequently, **102** inhibited the PDI activity in the low micromolar range, with IC_50_ = 1.37 μM, suggesting that **102** can identify the target. Furthermore, **98** was evaluated for gene transcription in U-87-MG cells. It upregulated 68 genes, including CALR, HSAP5, MYZAP, NQO1, SLC7A11, and SLC7A11, which are responsible for protein folding and the knockdown of the KDELR3 proteins, indicating that the compound folds the protein and attains a cysteine reactive signature.

### 4.6 Tubulin Inhibitors

Tubulin is a diametric globular protein that forms microtubules and is responsible for various cellular processes. GBM shows major alterations in the cytoskeleton of microtubules, such as anomalous γ-tubulin and class III β-tubulin isotype (βIII-tubulin) expression, which are linked to anaplastic tumor phenotypes.^533−538^ Additionally, the common multi-lineage of the antigenic phenotype (fibronectin+/CD44+/vimentin+/microtubule associated protein-2+/GFAP+/βIII-tubulin+) was found in undifferentiated GBM cells and glial fibrillary acidic protein (GFAP)+ normal neural progenitors.^539^ Moreover, the expression of GFAP+/nestin+/βIII-tubulin+ cells in GBMs and normal human fetal astrocytes has been reported *in vitro*, indicating the involvement of tubulin in the progression and survival of GBM.^540,541^

**Figure 26:**
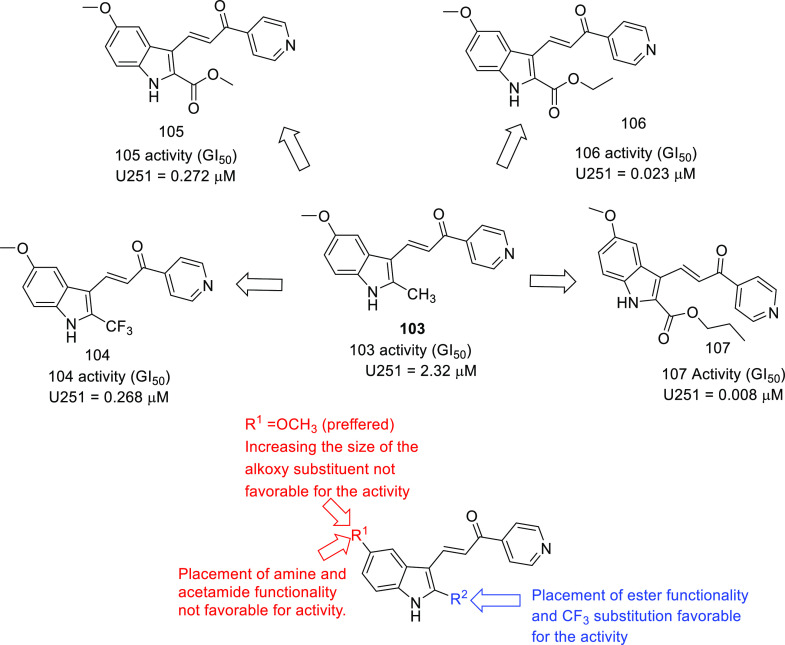
Indolyl propenones as putative anti-cancer drug against GBM.

No apoptotic cell death, or methuosis, involves the accumulation of macropinosomes, leading to the loss of membrane integrity. The literature indicates that small molecules with methuosis-inducing ability outshine the candidates for apoptosis-inducing conventional anti-cancer drugs. To exploit the above-mentioned information, Trabbic et al. initiated a medicinal chemistry campaign centered on the privileged bicyclic heteroaryl ring, indole, and reported a series of indolyl-based pyridinyl propenones.^73^ In the study, a previously reported methuosis inducing **103** was used as a lead. Well equipped with the structural requisites for methuosis-inducing ability of the lead **103**, such as para-pyridinyl, methoxy substitution (5-position), and small alkyl group (2-position), the authors first used the structural optimization program to determine the impact of various substitutions of the indole ring, as shown in Figure 26. Excitingly, some substitutions were pinpointed to mediate the anti-tumor effects from methuosis to microtubule disruption. In total, 17 compounds were synthesized and evaluated for their cell growth inhibitory effects against U251 GBM cell lines. Among the synthesized compounds, **104**–**107** displayed promising results with GI_50_ = 0.268, 0.272, 0.023, and 0.008 μM, respectively. Furthermore, **106** and **107** caused considerable accumulation of cells at the G2/M phase and increased the percentage of cells in the sub-G1/G0 phase. Additionally, the effect of selected **104**–**107** on microtubule polymerization was evaluated in U251 GBM cells using immunofluorescence microscopy. In this study, **107** distracted the staining pattern at a dose of 0.1 μM. In the biochemical method, polymerization disruption was also observed when **104**, **106**, and **107** were analyzed by Western blot analysis. Collectively, the switch in the mechanism responsible for the cytotoxic effects of the compounds from methuosis to microtubule disruption was accompanied by a substantial increase in potency.

**Figure 27:**
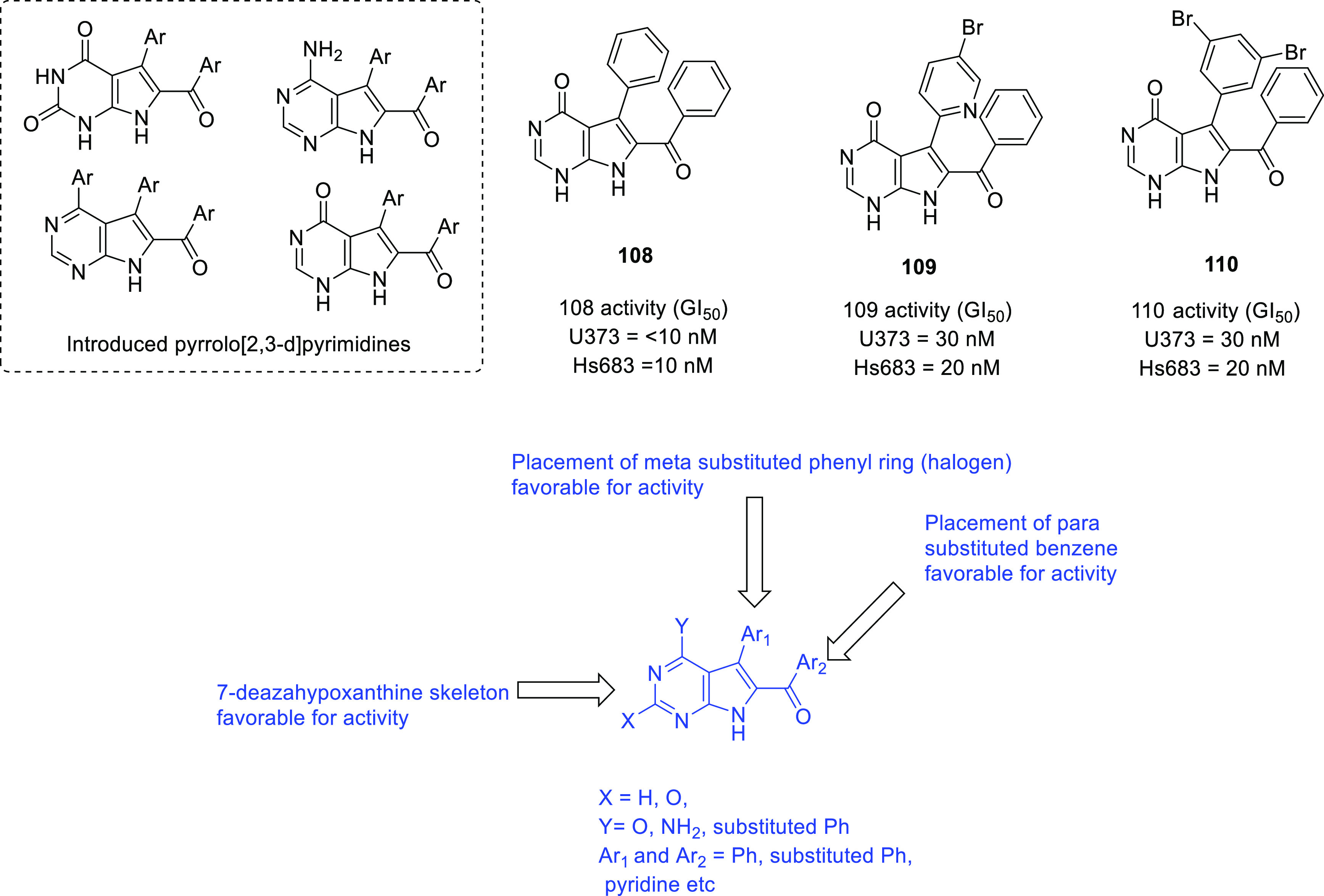
Pyrrolo[2,3-*d*]pyrimidines as potent tubulin-targeting scaffolds.

Owing to the magnificent anti-tumor profiles of marine pyrrole-derived alkaloids, Frolova et al. conducted a medicinal chemistry study on marine alkaloid rigidins and furnished 7-deazaxanthine, 7-deazaadenine, 7-deazapurine, and 7-deazahypoxanthine skeleton-based scaffolds.^74^ The adducts were evaluated for anti-tumor effects against glioma cell lines (U373, Hs683). The results were overwhelmingly positive, with the compounds demonstrating striking cell growth inhibitory effects in the two-digit nanomolar range (Figure 27). Mechanistic studies were conducted to elucidate the mechanism of the compounds revealing that **109** and **110** potentially disrupted the polymerization assembly and the microtubule cytoskeleton with GI_50_ = 30 nM against U373 cells and 20 nM against the Hs683 cell line.

**Figure 28:**
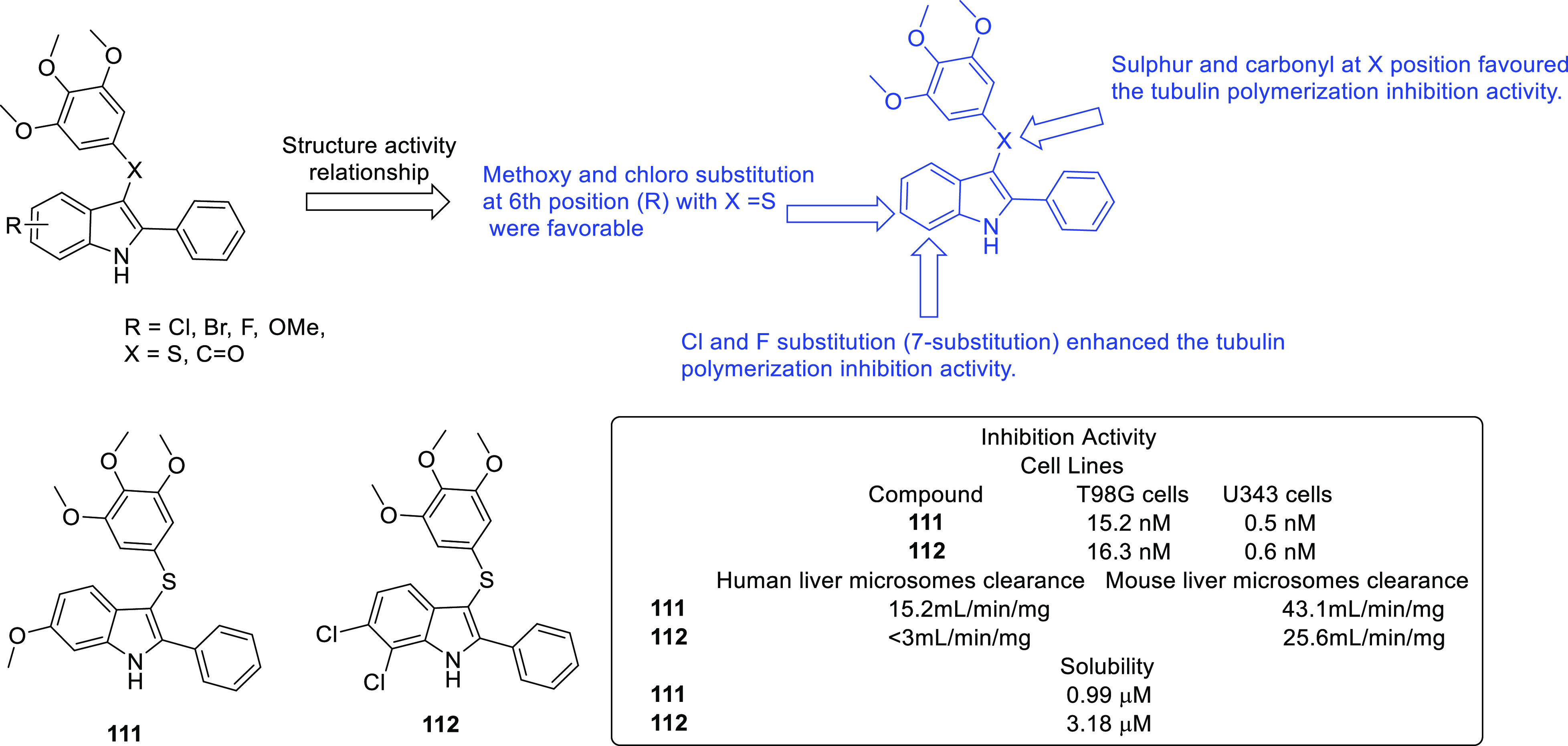
Indole-based anti-cancer agents.

In 2015, La Regina et al. designed indole-based tubulin assembly inhibitors that arrested mitotic progression and enhanced natural killer cell stimulation with hedgehog-based cancer cell inhibition.^75^ Specifically, a 2-phenylindole core was selected for exploration, and 39 compounds containing the 3,4,5-trimethoxyphenyl moiety were furnished. Methylene, ketone, or sulfur bridging groups were leveraged to tether indole cores, and 3,4,5-trimethoxyphenyl moieties and halogen or methoxy substituents were placed at positions 4–7 of indoles. The synthesized compounds were evaluated for tubulin and colchicine binding inhibition. Twenty-six compounds displayed significant inhibition in the low nanomolar range, and **111** and **112** were identified as the most potent in the series with IC_50_ values as follows: **111**, IC_50_ = 1.1 μM (tubulin assembly), colchicine binding inhibition = 96%; **112**, IC_50_ = 1.2 μM (tubulin assembly), colchicine binding inhibition = 92% (Figure 28). Furthermore, both compounds were evaluated against T98G and U343MG cells, in which both compounds significantly inhibited cell growth, with IC_50_ = 15.2 ± 1.6 nM in T98G cells, 0.5 ± 0.05 nM in U343 cells, 16.3 ± 1.5 nM in T98G cells, and 0.6 ± 0.05 nM in U343 cells. Additionally, both compounds showed moderate metabolic stability, including human microsomal stability, and solubility. Thus, **111** and **112** were active against GBM, and further optimization is required to establish their detailed preclinical profile (Figure 28).

**Figure 29:**
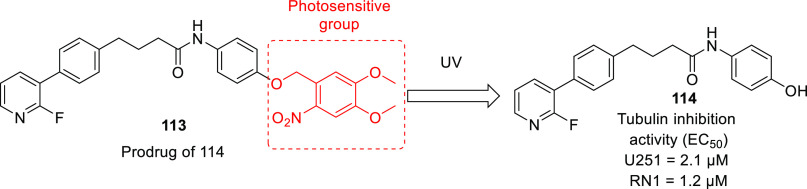
Photosensitive activation of microtubules destabilizing agent.

Given the findings of the ability of photoremovable protecting groups to confer spatial and temporal control of the biological effects to the scaffolds, Döbber et al. designed a prodrug of the potent tubulin inhibitor **114** leveraging the photosensitive DMNB group to block the pharmacophoric OH group of the compound.^76^ Subsequent explorations indicated that the prodrug demonstrated UV radiation-controlled anti-tubulin activity against glioma U251 and RN1 cells with EC_50_ = 2.1 and 1.2 μM, respectively. Additionally, **114** showed a significant impact on tubulin polymerization and promoted the apoptotic cell death of cancer cells (Figure 29). The outcome of the study clearly presents photosensitive activation of the prodrug as a useful strategy to attain selectivity toward cancer cells and normal cells.

**Figure 30:**
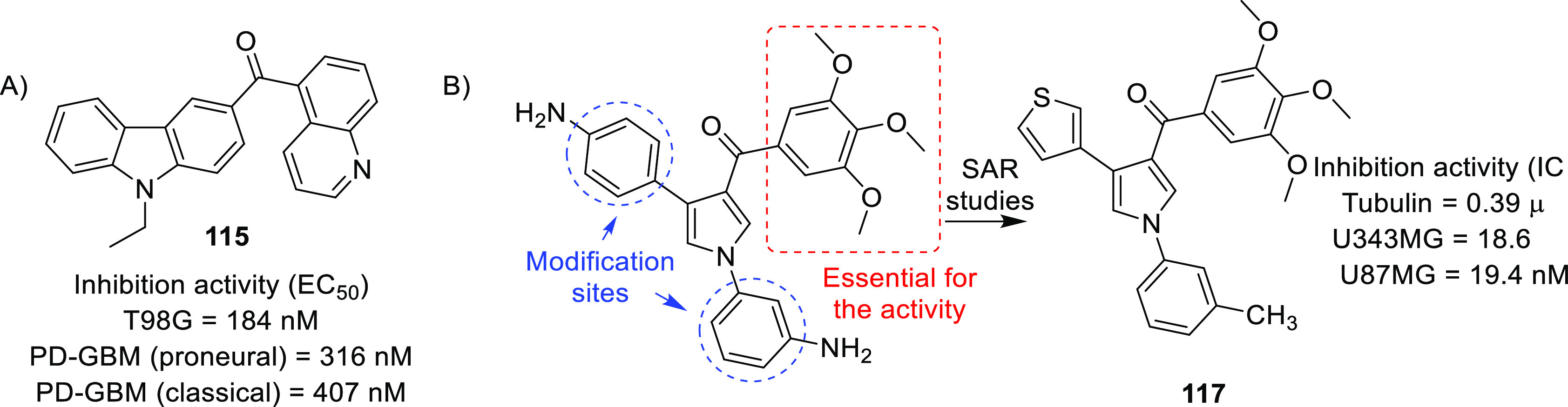
(A) Modified carbazoles as anti-GBM agents. (B) Pyrrole derivatives as tubulin targeting agents for GBM.

Diaz et al. revealed a series of modified carbazoles as microtubule destabilizing agents that potentially inhibited the growth of GBM cell lines.^77^ A series of various substituted carbazoles were synthesized and evaluated against T98G cells. In the series, **115** was found to be a promising compound, with IC_50_ = 184 nM. Furthermore, **115** was screened in patient-derived GBM cell lines, namely, PD-GBM (proneural), PD-GBM (classical), and PD-GBM (mesenchymal), and it potentially inhibited cell growth, as shown in Figure 30A. Moreover, the colchicine binding assay and docking studies revealed that **115** binds to the colchicine binding site of tubulin and disrupts microtubule stability, which leads to GBM cell death.

In 2021, Puxeddu et al. revealed the development of pyrrole derivatives as potential tubulin and hTopo inhibitors and found them to be effective against resistant GBM cells.^78^ In the development of pyrrole derivatives, structural engineering was carried out over lead compound **116**, which resulted in a series of compounds that were further evaluated for tubulin activity. Among the synthesized compounds, **117** displayed promising activity against tubulin, with IC_50_ = 0.39 μM. Furthermore, the activity of **117** was evaluated for hTopoI and hTopo 2, where **117** selectively inhibited hTopo II expression at 100 μM, whereas no activity was observed over hTopo I. Moreover, compound **117** showed potential inhibition against U343MG and U-87MG GBM cells (as shown in Figure 30B) and inhibited cancer proliferation, tumor angiogenesis, and *in vivo* tumorigenesis in a murine GBM model. Overall, the discovery of **117** opens the door for pyrrole derivatives as potential therapeutics against GBM.

**Figure 31:**
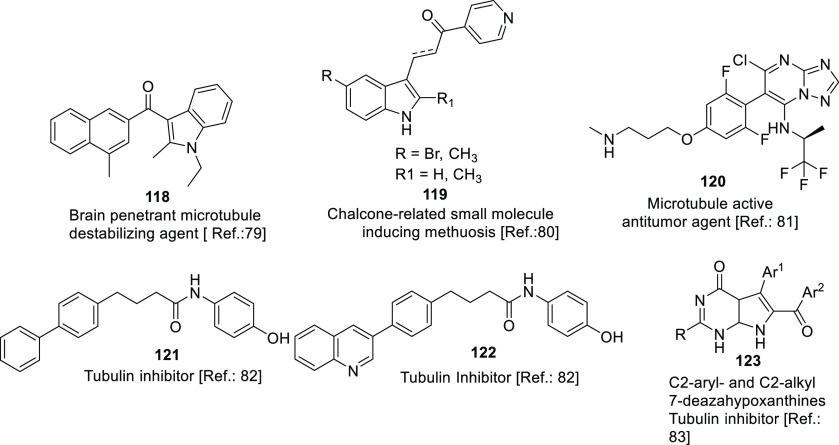
Microtubule disrupting agents for the treatment of glioma.

The chemical architectures of other recently reported microtubule disrupting agents with anti-GBM activity are shown in Figure 31.^79−83^

### 4.7 Hypoxia-Inducible Factor (HIF) Pathway Inhibitors

HIF is a key heterodimeric transcription factor that activates various transcription genes involved in tumor survival, invasion, angiogenesis, and glucose metabolism^542^ under hypoxic conditions in cancer. Stabilization/increased HIF1α expression results from the activation of the PAM pathway through overexpression of the EGFR gene and loss of PTEN, resulting in vascularization of tumors in GBM.^543,544^ Additionally, integrins activate the PAM pathway through extracellular matrix (ECM) adhesion, integrin-linked kinase (ILK) activation, a surge in HIF-1α, and VEGF production in GBM.^545,546^ Moreover, it was found that HIF-1α requires elevated concentrations of heat shock proteins 70 and 90, which induce tumor progression.^547^

**Figure 32:**
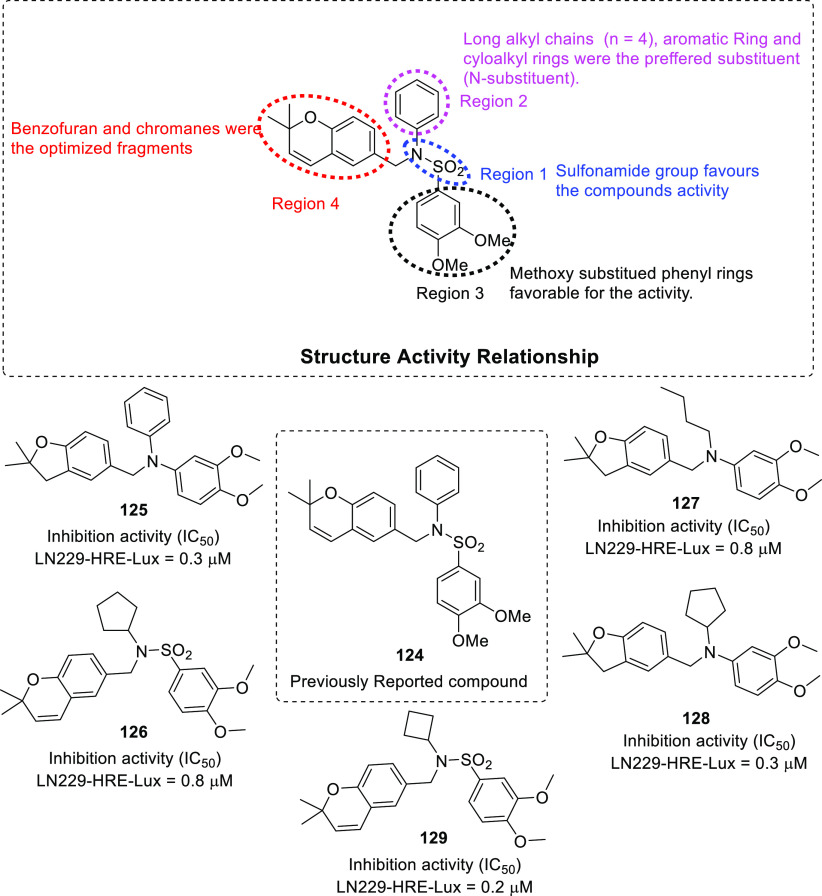
HIF pathway inhibitors as anti-cancer agents.

To develop potent HIF pathway inhibitors, Mooring et al. conducted a structural optimization campaign of previously reported compound **124**.^84^ For the structure analysis, **124** was divided into four regions, and each was explored in the context of substituent preference. Various substitutions were attempted at each region, and a series of compounds was accomplished that were further evaluated against HIF-1-mediated transcription in the LN229-HRE-Lux glioma cell line. Among the synthesized compounds, **125**–**129** were the most potent in series, with IC_50_ = 0.3, 0.8, 0.4, 0.3, and 0.2 μM, respectively. The SAR of the compounds is summarized in Figure 32. Additionally, compounds **125** and **126** suppressed the expression of HIF-1α, as demonstrated by Western blot analysis. In conclusion, the identified HIF pathway inhibitors exhibited beneficial effects against hypoxic tumor resistance to chemotherapy and radiotherapy.

**Figure 33:**
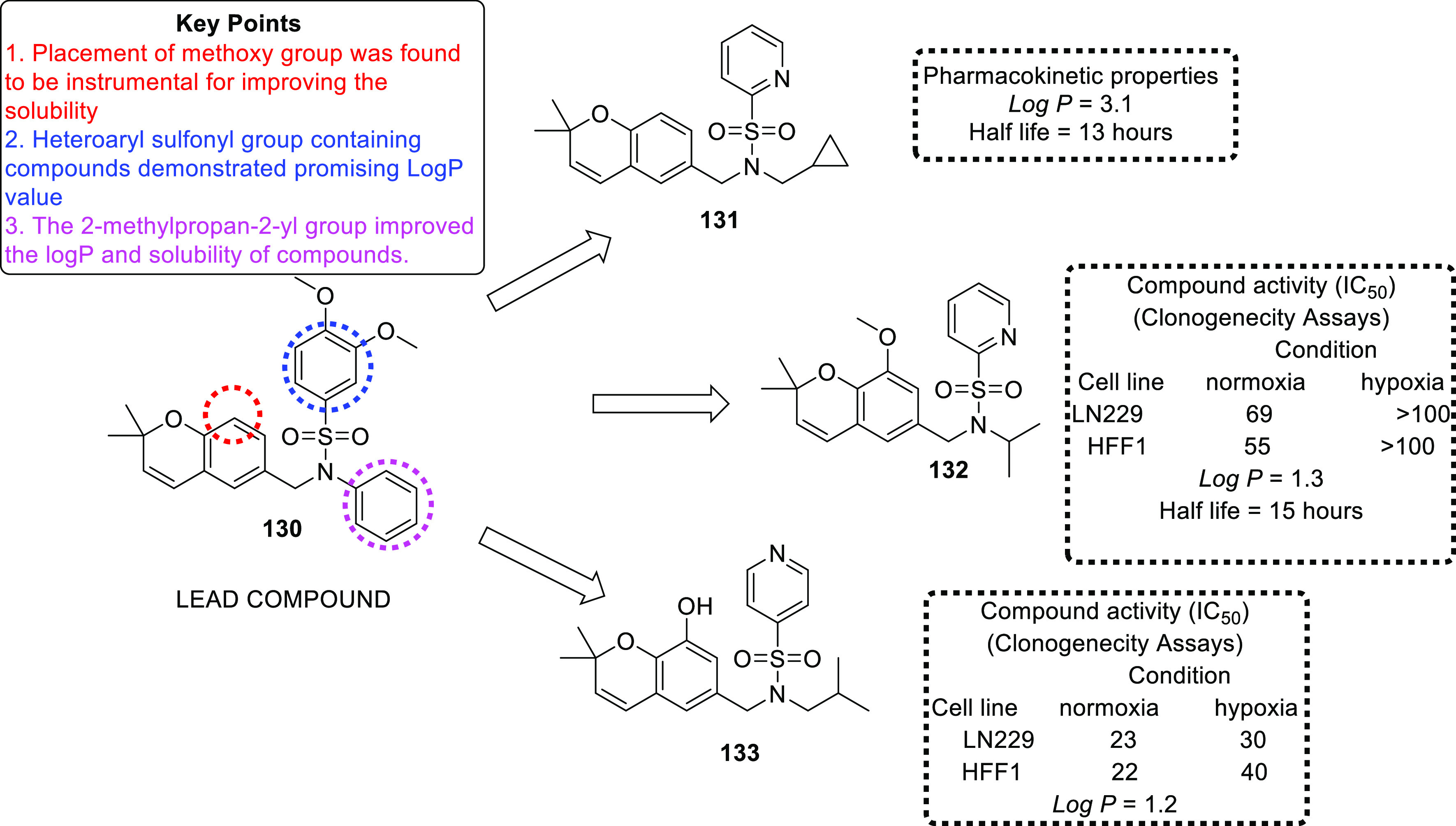
HIF-1 pathway inhibitors as anti-cancer agents.

In 2012, Mun et al. reported a novel HIF-1 pathway inhibitor with improved pharmacological properties as a potential anti-glioma agent.^85^ The group used compound **130**, which was previously identified through high-throughput screening as a lead. The lead modification study was initiated to overcome the limitation of the poor aqueous solubility of **130** that was pinpointed as an obstacle for the *in vivo* evaluations. The research group furnished a series of 12 compounds with molecular weights ranging from 371 to 403 g/mol. All the synthesized compounds were evaluated for HIF transcriptional activity in LN229-V6R cell lines, and the compounds showed promising results with IC_50_ values under or close to 5 μM (Figure 33). The compounds also demonstrated efficacy under hypoxic conditions, as demonstrated by Western blot. Among the tested compounds, **132** and **133** showed a reduction in HIF-1α activity at a concentration of 100 μM. Additionally, **132** and **133** elicited substantial cell growth inhibitory effects (LN229-V6R) under normoxic and hypoxic conditions, with IC_50_ = 73 and 146 μM and 92 and 113 μM, respectively. Furthermore, the aqueous solubility of compounds was determined, and the results confirmed the improved aqueous solubility of **131** and **132** (LogP = 3.1 and 1.3). Notably, *N*-[(8-methoxy-2,2-dimethyl-2*H*-chromen-6-yl)methyl]-*N*-(propan-2-yl)pyridine-2-sulfonamide (**132**) demonstrated a solubility improvement of ∼9000-fold compared with that of the lead. Additionally, both compounds showed an optimum PK profile, according to the results of the metabolic studies conducted in homogenized mouse liver, where **131** and **132** displayed *t*_1/2_ = 13 and 15 h, respectively. Overall, the designed compounds demonstrated promising efficacy coupled with an optimum PK profile, and the findings can be leveraged as an initiation point of several anti-glioma drug discovery campaigns.

### 4.8 Multi-targeting Compounds/Cocktail of Drugs

**Figure 34:**
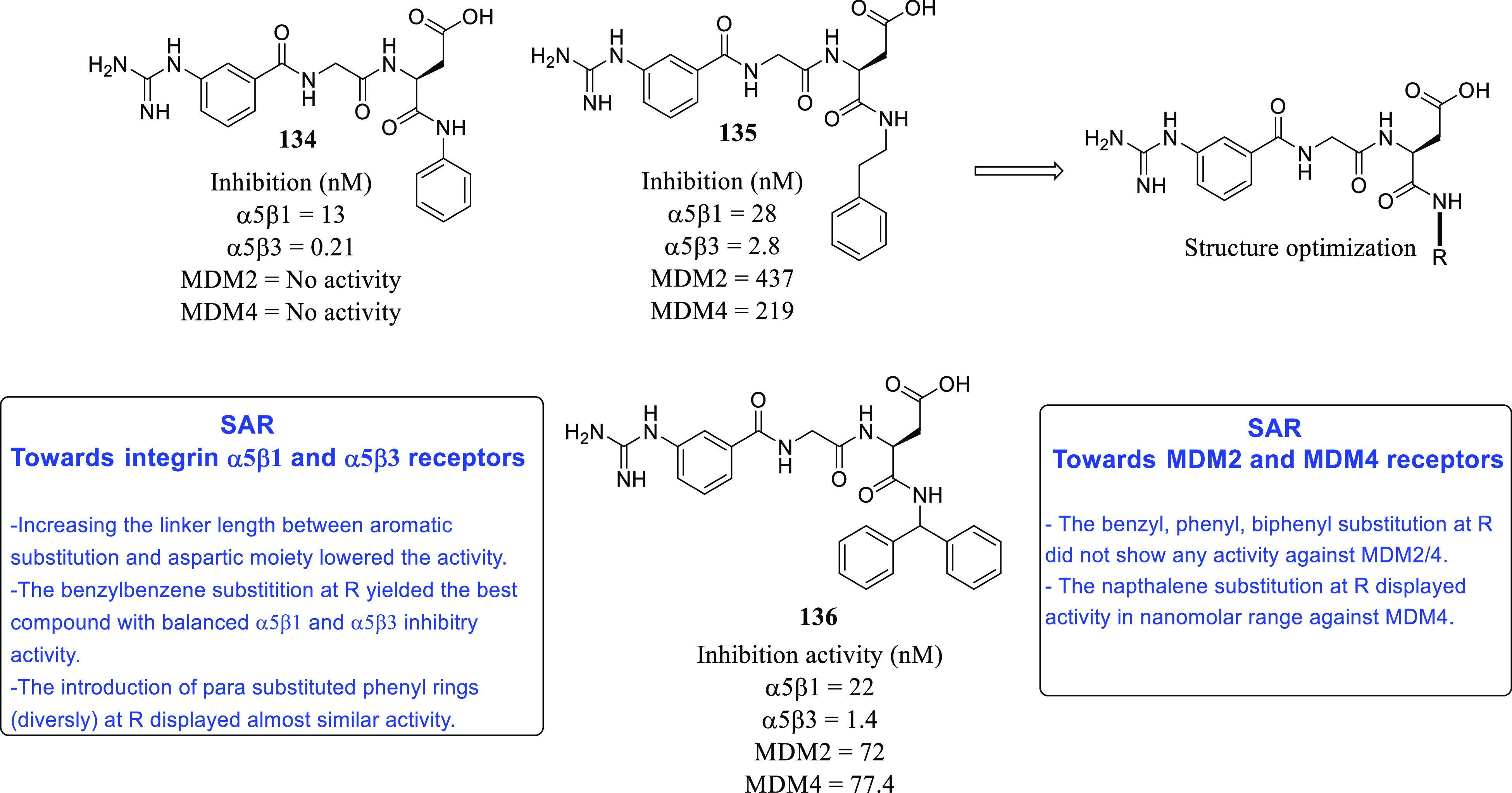
RGD integrins and dual MDM inhibitors for the treatment of GBM.

To extract the evidenced benefits attained from the simultaneous inhibition of multiple targets in cancer, Merlino et al. in 2018 reported a series of RGD integrins and dual MDM protein inhibitors to treat GBM.^89^ The group considered the chemical architecture of previously reported compounds **134** and **135** as a lead compound for the structural optimization program. Thus, **135** showed good integrin inhibitory potential coupled with a magnificent activity profile toward MDM2 and MDM4 (IC_50_ = 437 and 219 nM, respectively). The structural tuning program was planned to confer enhanced potency and a balanced activity profile to the new analogs of **135** toward both integrin and MDMs. In total, 14 compounds were synthesized and evaluated against integrin binding fibronectin (α5β1) and vitronectin (αvβ3) and the human p53/MDM2 or p53/MDM4 complex. Among the synthesized compounds, **136** was identified as the most potent in the series. Furthermore, the p53 protein-mediated activity of integrins and MDM2/4 inhibitor **136** was investigated in U-87MG GBM cells (Figure 34). The cells were treated with the standard integrin MDM2 inhibitor Nutlin-3 with or without **136**, and the p53 protein level was evaluated. Compound **136** was more efficacious than the combination of inhibitors. Continued investigations also confirmed that MDM2 inhibition plays a crucial role in the regulation and transcriptional control of p53, while MDM4 inhibition significantly increased PUMA gene transcription and triggered apoptosis. The anti-proliferative activity of **136** was evaluated against GBM cells and human T98G cells (exhibiting mutated p53), where it inhibited GBM cell growth with IC_50_ = 116 ± 10 nM, while efficacy against T98G cells was observed at micromolar concentrations. Additionally, a docking study of **136** with corresponding binding pockets was performed. In both αvβ3 and α5β1, **136** was bound through the canonical RGD binding pattern and interacted with the major amino acids Y122, S123, N215, Y133, S134, N224, Q221, D227, and D227. In MDM2, NMR and docking were performed, revealing that the biphenyl moiety of **136** reached the W23 and F19 pockets and that the remainder of the molecule was oriented toward the L2 loop or flipped into the N-terminal region. In the NMR study, a massive chemical shift was observed in the A13, S22, R29, K51, F55, Y56, G58, Y60, M62, F91, S92, V93, K94, and I103 amino acids, indicating that these residues might interact with **136**. The overall study led to the identification of multi-targeting RGD integrins and dual murine double minute protein inhibitors as anti-glioma agents that emerge as suitable alternatives to combination therapy.

**Figure 35:**
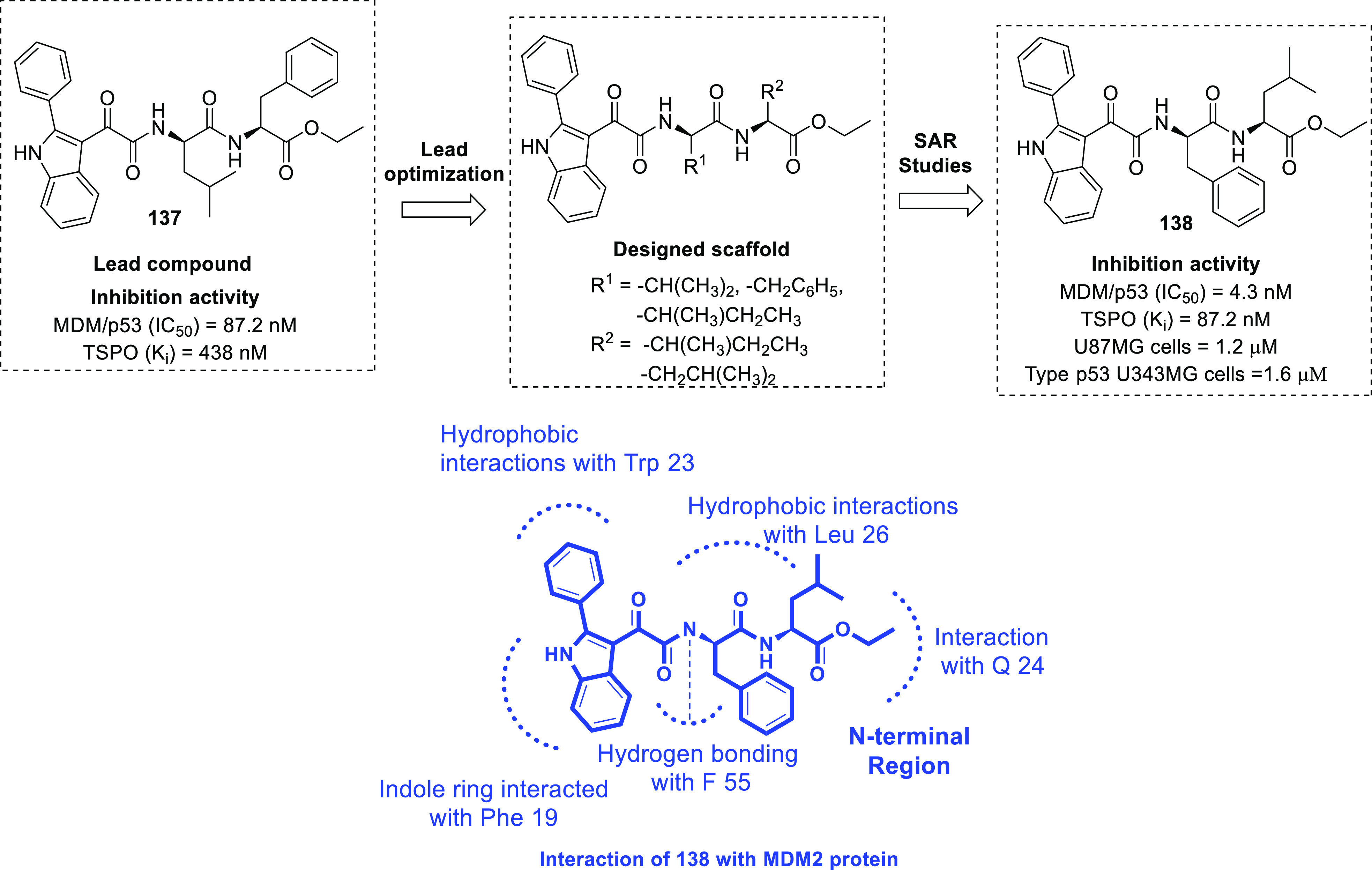
MDM-2 and TSPO for the treatment of gliomas.

In 2016, Daniele et al. optimized 2-phenylindolylglyoxylyldipeptide, a lead dual inhibitor of MDM-2 and TSPO (**137**, MDM/p53 interaction IC_50_ = 11.65 nM, TSPO *K*_i_ = 438 nM), to treat gliomas.^86^ To optimize the structural features, six compounds were synthesized and investigated as MDM2/p53 complex disruptors in U-87MG cells. Among the series, five compounds displayed promising inhibition in the IC_50_ range of 4.3–24.8 nM, and **138** was the most potent in the series, with IC_50_ = 4.3 nM. Furthermore, the binding affinity of selected compounds toward TSPO was evaluated using radioligand binding assays, in which **138** showed good binding activity with *K*_i_ = 87.2 nM, which was better than that of lead **137** (*K*_i_ = 438 nM). Docking studies of the most potent **138** were performed (MDM2 protein), in which the phenyl ring attached to the indole interacted with the Trp23 pocket and showed hydrophobic interactions with the L57, I61, F86, F91, I99, and I103 side chains, while the indole ring was involved in the interaction with Phe19. The glyoxylamide-NH formed a hydrogen bond with L54, and the side chain was involved in hydrophobic interactions with Leu26 residues I19, Y100, L54, and M50, while the methyl ester moiety interacted with Q24 residues of the binding pocket (Figure 35). Cell apoptosis studies suggested that the dual MDM-2/TSPO inhibitor triggered GBM cell apoptosis and caused cell cycle progression in the G2/M phase. Additionally, evaluation of the anti-proliferative activity of **138** in U-87MG and wild-type p53 U343MG cells demonstrated the remarkable inhibitory potential of **138** (IC_50_ = 1.2 and 1.6 μM, respectively). Overall, dual MDM-2/TSPO inhibitors were effective against glioma cell lines and can be used as therapeutic agents against cancer where p53 signaling is affected and TSPO is overexpressed.

**Figure 36:**
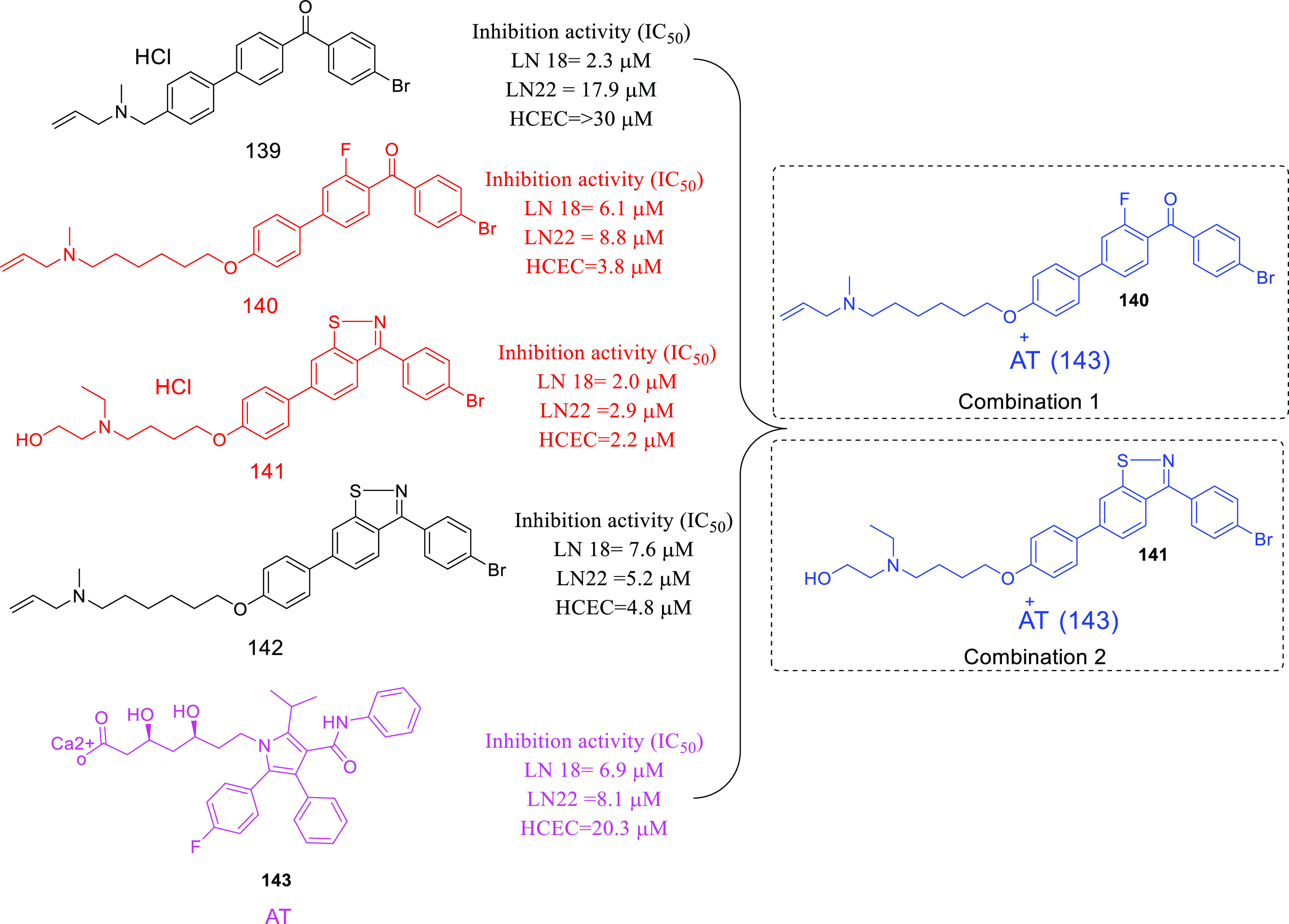
Combination of oxidosqualene cyclase inhibitors with Atorvastatin for the treatment of glioma.

In 2012, Staedler et al. reported that an effective combination of oxidosqualene cyclase inhibitors with atorvastatin (**143**) can yield conclusive benefits in the context of cancer treatment.^90^ To exploit these disclosures, the authors initiated a medicinal chemistry campaign and furnished 10 oxidosqualene cyclase inhibitors. The inhibitors were evaluated in 11 cancer cell lines derived from various tissues and in one non-tumoral human brain-derived endothelial cell line. All the synthesized compounds displayed good cell proliferation inhibition with IC_50_ values in the micromolar range. Among the series, **139**–**142** were the most potent against LN18 and LN229 GBM cells and HCEC brain-derived endothelial cells. Considering the above results and quest to amplify the anti-tumor potential, the combination of the most potent compounds and atorvastatin (**143**) was evaluated in human GBM LN18 and LN229 cells and non-tumoral HCEC endothelial cells. Compounds **140** and **141** in combination with AT (**143**) displayed promising results in the context of synergistic anti-cancer efficacy. Overall, the combination of oxidosqualene cyclase inhibitors with atorvastatin (**143**) could serve as a good combination to treat GBM (Figure 36).

**Figure 37:**
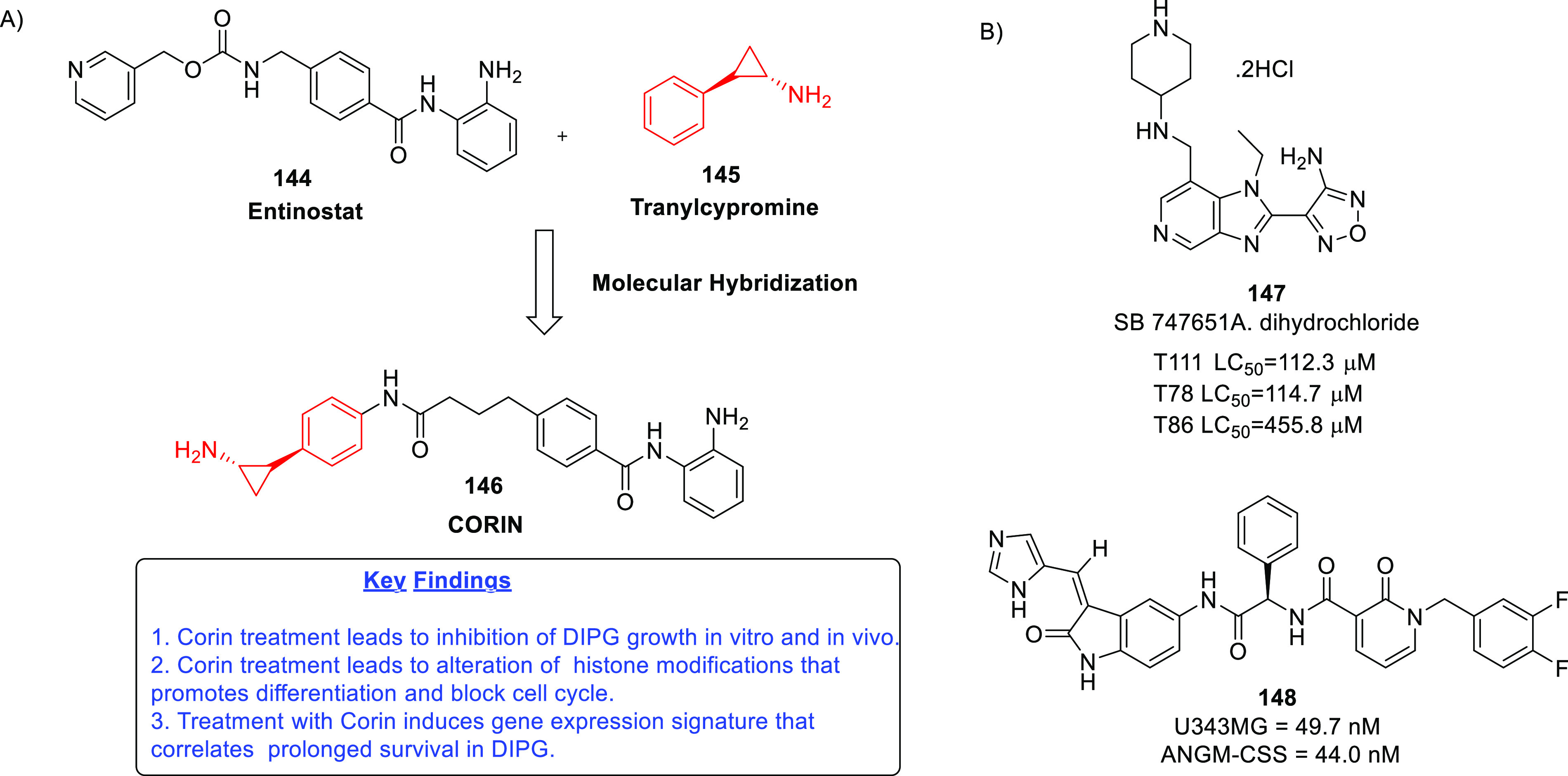
(A) Corin as a potential anti-glioma agent. (B) Multi-targeting compounds **147** and **148**.

Corin (**146**) is a synthetic hybrid agent that comprises the structural attributes of entinostat (**144**), a class I HDAC inhibitor, and tranylcypromine (**145**), an LSD1 inhibitor (Figure 37A). Recent studies centered on explorations of the mechanisms involved in diffuse intrinsic pontine glioma (DIPG), an incurable pediatric cancer, have revealed that H3K27M mutations contribute to epigenetic dysregulation. Given the above-mentioned findings, the potential of corin was evaluated to treat DIPG, and it was found that the H3K27me3 levels suppressed by H3K27 M histones were increased by corin treatment. Additionally, corin (**146**) increased HDAC-targeted H3K27ac and LSD1-targeted H3K4me1 at differentiation-associated genes. The induction of cell death, cell cycle arrest, and a cellular differentiation phenotype was observed with corin treatment along with transcriptional changes correlating with increased survival time in DIPG patients. The outcome of this study clearly shows that dual HDAC-LSD1 inhibition is a logical strategy to treat DIPG.^87^

In 2021, Arnon and colleagues selected the small-molecule inhibitor SB747651A (**147**) to explore its activity in GBM. SB747651A (**147**) is a multi-targeted small molecule that inhibits multiple pathways, including the MAPK, PI3K-Akt-mTOR, and JNK pathways. The team investigated this molecule using three well-characterized patient-derived GBM spheroid cultures (T111, T86, and T78). The results of the cell viability and apoptosis assays suggested that SB747651A, when combined with TMZ (alkylating chemotherapeutic agent), induced apoptosis-mediated cell death. SB747651A (Figure 37B) was next subjected to the limiting dilution assay, which demonstrated inhibited spheroid formation in all three glioma spheroid cultures. Additionally, the outcome of the cell migration assay indicated the ability of SB747651A to reduce the migration distance in T78 and T86 cells by 37.8% and 60.4%, respectively, at a concentration of 10 μM. To identify the mechanism of action of **147**, a phosphoprotein antibody array kit was used, and the results showed that **147** inhibited the phosphorylation of GSK3, CREB, mTOR, and SOX2 in cancer cells. *In vivo* evaluation of **147** using orthotopic xenograft mice and T78 spheroid culture showed a longer median survival of 128 days. Additionally, no acute lethal toxicity, behavioral changes, or weight loss was noticed in the mice with continuous treatment at a dose of 25 mg/kg for 5 weeks.^91^

In 2017, Daniele et al. revealed a dual inhibitor of PDK1 and aurora kinase (**148**) that inhibited the growth of U343MG and ANGM-CSS (an immortal cell line derived from a human GBM), with IC_50_ = 49.7 and 44.0 nM, respectively (Figure 37B).^88^ Additionally, **148** displayed a significant effect on cancer cell proliferation, triggered cellular apoptosis, and reduced tumor invasiveness. Thus, **148** appears sufficiently promising for detailed investigation in GBM.

To improve the efficacy and reduce the drug resistance of TMZ, Sahli et al. introduced hybrid drug nanoparticles of gold-TMZ combined with gemcitabine (GEM) and decitabine (DAC).^92^ The hybrid nanoparticles were formulated using the “Method In” strategy, evaluated in U-87 GBM cells, and characterized by Raman spectroscopy, UV–vis spectroscopy, and transmission electron microscopy (TEM). It was found that the nanoparticles of TMZ in combination with GEM and DAC displayed a synergistic effect and inhibited U-87 cells more predominantly over the glycolysis pathway than TMZ alone. Moreover, it was found that the formulation is suitable for the thermal destruction of cancer. Overall, the delivery of multiple drugs as a cocktail showed promising growth and opened the door to develop more efficacious drug combinations.

### 4.9 Degrader and PROTACs

**Figure 38:**
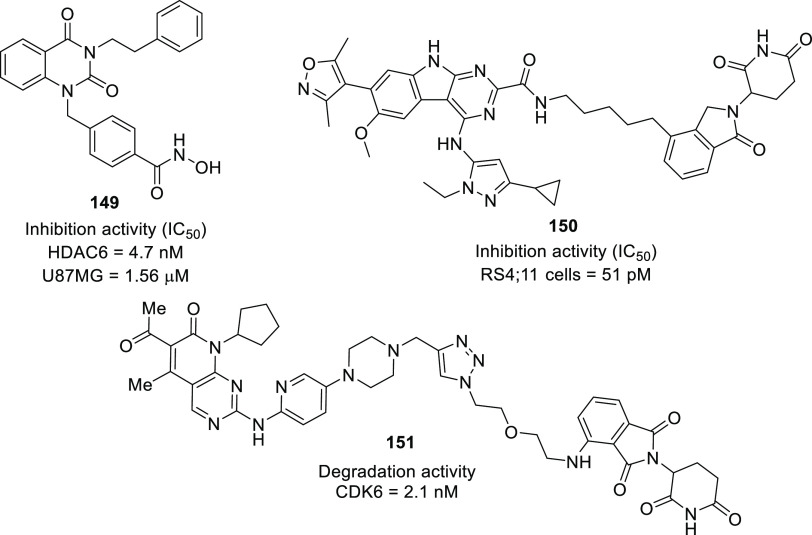
Degraders as anti-GBM agents.

In 2019, Liu et al. reported a highly selective HDAC6 inhibitor **149** with PROTAC-like efficacy for the treatment of GBM.^548^ Compound **149** caused p62 accumulation and proteasomal degradation, leading to proteolysis of aberrantly overexpressed HDAC6 isoforms in GBM. Additionally, **149** demonstrated substantial cell growth inhibitory effects on the U-87MG glioma cell line (IC_50_ = 1.56 μM), decreased cell migration, increased autophagic cancer cell death, and reduced the immunosuppressive activity of PD-L1 (Figure 38).

Recently, Tian et al. reported a BET degrader, ZBC260 (**150**), as a potent inhibitor of tumor progression and stem cell-like cells following Wnt/β-catenin signaling (Figure 38).^549^ First, the anti-proliferative activity of ZBC260 was evaluated in glioma cell lines (U-87, U251, H4, and A172 cell lines), revealing substantial dose-dependent cell growth inhibitory effects of **150**. Western blot analysis further demonstrated that ZBC260 (**150**) downregulated the expression of BRD2/3/4 in glioma cell Lines U-87, U251, H4, and A172. Subsequent studies revealed that ZBC260 arrested cell growth at the G2/M phase, promoted the expression of p21, p27 Bax, cleaved caspase-3, and caspase-9 and suppressed cyclin D1, cyclin B1, BCl-2, and BCl-X_L_.^550^ Furthermore, the effect of ZBC 260 on cell invasion, migration, and EMT was evaluated, and **150** downregulated the expression of the epithelial markers N-cadherin, SNA12, CD44, and vimentin and inhibited cell invasion and migration. ZBC260 also demonstrated *in vivo* anti-tumor potential and decreased the levels of Ki-67, Bcl-2, and PCNA. Notably, the stem cell-like markers ALDH-1, KLF4, SOX2, NANOG, and ABCG2 were significantly inhibited by ZBC 260, indicating that the compound can also inhibit CSCs. The mechanism of stem-like cell inhibition was studied by analyzing the expression of the proteins/genes GLI1, NICD1, and β-catenin, which are involved in maintaining CSCs. The compound reduced the expression of β-catenin, as confirmed by immunohistochemical and Western blot analyses.

In 2019, Su et al. developed a pomalidomide-based PROTAC with palbociclib (**151**) that targets and degrades CDK6 in cancer cells.^551^ POTAC CP10 (**151**) effectively degraded CDK6 in U-87 GBM cells with degradation rates of 72% and 89% at doses of 10 and 100 nM, respectively (Figure 38). Overall, the selective degradation of CDK by PROTAC in glioma cell lines determines the usefulness of degraders as a potential tool for anti-glioma therapy.

### 4.10 Natural-Product-Based Anti-GBM Agents

**Figure 39:**
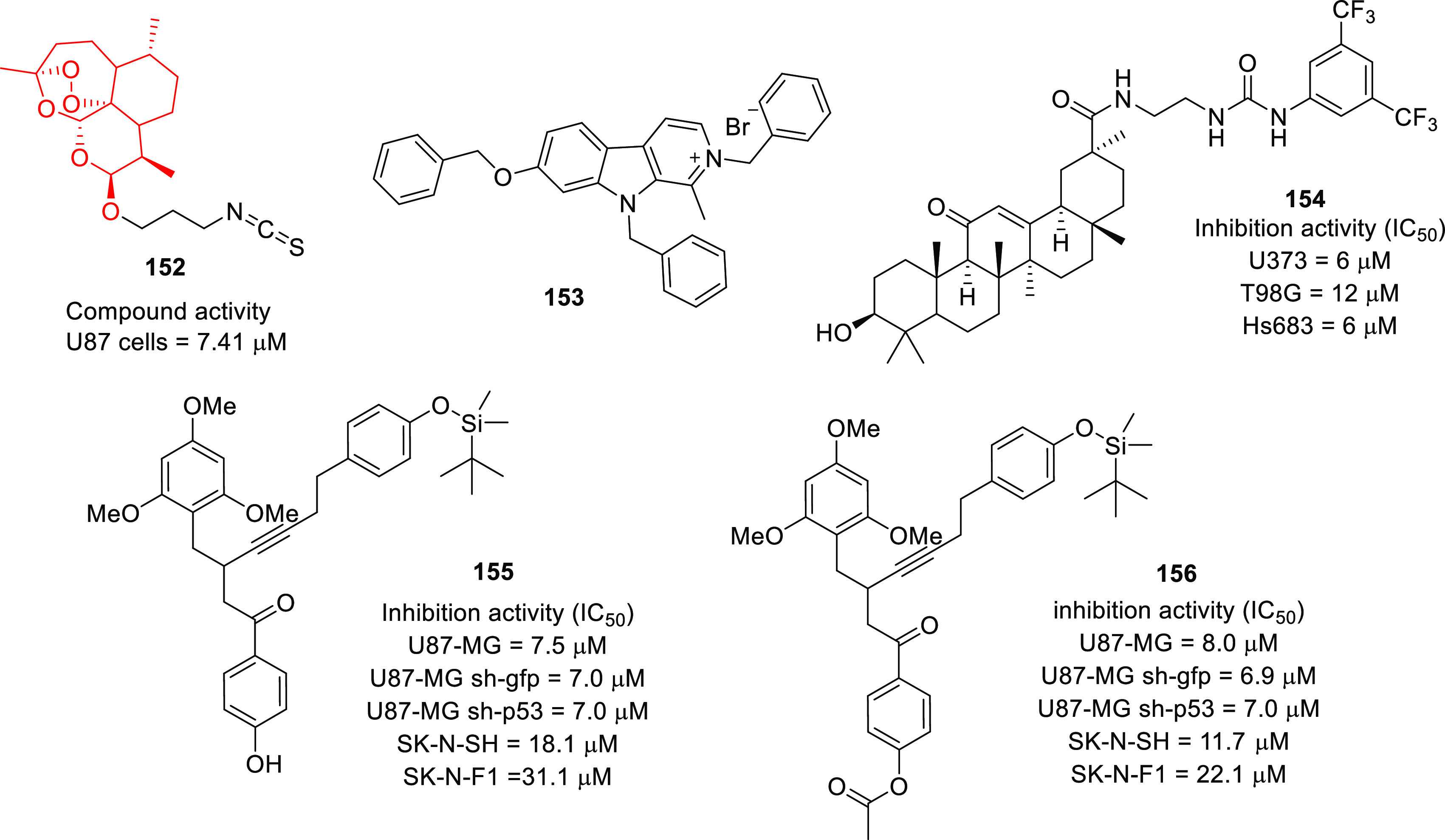
Natural-product-based anti-GBM agents.

In 2018, Nyein et al. disclosed a novel series of artemisinin–isothiocyanate hybrids as potential therapeutics for GBM.^93^ Artemisinin and sulforaphane scaffolds were fused for the molecular hybridization process, and the synthesized compounds were evaluated for *in vitro* anti-tumor effects against the U-87 glioma cell line. Among the synthesized compounds, **152** was the most potent in the series, with IC_50_ = 7.41 μM. Compound **152** was further screened for cell migration using the wound-healing assay, in which it reduced cell migration at 4 μM. Furthermore, **152** triggered apoptosis via caspase family activation, and downregulation was observed in the Bcl-2 protein with BAX upregulation. Additionally, autophagy was induced by **152**, which activated LC3-II and decreased the protein level of p62 (Figure 39). Collectively, artemisinin–isothiocyanate demonstrated promising cell growth inhibitory effects toward the GBM cell line by targeting multiple pathways.

In 2012, Frédérick et al. designed and synthesized a series of trisubstituted harmine derivatives as anti-GBM agents.^94^ Among the synthesized compounds, **153** exhibited remarkable anti-tumor effects against GBM cell lines (U373, T98G, and Hs683) with a mean IC_50_ value of 0.7 μM (Figure 39). Continued investigation of the cellular effects of **153** ascertained that it was cytostatic, with a GGR index of 0.40 μM. Additionally, Western blot analysis of **153** revealed that the compounds downregulated the growth of eukaryotic initiation Factor 2 (eIF-2) in Hs683 and U373 glioma cell lines, suggesting that the compounds possibly acted as protein synthesis inhibitors.^94^

In 2011, Lallemand et al. furnished a series of glycyrrhetinic acid derivatives and identified construct **154** as a promising anti-GBM agent, with IC_50_ = 12 and 16 nM against the T98G and Hs683 oligodendroglioma cell lines, respectively.^95^ Further explorations indicated that **154** inhibited the activity of the proteasome at a concentration (IC_50_) of 7 μM in U373GBM cells (Figure 39). In 2013, Campos et al. designed curcumin-based ligands and synthesized them using metal-catalyzed multi-component reactions.^96^ The synthesized adducts were evaluated for their cell growth inhibitory effects against GBM cell lines (U-87-MG, U-87-MG sh-gfp, and U-87-MG sh-p53) and neuroblastoma cell lines (SK-N-SH and SK-N-F1). Compounds **155** and **156** demonstrated promising cell growth inhibitory effects (Figure 39). Additionally, **155** and **156** were screened in normal hematopoietic progenitor cells, where neither compound produced a noticeable effect.

**Figure 40:**
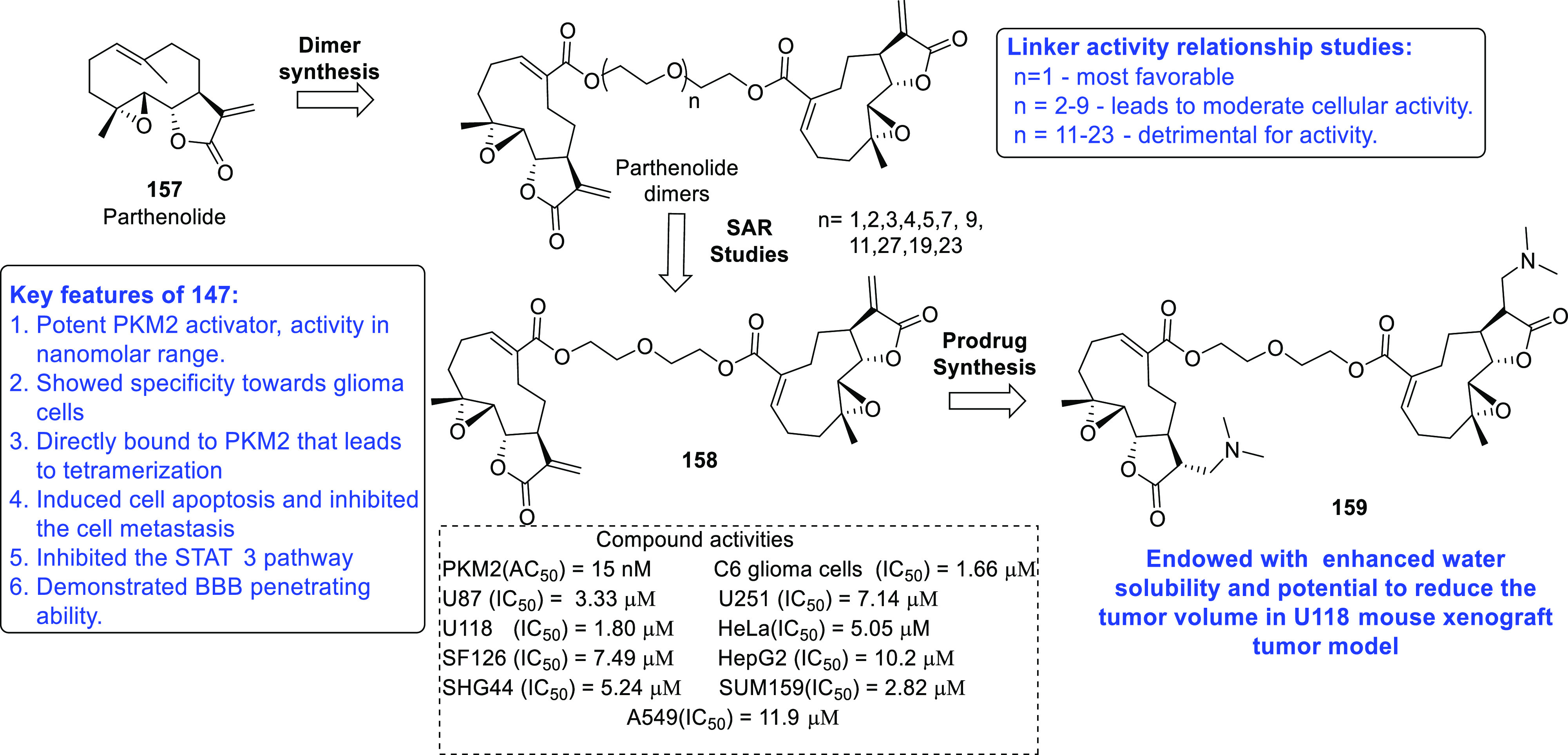
Parthenolide dimer as pyruvate kinase M2 activator.

In 2020, a team led by Xue et al. developed a parthenolide dimer as a pyruvate kinase M2 activator.^97^ The dimeric pyruvate kinase M2 is present in the nucleus of cancer cells and promotes the proliferation, invasion, and metastasis of tumor cells. Activation of PKM2 can promote tetramerization, which decreases glycolytic intermediates and prevents nuclear translocation of dimeric PKM2. Reduced nuclear translocation of dimeric PKM2 may affect cancer cell growth and can be used as an approach against cancer. Previously, the group screened a library of natural compounds as PKM2 activators, where parthenolide **157** displayed moderate PKM2 activation activity. The results motivated the group to synthesize a series of 11 parthenolide dimers as PKM2 activators. Subsequent evaluations led to the identification of **158** inhibiting GBM cell (U-87 and U118) proliferation, inducing cell apoptosis, and inhibiting metastasis in a PKM2 expression-dependent manner by obstructing the STAT3 signaling pathway. Furthermore, *in vivo* studies were performed in the U118 mouse xenograft tumor model. Owing to the low water solubility of **158**, its prodrug (**159**) was synthesized and administered to the mouse at a dose of 50 mg/kg i.p. for 3 weeks. Compound **159** demonstrated remarkable tumor growth inhibitory potential (Figure 40).

**Figure 41:**
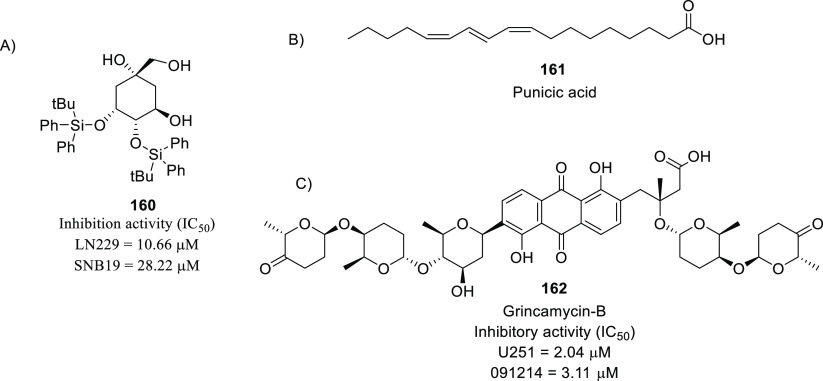
(A) Quinic acid derivatives-based anti-GBM agents. (B) Punicic acid. (C) Grincamycin-B.

Murugesan et al. proposed quinic acid derivatives as potential inhibitors of GBM cells.^98^ A total of 16 quinic acid derivatives were synthesized and evaluated in LN229 and SNB19 cell lines, where **160** showed promising anti-proliferative activity, with IC_50_ = 10.66 and 28.22 μM, respectively. Furthermore, **160** was formulated into nanoparticles, which showed a similar effect as **160** alone, and biological studies confirmed that **160** induces apoptosis through the ROS-mediated pathway and caspase 3/7 (Figure 41A). In 2019, Mete et al. evaluated punicic acid (PA), a polyunsaturated fatty acid obtained from pomegranate seed oil, against the GBM cell line.^99^ PA (**161**) showed potential cell growth inhibition over the T98 GBM cell line, and the IC_50_ dose was found to be 9.85 μL/mL. Additionally, **161** inhibited cancer cell migration and induced apoptosis by inhibiting the PAM signaling pathway. Overall, PA (**161**) showed impactful results against GBM cell lines and can be used in combination with other anti-GBM drugs (Figure 41B). In 2020, Yao et al. screened the anti-cancer potential of grincamycin-B (**162**), a marine natural product, against GBM cell lines.^100^ Grincamycin-B (**162**) showed potential cell inhibition against the U251 and 091214 GBM cell lines, with IC_50_ = 2.04 ± 0.24 and 3.11 ± 0.25 μM, respectively. Further biological evaluation revealed that grincamycin-B (**162**) targets CSCs in GBM by targeting the RHOA and PI3K/Akt signaling pathways (Figure 41C).

### 4.11 Imaging Tools/Chemical Probes for GBM

**Figure 42:**
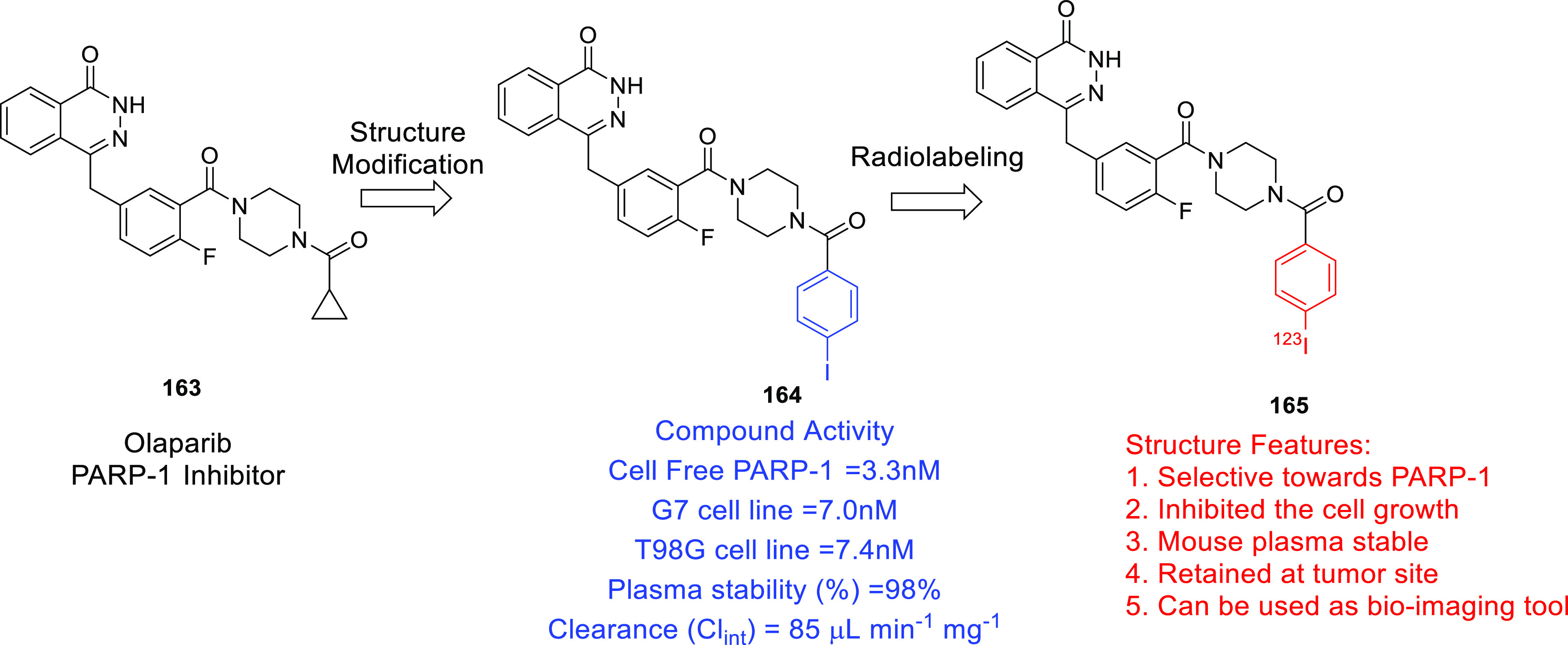
Radiolabeled olaparib as bioimaging tool for glioma detection.

In 2015, Zmuda et al. introduced novel radio-iodinated tracers with specificity to PARP-1 for GBM imaging.^101^ Olaparib (**163**), a clinical PARP-1 inhibitor, was used as a lead, and a series of olaparib analogs were synthesized bearing various substitutions. Initially, all the synthesized compounds were evaluated using the PARP-1 inhibition assay, lipophilicity (LogP_oct_), and percentage plasma protein binding (%PPB) by high-performance liquid chromatography; all the synthesized compounds exhibited promising results. Among the synthesized compounds, **164** showed the highest activity, with a cell-free PARP-1 inhibition IC_50_ value of 3.3 nM, lipophilic properties (LogP_oct_) of 3.0, and a percentage of plasma protein binding (%PPB) of 96% (Figure 42). The lead compound **164** was further assessed over primary G7 and established T98G human GBM cell lines, where it displayed potential cell growth inhibition with IC_50_ = 7.0 and 7.4 nM with plasma stability of 98% and intrinsic clearance (CL_int_) of 85 μL min^–1^ mg^–1^, respectively. The lead compound was next labeled with ^125^I (**165**), and the *ex vivo* biodistribution was evaluated in nude mice in a human GBM xenograft model. *Ex vivo* studies revealed that radiotracer **165** specifically binds to PARP-1 and is retained at the tumor site. The overall results indicated that **165** displayed good *in vivo* properties to serve as a promising imaging tool in GBM surgeries.

**Figure 43:**
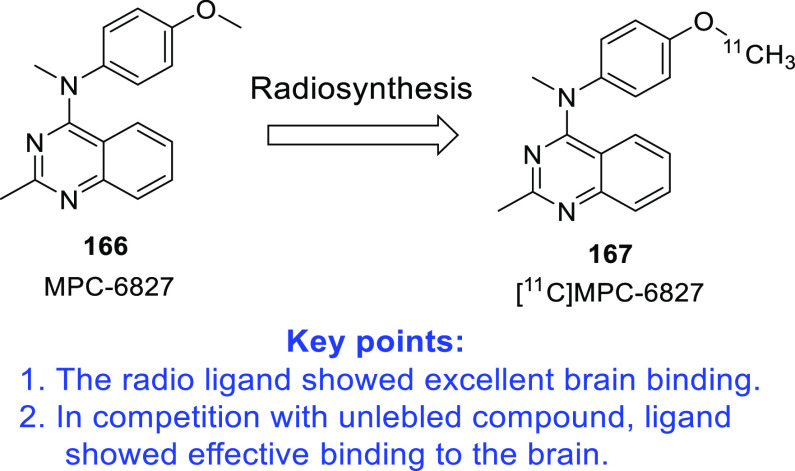
[^11^C] MPC-6627 radio ligand for GBM imaging.

In 2018, Kumar et al. introduced the first microtubule positron emission tomography (PET) radioligand, [^11^C]MPC-6827, with brain-penetrating ability properties to detect GBM.^102^ Compound **166** (MPC-6827), a microtubule inhibitor (IC_50_ = 1.5 nM), was first evaluated over various brain targets, where the compound demonstrated targeting against the histamine-4 receptor and sigma-1 receptor with *K*_i_ = 155 and 426 nM, respectively (Figure 43). Furthermore, adduct **167** ([^11^C]MPC-6827) was synthesized, and *in vivo* binding was evaluated in white male mice. The compound retained its peak in the brain for 5 min and was gradually washed out, indicating desirable kinetics. Additionally, **167** showed 70% brain accumulation followed by accumulation in the muscles, spleen, and lungs with specific binding of 60%, 42%, and 30% at a dose of 5 mg/kg i.v., respectively. Notably, the *in vivo* binding of [^11^C]MPC-6827 (**167**) was evaluated with unlabeled MPC-6827, and the compound showed specific binding and retention in the brain, confirming its potential as a bioimaging tool.

**Figure 44:**
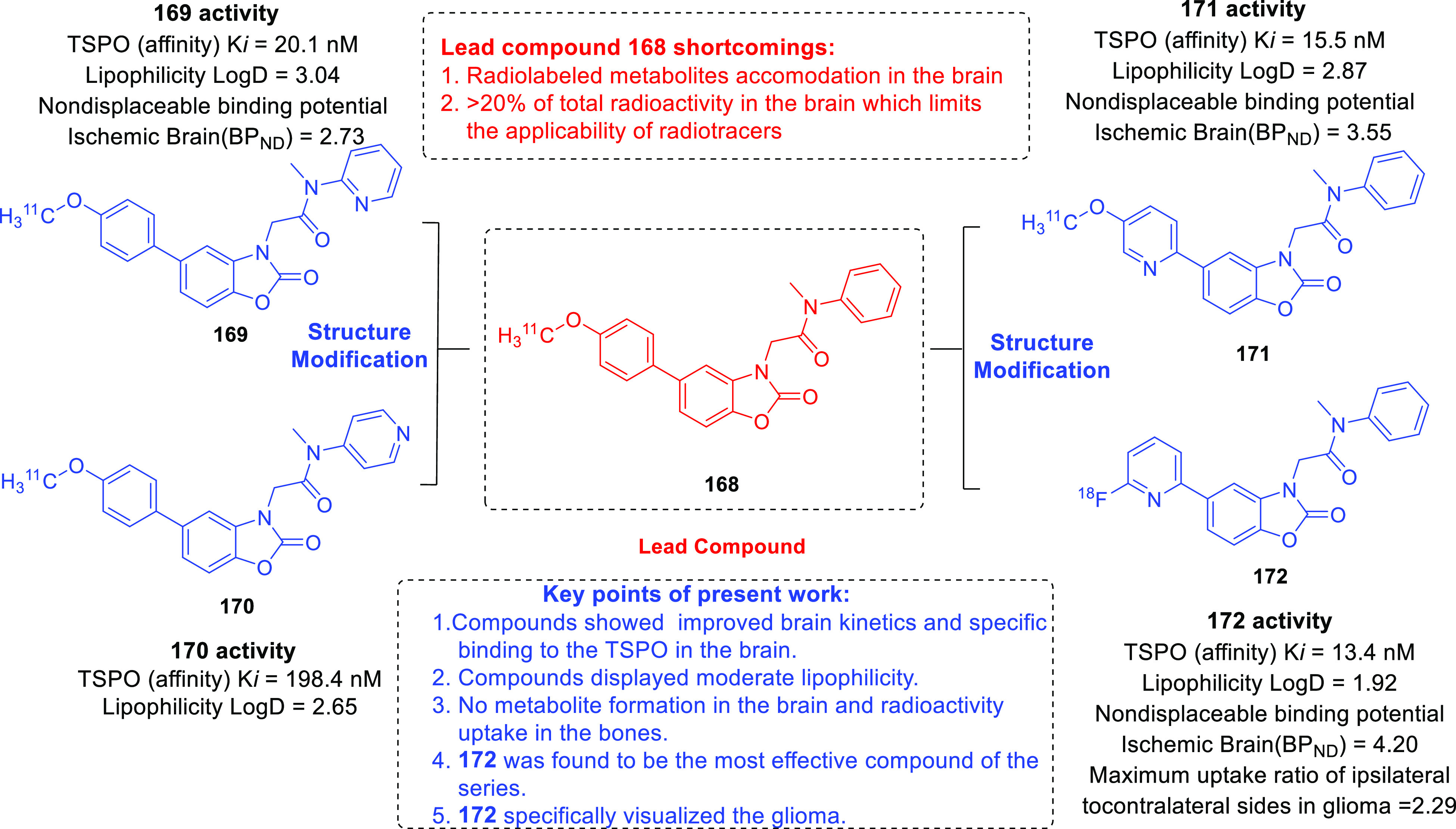
TSPO radiotracers for ischemic brain and glioma.

In 2017, Fujinaga et al. developed ^18^F-labeled radiotracers for PET imaging to visualize TSPO S in ischemic brains and gliomas.^103^ Many PET tracers for TSPO have been reported that display high *in vitro* affinity; however, most of the compounds exhibit low *in vivo* specific binding and slow brain kinetics in the human brain. Considering the above findings, the group designed and synthesized four new unlabeled and [^11^C],[^18^F]-labeled acetamido benzoxazolone analogs using **168** as a lead compound (Figure 44). Initially, the *in vitro* binding affinity (*K*_i_) of the synthesized compounds was evaluated for TSPO in the rat brain by assaying competitive binding, where **169**, **171**, and **172** showed a high binding affinity with *K*_i_ = 20.1, 15.5, and 13.4 nM, respectively. The lipophilicity values of the compounds were in the range of 2.35–3.00. The *in vitro* and *in vivo* specific binding of [^11^C]-labeled **168**–**171** and [^18^F]-labeled **172** for TSPO was investigated in the ischemic rat brain, where the compounds displayed radioactivity on the ipsilateral side compared with the contralateral side with average binding concentrations of 12.0, 1.2, 21.6, and 29.8, respectively. PET imaging displayed a higher uptake of radioactivity on the ipsilateral sides of the brain with standard uptake values as follows: **169**, 1.72; **171**, 0.98; and **172**, 1.70. Additionally, displacement studies using unlabeled and labeled **169**–**172** revealed that the labeled compounds were highly specific for TSPO in the ischemic brain. Furthermore, the biodistribution of **172** was investigated in the bones and whole body of mice, and the results indicated high uptake of radioactivity in the lungs, heart, and kidneys, moderate uptake in the small intestine, muscle, liver, spleen, and testis, and no significant activity in the bones. The radiolabeled metabolite accommodation in the brain was investigated by HPLC, indicating that **172** was metabolized into a single metabolite that was not accommodated in the brain for a long time. Finally, PET imaging studies of **172** were performed in a rat model bearing C6 glioma cells in the brain. The images displayed good accommodation of the compound at the tumor site, while the radioactivity was rapidly cleared from the contralateral side, suggesting that the radiotracer specifically targets the GBM. Overall, **172** demonstrated sufficient promise to emerge as a potential chemical tool to trace inflammation and GBM.

**Figure 45:**
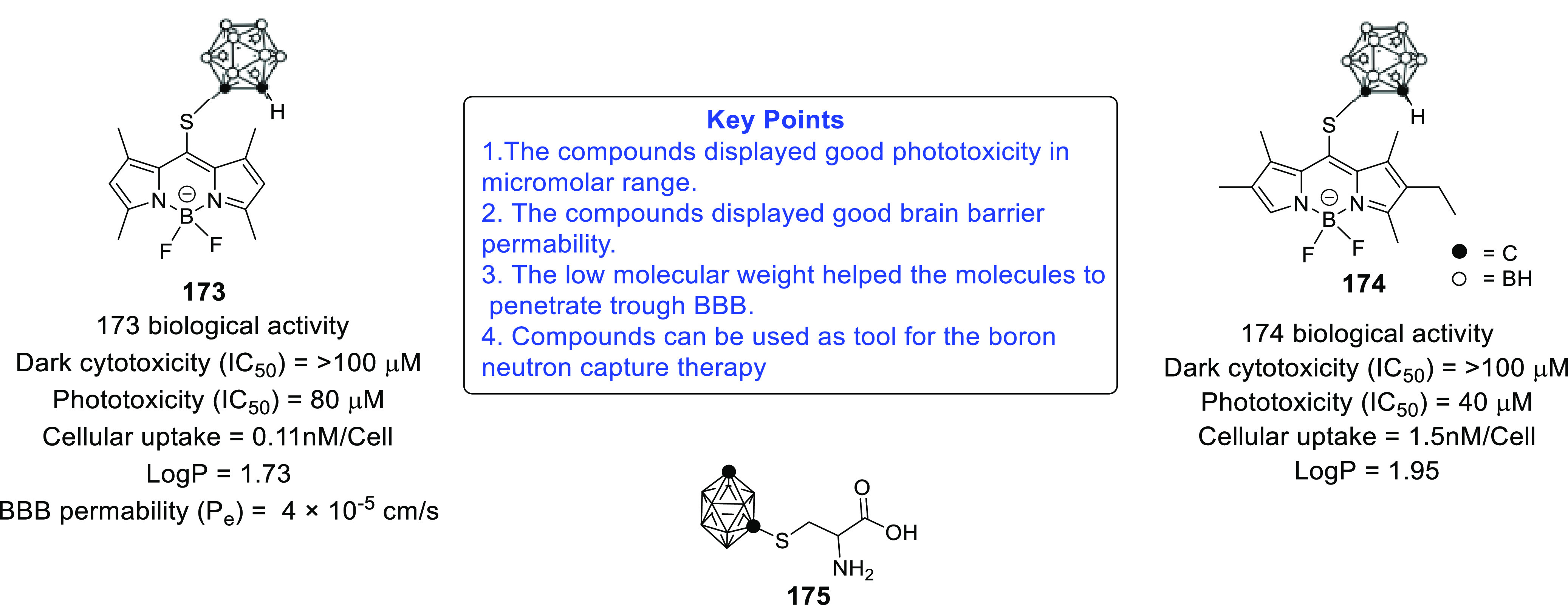
Carborane-containing boron dipyrromethenes (BODIPYs) as probes for the boron neutron capture therapy.

In 2016, Xuan et al. synthesized a series of carborane-containing boron dipyrromethenes (BODIPYs) as probes for boron neutron capture therapy.^104^ A total of seven compounds with molecular weights of 366–527 Da and LogP = 1.5–2.7 were synthesized from the corresponding 2,6-diiodo-substituted compounds using Suzuki and Sonogashira coupling reactions. All the synthesized compounds showed no dark cytotoxicity in T98G GBM cells; however, **173** and **174** displayed good phototoxicity, with IC_50_ = 80 and 40 μM, respectively. The cell uptake values for **173** and **174** were 0.11 and 1.5 nM/cell with LogP = 1.73 and 1.95, respectively. The subcellular localization sites of the compounds were determined in the Hep2 cell line using fluorescence microscopy, revealing that the compounds were localized primarily in the endoplasmic reticulum (Figure 45). Furthermore, the BBB permeability of the synthesized compounds was evaluated in the hCMEC/D3 cell line. All the compounds showed lower permeability than *P*_e_ = 3 × 10^–6^ cm/s; however, **174** showed good BBB permeability, with *P*_e_ = 4 × 10^–5^ cm/s. Altogether, carborane-containing boron dipyrromethenes (BODIPYs) showed promising results in initial evaluations, and further modifications and evaluations are required to establish them as useful candidate probes for boron neutron capture therapy. In 2019, He et al. incorporated *m*-carborane into the amino acid cystine (**175**), and its activity was observed in the U-87 GBM cell line.^105^ It was found that **175** was rapidly taken up by U-87 cells and showed a reduction in cell viability in a dose-dependent manner. Moreover, **175** targeted CDKs and other genes associated with the cell cycle triggered cell death at S phase, which was further validated with qPCR studies (Figure 45).

**Figure 46:**
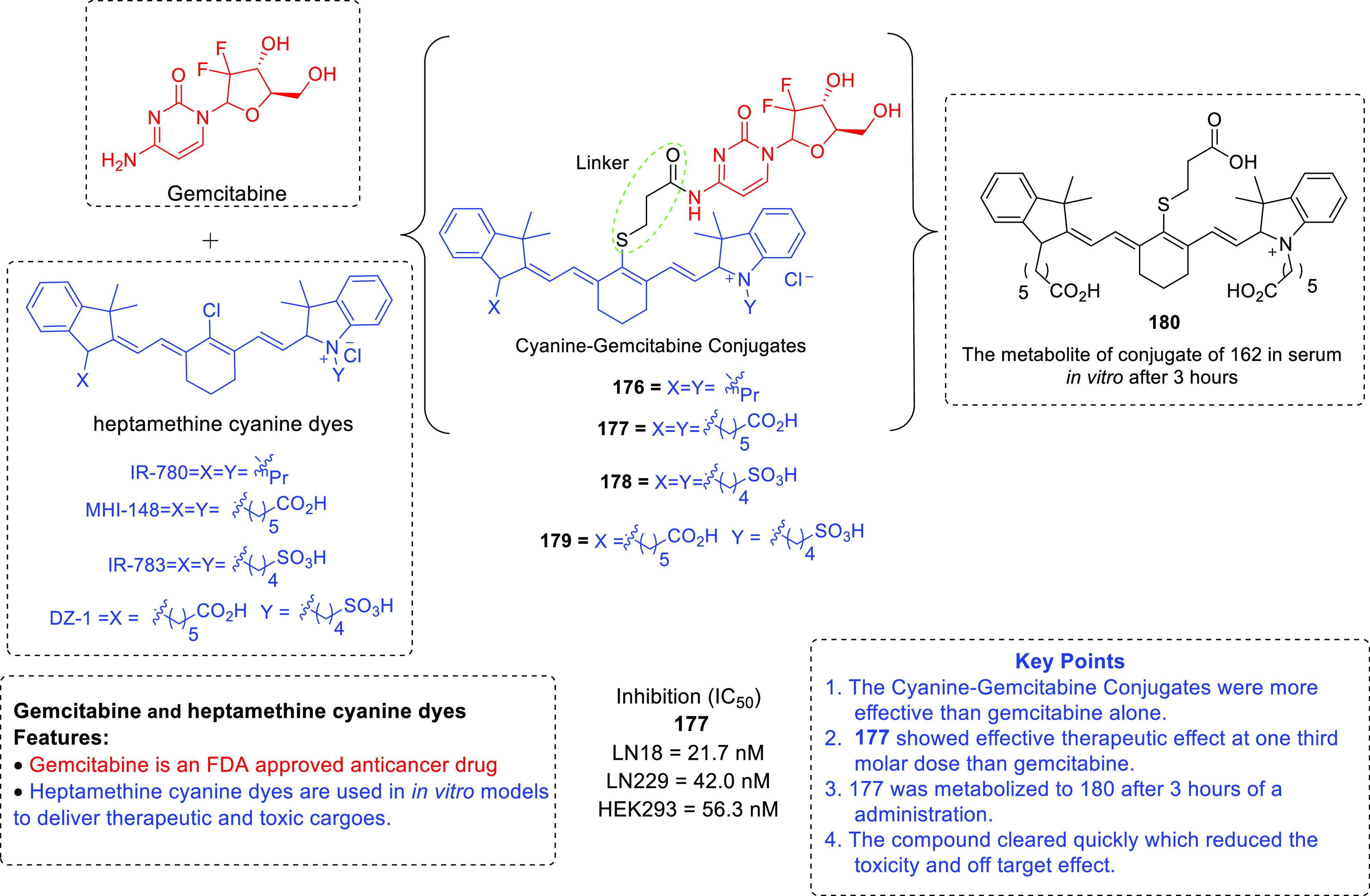
Cyanine–gemcitabine conjugates as targeted theranostic agents.

In 2019, Jiang et al. investigated cyanine–gemcitabine conjugates as targeted theranostic agents against GBM tumor cells.^106^ Gemcitabine is a well-established drug against a wide range of solid tumors and has been approved for the treatment of breast, non-small-cell lung, ovarian, and pancreatic cancers. Gemcitabine has also been investigated for GBM; however, the therapeutic response was not promising, and it was deduced that the low BBB permeability, short half-life because of enzyme metabolism, and selectivity of gemcitabine toward tumor versus healthy tissues must be improved. The literature precedents reveal the ability of heptamethine dyes (cyanine-7 or Cy7) to preferentially accumulate and retain tumors and their use in *in vivo* tumor models to deliver therapeutic and toxic cargoes. To overcome the limitations of gemcitabine, the group designed four cyanine–gemcitabine conjugates, **176**–**179**, which locally aggregate to the tumor and achieve the desired therapeutic value. Structural engineering attempts (Figure 46) revealed that replacement of the exocyclic amine group from gemcitabine does not affect the activity of the drug, whereas replacement of the chloro group from heptamethine dyes with various substitutions prolonged the half-life. The cytotoxicity of all the compounds was evaluated against U-87 cells, revealing that the compounds displayed substantial cytotoxicity (IC_50_) within the range of 0.01–0.02 μM. The effects of **177** and gemcitabine were also evaluated in LN18, LN229, and HEK293 cell lines, and the results were overwhelmingly positive because adduct **177** displayed a magnificent anti-tumor profile (Figure 46). Furthermore, *in vivo* studies were performed in a mouse model xenograft model bearing U-87 glioma cells, where both **177** and the drug significantly reduced tumor growth. Notably, **177** was more effective and achieved a therapeutic effect at a one-third molar dose than gemcitabine. Additionally, drug localization was monitored by fluorescence imaging of tumors, which showed that the **177** conjugate cleared from the mice within 24 h. Thus, the synthesized conjugate **177** was effective against GBM and demonstrated the potential to achieve a therapeutic effect and overcome the barriers associated with using gemcitabine.

### 4.12 Miscellaneous

**Figure 47:**
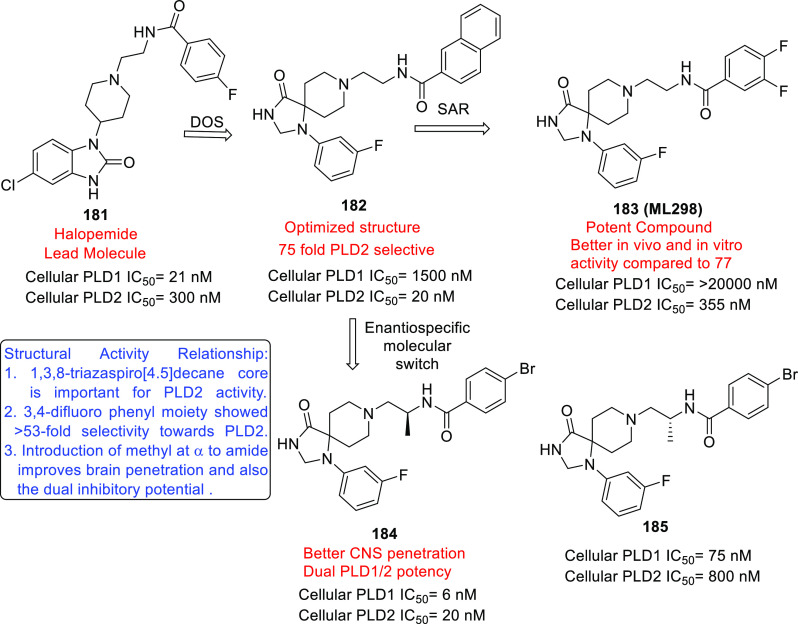
PLD targeting agents for the treatment of GBM.

In 2013, O’Reilly and colleagues developed a series of dual PLD1/2 and PLD2 (phospholipase D) selective inhibitors.^107^ They performed diversity-oriented synthesis (DOS) of halopemide (**181**), a classical atypical anti-psychotic agent, which showed a direct and potent dual PLD1/2 inhibitory effect (PLD1 IC_50_ = 21 nM; PLD2 IC_50_ = 300 nM). The above approach identified a PLD inhibitor (**182**) that elicited pronounced selectivity (75-fold higher selective inhibition) toward PLD2. The 1,3,8-triazaspiro[4.5]decane core was identified as a PLD2-preferring motif. Keeping the 3-fluorophenyl moiety of **182** constant, different amides were explored to improve the PLD2 selectivity of the compounds. Compound **183** (ML298), bearing a 3,4-difluorophenyl moiety, was identified with >53-fold selectivity toward PLD2. Furthermore, as a part of structural optimization, the team introduced a chiral methyl group at the α position to the amide group, leading to the synthesis of **184** and **185**. The (*S*)-enantiomer (**184**) was found to be the most potent compound of the series, showing dual potency toward PLD1 (IC_50_ = 6 nM) and PLD2 (IC_50_ = 20 nM). Notably, the results of the cell-based assays using U-87-MG cells revealed that both **183** and **184** cells dose-dependently decreased invasive migration in U-87-MG GBM cells (Figure 47).

**Figure 48:**
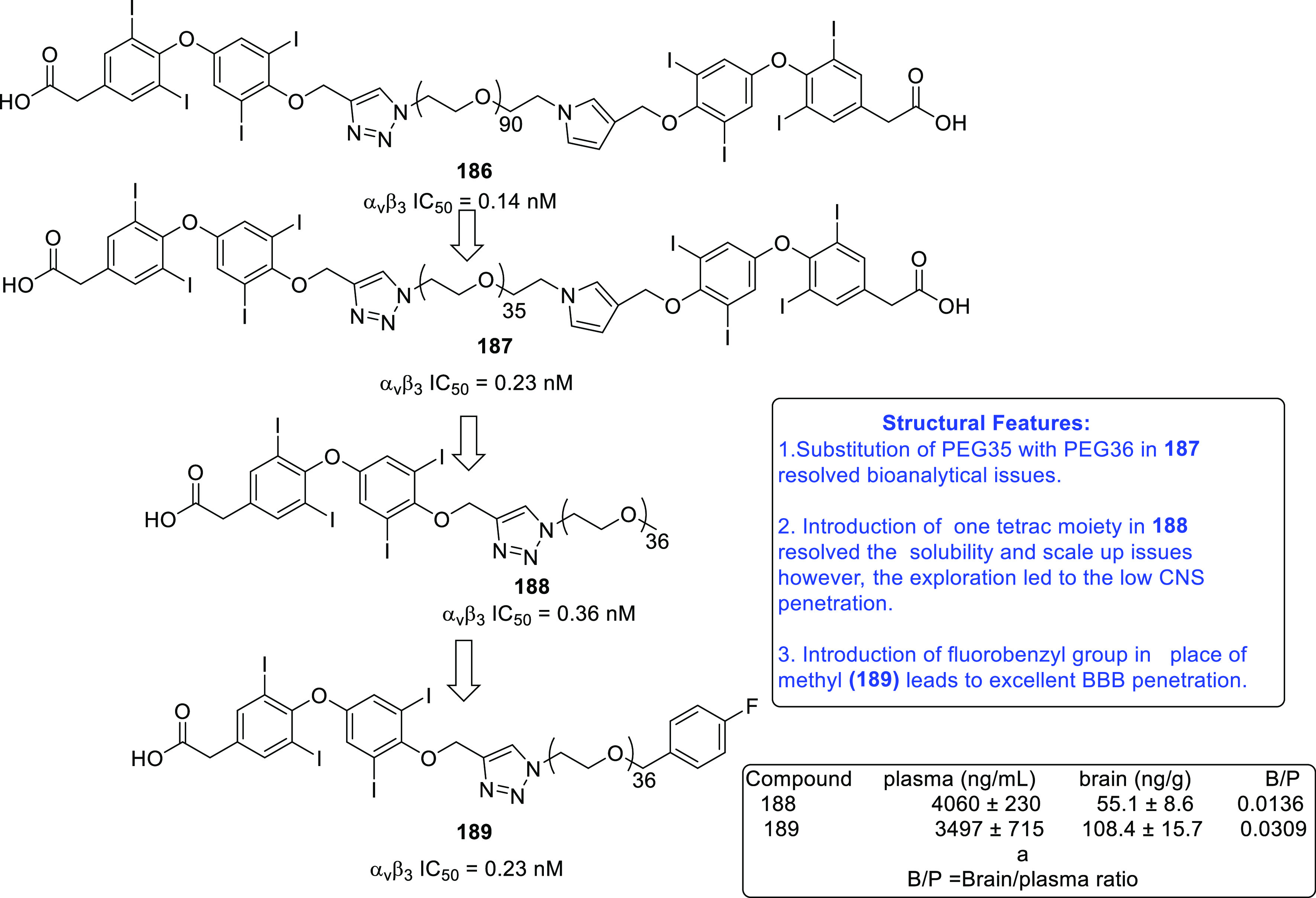
Thyrointegrin α_v_β_3_ atagonists as effective tools against GBM.

In 2021, Bruce and colleagues synthesized new thyrointegrin α_v_β_3_ antagonists as anti-glioma agents (Figure 48). They previously reported compound P-bi-TAT (**186**), a conjugate of tetraiodothyroacetic acid and polyethylene glycol (PEG) 4000, as an efficient agent in a GBM mouse model. They synthesized a smaller and monodisperse PEG36 derivative **187** that exhibited integrin α_v_β_3_ binding affinity; however, its detailed investigation was blocked due to low aqueous solubility. To circumvent this issue, **188** was designed and demonstrated remarkably higher aqueous solubility (>120 mg/mL) than **187** (1.4 mg/mL). Further investigation (PK studies) revealed that **188** could not cross the BBB, likely due to its deliberately decreased lipophilicity to improve the aqueous solubility. Considering the above findings, another compound (**189**) bearing a fluorobenzyl group was furnished that demonstrated significant α_v_β_3_ binding ability (0.23 nM) coupled with good aqueous solubility (120 mg/mL) and BBB permeability. **189** displayed substantial *in vivo* anti-tumor potential because it led to a reduced GBM tumor size with a maximum loss of 98% of the tumor following 21 days of administration at a dose of 10 mg/kg. Fluorescence dye labeling studies also indicated that **189** easily crosses the BBB and localizes to GBM brain tumor tissue compared with normal brain tissue (Figure 48).^108^

**Figure 49:**
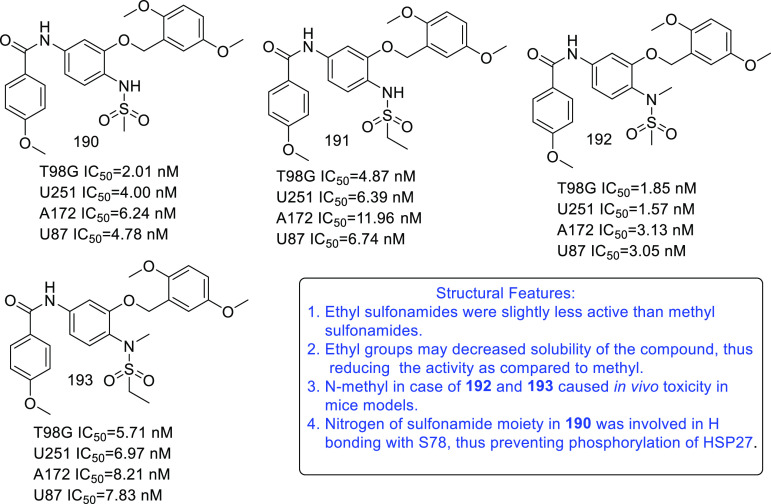
Nimesulide analouges as potential anti-GBM agents.

In 2021, Li et al. investigated four nimesulide analogs to treat GBM.^109^ In their previous studies, these four compounds were identified as dual HSP27 and tubulin inhibitors. In the present study, the authors planned to evaluate the ability of the compounds to modulate androgen receptor function in GBM cells. Compounds **190**–**193** were tested against four GBM cell lines, and they showed relatively better inhibitory effects toward T98G cells (Figure 49). Because T98G cells express higher concentrations of androgen receptors, the selectivity of the compounds is related to their modulatory potential of androgen receptor expression. Furthermore, the findings of the *in vivo* toxicity studies revealed that **190** and **191** were devoid of toxicity. For the mechanistic studies, **190** was selected to investigate the molecular mechanisms responsible for its efficacy in androgen receptor-overexpressing GBM cells, and the results confirmed the HSP27 inhibitory activity of **190**. Molecular docking studies showed that the chemical architecture of **190 (**nitrogen from the sulfonamide moiety) was involved in hydrogen bonding interactions with serine residue S73. Additionally, **190** suppressed androgen receptor transcription, induced degradation of androgen receptors in tumor tissues, and elicited remarkable tumor growth inhibitory effects in a U-87 xenograft model.

**Figure 50:**
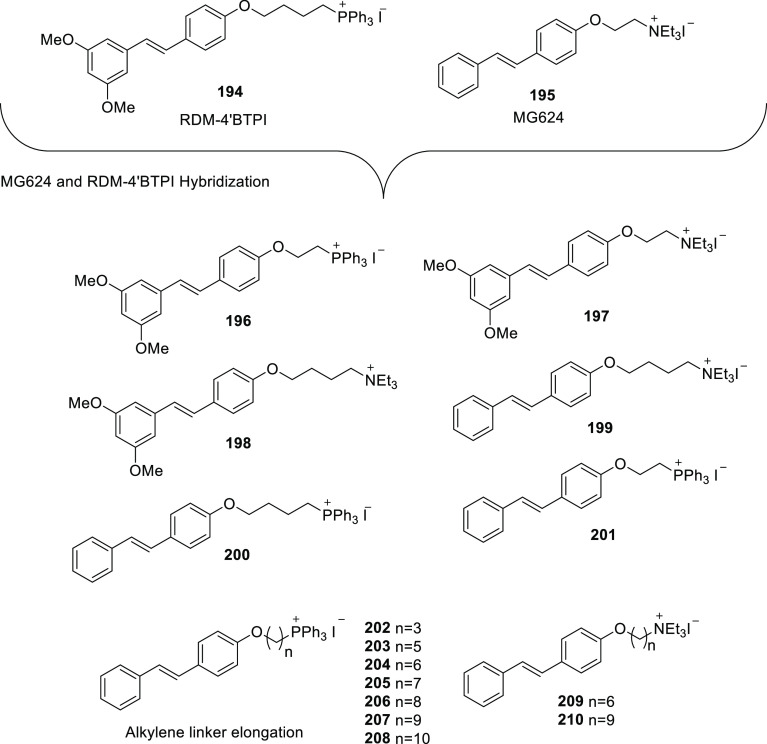
α7-nAChR and α9-nAChR antagonists for the treatment of glioma.

The literature has revealed that α7- and α9α10-containing nicotinic acetylcholine receptors (nAChRs) are highly expressed in GBM cell lines. Considering these revelations, Pallavicini et al. synthesized potent anti-GBM hybrid scaffolds via fusion of the pro-oxidant mitocan agent RDM-4′ BTPI (**194**) and antagonist of the α7 and α9α10 nAChR agent MG624 (**195**).^110^ The structural template of the designed hybrids comprised three structural elements: a stilbene core, an alkylene linker, and a terminal onium. All the compounds were evaluated for their *in vitro* functional activity on α7 and α9α10, nAChR subtypes, demonstrating the potency of all the ammonium-based compounds in inhibiting 10 μM or 200 μM acetylcholine (ACh)-induced currents in oocytes expressing the human α7 and α9α10 subtypes. Furthermore, the authors evaluated the cell growth inhibitory effects of the compounds toward U-87MG glioblastoma, A549 adenocarcinoma, SH-SY5Y neuroblastoma, and wild-type mouse astrocytes on all nine compounds (the six ammonium compounds **195**, **197**, **198**, **199**, **209**, and **210** and the three phosphonium compounds **194**, **200**, and **208**). The MTT assay demonstrated that **197** and **198** were selectively toxic against GBM cells. Notably, the enhanced anti-GBM activity of ammonium-based hybrid **199** coincided with greater antagonism against the α7 and α9α10 subtypes. Additionally, the ability of all nine compounds to interfere with ATP production was evaluated by incubating them with U-87MG cells for 1 or 72 h, where **194**, **200**, **208**, and **209** significantly reduced ATP production after only 1 h of incubation (Figure 50).

**Figure 51:**
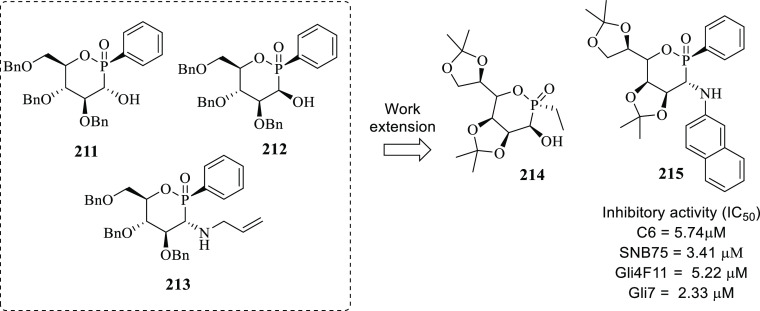
Oxaphosphinanes as anti-GBM agents.

In 2012, Clarion et al. reported a series of new oxaphosphinanes as anti-GBM agents.^111^ A total of 26 compounds were synthesized, and their cancer cell growth inhibition potential was evaluated against the C6 rat GBM cell line using the MTT assay. All the screened compounds displayed promising activity, and **211**–**213** were the best inhibitors of the series, with EC_50_ = 0.52, 23.81, and 0.49 μM, respectively (Figure 51). In 2014, the same group extended the work and introduced a new series of d-glycero-d-talo- and d-glycero-d-galactopyranose analogs (C-glycoside mimetics) as proliferation, migration, and invasion inhibitors of GSCs.^112^ Among the synthesized compounds, 10 compounds showed adequate inhibitory efficacy with IC_50_ < 10 μM toward GSCs (Gli4 and Gli7) and GBM cell lines (SNB75 and C6). Additionally, two compounds, **214** and **215**, were exhaustively investigated, revealing overwhelmingly positive results. Specifically, **214** was the most promising because it manifested significant effects against GLI4 and GLI7 cell lines, inhibited cell invasion, and targeted CNS cancer cells without affecting normal astrocyte and cortical neuron survival (shown in Figure 51).

**Figure 52:**
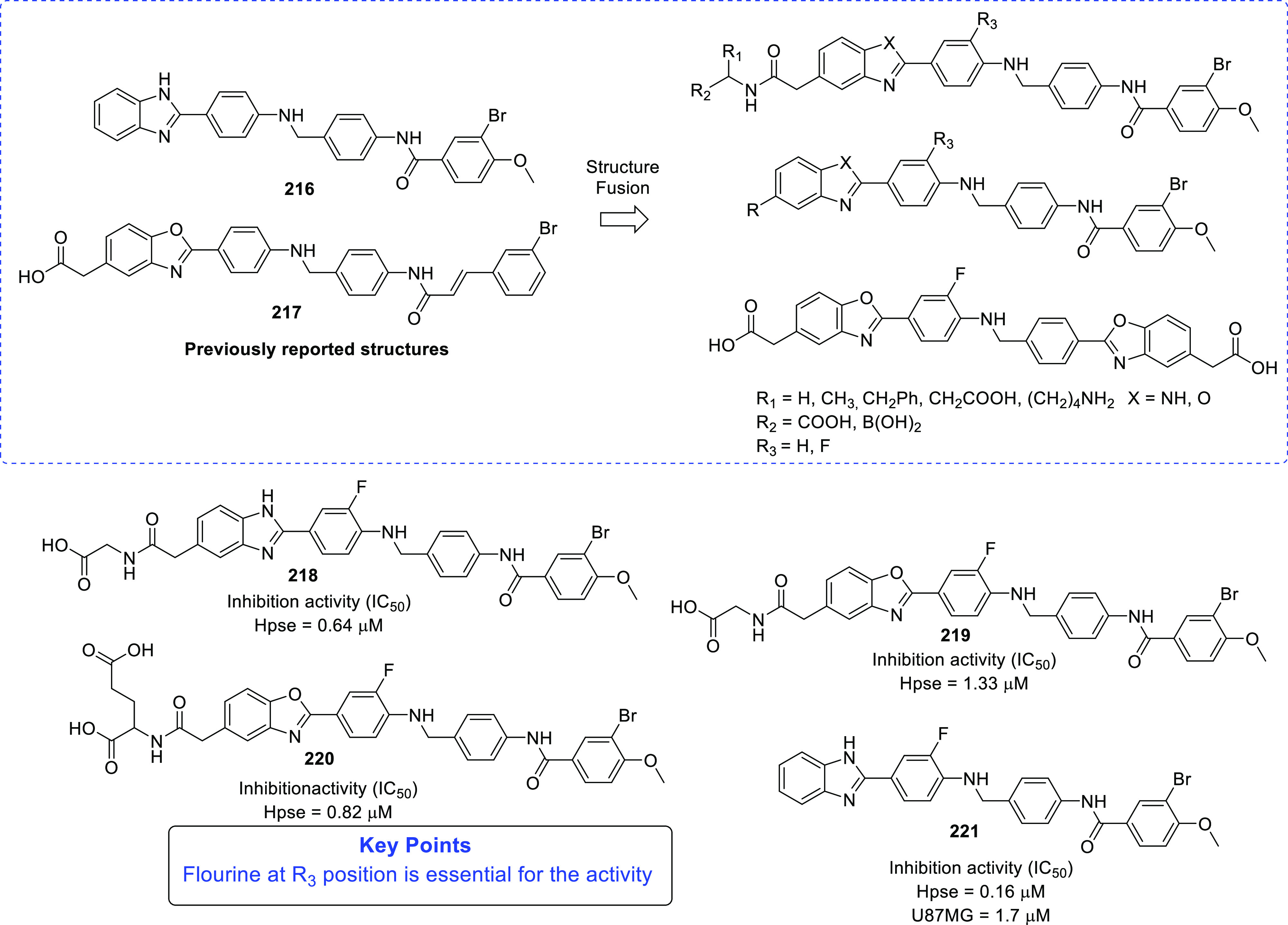
Benzazole derivatives as potent anti-heparanase agents.

In 2018, Madia et al. reported novel benzazole derivatives as potent anti-heparanase agents.^113^ The previously reported **216** and **217** were used as leads, and the planned modifications led to three series of compounds (Figure 52). All the synthesized compounds were evaluated for Hpse inhibitory activity. Among them, **218**–**221** were the most potent in the series, with IC_50_ = 0.64, 1.33, 0.82, and 0.16 μM, respectively. Computational studies were performed to determine the binding interactions responsible for Hpse inhibition, and the crystal structure of Hpse with PDB ID 5E9C was used to model the ligands with the binding pocket. In the model, **218** displayed interactions with Q270 and R272, and **220** formed a hydrogen bond with R272 and N227. The benzamide moiety of the structures was involved in interactions with the G350, A388, N390, and Y391 residues of the binding pocket. Furthermore, the anti-proliferative activity of the most potent compounds in series **218**–**221** was evaluated against U-87MG (glioma) cell lines along with other human cancer cell lines. The results led to the identification of **221** as a potent anti-proliferative compound because it exerted significant inhibitory effects on the U-87MG cell lines, with IC_50_ = 1.7 μM. Additionally, in the Matrigel invasion assay, **221** was substantially active against U-87MG cells in the context of the inhibitory potential.

**Figure 53:**
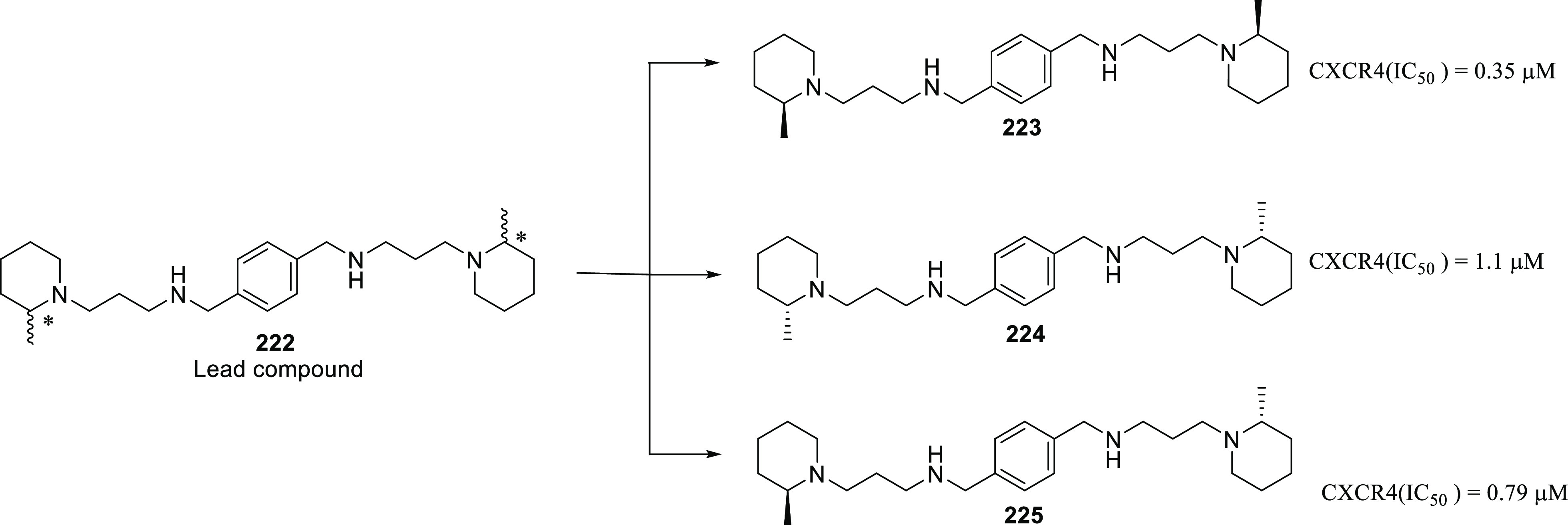
Non-cyclam tetraamines as CXCR4 inhibitors.

In 2012, a team led by Laia Ros-Blanco reported non-cyclam tetraamines that inhibited the type 4 CXC chemokine receptor and glioma-initiating cells.^114^ CXCR-4 is a transmembrane receptor that regulates various cell types, including CSCs. The group used their previously synthesized compound **222** as a lead that was reported as a potent HIV-1 entry CXCR4 co-receptor inhibitor targeting CXCR4 co-receptors without cytotoxicity. A total of three compounds were synthesized, and affinity toward the CXCR4 receptors employing a conventional K^+^ channel patch-clamp assay was evaluated. Compounds **223**–**225** displayed good affinity, with IC_50_ = 0.35, 1.1, and 0.79 μM. In the toxicity studies, the maximum non-lethal doses for **223**–**225** were 2.0, 1.5, and 2.5 mg/kg, while the minimum lethal doses were 2.5, 2.0, and 3.0 mg/kg, respectively. The compounds were then evaluated against glioma-initiating cells by monitoring the level of CD44+; all the compounds decreased the level of CD44+. The results were further confirmed by *in vivo* experiments in the brains of NOD-SCID mice, where compounds decreased the number of glioma-initiating cells (Figure 53).

**Figure 54:**
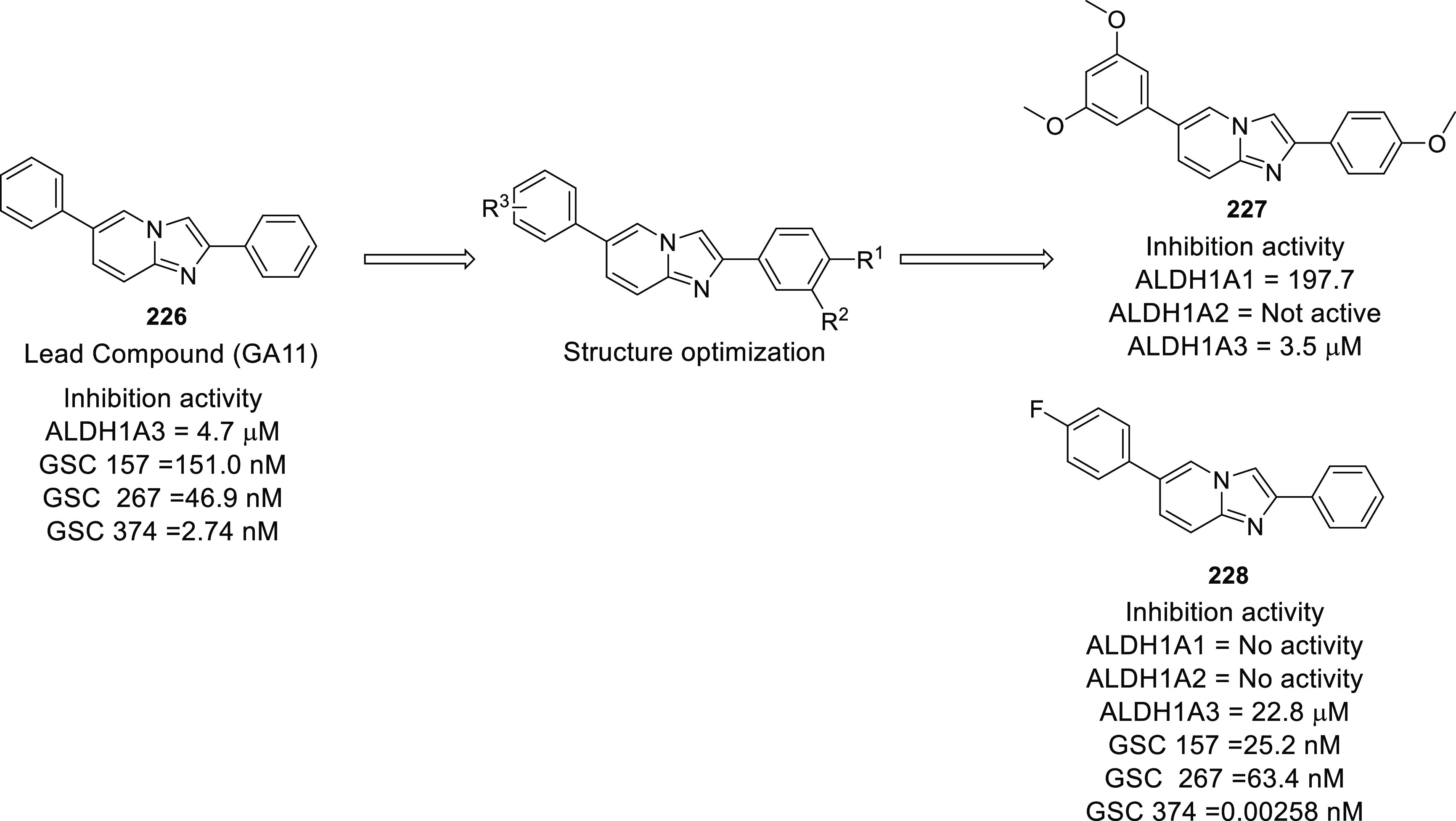
Aldehyde dehydrogenase inhibitors against GBM.

Quattrini et al. reported some novel chemotypes as aldehyde dehydrogenase inhibitors to treat GBM.^115^ The group used the previously reported potent aldehyde dehydrogenase inhibitor **226** GA11 (ALDH1A3, IC_50_ = 4.7 ± 1.7 μM, *K*_i_ = 0.54 ± 0.11 μM) as a lead and optimized the second and sixth positions of the phenyl ring of the heterocyclic core. A series of compounds was synthesized and evaluated against various aldehyde dehydrogenases, where all the synthesized compounds displayed inhibition activity in the low micromolar range. Among the synthesized compounds, **228** displayed selectivity toward ALDH1A3, with IC_50_ = 22.8 μM, while **227** was the most potent in the series, with IC_50_ = 3.5 μM (Figure 54). The co-crystal structure of **227** with ALDH1A3 revealed that the oxygen atom on the 2-phenyl ring and the methoxy group on the 6-phenyl ring formed hydrogen bonds with the Q304, W189, and T140 residues of the binding pocket. The compound displayed additional π–π stacking with E135 and Y472 residues, and the 6-phenyl ring established hydrophobic contact with the protein backbone. Furthermore, the anti-proliferative activity of the selected compounds was evaluated in the GSC 157, 267, and 374 cell lines, and **228** was found to be the most potent, with IC_50_ = 25.2, 63.4, and 0.00258 nM, respectively.

**Figure 55:**
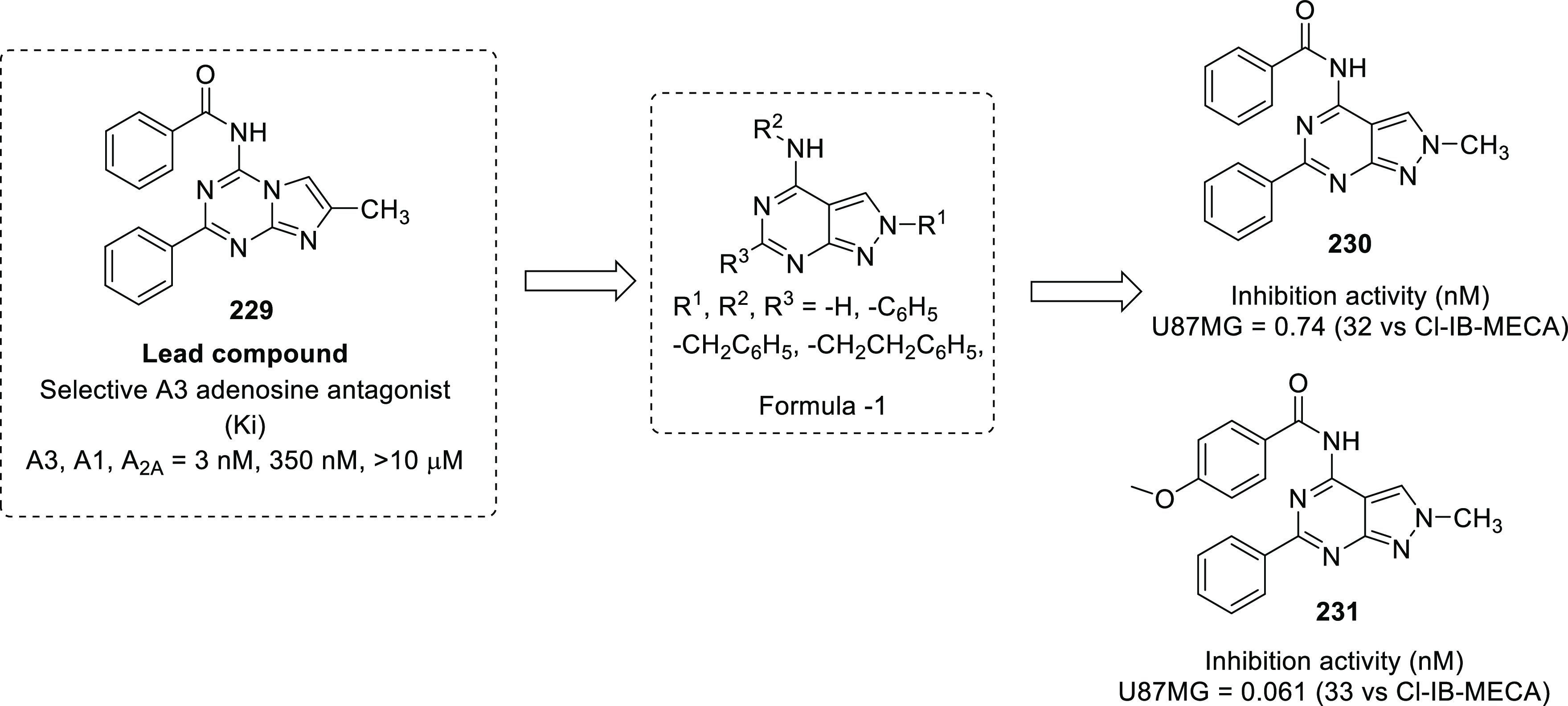
Adenosine A3 receptor antagonists against GBM.

In 2010, Taliani et al. reported novel *N*^2^-substituted pyrazolo[3,4-*d*]pyrimidine as an adenosine A3 receptor antagonist.^116^ The group used the previously reported compound **229** (a selective A3 inhibitor) as a lead and designed a series of A3 adenosine receptor antagonists. The anti-proliferative properties of the compounds were investigated in the U-87MG cell line, and **230** and **231** exhibited striking anti-proliferative activity, with IC_50_ = 0.74 and 0.061 nM, respectively. Additionally, docking studies were performed for the A_3_ receptor using AudoDock-4, revealing that **231** binds to the outer portion surrounded by TMs III, V, VI, and VII helices. The methyl group at R_2_ interacted with the L246 residue, and the pyrazolopyrimidine formed π-stacking with F168 with additional H-bonding with N250. The 6-phenyl ring interacted with residues I186, L91, W243, L246, and S247, where W243 was considered a crucial amino acid for the antagonistic property (Figure 55).

**Figure 56:**
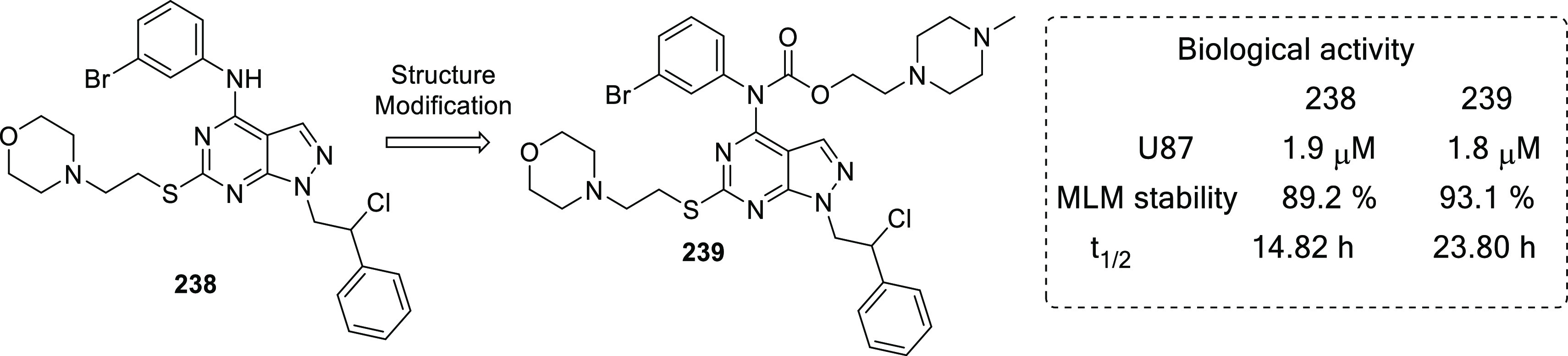
Pyrazolo[3,4-*d*]pyrimidine as potent anti-GBM agent.

Focusing on the PK properties of pyrazolo[3,4-*d*]pyrimidine, a group led by Schenone introduced 13 prodrugs from nine drugs bearing pyrazolo[3,4-*d*]pyrimidine as potential anti-GBM agents.^117^ All introduced compounds and their prodrugs were evaluated for *in vitro* ADME and biological assays, where **238** and **239** were found to be the most promising leads from the series. In studies, it was found that prodrug **239** showed significant results compared with **238** against the U-87MG cell line, with IC_50_ = 1.9 and 1.8 μM, respectively. Further evaluations revealed that **239**, converted into its parent form by the following hydrolysis process, showed a higher measured plasma concentration and prolonged the survival rate of mice in the GBM orthotopic mouse model. The group continued their efforts and developed a polymer formulation of compound **238**. Initially, **238** was screened over various GBM cell lines, where it potentially inhibited cell growth as follows (IC_50_): GIN8, 11.2 μM; GIN28, 7.7 μM; and GCE28, 7.2 μM. Furthermore, **239** was formulated in polymers using 2D inkjet printing, where a dispersion of 4 in Pluronic F-68, Tween 80, or PVPVA was found to be an efficient method; however, it showed comparable cytotoxicity to **238** in DMSO.^118^ Overall, pyrazolo[3,4-*d*]pyrimidines have shown magnificent results against GBM cell lines, and further developments might result in more efficient therapeutics against GBM (Figure 56).

**Figure 57:**
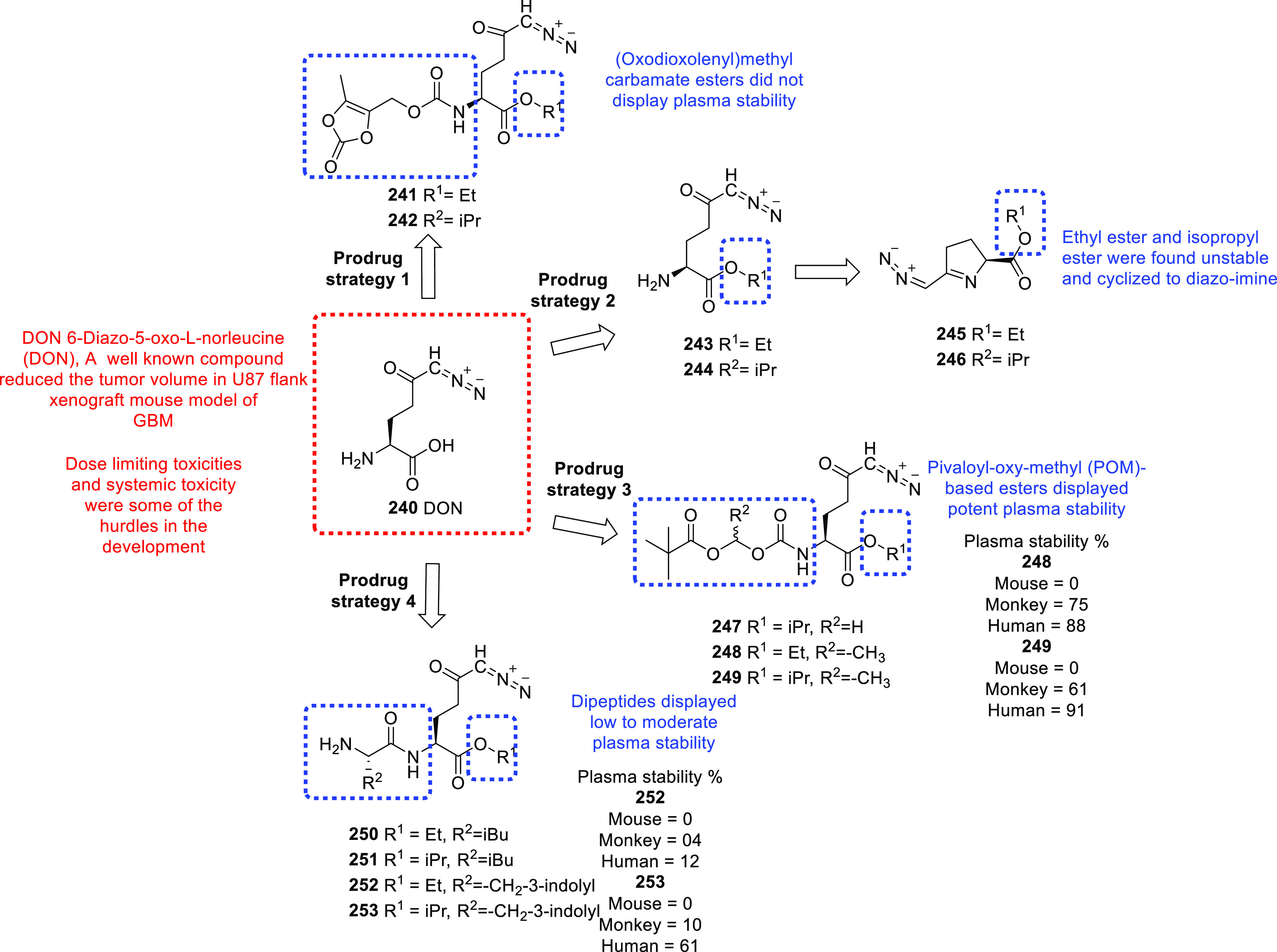
Identification of diazo-5-oxo-l-norleucine (DON) prodrugs for the treatment of GBM.

In 2016, Rais et al. identified prodrugs of 6-diazo-5-oxo-l-norleucine (DON) with improved CSF delivery to treat GBM.^119^ DON (**240**) is a glutamine mimic non-natural amino acid that inhibits *in vitro* glutamine-dependent human cancer cells, reduces the tumor size, and improves the survival rates. However, dose limitation and systemic toxicity are some of the obstacles in its development. Hence, the group explored the structure of DON (**217**), revealing that the furnished prodrugs **248** and **249** showed high plasma stability in monkeys and humans (Figure 57). Additionally, **249** displayed 10-fold enhancement in CSF delivery compared with DON. Overall, the defined strategy was found to be effective, providing an excellent opportunity to deliver DON to GBM patients.

**Figure 58:**
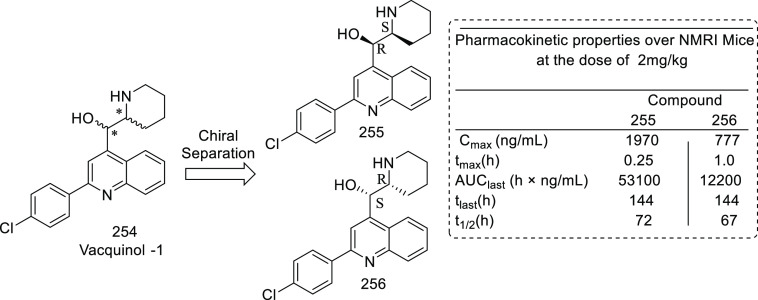
Vacquinol-1 stereoisomers for the treatment of glioma.

In 2016, Hammarström et al. described the distinct stereochemical features of the previously reported [2-(4 chlorophenyl)quinolin-4-yl](piperidin-2-yl)methanol (Vacquinol-1) (**254**) and delineated its oncolytic efficacy and *in vivo* PK properties.^120^ First, the *erythro* was separated from the *threo* racemates by HPLC using a standard-phase Kromasil silica column and was divided into two fractions. The first fraction (I) was separated again using semi-preparative HPLC and a Chiralcel OD-H column, while the second (II) fraction was isolated using a Chiralpak AD-H column, resulting in four enantiomerically pure fractions. All the fractions were evaluated in human patient-derived U3013 GBM cells using an ATP-based *in vitro* viability assay.The enantiomers obtained from the second fraction were more active than those from the first fraction, with IC_50_ = 3.5 and 3.8 μM (*erythro* enantiomers) and 9.9 and 10.5 μM (*threo* enantiomers), respectively. To evaluate the absolute configuration of compounds from the second fraction, single-crystal X-ray diffraction and Flack’s X chirality parameters were used, leading to the identification of **255** and **256**. Furthermore, the PK properties of the compounds were evaluated in male NMRI mice treated with a single dose of 20 mg/kg (orally (p.o.)) or 2 mg/kg (intravenously (i.v.)). The data suggested that **255** endowed good BBB crossing ability and was free from systemic or CNS toxicity. Furthermore, **255** was evaluated in a zebrafish model where no toxic effect was observed on zebrafish development even at the highest tested concentration of 50 μM. Additionally, **255** reduced tumor growth over U3013 human glioma cells labeled with cell tracker green zebrafish larvae, suggesting that **255** can inhibit human glioma cells without severe toxicity (Figure 58).

**Figure 59:**
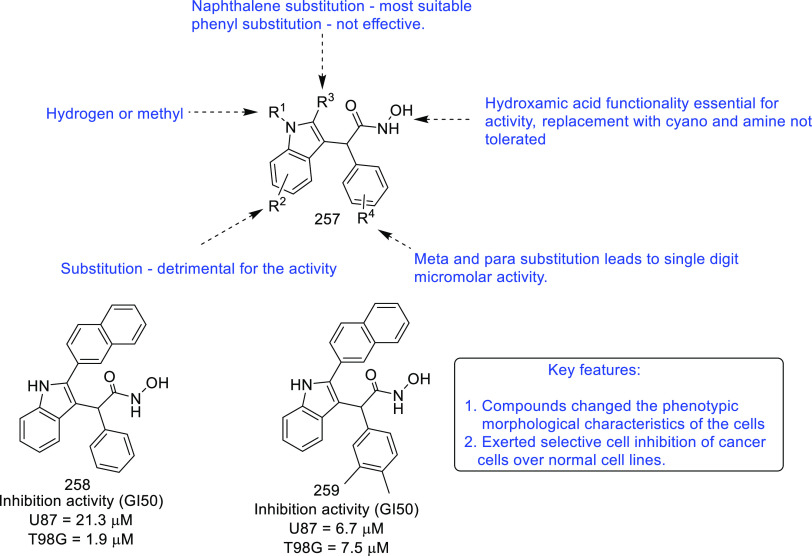
2-Aryl-2-(3-indolyl)acetohydroxamates active against MDR cancer cell line.

In 2015, Aksenov et al. disclosed a series of 2-aryl-2-(3-indolyl)acetohydroxamic acids that were active against multi-drug-resistant, GBM neurosphere stem-like, and apoptosis-resistant cells.^121^ The cytostatic properties of the hydroxamates were responsible for the anti-proliferative effects against apoptosis-resistant U373 GBM cells (Figure 59).

**Figure 60:**
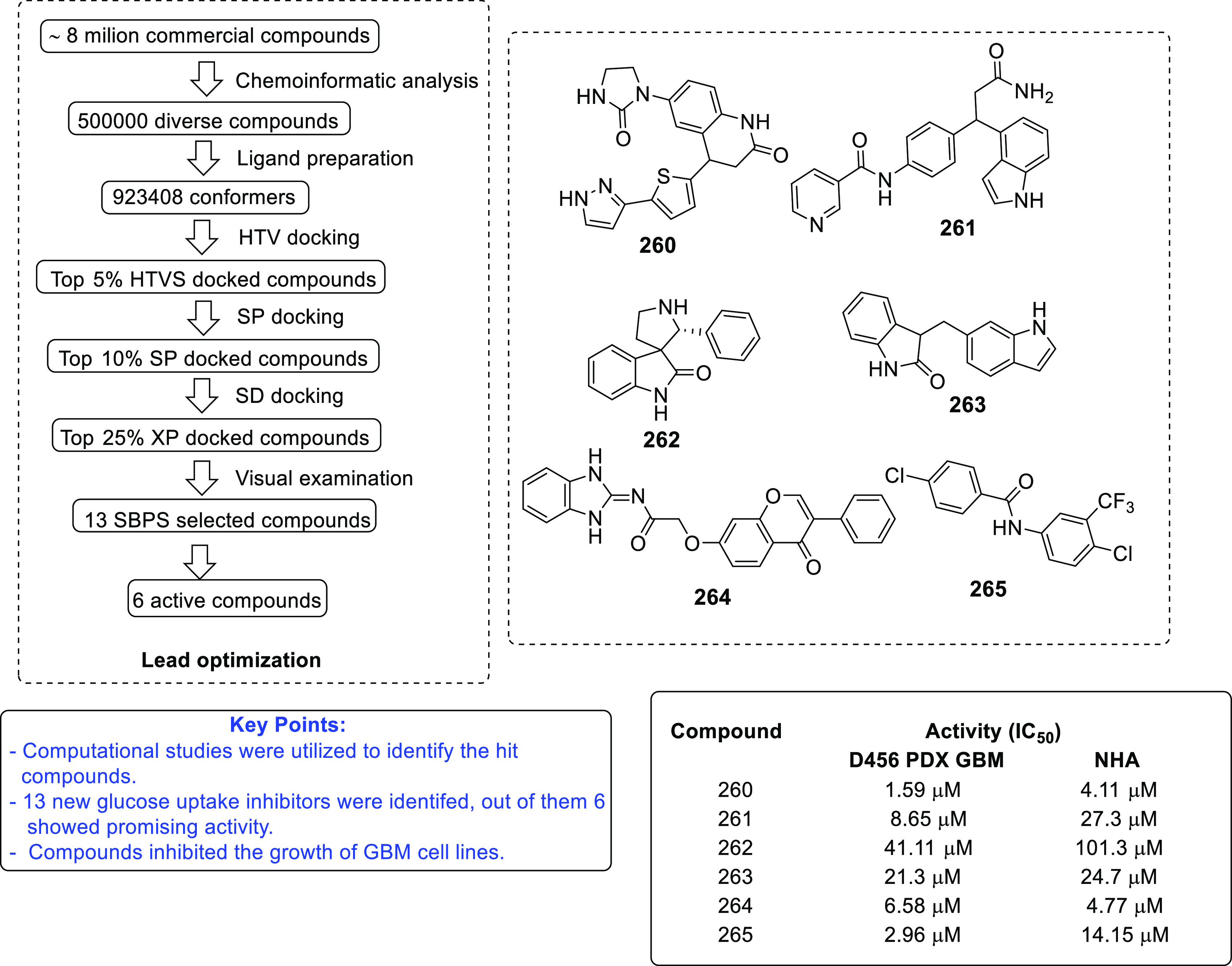
GULT inhibitors as anti-cancer agents.

To establish candidates for GULT inhibitors as potential therapeutics for GBM, Landis et al. screened a library of 500 000 compounds via structure-based virtual screening and identified 13 hit ligands.^122^ Based on the scaffold, the hits were categorized into four classes: indolinones/imidazolinones, isoflavone, dihydroquinolinones, and miscellaneous. The hits were screened over D456 GBM PDX cells, where six compounds inhibited cell growth with IC_50_ values in the range of 1.69–41.22 μM (Figure 60). The three most potent compounds from the library were further evaluated in normal human astrocytes and neurons, where all the tested compounds produced minimal toxicity. Compounds **260** and **261** were tested for glucose uptake inhibition over GBM PDX lines D456, GBM157, and GBM1016, and the results indicated that the compounds showed significant glucose uptake inhibitory potential. Overall, the findings indicate the selective anti-glioma effects of GULT inhibitors.

**Figure 61:**
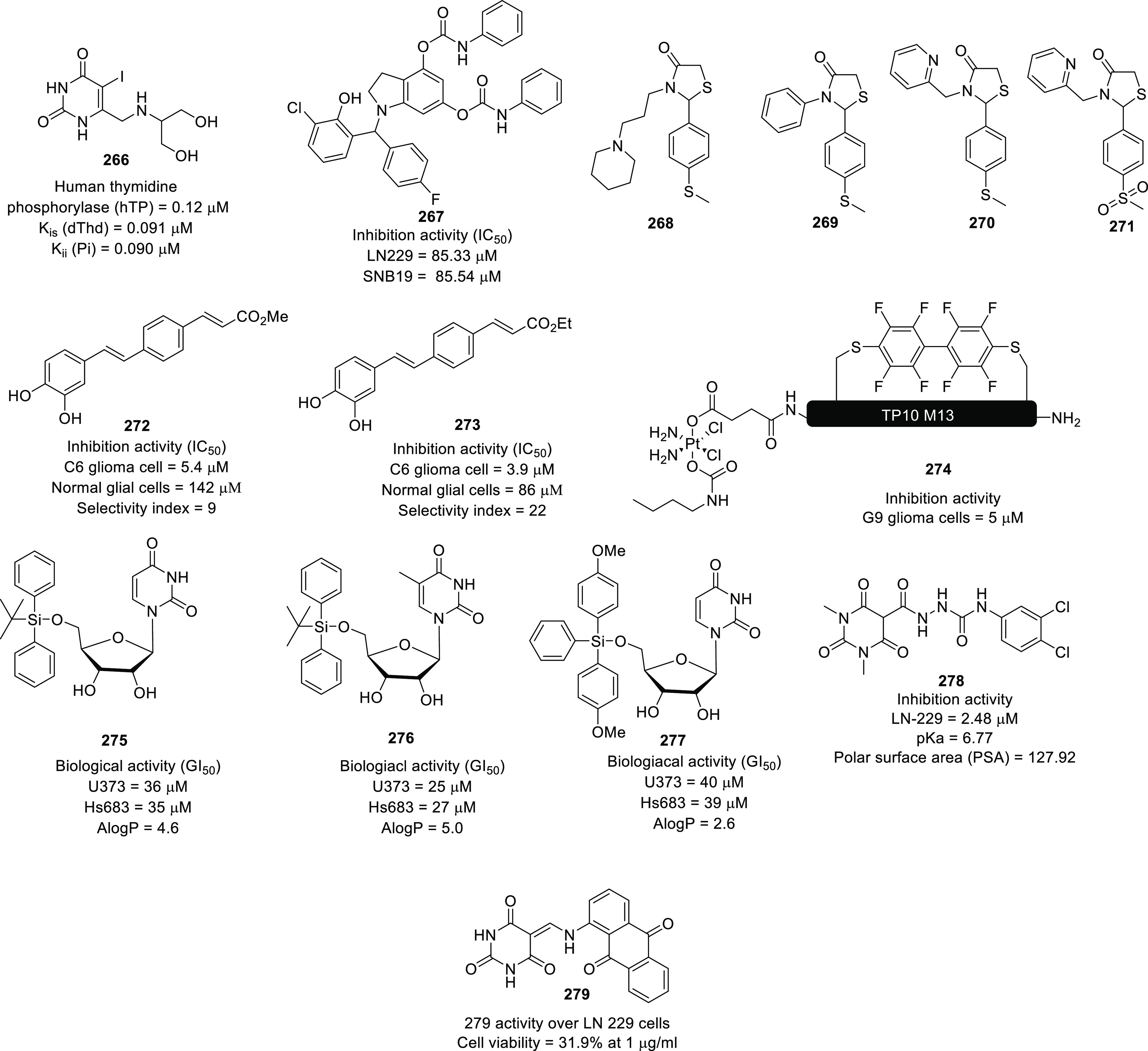
Various chemical structures as potential anti-GBM agents.

In 2019, de Moura Sperotto et al. reported a non-competitive human thymidine phosphorylase inhibitor **266** that demonstrated striking tumor growth reduction in a U-87MG human GBM model.^123^ In 2021, Nguyen et al. revealed a G protein-coupled receptor 17 (GPR17) **267** ligand that inhibited the growth of the LN229 glioma cell line with IC_50_ = 85.33 μM.^124^ In 2016, da Silva et al. reported thiazolidin-4-one-based compounds **268**–**271** as potential anti-GBM agents that reduced the viability of C6 cells by 61.2%, 52.2%, 48.0%, and 47.2%, respectively (Figure 61).^125^ In 2016, Shard et al. furnished styryl–cinnamate hybrids using Perkin–Heck reactions and evaluated them for *in vitro* cytotoxic effects against the C6 glioma cell line.^126^ Among the synthesized compounds, **272** and **273** showed potential cell growth inhibition, with IC_50_ = 5.4 and 3.9 μM, respectively. Further analysis revealed that the selected compounds promoted apoptosis through caspase-6, which was confirmed by Bax expression Western blot analysis. Additionally, metabolomics studies of **273** suggested that compounds reduced the levels of glutathione and other metabolites. In 2020, Fadzen et al. synthesized a perfluoroaryl macrocyclic peptide–platinum(IV) prodrug conjugate and evaluated its cell growth inhibitory effects against glioma stem-like cells using a CellTiter-Glo luminescent assay.^127^ The results of the assay indicated that the Pt(IV)-M13 conjugate (**274**) exhibited activity similar to that of cisplatin, with IC_50_ = 5 μM. Furthermore, the conjugates were evaluated for cell uptake and localization of G9 glioma stem cells, where the Pt(IV)-M13 conjugate (**274**) displayed significant improvement (drug uptake) and was accommodated in the cytosol. Additionally, pharmacokinetics and biodistribution studies were performed, and the results revealed that the conjugate was more stable in albumin and that more brain uptake was reported. Notably, a 15-fold higher amount of platinum was found in the brain after treatment (5 h) with the conjugate compared with that after cisplatin treatment (Figure 61). In conclusion, the conjugation of perfluoroaryl macrocyclic peptide with the platinum(IV) prodrug (cisplatin) (**274**) is an effective strategy to overcome the limitations of cisplatin. In 2016, Panayides et al. synthesized silyl- and trityl-substituted nucleosides that displayed promising U373 and Hs683 glioma cell growth inhibition with GI_50_ values in the range of 25–100 μM.^128^ Among the evaluated compounds, **276** was the most abundant in the series, with GI_50_ = 25 and 27 μM, respectively. Additionally, the lipophilicity value for **275**–**277** was evaluated, and the ALogP values were 4.6, 2.6, and 5.0, respectively. In 2016, Hron et al. synthesized a series of 1,3-diazinane-5-carboxamide analogs of 1,3-diazinane-5-carboxamide (merbarone analogs), and their anti-cancer activity was evaluated in the LN-229 GBM cell line.^129^ Among the synthesized compounds, **278** was the most potent in the series and showed activity at 2.48 μM (Figure 61).

In 2017, Pianovich et al. revealed a series of aminomethylidene-diazinanes as potential anti-GBM agents against the LN229 cell line.^130^ In the series, **279** was found to be the most promising, which reduced the cell viability to 31.9% at a concentration of 1 μg/mL (Figure 61).

**Figure 62:**
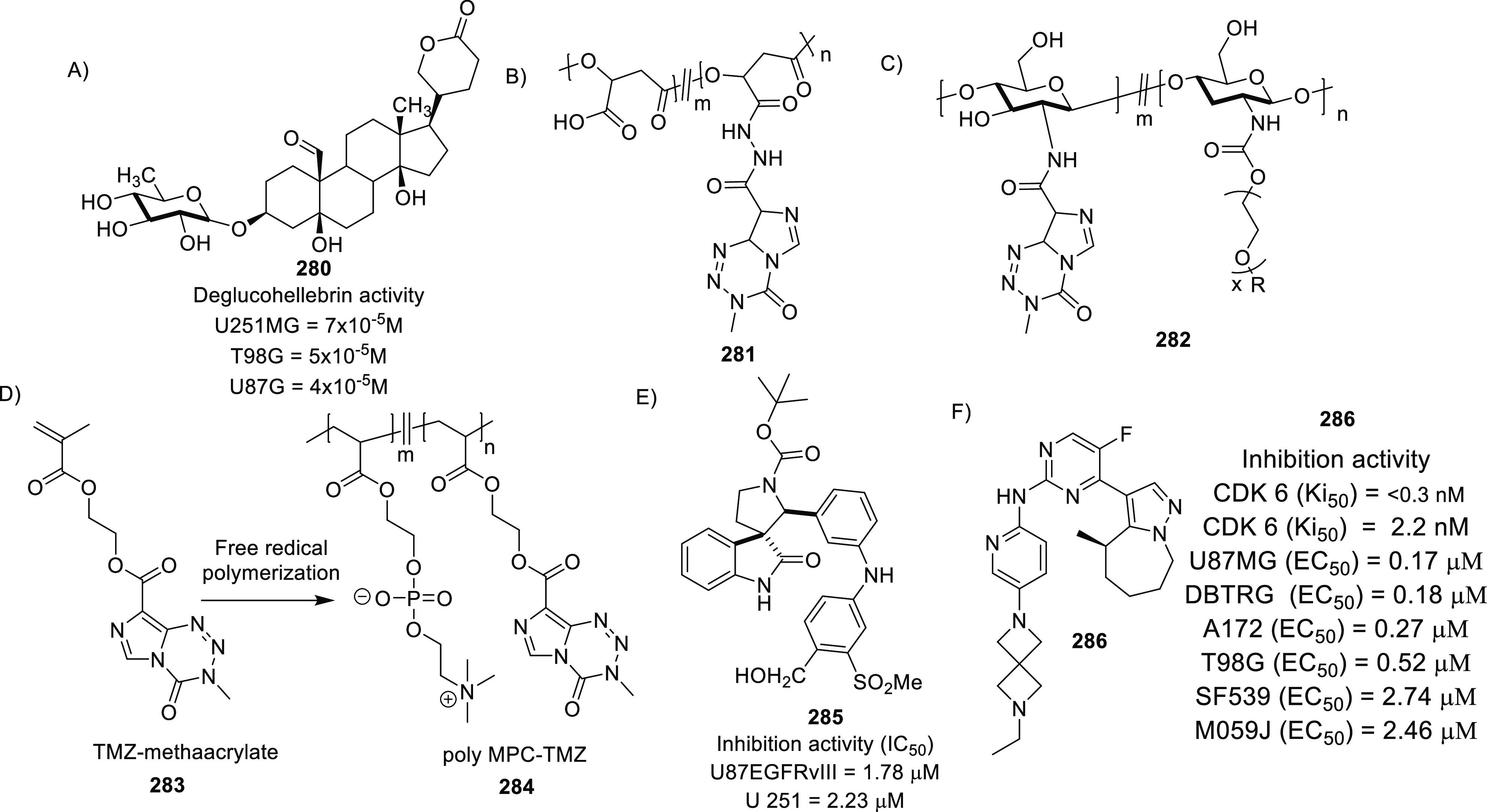
Various scaffolds and polymers for the treatment of GBM.

In 2020, Vartholomatos et al. investigated the effect of deglucohellebrin (**280**), a natural product obtained from the plant *Helleborus odorus* subsp. cyclophyllus (family Ranunculaceae), over three GBM cell lines.^131^ In studies, DHT significantly inhibited GBM cell viability, with IC_50_ = 7 × 10^–5^, 5 × 10^–5^, and 4 × 10^–5^ M over U251MG, T98G, and U-87G GBM cell lines, respectively. Further studies illustrated that DGH arrested the cell cycle at the G2/M phase, induced apoptosis, and did not show any cytotoxicity in the zebrafish model (Figure 62A). In addition to the new therapeutic discovery, efforts have been invested to improve the PK and pharmaceutical properties of the existing alkylating agent TMZ by utilizing various polymers.^132,133^ To enhance TMZ drugable properties, Patil et al. developed poly(β-l-malic acid)-based nanovehicles (**281**), which ameliorated the half-life of TMZ 3–4 times (5–7 h) compared with free TMZ (1.8 h) and significantly inhibited the growth of the GBM cell lines U-87MG and T98G (Figure 62B).^134^ Fang et al. published a report on the modification of TMZ with poly(ethylene glycol) (PEG)-chitosan as a prodrug (**282**).^135^ The developed prodrug prudently elongated the half-life 7-fold at physiological pH and inhibited the growth of the GBM cell lines U118, SF767, and GBM6, with IC_50_ = 86.5, 66.0, and 119.8 μM, respectively (Figure 62C).

Skinner et al. revealed a prodrug of TMZ equipped with poly(2-methacryloyloxyethylphosphorylcholine) (polyMPC) polymer (**284**) via free radical copolymerization with enhanced high drug loading (50 mol% or greater).^136^ Moreover, the prodrug showed excellent water solubility (>25 mg/mL) and provided stability to TMZ in aqueous medium (Figure 62D). Liver X receptor β (LXRβ) has emerged as a promising target against GBM; however, the development of selective agonists for LXRβ is onerous due to the highly homologous binding pockets of LXRα and LXRβ. The selectivity is only accomplished by targeting Val versus Ile, the only variance in the binding pocket. To overcome the selectivity, Chen et al. screened 9500 in-house libraries using machine-learning-based virtual screening, which resulted in 59 biologically relevant compounds with 13 LXRβ agonists.^137^ Further optimization efforts led to the identification of the selective LXRβ agonist 285, which selectively binds to LXRβ (IC_50_ = 86 μM) and inhibits the growth of the U-87EGFRvIII cell line, with IC_50_ = 1.78 μM. Moreover, **285** displayed significant anti-tumor activity over the *in vivo* xenograft model and showed promising PK properties (Figure 62E). In 2019, Bronner et al. revealed brain-penetrable CDK 4/6 inhibitors as potential anti-GBM agents.^138^ In the series, **286** was found to be a promising compound that inhibited various GBM cell lines in the low micrololar range (Figure 62F).

**Figure 63:**
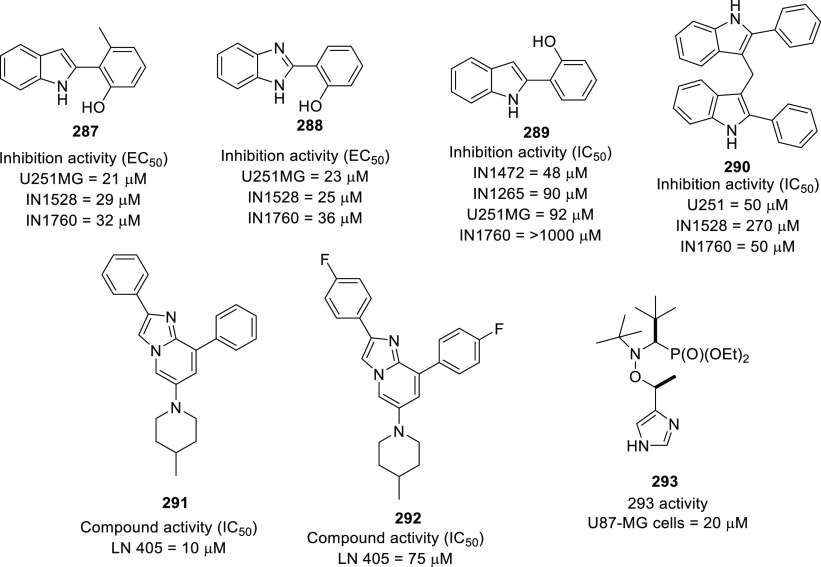
Various scaffolds as potential anti-GBM agents.

In 2018, Shrer et al. published a combination of phenols and indoles as potential anti-GBM agents.^139^ A series of compounds was introduced where **287** and **288** were found to be most potent against GBM cell lines, indicating that the combination of phenols and indoles can be utilized to develop new/novel anti-GBM agents. In 2013, Prabhu et al. carried out a preliminary investigation of various 2-arylindoles against GBM cell lines.^140^ A total of seven compounds were screened, of which **289** was found to be most promising against GBM cell lines, and the results are shown in Figure 63. In 2017, another group, Sherer et al., attempted a preliminary SAR investigation of indol-3-carbinol, which showed a potential impact on GBM cell lines.^141^ In the series, **290** showed favorable GBM cell growth inhibition in preliminary studies, indicating that indol-3-carbinol can be used as a promising lead in the development of anti-GBM agents. To identify new scaffolds against GBM, Güçlü et al. introduced a series of imidazopyridines that potentially inhibited the growth of LN-405 cells.^142^ Among the series, **291** and **292** were found to be the most promising compounds, with IC_50_ = 10 and 75 μM, respectively. In further studies, it was found that **291** and **292** arrested cell growth at the G0/G1 phase along with acceptable log BBB and Caco-2 permeability and are safe over the WS1 cell line (Figure 63). In 2019, Yamasaki et al. attempted a chemical engineering program over imidazole-containing alkoxamines that enhanced the homolysis rate of the C-ON bond of unstable alkoxamines.^143^ It was found that bond cleavage followed the protonation and/or methylation that led to notable anti-tumor activity. Among the series of 16 compounds, **293** was found to be most promising, with IC_50_ = 20 μM. In further studies, **293** displayed acceptable LogD_7.4_ and p*K*_a_ properties, which make it a magnificent lead for further development (Figure 63).

**Figure 64:**
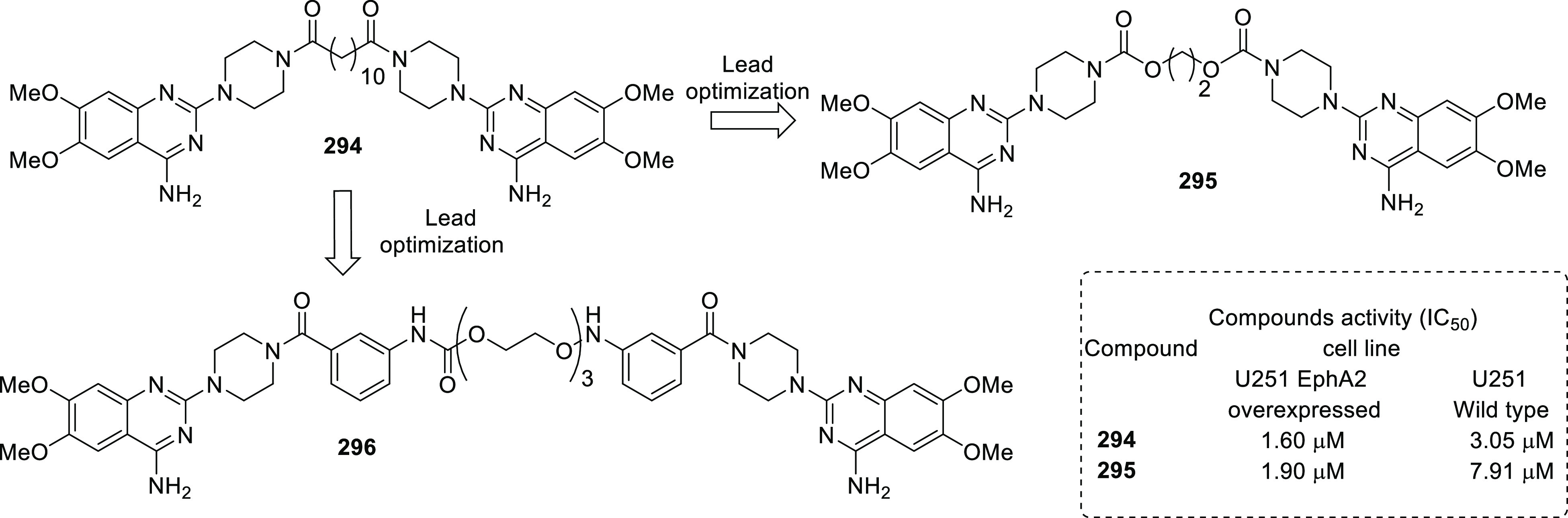
EphA2 agonist as potential anti-GBM agents.

In 2020, Orahoske et al. attempted to optimize the EphA2 agonist Doxazosin to identify the lead compounds against GBM.^282^ A medicinal chemistry campaign was carried out over Doxazosin (**294**), and a series of 27 compounds was introduced by utilizing alkylglycol polyethylene as linkers. All the synthesized compounds were evaluated over U251 EphA2 overexpressed and U251 wild-type GBM cell lines, where **295** and **296** were found to be the promising compounds in series which activity is shown in the figure. Moreover, both **295** and **296** induced the EphA2 phosphorylation which suggesting the EphA2 mediated anti-proliferative properties and make EphA2 as an effective target against GBM (Figure 64).

**Figure 65:**
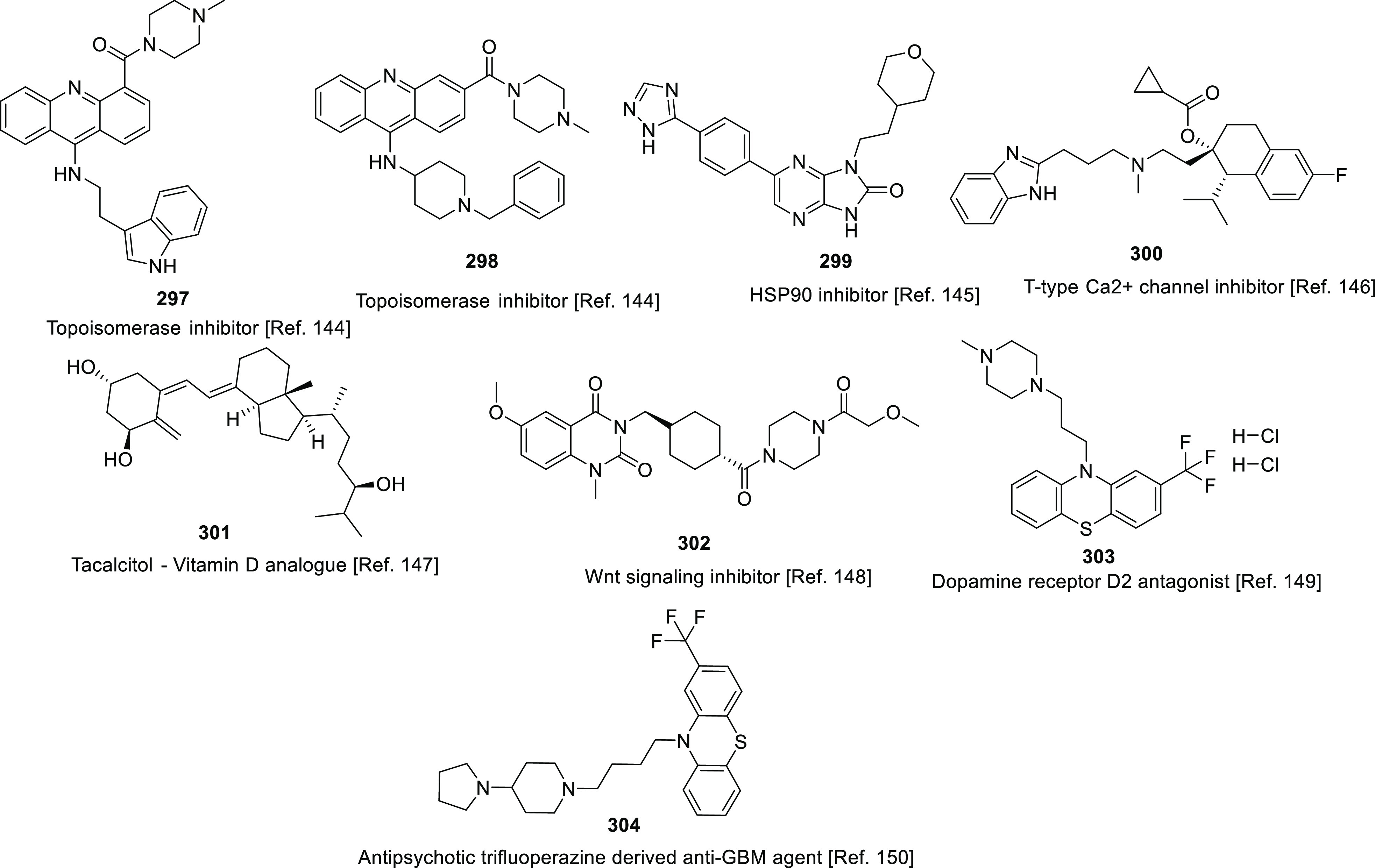
Anti-GBM agents.

The chemical structures of other promising anti-GBM agents belonging to this category are shown in Figure 65.^144−150^

## 5 Drug Delivery Approach

**Figure 66:**
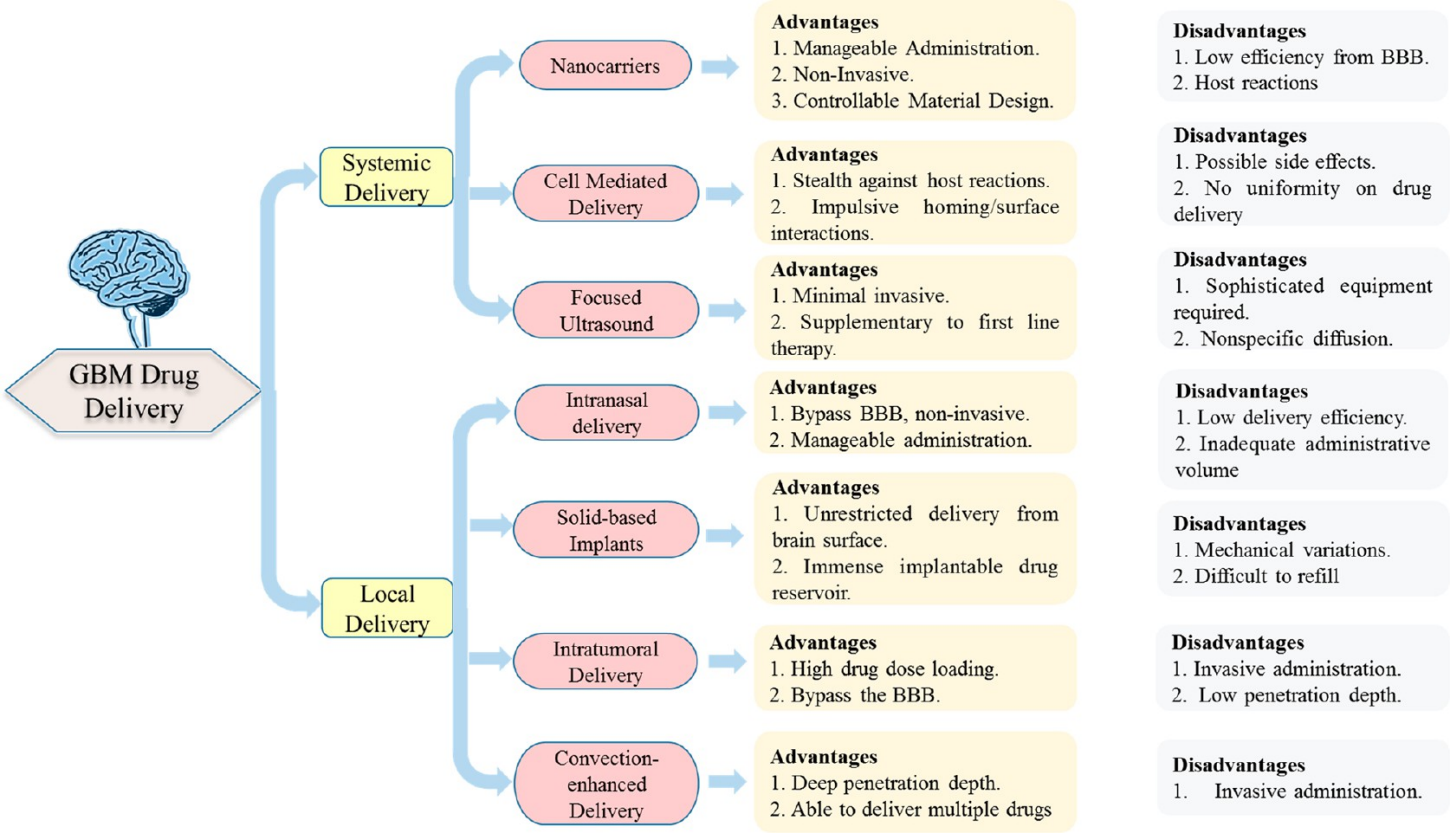
Different approaches for drug delivery to brain.

Logical design of small-molecule inhibitors followed by optimization of efficient and robust synthetic routes is of paramount importance; however, equally important is the selection of the approach to deliver the drugs to the brain. This section will present an overview of the drug delivery strategies that can be leveraged to serve the aforementioned purpose (Figure 66). Table 4 summarizes recent advances in GBM drug delivery.

**Table 4:** Recent Advances in GBM Drug Delivery

formulation	details
nanomaterials	Nanomaterials have demonstrated promise as carriers for therapeutic agents for GBM through an enhanced permeability and retention (EPR) effect.^552,553^ However, some investigations demonstrating unfavorable results, such as poor BBB penetration and EPR-mediated accumulation of nanomaterials in solid brain tumors, create doubts regarding their utility.^554^ In this context, several advancements have been made for transcytosis across the BBB for GBM treatment:
	(1) Gold nanoparticles (AuNPs) endowed with the ability to undergo a legumain-triggered click cycloaddition could selectively accumulate in the glioma site, enabling the capacity to precisely diagnose the glioma.^555^
	(2) A study reported that tandem nanomicelles co-functionalized with Angiopep-2 and cell-penetrating peptides demonstrated high glioma cell selectivity and long blood circulation times, coupled with enhanced BBB permeation.^556^
	(3) A study reporting the sequential targeting of BBB/BBTB and brain tumor cells with STICK nanoparticles revealed the ability to transpass the BBB/BBTB via glucose-transporter-mediated transcytosis.^556^
	(4) A study reporting that neutrophils carrying liposomes that contain paclitaxel (PTX) can penetrate the brain and efficiently slow the recurrent growth of tumors.^557^
	(5) A study reported that the strategy of camouflaging nanoparticles with brain metastatic tumor cell membranes exhibited favorable results, as the resulting biomimetic nanoparticles displayed superb BBB penetration and effective suppression of tumor growth.^558^

focused ultrasound (FUS)	• Explorations on microbubble-mediated FUS-mediated disruption of the BBB^559^ reveal that the combination of ultrasound with microbubbles is safe, as the treatment has not been found to be associated with neuronal damage or long-term vascular damage.^560^
	• FUS has demonstrated significant promise for GBM and DIPG. In a clinical MRI-guided FUS study, a several-fold enhanced concentration of TMZ in sonicated versus unsonicated tumor tissue was evidenced.^561^
	• An implantable ultrasound device (CarThera SonoCloud) in combination with carboplatin (systemically administered) was evaluated in a phase I clinical investigation. The study results indicated an improved progression-free survival in 11 GBM patients.^562,563^
	• The combination of FUS with immunotherapy also appears to be promising, as immune cells are unable to cross the BBB owing to the low expression of leukocyte adhesion molecules in CNS endothelial cells. Upon the disruption of the BBB with FUS, it is anticipated that immune cells can extravasate.^564,565^

cell-mediated delivery	• Recent development in the field of targeted DDS for GBM involves the cell-mediated delivery of anti-cancer drugs and nanomaterials. Unlike conventional tumor targeting, the methodology of cell-mediated targeting is based on interactions of the BBB or the tumor with various surface proteins on the outer membranes.^566,567^
	• Spurred by the inherent ability of neutrophils to traverse the BBB, Xue et al. explored cell-mediated delivery of paclitaxel for the treatment for recurrent malignant glioma.^557^ The study methodology involved the internalization of paclitaxel-loaded cationic liposomes within isolated neutrophils. The outcome of the study was favorable, as slower recurrence of tumor growth and remarkable improvement in survival rate were evidenced with the cell-mediated drug delivery.
	• Recently, Wang et al. evaluated a logical approach of leveraging the BBB-penetrating ability of metastatic cells via concerted interactions between their surface proteins and the receptors of vascular endothelial cells.^558^ For the study, membranes extracted from metastatic B16F10 and 4T1 cell lines were leveraged for the encapsulation of polycaprolactone (PCL) nanoparticles loaded with indocyanine green (ICG). Resultantly, 11-fold higher fluorescent signals were emitted by the membrane-coated nanoparticles administered to orthotopic glioma-bearing mice than the nanoparticles without a membrane coating.

intra-nasal delivery	• The nasal cavity provides access to the brain, making intranasal delivery as another option to overcome the BBB. Lately, the intra-nasal delivery of nanoparticles has garnered significant attention and is conceived to overcome the hurdles associated with conventional intra-nasal drug administration methods.^568,569^
	• Recently, delivery of chitosan nanoparticles loaded with siRNAs by targeting galectin-1 was reported. The results of the exploration revealed significant decreases in the expression of galectin-1 in the tumor microenvironment. Overall, these results were quite optimistic and strengthened the candidature of intra-nasal gene delivery using nanoparticles as a prudent methodology for the treatment of GBM.^570^
	• Another recent investigation reported the intra-nasal delivery of targeted polyfunctional gold–iron oxide nanoparticles loaded with therapeutic microRNAs. The novel theranostic nanoformulation was intended to be leveraged for combined theranostic multi-modality imaging as well as pre-sensitization of GBM to TMZ. The results of the study demonstrated a significant increase in survival of mice co-treated with the nanoformulation compared to the untreated group.^571^

convection enhanced delivery (CED)	• CED is a delivery approach that utilizes the applications of a microcatheter to deliver the drug. External pressure gradient is generated via a motor-driven pump that induces fluid convection in the brain, leading to deeper penetration of drugs at the target tissue.^572^
	• Recently, a study demonstrated that CED-administered cisplatin-loaded nanoparticles remarkably enhanced the survival rate in a GBM rat brain tumor model.^573^
	• Another study results reported that magnetic nanoparticles to deliver O6-benzylguanine, an MGMT inhibitor, administered by CED, exhibited significant distribution within the mouse brain, along with a significant increase in median survival rate.^574^
	• A recent exploration reported remarkable distribution of biodegradable PNPs designed for the delivery of herpes simplex virus type I thymidine kinase (HSVtk) DNA administered via single CED infusion. In addition, nanoparticles administered via CED in combination with the systemic administration of ganciclovir demonstrated increased survival rates in tumor-bearing rats.^575^ Collectively, the results indicate the increased efficacy of gene therapy in combination with CED.
	• At present, CED is undergoing clinical stage investigations for GBM and DIPG.^576,577^

intra-arterial drug delivery	• This approach involves the direct administration of the drug into an artery in the proximity of the tumor. Several explorations were conducted to evaluate the potential of intra-arterial drug delivery in GBM patients. Despite some favorable results in the context of survival via treatment with nimustine, bevacizumab, or carboplatin in combination with other conventional chemotherapy, toxicity and low drug efficacy were identified as hurdles limiting this delivery approach.^332,578−580^

solid implant-based drug delivery	• GLIADEL wafers (solid implants) are FDA-approved biodegradable wafers containing the alkylating drug carmustine, and these wafers are indicated for the treatment of GBM. GLIADEL wafers release cytotoxic concentrations of carmustine into the tumor resection cavity, wherein after exposure to the aqueous environment of the resection cavity, the anhydride bonds in the copolymer are hydrolyzed. After hydrolysis the wafers releases carmustine, carboxyphenoxypropane, and sebacic acid into the surrounding brain tissue.^581^
	• Owing to the drawbacks associated with the use of the wafers as well as other solid implant-based therapy such as limited penetration, intra-cavity migration, and rapid drug release,^582,583^ efforts have been invested to negate the aforementioned challenges and pinpoint some solutions. A novel implantable device composed of wireless electronics integrated with a drug–polymer reservoir exemplifies one such development.^584^ Also, deeply located GBM cells were recently targeted via a solid implant composed of PCL nanofibers and a drug-conjugated hydrogel.^585^

intra-tumoral delivery (direct injection into the tumor site)	• Recently, some intra-tumoral formulations for the treatment of GBM have been reported. Brain-penetrating nanoparticles loaded with paclitaxel exemplifies such formulation that demonstrated improved drug distribution within GBM tissue and enhanced therapeutic efficacy.^586^
	• Another study reported that injectable lipid nanocapsules (hydrogel loaded with 4-(*N*-lauroyl)-Gemcitabine) demonstrated sustained release of drug for a month and exhibited promising therapeutic potential in the context of prevention of recurrence.^587^
	• A study aimed at the evaluation of cocktails (chemoimmunotherapy and hydrogel composites with the property of *in situ* gelation) revealed increased survival rate coupled with significant anti-tumor immune responses in an orthotopic brain tumor model *in vivo*.^588^

## 6 Conclusions

Glioblastoma is a devastating brain cancer, and the treatment strategies currently employed for its treatment do not significantly improve the overall survival of GBM patients. Being highly angiogenic, these invasive tumors often acquire resistance to chemotherapy. Unfortunately, immunotherapy has not demonstrated remarkable efficacy because the brain is an immune-privileged tissue and GBM is considered a cold tumor.^22^ Additionally, the BBB leads to restricted uptake of drugs by the brain, further limiting the therapeutic options. Furthermore, the documented progress of brain tumor drug discovery programs has created apprehension in researchers because brain tumor treatment has been associated with precedential failures, as demonstrated by the low FDA approval rate of CNS drugs compared with non-CNS drugs. However, an increasing number of studies have focused on identifying effective targets that can counter the intra-tumoral molecular heterogeneity of GBM via BBB permeable therapy. Notably, substantial progress has been made in understanding the mechanisms involved in regulating GBM initiation and progression. Subsequently, the findings have facilitated the construction of tractable anti-GBM agents that can follow an uninterrupted development path, can be steered to advanced stage clinical settings, and can be translated to more personalized, cell-type-specific, effective, and safe treatments for GBM in the near future.

Notably, to overcome the above-mentioned challenges and expand the list of therapeutic options for GBM, many studies have focused on small-molecule inhibitors in the past decade. Thus, most of the obstacles encountered in GBM drug discovery have been addressed by the implementation of robust drug design strategies to construct small-molecule inhibitors with optimistic preclinical/preliminary profiles. Several targets have been validated to treat GBM, such as PI3K, FAK, HDAC, HIF, TSPO, tubulin, IDH, and PDI. Appreciably, medicinal chemists have demonstrated marked proficiency in designing and furnishing new scaffolds using rational strategies and have leveraged various heterocycles ranging from monocyclic to fused rings. Drug design strategies have been adequately embellished with structural engineering programs determining the impact of scaffold installation, bioisosteric replacement, structural simplification, structural rigidification, stereoelectronic variation, and other subtle structural variations on the activity. Chemists have not merely relied on the concept of single targeting agents to design new chemical tools for GBM treatment but have also expanded the drug design approaches to multi-targeting agents, modulating the simultaneous inhibition of more than one target in GBM, along with the concept of degraders (PROTACs). Some specific studies covered in this compilation exemplify the above-mentioned efforts of medicinal chemists, such as the following: (i) the accommodation of memantine in the HDAC inhibitory structural template to attain CNS-penetrating hydroxamic acids; (ii) the design of selective isoform inhibitors of PI3K to extract amplified anti-glioma efficacy; (iii) the aptamer functionalization of nanosystems to target GBM through the BBB; (iv) the pragmatic design of HDAC6 biased inhibitors based on the enhanced expression of HDAC6 isoforms in GBM; (v) the identification and modification of metabolic spots (vulnerable sites) of the potent anti-GBM agent to confer suitable PK properties; (vi) uncaging an inactive precursor of vorinostat by heterogeneous Pd catalysis in glioma cells; (vii) exploiting the prodrug strategy to design adducts of 6-diazo-5-oxo-L-norleucine (DON) with improved CSF delivery; (viii) the preliminary exploration of the rationally constructed dual HDAC/LSD1 inhibitor Corin for the treatment of DIPG, an incurable pediatric cancer; (ix) conventional structure-based virtual screening to design selective anti-glioma effects of GULT inhibitors; (x) establishing the BET degrader ZBC260 as a potent inhibitor of tumor progression and stem-cell-like cells (GBM); and (xi) investigating radiolabeled olaparib as a bio-imaging tool for glioma detection. The findings of the above-mentioned studies combined with other approaches encompassed in this Perspectove represent valuable information that can be leveraged to further numerous pursuits in this direction.

Owing to the large number of scaffold furnishment programs conducted recently, the pre-clinical pipeline of anti-GBM drugs comprises numerous candidates that appear to be suitable chemical tools capable of overcoming the obstacles of the anti-GBM drug discovery process and should be exhaustively explored to develop a therapeutic for GBM in the near future. Some of the agents identified through the above-mentioned scaffold construction approaches have already entered clinical trials, and some of them are expected to emerge as effective therapeutics for GBM. Overall, this Perspective highlights the significant advancements in the field of anti-GBM drug discovery with clear-cut knowledge of the challenges associated with the development of CNS drugs, particularly the ideal physicochemical properties required by chemotherapeutics for GBM. Although the stage appears to be set for the near future, efforts must be precisely channeled toward exploiting the promise demonstrated by the numerous studies covered in this Perspective. Given the historical failure associated with the clinical advancement of GBM drugs, the challenge is relatively more stringent compared with that encountered in other malignancies. Thus, the expertise of interdisciplinary teams composed of medicinal chemists, organic chemists, biologists, and formulation chemists along with researchers’ well-versed computational aspects of drug design would be required to steer the clinical progress of the candidates covered in this Perspective.

## Abbreviations Used

ABL: Abelson murine leukemia viral oncogene homolog 1
ALK: anaplastic lymphoma kinase
BBB: blood–brain barrier
BBTB: blood–brain–tumor barrier
BET: bromodomain and extra-terminal domain
CDK: cyclin-dependent kinase
CED: convection enhanced delivery
CLK: CDC-like kinase
CNS: central nervous system
CSC: cancer stem-like cell
CSF: cerebrospinal fluid
CSF1R: colony-stimulating factor 1 receptor
CXCR: C-X-C motif chemokine receptor
DDS: drug delivery system
DIPG: diffuse intrinsic pontine glioma
DNA: deoxyribonucleic acid
DRD: dopamine receptor D
DYRK: dual-specificity tyrosine-regulated kinases
ECM: extracellular matrix
ECS: extracellular space
EGFR: epidermal growth factor receptor
EZH2: enhancer of zeste homolog 2
FAK: focal adhesion kinase
FGFR: fibroblast growth factor receptor
FLT3: FMS-like receptor tyrosine kinase
GBM: glioblastoma
GPCR: G protein-coupled receptor
GSC: glioblastoma stem cell
HER: human epidermal growth factor receptor
HIF: hypoxia inducible factor
HSP: heat shock protein
HDAC: histone deacetylase
HML: human mouse liver microsome
IDH: isocitrate dehydrogenase
IDO: indoleamine 2,3-dioxygenase
IGF1R: insulin-like growth factor 1 receptor
IL: interleukin
JAK1: janus kinase 1
KIT: kit proto-oncogene
LSD1: lysine-specific demethylase 1
MAPK: mitogen-activated protein kinase
MDM2: murine double minute-2
MET: met proto-oncogene
MGMT: O-6 methylguanine-DNA methyl transferase
MKK: mitogen-activated protein kinase kinase
MLM: mouse liver microsome
MMP: matrix metalloproteinase
MTIC: 3-methyl(triazen-1-yl)imidazole-4-carboximide)
mTOR: mechanistic target for rapamycin kinase
NF-κB: nuclear factor-κB
PARP: poly(ADP-ribose) polymerase
PDGFR: platelet-derived growth factor receptor
PDI: protein disulfide isomerase
PDK1: 3-phosphoinositide-dependent kinase 1
PET: positron emission tomography
PI3K: phosphoinositide 3-kinase
PLD: phospholipase D
PLK1: polo-like kinase 1
PNP: polymeric nanoparticle
RAF: Raf proto-oncogene
RET: rearranged during transfection
ROS: reactive oxygen species
RTK: receptor tyrosine kinase
SAR: structure–activity relationship
SMO: smoothened frizzled class receptor
SRC: src proto-oncogene
STAT-3: signal transducer and activator of transcription 3
STK: serine/threonine-specific protein kinase
TGF-β2: transforming growth factor beta-2
TIE2: tyrosine-protein kinase receptor
TMZ: temozolomide
TSPO: translocator protein
VEGFR: vascular endothelial growth factor receptor
